# Diiron(I) Complexes with Promising Anticancer Properties Obtained by CO-To-Isocyanide Substitution of [Fe_2_Cp_2_(CO)_4_]

**DOI:** 10.1021/acs.inorgchem.6c03964

**Published:** 2026-09-24

**Authors:** Sara Benetti, Giulia Salluce, Axia Marlin, Tommaso Nottoli, Giulio Bresciani, Filippo Lipparini, Tiziana Funaioli, Tarita Biver, Pierre Mesdom, Stefano Zacchini, Massimo Guelfi, Lorenzo Biancalana, Gilles Gasser, Fabio Marchetti

**Affiliations:** † 9310 University of Pisa, Department of Chemistry and Industrial Chemistry, Via Moruzzi 13, 56124 Pisa, Italy; ‡ Chimie ParisTech, PSL University, CNRS, Institute of Chemistry for Life and Health Sciences, Laboratory for Inorganic Chemical Biology, 11 Rue Pierre et Marie Curie, 75005 Paris, France; § The Centre for Instrument Sharing of the University of Pisa (CISUP), Lungarno Pacinotti 43/44, 56126 Pisa, Italy; ∥ University of Bologna, Department of Industrial Chemistry “Toso Montanari”, Via Gobetti 85, 40129 Bologna, Italy

## Abstract

The diiron­(I) complexes [Fe_2_Cp_2_(CNR)_3_{CN­(Me) (R)}]­[CF_3_SO_3_] (**[2a-b]­[CF**_**3**_ **SO**_**3**_ **]**, R = Xyl, Cy; Xyl = 2,6-C_6_H_3_Me_2_, Cy = cyclohexyl) and [Fe_2_Cp_2_(CNR)_2_{CN­(Me) (R)}_2_]­[CF_3_SO_3_]_2_ (**[3a-c]­[CF**_**3**_ **SO**_**3**_ **]**_**2**_, R = Xyl, Cy, Me) were synthesized in moderate-to-high yields from [Fe_2_Cp_2_(CO)_4_]. They were characterized by IR and NMR spectroscopy and single-crystal X-ray diffraction in five representative cases. The electrochemical behavior of **[2a]­[CF**_**3**_ **SO**_**3**_ **]** and **[3a]­[CF**_**3**_ **SO**_**3**_ **]**_**2**_ was investigated by cyclic voltammetry and IR-spectroelectrochemistry, assisted by DFT calculations. UV–vis and ^1^H NMR studies showed ≥84% of each complex remaining intact after 72 h of incubation at 37 °C in aqueous media. Complexes **[3a-c]­[CF**_**3**_ **SO**_**3**_ **]**_**2**_ displayed significant aqueous solubility, and **[2b]­[CF**_**3**_ **SO**_**3**_ **]** and **[3a-b]­[CF**_**3**_ **SO**_**3**_ **]**_**2**_ exhibited amphiphilic character (−1 < Log *P*_*ow*_ < +1). The monocationic species **[2a-b]­[CF**_**3**_ **SO**_**3**_ **]** exerted strong cytotoxicity in A2780, A549, and U87 cancer cell lines, with **[2a]­[CF**_**3**_ **SO**_**3**_ **]** showing IC_50_ values in the nanomolar range. Among dicationic complexes, **[3a]­[CF**_**3**_ **SO**_**3**_ **]**_**2**_ exhibited significant activity despite limited cellular uptake, whereas **[3b-c]­[CF**_**3**_ **SO**_**3**_ **]**_**2**_ were inactive. Compounds **[2a]­[CF**_**3**_ **SO**_**3**_ **]**, **[2b]­[CF**_**3**_ **SO**_**3**_ **]** and **[3a]­[CF**_**3**_ **SO**_**3**_ **]**_**2**_ impaired mitochondrial bioenergetics without detectable elevation of intracellular ROS levels in both A549 and U87 cells. No significant interaction with double-stranded DNA was detected by ethidium bromide displacement assays. Conversely, fluorescence titrations with bovine serum albumin indicated the formation of 1:1 adducts for **[2a]** ^**+**^ and **[3a]** ^**2+**^.

## Graphical abstract

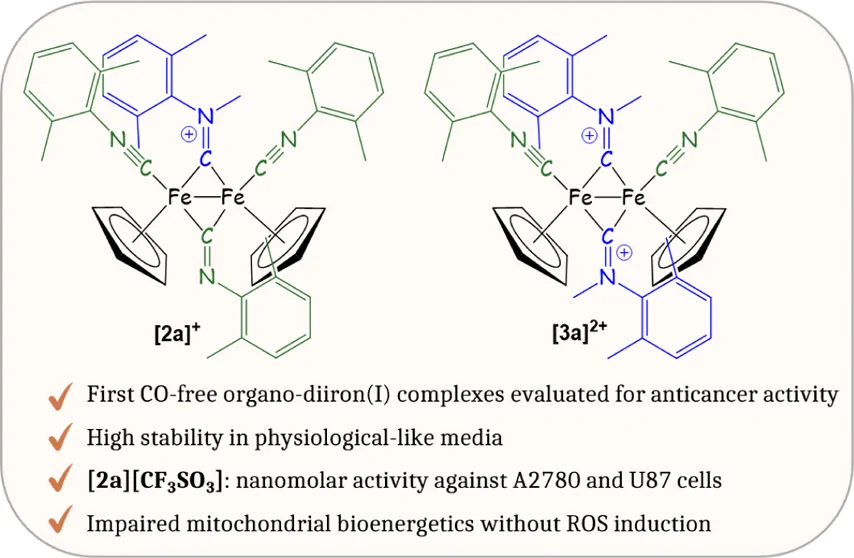

## 1 Introduction

The unique properties of transition metals, such as the availability of multiple oxidation states and coordination environments, confer a level of structural versatility to metal-based pharmaceuticals that is unattainable with purely organic compounds.^1,2^ Currently, a few platinum complexes are employed as first-line treatments for a wide range of tumors.^3,4^ However, despite their effectiveness, their use is often accompanied by severe side effects and the gradual development of drug resistance.^5,6^ As a result, significant research efforts have been directed toward the discovery of new metal-based anticancer compounds to overcome these limitations,^7,8^ including innovative platinum drug candidates,^9^ as well as various promising titanium, ruthenium, gold, iridium and other metal complexes.^1,10–12^ In general, the design of new complexes requires careful attention to avoid drawbacks that may lead to systemic toxicity in vivo. These include uncertain speciation in physiological media arising from insufficient solubility^13^ or low stability,^14–16^ intrinsic metal toxicity,^17^ and excessive drug lipophilicity (octanol/water partition coefficient, Log *P*_*ow*_, values higher than 3 have been regarded as critical).^18–20^

Iron, an endogenous element and therefore relatively less toxic than nonendogenous heavy metals, is an attractive choice for metal-based drug development,^21,22^ and organoiron­(II) complexes, including variably substituted ferrocenes and half-sandwich cyclopentadienyl derivatives, have emerged as promising anticancer agents.^23–25^ Their activity has often been associated with increased intracellular production of reactive oxygen species (ROS).^26^

Our research aims to improve the properties of iron-based anticancer compounds, focusing on diiron complexes. The advantages of this approach stem from the increased number of coordination sites and the unusual reactivity patterns enabled by the cooperative dimetallic core,^27,28^ offering greater structural diversity and thus fine control over physicochemical and biological properties. Starting from the commercially available [Fe_2_Cp_2_(CO)_4_] (Fp_2_, Cp = η^5^–C_5_H_5_), a straightforward and broadly applicable two-step reaction provides access to a family of cationic aminocarbyne complexes (Scheme 1a–b, structure **B**).^29,30^ Interestingly, mononuclear analogues, namely monoiron cyclopentadienyl aminocarbyne complexes featuring the FeCp­(≡CNR) platform, remain unknown because isocyanide alkylation is not feasible in these systems.^31^

**Scheme 1:**
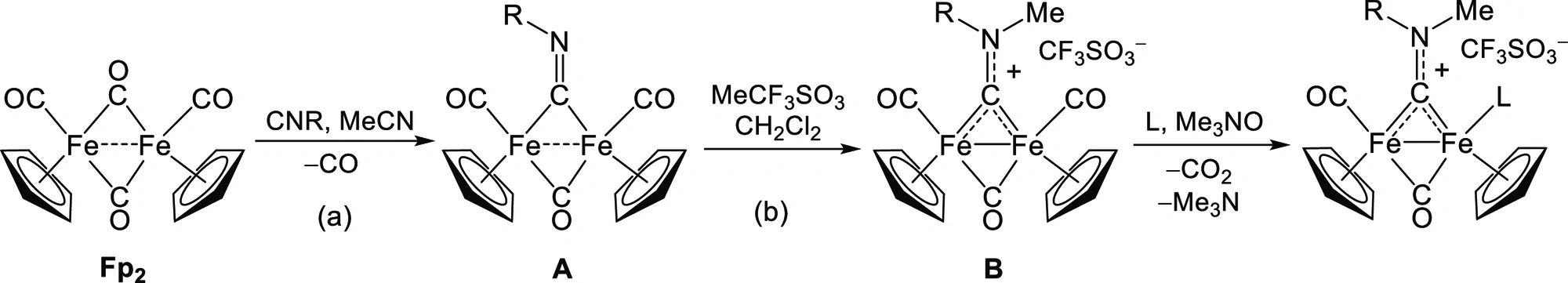
Synthesis of Mono-Isocyanide Diiron Complexes **A** From the Commercial Precursor Fp_2_ by CO/CNR Substitution, Followed by Methylation to Afford Aminocarbyne Complexes **B** (R = Alkyl or Aryl) and Subsequent Replacement of a Single CO Ligand by Neutral Donor Ligands (L = Pyridines, Phosphines or Isocyanides)[Fn s1fn1]

a
**Fp**_**2**_ and **A** exhibit fluxional behavior in organic solvents, involving rapid rearrangement of the Fe_2_Cp_2_ framework together with exchange of CO and CNR ligands between terminal and bridging coordination modes^42^.

Complexes of type **B** yielded promising results in terms of both antiproliferative activity and selectivity toward cancer cells.^32^ The mechanism of action appears to involve intracellular disruption of the organometallic framework. This process leads to the release of carbon monoxide, which may contribute to the therapeutic activity,^33,34^ together with reactive iron­(I) species that perturb cellular redox homeostasis.

Some dicarbonyl derivatives bearing P-^35^ or *N*- donor ligands^36^ are directly available from **B** using trimethylamine-*N*-oxide (Scheme 1c),^37^ and have displayed significantly improved activity. This demonstrates that appropriate modification of the iron coordination sphere through CO substitution represents a viable strategy for optimizing the anticancer profile of diiron aminocarbyne compounds. Consistent with the fact that the vast majority of reported [Fe_2_Cp_2_(CO)_4_] derivatives still contain carbonyl ligands,^29,38–40^ *noncarbonyl* diiron­(I) complexes have never been investigated as anticancer agents, to the best of our knowledge.^41^

This background prompted us to investigate carbonyl-free complexes, a modification expected to significantly influence the mechanism of action. To this end, isocyanides (CNR) represent a convenient choice for several reasons: (1) the introduction of multiple isocyanide ligands onto the Fe_2_Cp_2_ core is feasible, as demonstrated in pioneering organometallic studies;^42^ (2) isocyanide ligands coordinated to a diiron­(I) core offer the opportunity to increase the net charge of the complex, thereby improving air stability as well as aqueous solubility and stability under physiological conditions, via conversion to cationic aminocarbyne ligands;^29^ (3) isocyanides display versatile reactivity that could be retained in biological environments, potentially broadening the pharmacological profile of the resulting complexes;^43–45^ (4) there is growing interest in the use of isocyanides in medicinal chemistry;^46^ (5) incorporating a single isocyanide ligand into **B**-type derivatives (Scheme 1c) enhances cytotoxicity.^47^

In this work, we report a straightforward protocol for complete CO/CNR substitution starting from Fp_2_. This reaction has previously been described as nonselective, affording [Fe_2_Cp_2_(CNR)_4_] complexes in admixture with the corresponding [Fe_2_Cp_2_(CO) (CNR)_3_] adducts.^48–50^ The subsequent formation of mono- and bis-aminocarbyne derivatives provides access to robust species unprecedented in bioorganometallic chemistry, with physicochemical properties that make them suitable candidates for evaluation as iron-based anticancer agents.

## 2 Results and Discussion

### 2.1 Synthesis, Structural Characterization and DFT Calculations

Although single or double CO substitution by isocyanides can be accomplished starting from **B**-type complexes (Scheme 1),^47^ complete (triple) CO substitution is not feasible because progressively increasing FeFe → CO back-donation strengthens the coordination of the remaining CO ligands. Therefore, the tetra–isocyanide complexes [Fe_2_Cp_2_(CNR)_4_] (R = Xyl, **1a**; Cy, **1b**; Me, **1c**; Xyl = *meta*-xylyl, Cy = cyclohexyl) were synthesized by reacting [Fe_2_Cp_2_(CO)_4_] with the corresponding isocyanide using an optimized literature procedure (Scheme 2a) relative to that previously reported for **1a** and **1c**. Complex **1b** is unprecedented. Notably, **1a** was isolated in significantly improved yield (90% vs 60% previously reported)^50^ and **1c** was obtained free from the three–CO–substituted side-product.^48^ The reaction with CNXyl proceeded smoothly under stoichiometric conditions (4 equiv. CNXyl vs Fp_2_) in refluxing toluene, yielding a dark-green precipitate of **1a** after 1 h, which was recovered by filtration. In contrast, complete CO substitution with CNCy and CNMe required an excess of isocyanide (two- and 5-fold molar excess, respectively) in refluxing xylene, as well as prolonged reaction times (3 and 24 h, respectively). Products **1b**-**c** were formed together with a black insoluble precipitate and were not isolated, but used directly in the subsequent step. Conversely, **1a** was characterized by IR and ^1^H and ^13^C NMR spectroscopy: in contrast to previous reports,^50^ the NMR spectra are well resolved and indicate the absence of fluxional behavior, in contrast to the well-documented case of the carbonyl homologue Fp_2_.^51^

**Scheme 2:**
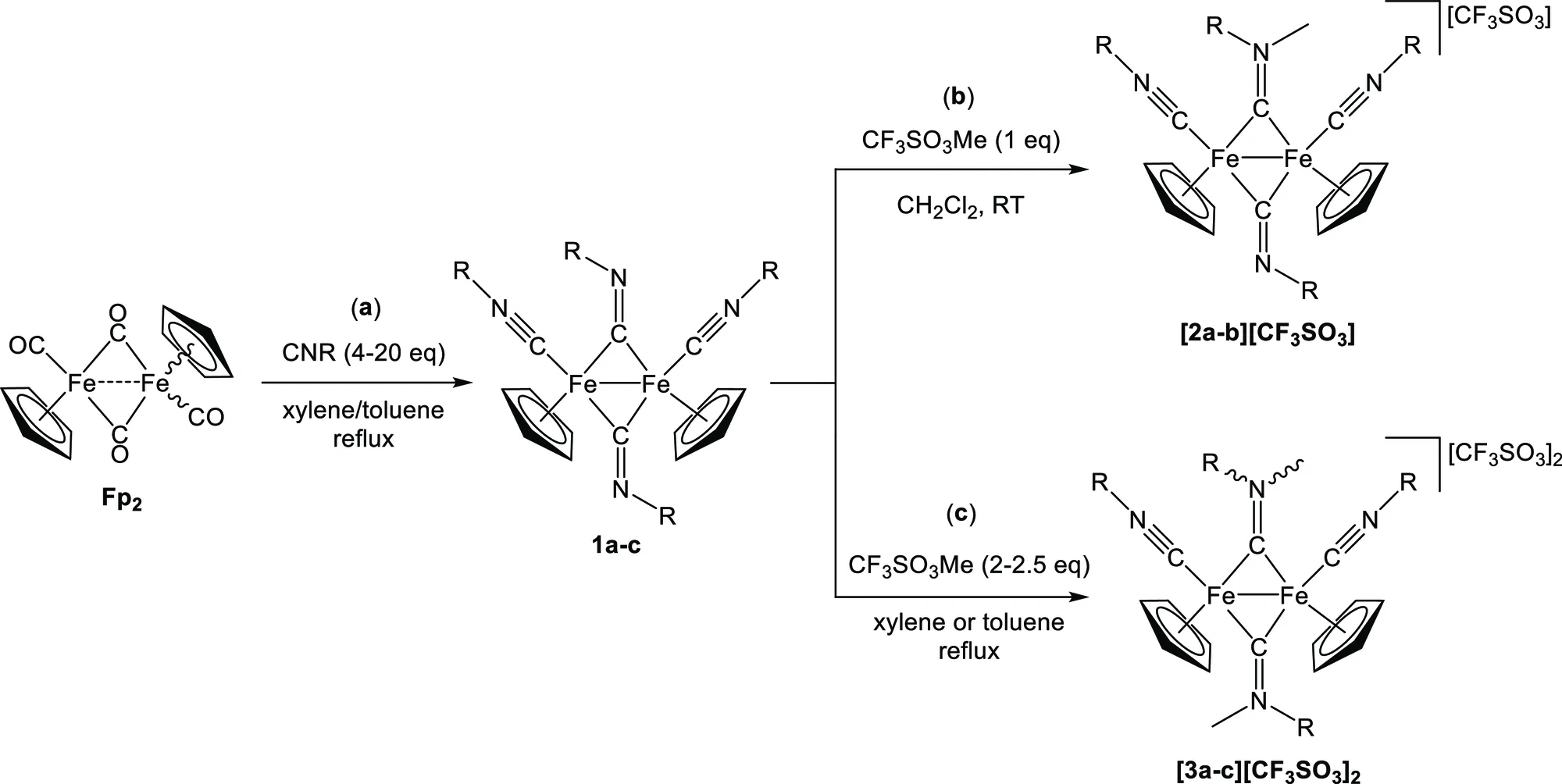
Two-step Synthesis of Carbonyl-free Mono- (**2**) and Bis-Aminocarbyne (**3**) Diiron Aminocarbyne–Isocyanide Complexes Isolated as Triflate Salts^a^

a R = *meta*-xylyl (Xyl) for **1a**, [**2a**]^+^, [**3a**]^2+^; R = cyclohexyl (Cy) for **1b**, [**2b**]^+^, [**3b**]^2+^; R = Me for **1c**, [**3c**]^2+^. Wavy bonds indicate the existence of stereoisomers.

The reactions of **1a**–**b** with methyl triflate (1 equiv) in CH_2_Cl_2_ at room temperature afforded the novel CO-free monoaminocarbyne complexes [Fe_2_Cp_2_(CNR)_3_{μ-CN­(Me) (R)}]^+^ (R = Xyl, **[2a]** ^**+**^; Cy, **[2b]** ^**+**^), see Scheme 2b. Instead, the bis-aminocarbyne derivatives [Fe_2_Cp_2_(CNR)_2_{μ-CN­(Me) (R)}_2_]^2+^ (R = Xyl, **[3a]** ^**2+**^; Cy, **[3b]** ^**2+**^; Me, [**3c**]^2+^) were obtained by treating **1a**–**c** with a slight excess (>2 equiv) of methyl triflate in refluxing toluene or xylene (Scheme 2c). Remarkably, **[3b-c]** ^**2+**^ were prepared in one pot from Fp_2_, bypassing the isolation of the neutral, potentially air-sensitive and difficult-to-handle intermediates **1b**–**c**.

Complexes **[2a-b]­[CF**_**3**_ **SO**_**3**_ **]** and **[3a-c]­[CF**_**3**_ **SO**_**3**_ **]**_**2**_ were purified by alumina chromatography and isolated as air-stable green powders in yields ranging from 30% to 92%. While **[3a-b]** ^**2+**^ are reported here for the first time, **[3c]** ^**2+**^ had previously been accessible only through an eight-step synthesis from [Fe_2_Cp_2_(CO)_4_], with an overall yield below 12%.^52^

Compounds **[2a-b]­[CF**_**3**_ **SO**_**3**_ **]** and **[3a-c]­[CF**_**3**_ **SO**_**3**_ **]**_**2**_ were characterized in the solid state by elemental analysis and IR spectroscopy, and in solution by IR and multinuclear (^1^H, ^13^C) NMR spectroscopy in various solvents (Figures S1–S25).

In principle, stereoisomerism is expected for the organometallic cations **[2]** ^**+**^ and **[3]** ^**2+**^, arising from both the relative cis/trans orientation of the cyclopentadienyl ligands around the Fe_2_Cp_2_ core and the relative syn/anti arrangement of the substituents on the bridging ligands (Schemes S1–S2).^29^

The structures of specific stereoisomers of **[2a]** ^**+**^, **[3a]** ^**2+**^, and **[3b]** ^**2+**^ were determined by single-crystal X-ray diffraction (*S*C-XRD, see below). In parallel, the *cis-anti* (ca) and *cis-syn* (cs) structures of **[2a,b]** ^**+**^ and **[3a,b]** ^**2+**^ were optimized in the gas phase at the B3LYP level, showing very good agreement with the corresponding crystallographic data (Table S2), particularly regarding the Fe_2_(μ-C)_2_ core (Figure S28).

The ^1^H and ^13^C NMR spectra of **[2a-b]** ^**+**^ display a single set of resonances, indicating the predominance of one diastereoisomer in solution. ^1^H NOESY experiment performed on **[2a]** ^**+**^ by selective irradiation of the Cp resonance at 5.18 ppm (Figure S14) revealed NOE cross-peaks with the other Cp signal (4.42 ppm) and with the signals of methyl groups belonging to the aminocarbyne (4.61 ppm) and xylyl (2.83, 2.33 ppm) moieties, supporting the assignment of a *cis*-*anti* configuration. In contrast, the Cp resonances of **[2b]** ^**+**^ are closely spaced in chemical shift (δ = 4.94, 4.90 ppm) and exhibit noticeable line broadening (Figure S15). This behavior is consistent with rapid *anti*–*syn* interconversion on the NMR time scale. DFT calculations confirm that the *syn* and *anti* forms are nearly isoenergetic (<1 kcal/mol). Direct rotation around the μ-C–N bond is, however, associated with a prohibitive energy barrier, rendering such a pathway unlikely. The observed fluxional behavior is therefore more plausibly attributed to transient exchange between bridging and terminal coordination modes of one isocyanide ligand, a process well documented in neutral Fe_2_Cp_2_-based isocyanide complexes.^42^

The bridging aminocarbyne ligands of **[2a-b]** ^**+**^ give rise to characteristic downfield ^13^C NMR resonances at 330–340 ppm,^29,53,54^ whereas the bridging and terminal isocyanide ligands are observed at ca. 230 ppm and 162–175 ppm, respectively. These assignments are consistent with the infrared absorptions in the 1530–1500 (aminocarbyne C–N), 1780–1720 (isocyanide μ-C=N) and 2130–2100 (isocyanide t-C≡N) cm^–1^ regions. Comparison with the corresponding tricarbonyl complexes [Fe_2_Cp_2_(CO)_3_{μ-CN­(Me) (R)}]^+^ (R = Xyl, Cy; **B**-type complexes, see Scheme 1)^29,32^ reveals a marked decrease in the carbyne–N stretching wavenumber (−30/36 cm^–1^), accompanied by an upfield shift of the corresponding ^13^C aminocarbyne resonance (+10.7/15.2 ppm) (Table S1). This trend reflects increased FeFe → aminocarbyne π-back-donation^29^ in **[2a-b]** ^**+**^ relative to **B**, as expected upon CO/CNR substitution. Conversely, the Cp and *N*–Me resonances in **[2a-b]** ^**+**^ are shifted upfield compared to their tricarbonyl analogues **B**.

Consistent with the NMR data, the *S*C-XRD structure of **[2a]­[CF**_**3**_ **SO**_**3**_ **]** confirms a *cis*-*anti* stereochemical arrangement (Figure 1). This finding contrasts with the *trans-anti* geometry previously observed in the solid state for the tetra–isocyanide complexes [Fe_2_Cp_2_(CNXyl)_2_(μ-CNXyl)_2_] (**1a**)^55^ and [Fe_2_Cp_2_(CNPh)_2_(μ-CNPh)_2_].^56^ A *cis* disposition of the Cp rings has, however, been recognized in [Fe_2_Cp_2_(CO)_2_(μ-CNMe) (μ-CNMe_2_)]^+^ ^57^ and [Fe_2_Cp_2_(CO) (CNMe) (μ-CO) (μ-CNMe_2_)]^+^, which share structural similarities with **[2a]** ^**+**^.

**Figure 1:**
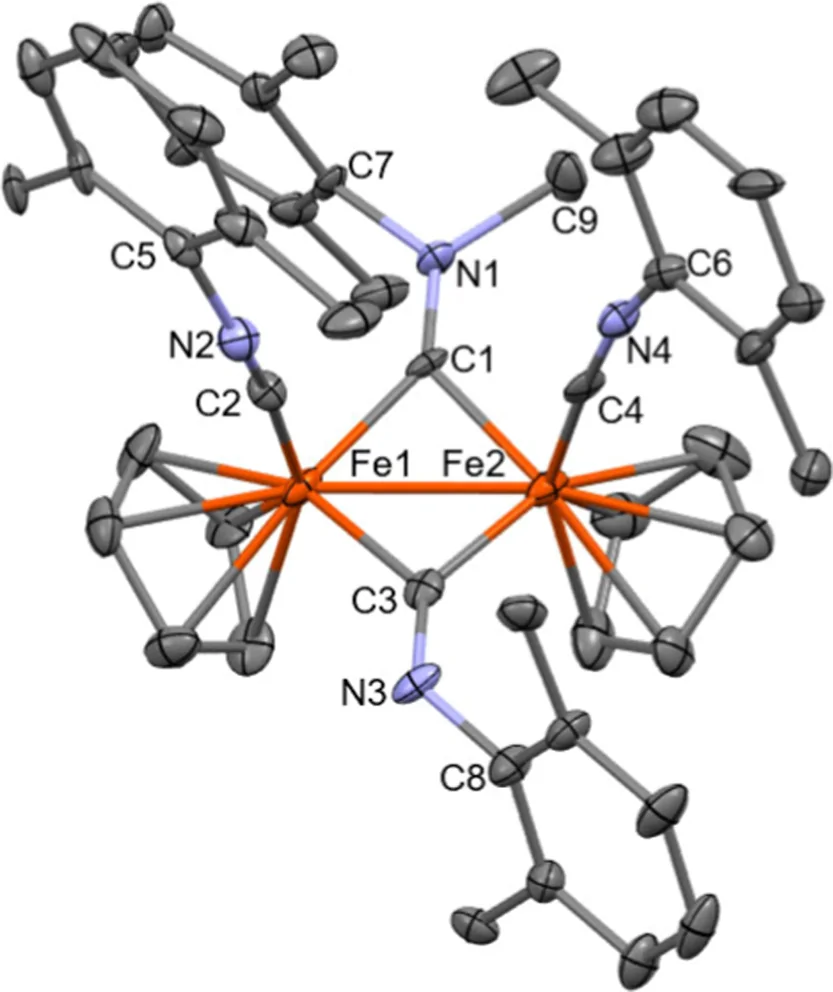
Single-crystal X-ray structure of **[2a]** ^**+**^. This compound crystallizes in space group *Pca*2_1_. Displacement ellipsoids are drawn at the 30% probability level. Hydrogen atoms are omitted for clarity. Selected bond lengths (Å) and angles (°): Fe1–Fe2 2.5134(18), Fe1–C1 1.920(16), Fe2–C1 1.889(10), Fe1–C3 1.903(10), Fe2–C3 1.913(16), Fe1–C2 1.831(11), Fe2–C4 1.788(11), Fe1-Cp_av_ 2.11(2), Fe2-Cp_av_ 2.13(2), C1–N1 1.244(16), N1–C9 1.588(15), N1–C7 1.452(9), C3–N3 1.302(17), N3–C8 1.376(10), C2–N2 1.140(14), N2–C5 1.413(11), C4–N4 1.193(14), N4–C6 1.356(10), Fe1–C1–Fe2 82.6(5), Fe1–C3–Fe2 82.4(5), Fe1–C2–N2 174.0(11), Fe2–C4–N4 175.4(12), C1–N1–C9 124.8(9), C1–N1–C7 126.8(10), C7–N1–C9 108.0(8), C3–N3–C8 131.6(11), C2–N2–C5 160.8(11), C4–N4–C6 165.3(10).

The short C1–N1 bond distance [1.244(16) Å], falling within the range expected for diiron aminocarbyne systems (Figure S31), is consistent with significant π-character. N1 adopts an essentially ideal trigonal-planar geometry [sum angles 359.6(16)°]. In conjunction with the ^13^C NMR data, these structural features support assignment of the bridging CN­(Me) (Xyl) ligand as an aminocarbyne-iminium hybrid.

The short C2–N2 [1.140(14) Å] and C4–N4 [1.193(14) Å] bond lengths are in line with those reported for other Fe^I^–CNXyl complexes.^42,58,59^ Accordingly, the Fe1–C2–N2 [174.0(11)°], Fe2–C4–N4 [175.4(12)°], C2–N2–C5 [160.8(11)°] and C4–N4–C6 [165.3(10)°] angles support a quasi-linear coordination geometry at nitrogen. In contrast, the C3–N3 distance [1.302(17) Å] and the C3–N3–C8 angle [131.6(11)°] agree with a C = N double bond and sp^2^ hybridization of N3.

The ^1^H and ^13^C NMR spectra of **[3a-b]** ^**2+**^ display two sets of signals. The major isomer exhibits equivalent Cp and isocyanide ligands, consistent with an *anti*-arrangement of the aminocarbyne *N*-substituents (Figure S19); conversely, the minor isomer shows distinct resonances for each terminal ligand, indicative of a *syn* configuration, the *cis* disposition of the Cp ligands being supported by ^1^H NOESY data (Figure S20). The *cis*-*anti* isomer strongly predominates over the *cis*-*syn* isomer in the cyclohexyl derivative **[3b]** ^**2+**^ (9:1 ratio), while a less pronounced preference is observed for the xylyl analogue **[3a]** ^**2+**^ (1.5:1 ratio). In contrast, **[3c]** ^**2+**^ exists as a single species in solution.^52^

Compounds **[3a-c]** ^**2+**^ exhibit strong IR absorptions in the 2130–2200 and 1530–1600 cm^–1^ regions, and ^13^C NMR signals in the 143–165 and 318–337 ppm ranges, consistent with terminal isocyanide and bridging aminocarbyne ligands, respectively.

For **[3a]** ^**2+**^, the *cis*-*anti* configuration was confirmed in the solid state by *S*C-XRD; its bis-triflate salt crystallized in the centrosymmetric space group *C*2/*c* as a racemic (1:1) mixture of (RR) and (SS) enantiomers. In contrast, **[3b]­[CF**_**3**_ **SO**_**3**_ **]**_**2**_ crystallized as both *cis-anti* and *cis-syn* stereoisomers. The *cis*-anti form was obtained as individual enantiomers from independent crystallization batches; the (RR) and (SS) enantiomers crystallized in distinct Sohncke space groups (P2 and *P*2_1_2_1_2, respectively) (Figures S26–S27 and S32–S34).^60,61^

Across all crystallographically characterized cations **[3a-b]** ^**2+**^ (Figures 2, 3, 4, and 5), the C1–N1 bond lengths (1.27–1.30 Å) are comparable to those observed for **[2a]** ^**+**^, in accordance with the description of the bridging ligands as aminocarbyne-iminium hybrids. To the best of our knowledge, only [Fe_2_(η^5^-C_5_H_4_Me)_2_(CO)_2_(μ-CNMe_2_)_2_]^2+62^ and [Fe_2_(CO)_6_(μ-CNEt_2_)_2_][Bibr ref63] had been structurally characterized prior to this work among diiron complexes bearing two bridging aminocarbyne ligands.

**Figure 2:**
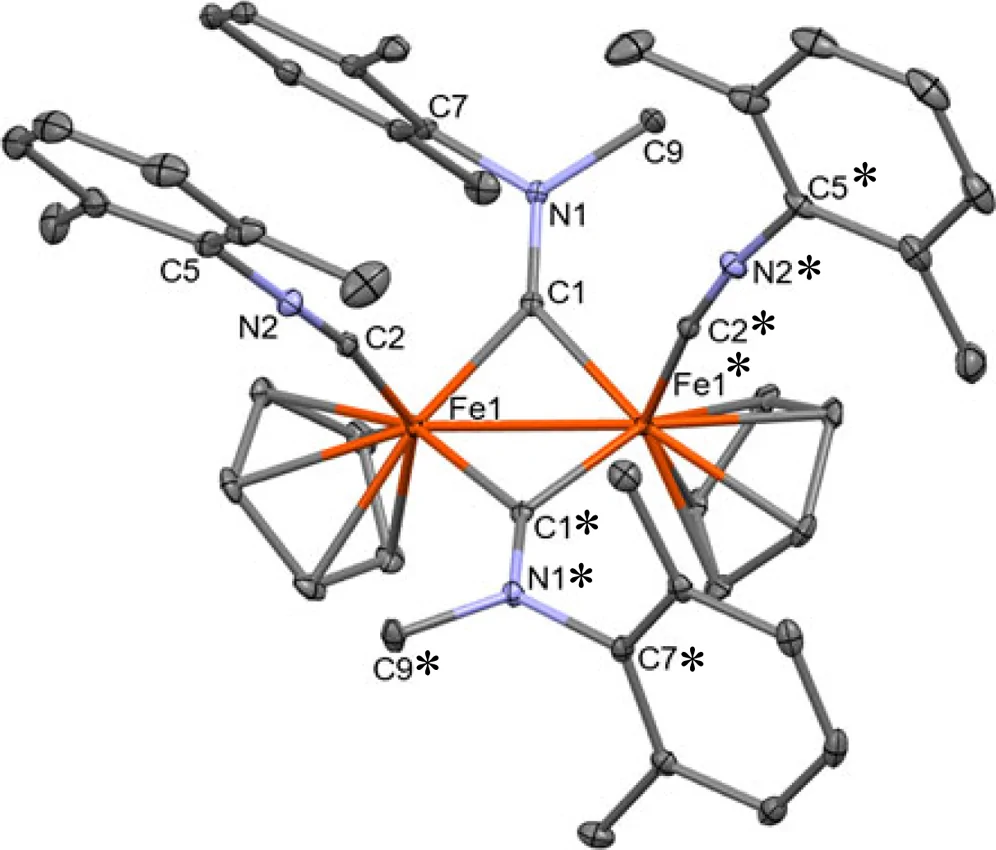
Single-crystal X-ray structure of *cis-anti*-**[3a]** ^**2+**^. The compound crystallizes as a 1:1 (RR) and (SS) racemic mixture in the centrosymmetric space group *C*2/*c*. Displacement ellipsoids are drawn at the 30% probability level. Hydrogen atoms are omitted for clarity. Selected bond lengths (Å) and angles (°): Fe1–Fe1* 2.4768(4), Fe1–C1 1.8771(14), Fe1*-C1 1.8800(14), Fe1–C2 1.8486(15), Fe1-Cp_av_ 2.120(3), C1–N1 1.2959(18), N1–C9 1.4810(18), N1–C7 1.4617(17), C2–N2 1.161(2), N2–C5 1.4060(19), Fe1–C1–Fe1* 82.49(6), Fe1–C2–N2 170.04(13), C1–N1–C9 123.54(12), C1–N1–C7 121.73(12), C7–N1–C9 114.70(11), C2–N2–C5 171.29(15). Atoms labeled with * are generated by a 2-fold rotation axis.

**Figure 3:**
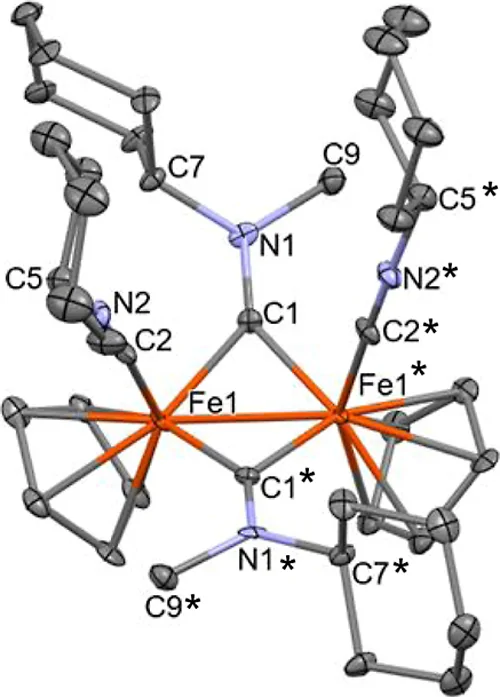
Single-crystal X-ray structure of *cis-anti*-(RR)-**[3b]** ^**2+**^. This enantiomer crystallizes in the chiral space group *P*2. Displacement ellipsoids are drawn at the 30% probability level. Hydrogen atoms are omitted for clarity. Selected bond lengths (Å) and angles (°): Fe1–Fe1* 2.489(5), Fe1–C1 1.849(18), Fe1*-C1 1.906(18), Fe1–C2 1.838(18), Fe1-Cp_av_ 2.12(4), C1–N1 1.27(2), N1–C9 1.50(2), N1–C7 1.50(2), C2–N2 1.16(2), N2–C5 1.45(3), Fe1–C1–Fe1* 83.0(7), Fe1–C2–N2 175.6(17), C1–N1–C9 120.0(16), C1–N1–C7 124.9(16), C7–N1–C9 115.0(15), C2–N2–C5 172(2). Atoms labeled with * are generated by a 2-fold rotation axis.

**Figure 4:**
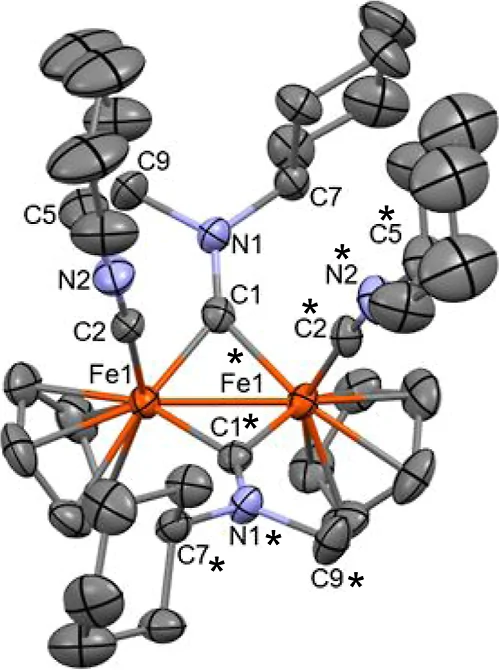
Single-crystal X-ray structure of *cis-anti*-(SS)-**[3b]** ^**2+**^. This enantiomer crystallizes in the chiral space group *P*2_1_2_1_2. Displacement ellipsoids are drawn at the 30% probability level. Hydrogen atoms are omitted for clarity. Selected bond lengths (Å) and angles (°): Fe1–Fe1* 2.4696(15), Fe1–C1 1.883(6), Fe1*-C1 1.880(6), Fe1–C2 1.836(6), Fe1-Cp_av_ 2.105(8), C1–N1 1.282(8), N1–C9 1.491(9), N1–C7 1.481(8), C2–N2 1.144(9), N2–C5 1.438(10), Fe1–C1–Fe1* 82.0(2), Fe1–C2–N2 175.8(7), C1–N1–C9 121.1(6), C1–N1–C7 122.6(5), C7–N1–C9 116.3(5), C2–N2–C5 171.4(9). Atoms labeled with * are generated by a 2-fold rotation axis.

**Figure 5:**
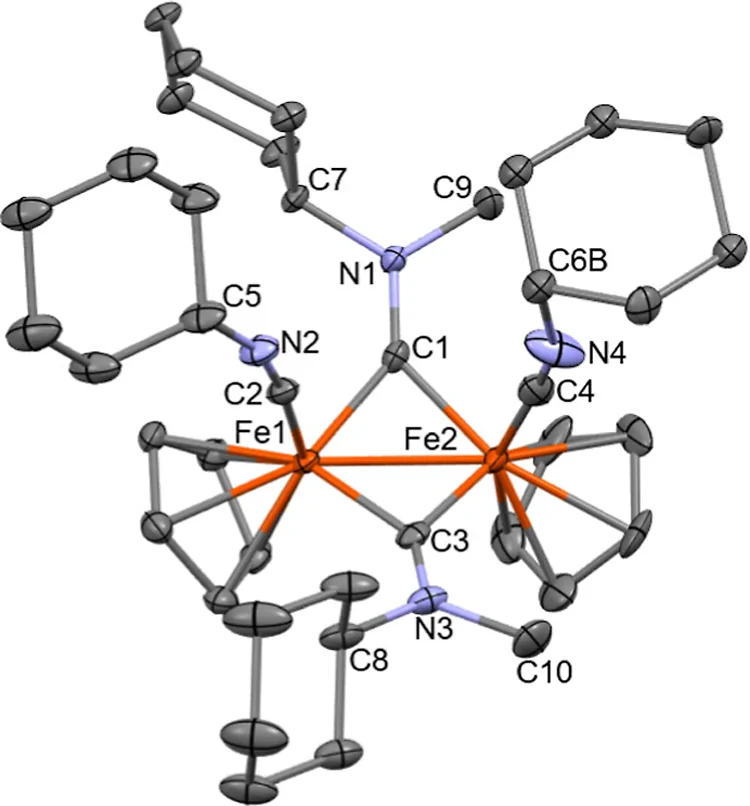
Single-crystal X-ray structure of *cis-syn-* **[3b]** ^**2+**^. The compound crystallizes in the centrosymmetric space group *Pbcn*. Displacement ellipsoids are drawn at the 30% probability level. Hydrogen atoms are omitted for clarity. Selected bond lengths (Å) and angles (°): Fe1–Fe2 2.4762(11), Fe1–C1 1.876(6), Fe2–C1 1.863(5), Fe1–C3 1.876(6), Fe2–C3 1.865(6), Fe1–C2 1.829(6), Fe2–C4 1.829(7), Fe1-Cp_av_ 2.119(13), Fe2-Cp_av_ 2.103(13), C1–N1 1.300(7), C3–N3 1.298(7), N2–C2 1.155(8), N4–C4 1.153(9), C9–N1 1.468(7), C7–N1 1.490(6), C10–N3 1.477(8), C8–N3 1.488(8), C6B–N4 1.638(15), C5–N2 1.464(8), Fe1–C1–Fe2 83.0(2), Fe1–C3–Fe2 82.9(2), C4–N4–C6B 161.0(9), C2–N2–C5 163.6(6), C1–N1–C9 121.4(5), C1–N1–C7 122.4(5), C7–N1–C9 116.1(4), C3–N3–C10 120.8(6), C3–N3–C8 122.7(5), C8–N3–C10 116.4(5).

A natural bond orbital analysis was performed on **[2a-b]** ^**+**^ and **[3a-b]** ^**2+**^ to extract Wiberg bond orders, which are summarized in Table S3. In **[2a-b]** ^**+**^, the C1–N1 Wiberg bond order ranges from 1.41 to 1.44, consistent with its description as an aminocarbyne (formal bond order = 1)/iminium (formal bond order = 2) hybrid. The Wiberg bond order is appreciably higher for R = Cy than for R = Xyl (1.442 vs 1.405 in the corresponding *cis*-anti forms), indicating that the iminium contribution, associated with positive charge localization at nitrogen, is better stabilized by the more electron-donating *N*-substituent (Cy). The bridging isocyanide ligand exhibits an almost ideal double-bond character (C3–N3 bond order ranging from 2.049 to 2.080). In contrast, the terminal isocyanide bond indexes (C2–N2 and C4–N4) are significantly lower than the formal triple bond value (2.257–2.344), in agreement with π back-donation from the iron centers. In **[3a-b]** ^**2+**^, the Wiberg bond orders of the bridging aminocarbyne ligands increase relative to the monoaminocarbyne systems **[2a-b]** ^**+**^ (e.g., 1.464 vs 1.405 for the *cis*-anti forms of **[3a]** ^**2+**^ and **[2a]** ^**+**^). This trend is accompanied by elongation of the Fe-carbyne bonds (Wiberg b.o. of 0.834 vs 0.875 for *cis*-anti-**[3a]** ^**2+**^ and *cis*-anti-**[2a]** ^**+**^) and reflects a reduced degree of metal-to-ligand back-donation in the dicationic species. Accordingly, the Wiberg bond orders of the terminal isocyanide ligands increase slightly (ca. + 0.07) in **[3a-b]** ^**2+**^ compared to **[2a-b]** ^**+**^.

The cations [**2a,b**]^+^ and [**3a,b**]^2+^ were also compared with their carbonyl analogues, where the CNR ligands are replaced by CO, namely [**2a,b** ^**CO**^]^+^ and [**3a,b** ^**CO**^]^2+^ (Tables S4–S5). The former correspond to the well-established family of compounds **B** in Scheme 1, whereas [**3a,b** ^**CO**^]^2+^ have been reported only sporadically in literature.^62^ Owing to the stronger π-acceptor character of carbonyl ligands, FeFe → μ-CN back-donation is reduced, leading to strengthening of the C1–N1 bond. In the dicationic system [**3a,b** ^**CO**^]^2+^, this electronic effect combines with the increased positive charge to enhance the iminium contribution, which reaches a maximum in the *N*-cyclohexyl derivative [**3b** ^**CO**^]^2+^ (Wiberg bond order = 1.557).

### 2.2 Electrochemistry

The electrochemical behavior of **[2a]­[CF**_**3**_ **SO**_**3**_ **]** and **[3a]­[CF**_**3**_ **SO**_**3**_ **]**_**2**_ was investigated by cyclic voltammetry (CV) and IR-spectroelectrochemistry (IR-SEC) in CH_3_CN solutions containing 0.1 mol dm^–3^ [N^*n*^Bu_4_]­PF_6_ as supporting electrolyte. In parallel, a computational study was performed to optimize the geometries of the electrogenerated species using the PCM solvation model for acetonitrile (ε = 35.688, see Tables S6–S7).^64^ The corresponding IR spectra (Figures S29 and S30), calculated within the double-harmonic approximation, are consistent with the optimized structures of the electrogenerated species.

Both complexes display three redox processes: **[2a]** ^**+**^ undergoes two successive oxidations and one reduction, whereas **[3a]** ^**2+**^ exhibits a single oxidation and two reductions (Figures S35 and S36). All redox events are diffusion-controlled and involve one-electron steps that are electrochemically and chemically reversible on the CV time scale, except for the second oxidation of [**2a]** ^**+**^, which is followed by fast chemical reactions, and the oxidation of **[3a]** ^**2+**^, which is electrochemically quasi-reversible. The formal electrode potentials of the observed redox processes are summarized in Table 1 and compared with those of the recently reported tricarbonyl analogue [Fe_2_Cp_2_(CO)_2_(μ-CO)­{μ-CN­(Me) (Ph)}]^+^, **[2d** ^**CO**^ **]** ^**+**^.^65^ Complexes **[2a]** ^**+**^ and **[3a]** ^**2+**^ are reduced to their neutral counterparts at similar potentials (about −1.4 V), while the oxidation to the tricationic species occurs at approximately 300 mV higher potential for **[3a]** ^**2+**^.

**Table 1:** Formal Electrode Potentials (V, *vs* Ag/AgCl and, in Brackets, *vs* FeCp_2_) and Peak-To-Peak Separations (mV, Measured at 0.1 V/s) for the Redox Processes of **[2a]** ^**+**^ and **[3a]** ^**2+**^ in CH_3_CN/[N^*n*^Bu_4_]­PF_6_, and of **[2d** ^**CO**^ **]** ^**+**^ in CH_2_Cl_2_/[N^*n*^Bu_4_]­PF_6_

	reduction processes				oxidation processes			
	*E*°_1_	Δ*E*_p_	*E*°_2_	Δ*E*_p_	*E*°_3_	Δ*E*_p_	*E*°_4_	Δ*E*_p_
*Net charge variation*	+1→0		+2→+1		+1→+2		+2→+3	
[**2a**]^+^	–1.38 [−1.80]	75			+0.50 [+0.08]	76	+1.33 ^[^ ^a^ ^]^ [+0.91]	
[**3a**]^2+^	–1.45 [−1.87]	68	–0.66 [−1.08]	67			+1.62 [+1.20]	110
**[2d** ^**CO**^ **]** ^**+**^	–0.96 [−1.41]	88					+1.42 ^[^ ^b^ ^]^ [+0.97]	101

a Peak potential for irreversible processes.

b Two-electron process.

Assuming that the redox potentials of the investigated complexes in aqueous solution do not differ significantly from those measured in CH_3_CN, only the first oxidation of **[2a]** ^**+**^ and the first reduction of **[3a]** ^**2+**^ might be accessible under physiological conditions. Indeed, the biologically relevant potential window is generally considered to span −0.4 to +0.8 V vs SHE (corresponding to −0.6 to +0.6 V vs Ag/AgCl).^66,67^

Comparison with the carbonyl analogue **[2d** ^**CO**^ **]** ^**+**^ indicates that replacement of π-acceptor CO ligands with prevalently σ-donor isocyanides renders the reduction of **[2a]** ^**+**^ more difficult by ≈400 mV. The oxidation behavior also differs markedly: while **[2a]** ^**+**^ exhibits two consecutive one-electron oxidation steps (see above), **[2d** ^**CO**^ **]** ^**+**^ undergoes a single reversible two-electron oxidation. Redox processes found to be reversible on the CV time scale were further investigated by *in situ* IR-SEC using an optically transparent thin-layer electrochemical (OTTLE) cell (Table 2).^68^

**Table 2:** Infrared Stretching Frequencies (cm^–1^) of Terminal (ν^t^_CN_) and Bridging (ν^b^_CN_) Isocyanide Ligands in **[2a]** ^**+**^, **[3a]** ^**2+**^, and Their Reduction/Oxidation Derivatives in CH_3_CN Solution

complex	ν^t^_CN_			ν^b^_CN_
**2a**	2036s	2002s	1980sh	1650m,br
[**2a**]^+^	2099vs	2075sh		1760m,br
[**2a**]^2+^	2135vs			
**3a**				1690m,br
[**3a**]^+^	2070vs	2050sh		
[**3a**]^2+^	2129vs	2118sh		
[**3a**]^3+^	2165w			

Upon reduction of **[2a]** ^**+**^ (−1.20 to −1.40 V vs Ag pseudoreference electrode), both the terminal and bridging isocyanide bands undergo a pronounced redshift (from 2099vs, 2075sh, 1760br cm^–1^ to 2036s, 2002s, 1980sh, 1650br cm^–1^; Figure S37, top), in accordance with the formation of the radical **2a**. This species remains stable on the spectroelectrochemical time scale, as evidenced by well-defined isosbestic points throughout the IR spectral sequence and full recovery of the initial spectrum upon back-oxidation (Figure S37, bottom). In agreement with the experimental IR data, the DFT-optimized structure of **2a** shows elongation of the terminal isocyanide C–N bonds (from 1.169 and 1.171 Å in **[2a]** ^**+**^ to 1.182 and 1.183 Å in **2a**) and shortening of the corresponding Fe–C bonds (from 1.821 and 1.816 Å in **[2a]** ^**+**^ to 1.784 and 1.782 Å), accompanied by slight bending of the CNC angles (from 170° and 167° to 163° and 160°). The Wiberg bond orders of the terminal isocyanide C–N bonds decrease from 2.257 and 2.269 in **[2a]** ^**+**^ to 2.114 and 2.159 in **2a**. These changes are in line with increased FeFe → isocyanide back-donation in the reduced species.

The IR spectra recorded for a solution of **[2a]** ^**+**^ upon increasing the applied potential at the working electrode (WE) from +0.50 to +0.75 V (vs Ag pseudoreference electrode) are consistent with the formation of the one-electron-oxidized dication **[2a]** ^**2+**^ (Figure S38, top). A largely reversible shift of the terminal isocyanide band to higher wavenumber (2135 cm^–1^) is observed (Figure S38, bottom), accompanied by disappearance of the bridging μ-CN absorption. The DFT-optimized structure of **[2a]** ^**2+**^ supports these findings, revealing substantial asymmetry of the bridging ligands, with the isocyanide acting as a semibridging ligand (Fe1–C3 and Fe2–C3 bond lengths measure 1.853 and 2.446 Å, respectively, see Figure 6).

**Figure 6:**
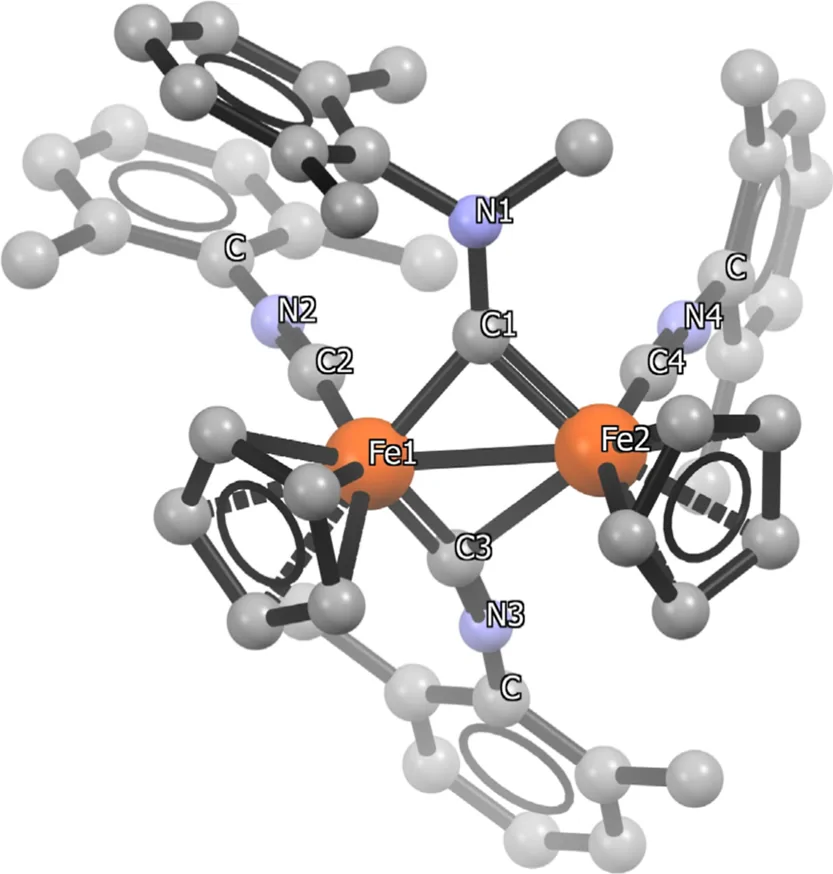
DFT-optimized structure of **[2a]** ^**2+**^ (PCM solvation model for acetonitrile). Selected bond lengths (Å) and angles (°): Fe1–C2 1.838, C2–N2 1.161, Fe1–C1 1.934, Fe2–C1 1.839, Fe2–C4 1.865, C4–N4 1.160, Fe1–C3 1.853, Fe2–C3 2.446, C3–N3 1.168, Fe1–C2–N2 174.83, C2–N2–C 176.39, Fe1–C3–N3 165.44, Fe1–C3–Fe2 71.93, C3–N3–C 168.89, Fe1–C1–Fe2 85.84, Fe2–C4–N4 176.56, C4–N4–C 177.25.

IR-SEC experiments on **[3a]** ^**2+**^ revealed remarkable stability of the accessible charge states (+3, +1, and 0), even over extended time scales (Figure S39, bottom, and Figure S40, bottom).

Two consecutive reductions were observed within the potential range −0.55 to −1.50 V. The first reduction (−0.55 to −0.80 V) is accompanied by a shift of the terminal isocyanide stretching bands to lower wavenumbers (from 2129s and 2118sh cm^–1^ to 2070s and 2050sh cm^–1^; Figure S39, top), consistent with increased FeFe → CNR π back-donation. The second reduction (−0.95 to −1.50 V) leads to disappearance of the terminal isocyanide bands and the appearance of a broad absorption at 1690 cm^–1^, indicative of bridging isocyanide coordination (Figure S39, middle). A DFT optimization of the doubly reduced species starting from the geometry of **[3a]** ^**2+**^ retained terminal isocyanide ligands and did not reproduce the experimental IR data. Alternative structures were therefore explored, including those featuring terminal aminocarbyne ligands; however, no stable minima consistent with the experimental IR data were identified. We therefore propose that double reduction promotes a more extensive reorganization of the coordination framework, possibly involving coupling and/or redistribution processes between isocyanide and aminocarbyne ligands.^69–72^

Upon gradually increasing the WE potential from +1.55 to +1.95 V in a solution of **[3a]** ^**2+**^, the terminal isocyanide band at 2129 cm^–1^ progressively decreases in intensity, while a weak new absorption emerges at higher wavenumber (2165 cm^–1^; Figure S40, top). The low intensity of this band is unlikely to originate from precipitation of the +3 oxidized species, as no solid formation was observed by visual inspection of the OTTLE cell. Rather, the quasi-reversible character of the corresponding electron transfer (ΔE_p_ = 110 mV in CV) suggests that oxidation is accompanied by structural rearrangement. This reorganization may attenuate the change in dipole moment associated with the CN vibration, thereby lowering its IR intensity. Consistently, the calculated structure of **[3a]** ^**3+**^ (Figure 7) indicates loss of ideal *C*_2_ symmetry arising from inequivalent terminal isocyanide ligands; the calculated splitting of the corresponding stretching modes (39 cm^–1^) is also in line with the experimental observations.

**Figure 7:**
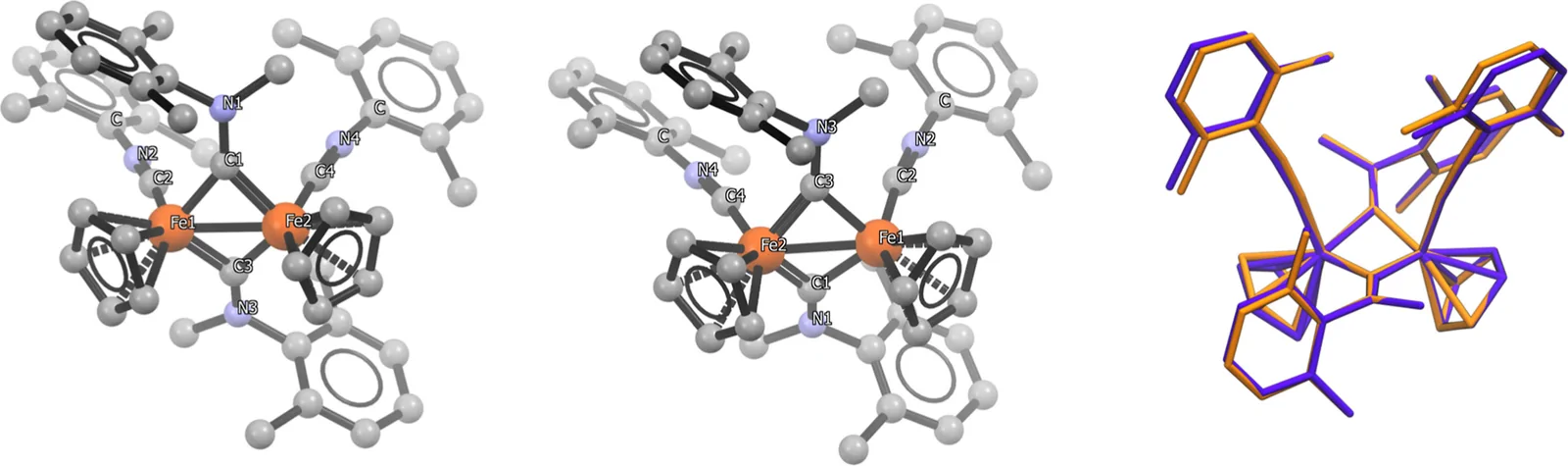
Comparative view of DFT-optimized structures of **[3a]** ^**2+**^ (in vacuo, left) and **[3a]** ^**3+**^ (PCM solvation model for acetonitrile, center). Selected bond lengths (Å) and angles (°) for **[3a]** ^**2+**^, with the corresponding values for **[3a]** ^**3+**^ given in parentheses: Fe1–C2 1.843 (1.907), C2–N2 1.163 (1.158), Fe1–C1 1.873 (1.979), Fe2–C1 1.871 (1.826), Fe2–C4 1.843 (1.856), C4–N4 1.163 (1.158), Fe1–C3 1.871 (1.967), Fe2–C3 1.873 (1.845), C3–N3 1.292 (1.282), Fe1–C2–N2 172.91 (172.66), C2–N2–C 170.53 (174.65), Fe1–C3–N3 137.52 (133.67), Fe1–C3–Fe2 82.30 (82.83), Fe1–C1–Fe2 82.30 (82.97), Fe2–C4–N4 172.90 (171.59), C4–N4–C 170.54 (171.76).

### 2.3 Behavior in Aqueous Solutions

The water solubility, octanol–water partition (Log *P*_ow_), and stability of the new complexes in physiological-like media were examined using established ^1^H NMR and UV–vis methods (Table 3 and S8).^73^ Water solubility reflects the distinct net charges of the complexes and the nature of the *N*-substituents: it is high (≈10^–2^ M) for the dicationic complex **[3c]­[CF**_**3**_ **SO**_**3**_ **]**_**2**_, appreciable (∼10^–4^ M) for **[3a-b]­[CF**_**3**_ **SO**_**3**_ **]**_**2**_ and significantly lower (<10^–4^ M) for the monocationic complexes **[2a-b]­[CF**_**3**_ **SO**_**3**_ **]**. Accordingly, Log *P*_*ow*_ values correlate with prevalent hydrophilicity for **[3c]­[CF**_**3**_ **SO**_**3**_ **]**_**2**_ and lipophilicity for **[2a]­[CF**_**3**_ **SO**_**3**_ **]**, while the remaining compounds display substantial amphiphilicity (−0.8 < Log *P*_*ow*_ < −0.1). The marked difference shown by **[2a]­[CF**_**3**_ **SO**_**3**_ **]** and **[2b]­[CF**_**3**_ **SO**_**3**_ **]** is consistent with the more hydrophobic character of the xylyl groups in the former (Log *P*_*ow*_ > 1.5) relative to the cyclohexyl group in the latter (Log *P*_*ow*_ = −0.27).

**Table 3:** Solubility in D_2_O Determined by ^1^H NMR Spectroscopy (Me_2_SO_2_ as the Internal Standard); Octanol/Water Partition Coefficients (Log *P*_ow_); Percentage of Residual Complex After 72 h at 37 °C in Aqueous Media, Determined by ^1^H NMR Spectroscopy (Ca. 10^–3^ M D_2_O/DMSO-*d*_6_ Solutions, Me_2_SO_2_ as the Internal Standard; Ca. 10^–3^ M DMEM-D/DMSO-*d*_6_ Solutions, TMSP-d_4_ as the Internal Standard) or UV–Vis Spectroscopy (Ca. 10^–4^ M Aqueous Solutions)^a^

complex	solubility/mol·L^–1^	Log *P*_ow_		% residual complex (72 h, 37 °C)			
			H_2_O (UV–vis)	%*DMSO (v/v)*	D_2_O/DMSO-*d*_6_ (^1^H NMR)	DMEM-d/DMSO-*d*_6_ (^1^H NMR)	%DMSO-*d*_6_ *(v/v)*
**[2a][CF**_**3**_ **SO**_**3**_ **]**	<10^–4^	>1.5	88	40			
**[2b][CF**_**3**_ **SO**_**3**_ **]**	<10^–4^	–0.27 ± 0.04	84	40			
**[3a][CF**_**3**_ **SO**_**3**_ **]**_**2**_	1.3 × 10^–4^	–0.06 ± 0.04	93	0	90^b^	86^b^	40
**[3b][CF**_**3**_ **SO**_**3**_ **]**_**2**_	5.5 × 10^–4^	–0.80 ± 0.01	90	0	96	89	40
**[3c][CF**_**3**_ **SO**_**3**_ **]**_**2**_	2.6 × 10^–2^ ^b^	<−1.5	98	0	84	89	0

a Data refer collectively to all isomers present in solution. TMSP = 3-(trimethylsilyl)­propionic-2,2,3,3-d_4_ acid sodium salt.

b TMSP-*d*_4_ was used as the internal standard.

The stability of the metal complexes was monitored over 72 h by UV–vis and ^1^H NMR experiments in aqueous solution and DMEM cell culture medium at 37 °C. Stability was further checked by UV–Vis measurements in a physiological buffer for **[2a]­[CF**_**3**_ **SO**_**3**_ **]** and **[3a]­[CF**_**3**_ **SO**_**3**_ **]**_**2**_ (see Section 6). Dimethyl sulfoxide was used as a cosolvent to achieve suitable concentration levels when necessary. NMR stability was evaluated only for **[3a-c]­[CF**_**3**_ **SO**_**3**_ **]**_**2**_ (Table S8 and Figures S41–S54), as DMSO contents exceeding 70% would be required for **[2a-b]­[CF**_**3**_ **SO**_**3**_ **]** to reach appreciable concentration for this technique.

Taken together, the obtained NMR and UV–vis results are consistent and indicate excellent stability of both series of complexes in physiological-like media, supporting their suitability for biological studies. Interestingly, the *anti*/*syn* ratio of **[3a,b]­[CF**_**3**_ **SO**_**3**_ **]**_**2**_ decreased over 72 h, indicating that structural rearrangements are operative in aqueous solution, leading to isomer interconversion.

### 2.4 Cellular Studies

#### 2.4.1 Antiproliferative Activity

The antiproliferative activity of the new diiron complexes was evaluated against the A2780 ovarian, A549 lung, and U87 glioblastoma cancer cell lines, as well as the normal fibroblast cell line MRC-5 (Table 4 and Figures S55–S58). The diiron tricarbonyl aminocarbyne complex [Fe_2_Cp_2_(CO)_2_(μ-CO) (μ-CNMe_2_)]­[CF_3_SO_3_], **B–Me**,^32^ and the reference drug cisplatin were included in the analyses as negative and positive controls, respectively.

**Table 4:** IC_50_ Values (μM) Determined After 48 h of Incubation of Cancer (A2780, U87, A549) and Non-cancer (MRC-5) Cell Lines With the Complexes^a^

complex	A2780	A549	U87	MRC-5
**[2a][CF**_**3**_ **SO**_**3**_ **]**	0.58 ± 0.02	2.5 ± 0.4	0.43 ± 0.02	1.5 ± 0.2
**[2b][CF**_**3**_ **SO**_**3**_ **]**	0.78 ± 0.09	12 ± 2	2.8 ± 0.2	5.14 ± 0.03
**[3a][CF**_**3**_ **SO**_**3**_ **]**_**2**_	2.30 ± 0.09	28 ± 4	6.0 ± 0.5	22 ± 3
**[3b][CF**_**3**_ **SO**_**3**_ **]**_**2**_	89 ± 2	>100	>100	>100
**[3c][CF**_**3**_ **SO**_**3**_ **]**_**2**_	>100	>100	>100	>100
**B–Me**	>100	>100	>100	>100
**cisplatin**	1.71 ± 0.11	13.4 ± 0.7	3.1 ± 0.3	6.6 ± 0.3

a Values are expressed as the mean ± standard deviation of three independent experiments, each performed with three technical replicates.

The monocationic complexes **[2a-b]­[CF**_**3**_ **SO**_**3**_ **]** exhibited potent cytotoxicity, and particularly **[2a]­[CF**_**3**_ **SO**_**3**_ **]** outperformed cisplatin and displayed IC_50_ values in the nanomolar range for A2780 and U87 cancer cell lines. Moving to dicationic complexes resulted in a drop of antiproliferative activity, and **[3b-c]­[CF**_**3**_ **SO**_**3**_ **]**_**2**_ proved substantially inactive against all cell lines. Conversely, **[3a]­[CF**_**3**_ **SO**_**3**_ **]**_**2**_ revealed strong cytotoxicity in A2780 and U87 cancer cells, albeit to a lower extent compared to the related compound **[2a]­[CF**_**3**_ **SO**_**3**_ **]**. These observations correlate with the Log *P*_*ow*_ values (Table 3), and highlight the crucial role played by the net charge of the complexes and the nature of the *N*-substituent. In particular, the more hydrophobic xylyl group enhances cytotoxicity even when the complex bears a +2 charge.

#### 2.4.2 Cellular Iron Uptake

The cellular uptake of the complexes was investigated in U87 cells, selected as a representative cell line. The intracellular ^56^Fe content was determined using inductively coupled plasma mass spectrometry (ICP–MS). Cells were incubated for 24 h with the complexes at a concentration of 4 μM in culture medium. This concentration was chosen to ensure reliable iron detection while maintaining sufficient cell viability for ICP–MS analysis. The highly cytotoxic monocationic complexes **[2a-b]­[CF**_**3**_ **SO**_**3**_ **]** displayed substantial intracellular accumulation (see Figure 8). Furthermore, analysis of the mitochondrial fraction showed negligible mitochondrial accumulation for **[2a]** ^**+**^ and **[2b]** ^**+**^ (Table S9). Despite its significant antiproliferative activity, **[3a]­[CF**_**3**_ **SO**_**3**_ **]**_**2**_ exhibited negligible internalization in U87 cells. Indeed, the intracellular ^56^Fe content was comparable to that of untreated cells (control) and of cells incubated with the inactive compounds **[3b-c]­[CF**_**3**_ **SO**_**3**_ **]**_**2**_ and **B–Me**. This contrasting behavior may reflect a slower uptake of **[3a]** ^**2+**^, leading to biologically effective intracellular concentrations only at later time points, or other factors, including protein binding (see below).

**Figure 8:**
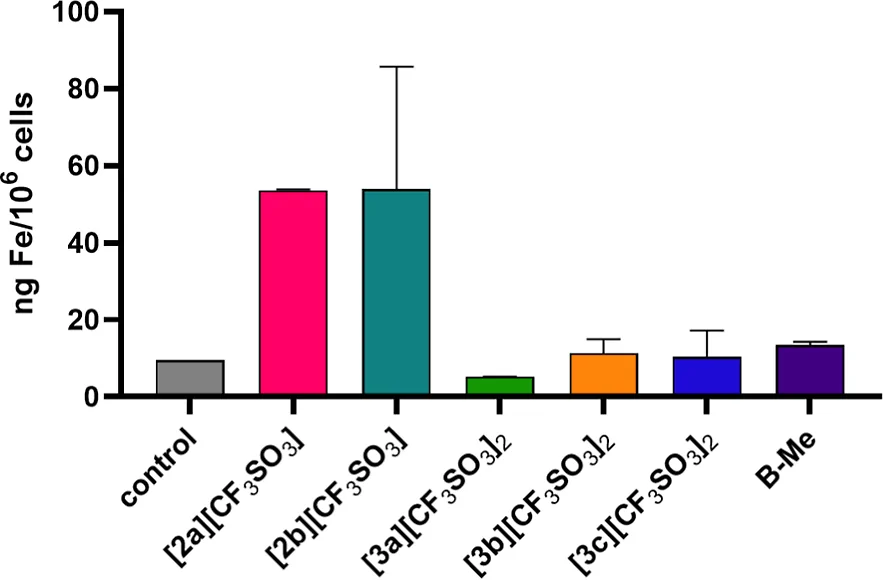
Cellular uptake of diiron complexes by U87 cells. Whole-cell accumulation after 24 h incubation with 4 μM of each complex (untreated cells served as the control), determined by ICP–MS through quantification of ^56^Fe. Iron uptake was normalized to the number of cells. Bars represent the mean of two independent replicates, and error bars indicate the corresponding standard deviations.

#### 2.4.3 Mitochondrial Dysfunction

To investigate whether the antiproliferative activity of the compounds was associated with mitochondrial dysfunction, oxygen consumption rate (OCR) was measured in U87 glioblastoma cells using the Seahorse XF Cell Mito Stress Test following 24 h treatment with the three active compounds (**[2a]­[CF**_**3**_ **SO**_**3**_ **]**, **[2b]­[CF**_**3**_ **SO**_**3**_ **]**, and **[3a]­[CF**_**3**_ **SO**_**3**_ **]**_**2**_) at concentrations corresponding to the IC_50_ values determined after 48 h exposure. Untreated cells and cisplatin-treated cells displayed comparable respiratory profiles throughout the assay, indicating that cisplatin did not significantly affect mitochondrial respiration under the experimental conditions employed. In contrast, all three diiron complexes altered mitochondrial respiratory parameters to different extents. Among the tested compounds, **[2b]­[CF**_**3**_ **SO**_**3**_ **]** induced the most pronounced reduction in basal OCR, whereas **[2a]­[CF**_**3**_ **SO**_**3**_ **]** and **[3a]­[CF**_**3**_ **SO**_**3**_ **]**_**2**_ produced more moderate decreases compared to untreated cells. Following oligomycin injection, OCR remained lower than the control in all diiron-treated groups, consistent with reduced ATP-linked respiration; as already noted for basal respiration, this reduction was comparatively milder for **[2a]­[CF**_**3**_ **SO**_**3**_ **]**- and **[3a]­[CF**_**3**_ **SO**_**3**_ **]**_**2**_-treated cells than for **[2b]­[CF**_**3**_ **SO**_**3**_ **]**-treated cells. Upon FCCP injection, however, this trend reversed: **[2b]­[CF**_**3**_ **SO**_**3**_ **]**-treated cells displayed a pronounced OCR increase, reaching maximal respiration values comparable to those of **[2a]­[CF**_**3**_ **SO**_**3**_ **]**- and **[3a]­[CF**_**3**_ **SO**_**3**_ **]**_**2**_-treated cells, whereas the latter two showed a markedly blunted response. This pattern points to a reduced spare respiratory capacity and an already-limited maximal capacity of the electron transport chain in cells treated with **[2a]­[CF**_**3**_ **SO**_**3**_ **]** and **[3a]­[CF**_**3**_ **SO**_**3**_ **]**_**2**_, and suggests that **[2b]­[CF**_**3**_ **SO**_**3**_ **]** instead primarily affects coupled, ATP-linked respiration while largely preserving the intrinsic capacity of the electron transport chain. After inhibition of mitochondrial electron transport by rotenone and antimycin A, OCR values converged in all experimental groups, confirming that the observed differences originated from mitochondrial respiration. These results demonstrate that **[2a]­[CF**_**3**_ **SO**_**3**_ **]**, **[2b]­[CF**_**3**_ **SO**_**3**_ **]**, and **[3a]­[CF**_**3**_ **SO**_**3**_ **]**_**2**_ impair mitochondrial bioenergetic function in U87 cells, with **[2a]­[CF**_**3**_ **SO**_**3**_ **]** and **[3a]­[CF**_**3**_ **SO**_**3**_ **]**_**2**_ also inducing an altered mitochondrial respiratory state, whereas cisplatin does not significantly affect mitochondrial respiration under the same experimental conditions (Figure 9).

**Figure 9:**
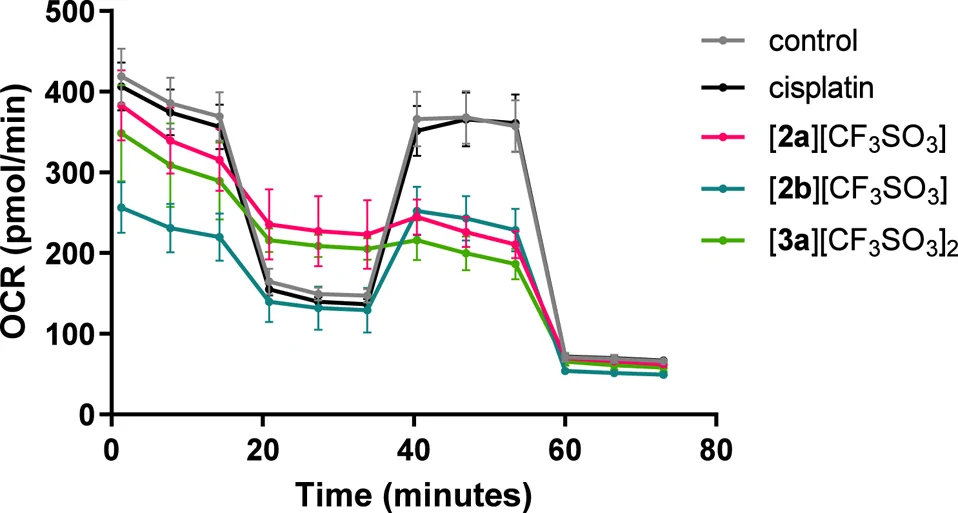
Oxygen consumption rate (OCR) profiles obtained from the Seahorse Mito Stress Test performed on U87 cells after 24 h treatment with the complexes at concentrations corresponding to their 48 h IC_50_ values. Oligomycin, FCCP, and rotenone plus antimycin A were sequentially injected by the Seahorse XF Analyzer at 18, 36, and 54 min, respectively. Data points represent the mean of six technical replicates, and error bars indicate the corresponding standard deviations.

To further investigate whether the metabolic alterations observed in the Seahorse assay were associated with mitochondrial dysfunction, the mitochondrial membrane potential (ΔΨ_*m*_) was evaluated by JC-1 staining in A549 and U87 cells. JC-1 is a membrane potential-sensitive fluorescent probe that accumulates in polarized mitochondria as red-emitting aggregates, whereas under conditions of mitochondrial depolarization it remains in its monomeric form, emitting green fluorescence. Consequently, a decrease in the aggregate-to-monomer (red/green) fluorescence ratio is indicative of loss of ΔΨ_*m*_.

Representative fluorescence images revealed a clear shift from the predominance of red fluorescent aggregates in the mitochondria of untreated cells to diffuse green fluorescence in both A549 (Figure 10a) and U87 cells (Figure 10c) after treatment with **[2a]­[CF**_**3**_ **SO**_**3**_ **]**, **[2b]­[CF**_**3**_ **SO**_**3**_ **]**, and **[3a]­[CF**_**3**_ **SO**_**3**_ **]**_**2**_, consistent with mitochondrial membrane depolarization. This qualitative observation was supported by the calculated aggregate-to-monomer fluorescence ratios, which decreased from 1.54 to 0.40, 0.60, and 0.36 in A549 cells treated with **[2a]­[CF**_**3**_ **SO**_**3**_ **]**, **[2b]­[CF**_**3**_ **SO**_**3**_ **]**, and **[3a]­[CF**_**3**_ **SO**_**3**_ **]**_**2**_, respectively (Figure 10b). Similarly, in U87 cells the ratio decreased from 0.85 in untreated cells to 0.38, 0.40, and 0.58 following treatment with **[2a]­[CF**_**3**_ **SO**_**3**_ **]**, **[2b]­[CF**_**3**_ **SO**_**3**_ **]**, and **[3a]­[CF**_**3**_ **SO**_**3**_ **]**_**2**_, respectively (Figure 10d).

**Figure 10:**
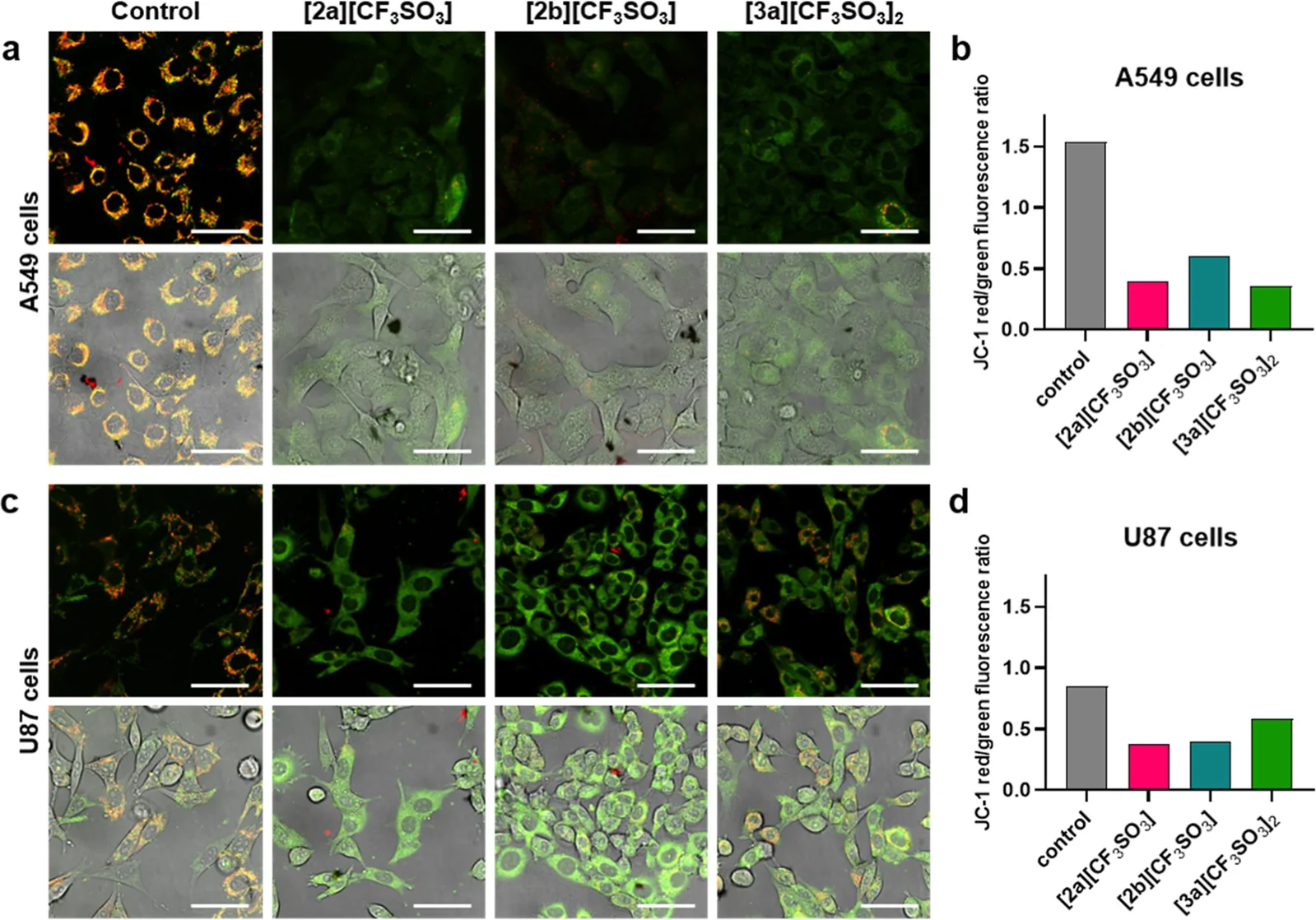
Evaluation of mitochondrial membrane potential (ΔΨ_*m*_) by JC-1 staining in A549 cells (top panel) and U87 cells (bottom panels). Representative fluorescence microscopy images of A549 (**a**) and U87 (**c**) cells stained with JC-1 following treatment with **[2a]­[CF**_**3**_ **SO**_**3**_ **]**, **[2b]­[CF**_**3**_ **SO**_**3**_ **]**, and **[3a]­[CF**_**3**_ **SO**_**3**_ **]**_**2**_. Representative merged fluorescence and bright-field images are shown below each fluorescence image. Green fluorescence represents the monomeric form of JC-1 and red fluorescence represents its aggregate form. The corresponding JC-1 aggregate to monomer fluorescence ratios (red/green fluorescence intensity), calculated from the representative images, are shown in panels **b** (A549) and **d** (U87). A decrease in the aggregate-to-monomer (i.e., red/green) fluorescence ratio indicates mitochondrial membrane depolarization. Cells were treated with compounds at concentrations corresponding to their IC_50_ values determined after 48 h exposure. Scale bars = 50 μm.

These findings support the conclusion that the three iron complexes induce mitochondrial membrane depolarization, consistent with the mitochondrial bioenergetic alterations revealed by Seahorse analysis. In light of the electrochemical results (Section 2), intracellular redox transformations of the complexes could contribute to this behavior.

To assess whether the observed mitochondrial dysfunction was accompanied by oxidative stress, intracellular ROS production was evaluated in A549 and U87 cells using the H_2_DCFDA assay. Under the experimental conditions employed, treatment with **[2a]­[CF**_**3**_ **SO**_**3**_ **]**, **[2b]­[CF**_**3**_ **SO**_**3**_ **]**, or **[3a]­[CF**_**3**_ **SO**_**3**_ **]**_**2**_ did not result in detectable intracellular ROS generation, as no increase in DCF fluorescence was observed by confocal microscopy compared with untreated cells (Figure S59). This result is noteworthy, considering that many iron-based organometallic anticancer agents are widely recognized to exert their activity through induction of intracellular ROS production (see Introduction). Importantly, even diiron­(I) complexes of type **B** (Scheme 1) have previously been shown to induce intracellular ROS production,^32,65^ indicating that complete CO/CNR substitution profoundly influences the biological properties of diiron­(I) aminocarbyne compounds.

### 2.5 Interaction with Biomolecules

Complexes **[2a]­[CF**_**3**_ **SO**_**3**_ **]** and **[3a]­[CF**_**3**_ **SO**_**3**_ **]**_**2**_ were evaluated for their binding to natural DNA and bovine serum albumin (BSA). Prior to these analyses, the stability of the complexes in a physiological buffer (ultrapure water containing NaCl 0.1 M and sodium cacodylate 0.01 M, pH = 7.0) was confirmed by UV–vis spectroscopy over a period of 90 min.

The potential binding to natural double-stranded DNA was investigated using an ethidium bromide (EB) displacement titration method. In this assay, DNA is first saturated with the fluorescent EB intercalator, after which increasing amounts of the metal complex are added. In general, a decrease in fluorescence intensity at the characteristic wavelength of intercalated EB (λ_ex_ = 520 nm, λ_em_ = 590 nm) indicates competition with the probe and suggests DNA binding by the metal complex.^74^ Figure S60 shows no significant fluorescence change for either **[2a]** ^**+**^ or **[3a]** ^**2+**^. These results suggest that the tested compounds do not significantly bind to the nucleic acid under the conditions examined.

BSA is intrinsically fluorescent, and its emission can be partially quenched when a small molecule enters its binding domains, primarily located in two hydrophobic pockets.^75^ A quenching effect was observed (Figure S61) upon the progressive addition of **[2a]** ^**+**^ or **[3a]** ^**2+**^. The fluorescence changes were analyzed (see Experimental Section), supporting the formation of a 1:1 metal–BSA adduct. The binding constants for this interaction were evaluated using HypSpec software (Figure 11), yielding logβ values of 5.53 ± 0.01 for the **[2a]** ^**+**^/BSA system and 6.35 ± 0.02 for **[3a]** ^**2+**^/BSA. These results raise the possibility that the antiproliferative activity of the complexes may be influenced by interactions with proteins.

**Figure 11:**
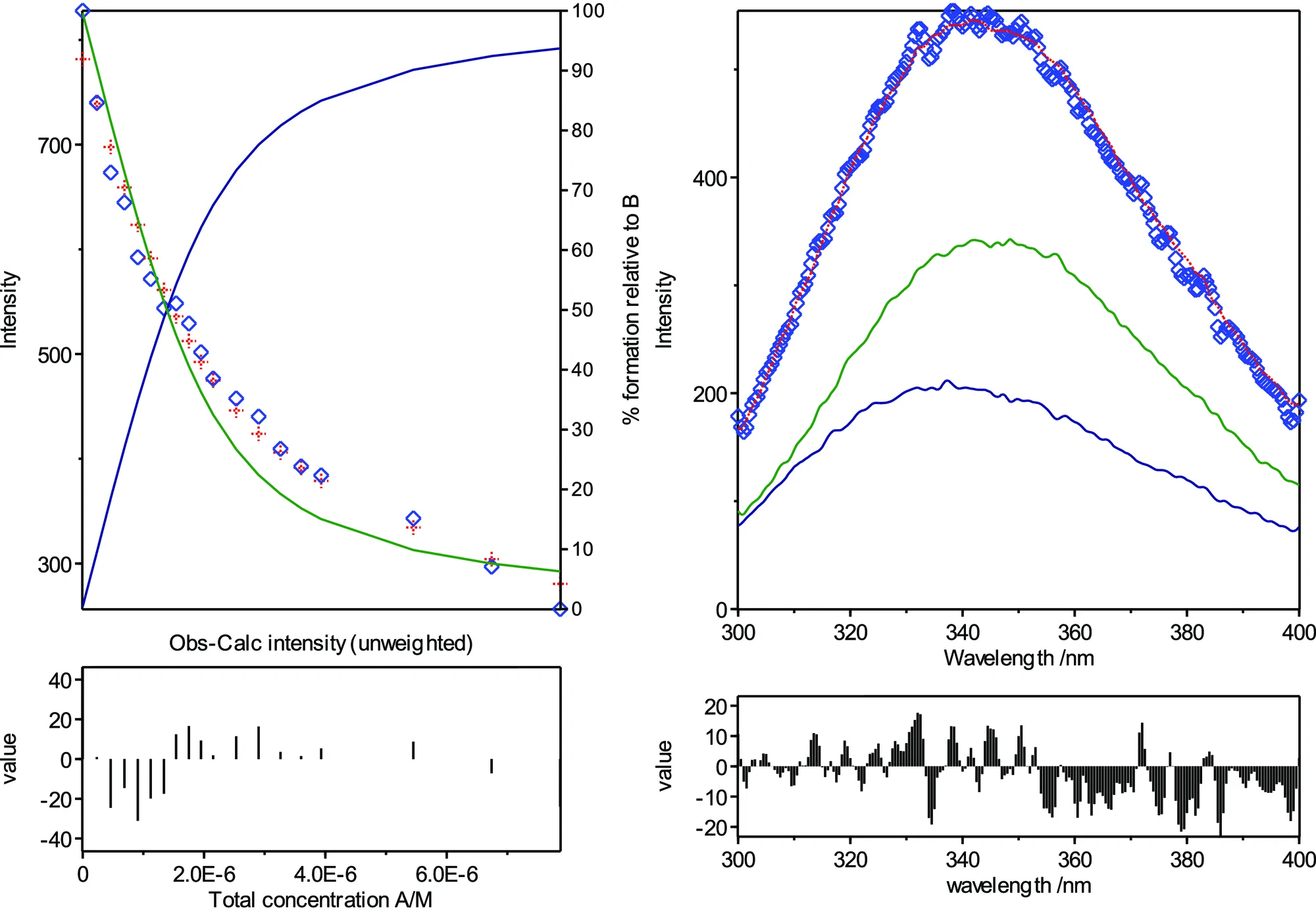
Example of data fitting by HypSpec software for the **[3a]** ^**2+**^/BSA binding experiment. C_BSA_ = 2.00 × 10^–6^ M; **[3a]** ^**2+**^ concentration from 0 to 7.87 × 10^–6^ M; 0.1 M NaCl; 0.01 M sodium cacodylate; pH 7.0; *T* = 25.0 °C; λ_ex_ = 280 nm. Left: binding isotherm and percentage formation diagram. Right: overall spectrum at a representative addition together with the spectral contributions of free and bound BSA. In both: free BSA in green, [**3a**]^2+^-bound BSA in blue, experimental data as blue open diamonds and calculated fit as red crosses; the bottom panels show the residuals.

## 3 Concluding Remarks

This work describes the straightforward synthesis, characterization, and preliminary anticancer evaluation of two series of isocyanide complexes, prepared from the readily available [Fe_2_Cp_2_(CO)_4_], featuring one or two aminocarbyne ligands and carrying net charges of +1 and +2, respectively. These compounds represent the first examples of noncarbonyl organoiron­(I) complexes investigated for anticancer activity. Cellular studies revealed a general correlation between cytotoxicity and lipophilicity, with the monocationic complexes showing the highest potency and one of them (**[2a]­[CF**_**3**_ **SO**_**3**_ **]**) clearly outperforming cisplatin. Mechanistic studies point to a mechanism of action distinct from that of classical platinum chemotherapeutic drugs, lacking significant DNA interaction and instead involving mitochondrial dysfunction, as evidenced by impaired mitochondrial respiration and loss of mitochondrial membrane potential. Moreover, the investigated compounds did not induce an appreciable increase in intracellular ROS levels, in contrast to other types of organoiron anticancer agents, including related diiron aminocarbyne carbonyl complexes.

These findings indicate that incorporating isocyanide ligands in place of CO ligands is a key design element that confers distinct biological properties. In addition, their combination with Cp and aminocarbyne ligands confers remarkable stability to the diiron framework, even in physiological-like media. The successful application of the synthetic protocol to a small panel of isocyanides bearing substituents with diverse electronic and steric properties suggests that further modulation of the physicochemical and biological properties is feasible using different isocyanides, thereby opening new perspectives for the development of this class of organometallic complexes for medicinal applications. Future studies, including in vivo investigations, could be performed on lead compounds of this class after a fine structural optimization, allowing a clearer evaluation of their therapeutic potential and safety profile.

## 4 Experimental Section

### 4.1 Methods and Materials

Reagents were purchased from Merck and TCI Europe and used as received. Methyl isocyanide^76^ and complex [Fe_2_Cp_2_(CO)_2_(μ-CO) (μ-CNMe_2_)]­[CF_3_SO_3_], **B–Me**,^32^ were synthesized according to the reported literature procedure. Solvents were obtained from Merck. All reactions were performed under a nitrogen atmosphere using anhydrous solvents. Toluene, dichloromethane and diethyl ether were purified using an SPS-5 MBraun solvent purification system. Xylene was dried by filtration through activated alumina and stored under N_2_. Chromatographic separations were carried out in air using neutral alumina (Merck) as stationary phase and technical-grade solvents. Infrared spectra of solutions were recorded on a PerkinElmer Spectrum 100 FT-IR spectrometer using a CaF_2_ liquid transmission cell (2300–1500 cm^–1^ range). IR spectra of solid samples (650–4000 cm^–1^) were recorded on an Agilent Cary 630 instrument equipped with a UATR sampling accessory (ZnSe crystal). UV–vis spectra were recorded on an Ultraspec 2100 Pro spectrophotometer using 1 cm PMMA cuvettes. IR and UV–vis spectra were processed with Spectragryph software. NMR spectra were recorded at 298 K on a Jeol JNM-ECZ500R spectrometer equipped with a Royal HFX Broadband probe. Chemical shifts (δ, ppm) are referenced to residual solvent signals (^1^H and ^13^C).^77^ Signal assignments were supported by ^1^H–^13^C *gs*-HSQC and *gs*-HMBC correlation experiments. Stereochemical assignments were established by 1D NOESY experiments involving selective irradiation of cyclopentadienyl resonances (mixing time 750 ms, relaxation delay 1 s, excitation bandwidth 15 Hz). Signals corresponding to secondary isomeric species, when observable, are reported in italics. Elemental (CHNS) analyses were performed on a Vario MICRO cube analyzer (Elementar).

## 5 Synthesis and Characterization of Diiron Complexes

[Fe_2_Cp_2_(CNXyl)_4_], **1a** (Figure 12).

**Figure 12:**
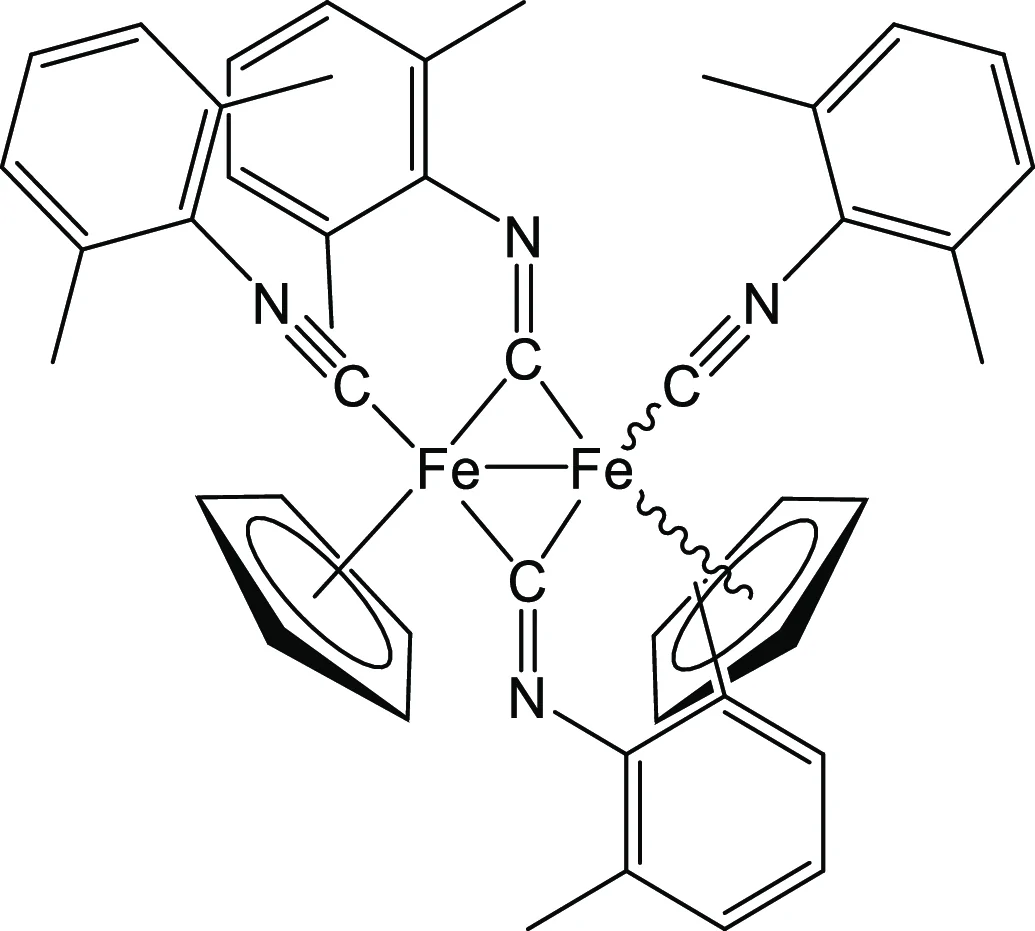
Structure of **1a**.

The title compound was prepared by a modified literature procedure, in which **1a** was isolated in 60% yield after filtration through cellulose and subsequent recrystallization from THF or benzene/hexane.^50^ A solution of [Fe_2_Cp_2_(CO)_4_] (452 mg, 1.28 mmol) in toluene (20 mL) was treated with 2,6-dimethylphenyl isocyanide (670 mg, 5.11 mmol, ≈4 equiv). The reddish-brown solution was heated for 1 h at reflux, during which it turned deep green. IR monitoring confirmed the complete consumption of [Fe_2_Cp_2_(CO)_4_] and the formation of **1a**. The solvent was removed under reduced pressure, then the residue was dissolved in CH_2_Cl_2_ and filtered through Celite. Evaporation of the solvent followed by washing of the resulting solid with hexane (50 mL) afforded **1a** as a green powder. Yield 881 mg (90%). Anal. Calcd for C_46_H_46_Fe_2_N_4_: C, 72.07; H, 6.05; N, 7.31. Found: C, 71.48; H, 5.89; N, 7.34. IR (toluene): ṽ/cm^–1^ = 2032s, 1998s (C≡N), 1669vs (μ-CN). IR (CH_2_Cl_2_): ṽ/cm^–1^ = 2039s, 2000s (C≡N), 1666vs (μ-C = N). ^1^H NMR (C_6_D_6_): δ/ppm = 7.04–6.64 (m, 12H, CH^Xyl^), 4.72, 4.61 (s, 10H, Cp); 2.56, 2.35, 2.06 (s, 24H, Me^Xyl^). ^1^H NMR (CDCl_3_): δ/ppm = 7.08–6.90 (m, 12H, CH^Xyl^); 4.62, 4.59 (s, 10H, Cp); 2.44, 2.43, 2.06 (s, 24H, Me^Xyl^). *Cis/trans* ratio = 1.3.

[Fe_2_Cp_2_(CNXyl)_3_{CN­(Me) (Xyl)}]­[CF_3_SO_3_], **[2a]­[CF_3_SO_3_]** (Figure 13).

**Figure 13:**
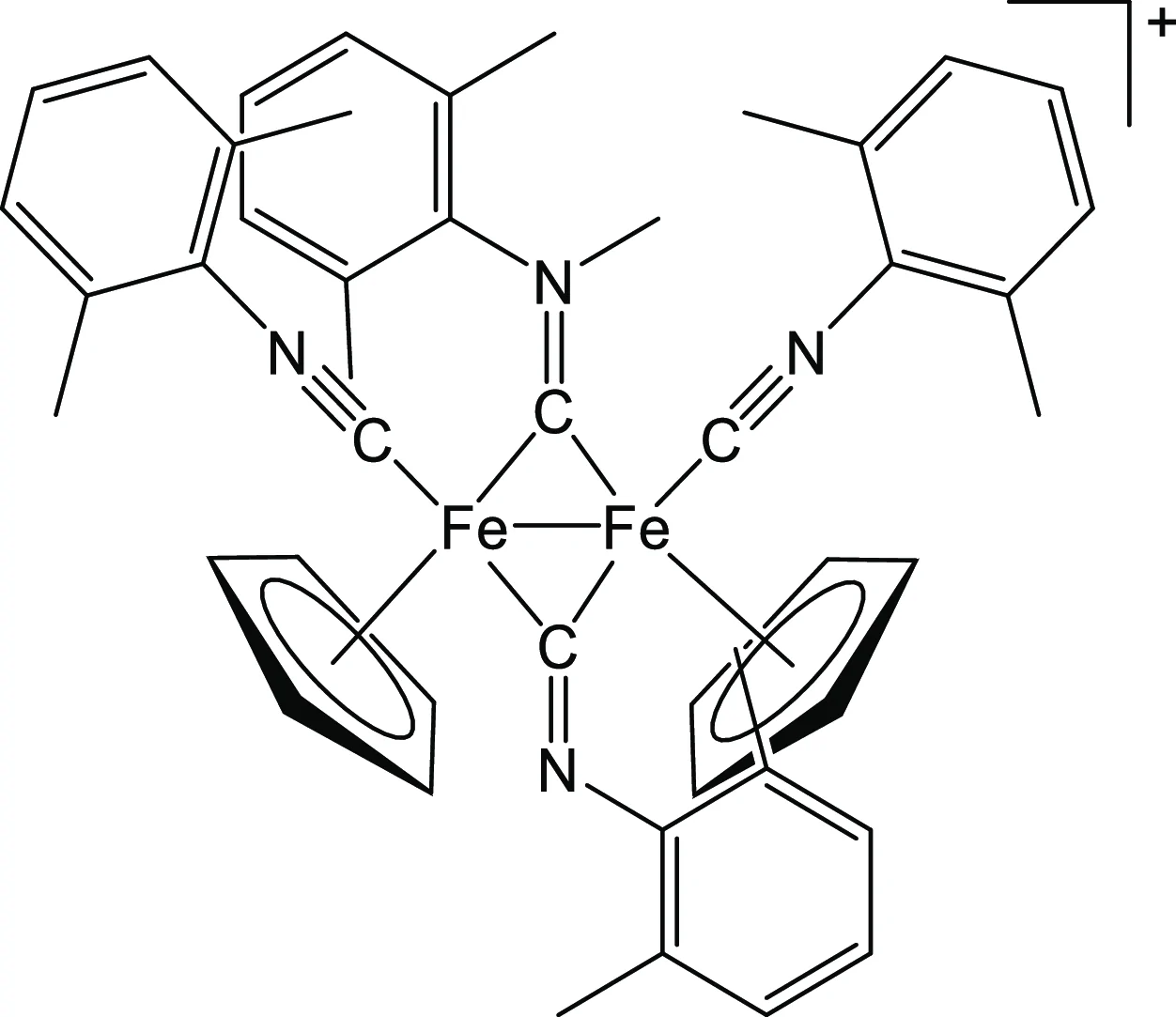
Structure of **[2a]** ^**+**^.

To a light green suspension of **1a** (133 mg, 0.174 mmol) in CH_2_Cl_2_ (25 mL), methyl triflate (19 μL, 0.17 mmol, 1.0 equiv) was added dropwise at room temperature. The mixture was stirred for 1 h, during which the color gradually deepened. The reaction mixture was then loaded directly onto an alumina column (d = 2.5 cm, h = 6 cm). Elution with CH_2_Cl_2_/THF mixtures removed nonpolar impurities, then CH_3_CN/MeOH (10:1 v/v) afforded a dark-green fraction corresponding to the title compound. The solvent was evaporated under reduced pressure, and the residue washed with hexane (20 mL) to give the product as a dark-green powder. Yield 149 mg (92%). Anal. Calcd for C_48_H_49_F_3_Fe_2_N_4_O_3_S: C, 61.95; H, 5.31; N, 6.02; S, 3.45. Found: C, 61.69; H, 5.16; N, 6.05; S, 3.53. IR (CH_2_Cl_2_): ṽ/cm^–1^ = 2099vs, 2075sh (C≡N), 1780br-m (μ-CNXyl), 1499w (μ-CNXylMe). IR (solid state): ṽ/cm^–1^ = 2076s, 2061s (C≡N), 1701m (μ-CNXyl), 1491w (μ-CNXylMe), 1255vs (SO_3_). ^1^H NMR (CDCl_3_): δ/ppm = 7.36–7.30 – 7.14–6.88 (m, 12H, arom CH); 5.18, 4.42 (s, 10H, Cp); 4.61 (s, 3H, NMe); 2.83, 2.34, 2.33, 1.97, 1.72 (s, 24H, Me^Xyl^). ^13^C­{^1^H} NMR (CDCl_3_): δ/ppm = 338.5 (μ-CNXylMe); 229.4 (μ-CNXyl); 174.9, 173.9 (C≡N); 148.0, 142.7, 134.8 (arom *N*–C); 134.5, 134.2, 133.6, 132.3 (arom Me–C); 129.4, 129.3, 129.1, 129.0, 128.9, 128.4, 128.3, 128.2, 127.8, 126.7, 123.5 (arom CH); 121.04 (q, ^1^J_CF_ = 320.8 Hz, CF_3_SO_3_); 89.0, 86.7 (Cp); 54.7 (NMe); 19.6, 19.2, 18.6, 18.1 (Me^Xyl^). Crystals of **[2a]­[CF**_**3**_ **SO**_**3**_ **]** suitable for single-crystal X-ray diffraction were obtained by slow diffusion of Et_2_O into a CH_2_Cl_2_ solution at 4 °C.

[Fe_2_Cp_2_(CNCy)_3_{CN­(Me) (Cy)}]­[CF_3_SO_3_], **[2b]­[CF_3_SO_3_]** (Figure 14).

**Figure 14:**
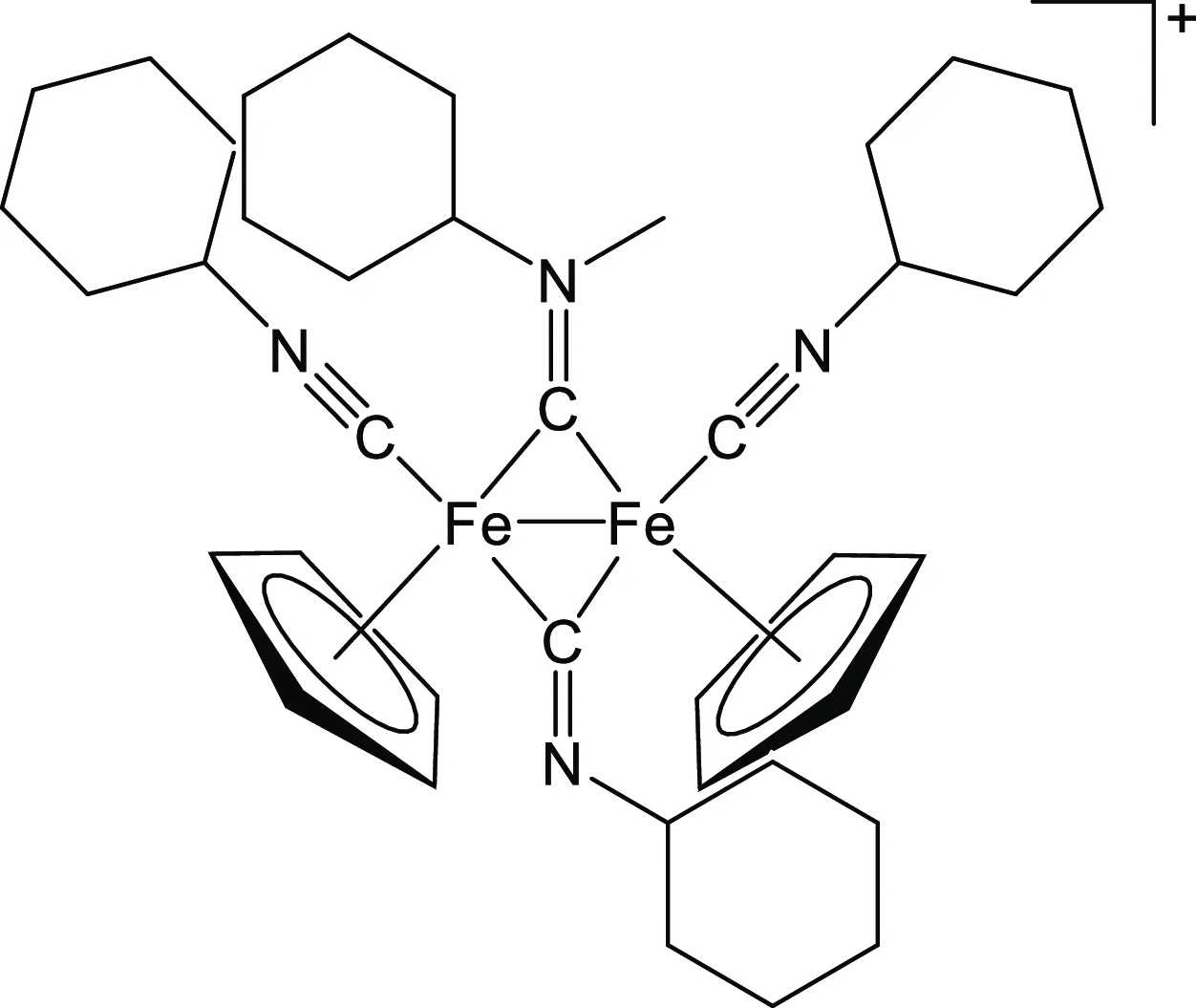
Structure of [**2b**]^+^.

A solution of [Fe_2_Cp_2_(CO)_4_] (200 mg, 0.565 mmol) in xylene (7 mL) was treated with cyclohexyl isocyanide (0.57 mL, 4.52 mmol, 8 equiv). The reddish-brown solution was heated to reflux for 3 h, affording a dark-red solution together with a black insoluble precipitate, attributed to unidentified byproducts. IR monitoring confirmed complete consumption of [Fe_2_Cp_2_(CO)_4_] and formation of **1b**. IR (xylene): ṽ/cm^–1^ = 2138vs, 2097vs (C≡N), 1661vs (μ-CN). IR (CH_2_Cl_2_): v/cm^–1^ = 2146s, 2112vs, 2085vs (C≡N), 1647vs (μ-CN). After removal of xylene under reduced pressure, CH_2_Cl_2_ (ca. 10 mL) was added. Methyl triflate (0.064 mL, 0.565 mmol, 1 equiv) was added dropwise at room temperature, and the mixture was stirred for 24 h, during which the color gradually turned dark green. The reaction mixture was loaded onto an alumina column (d = 3.5 cm, h = 6 cm). Elution with CH_2_Cl_2_/THF mixtures removed impurities, then CH_3_CN/THF (3:1 v/v) afforded a dark-green band corresponding to the title compound. The solvent was evaporated under reduced pressure, and the residue washed twice with hexane (50 mL) to give the product as a dark-green powder. Yield 170 mg (36%). Anal. Calcd for C_40_H_57_F_3_Fe_2_N_4_O_3_S: C, 57.01; H, 6.82; N, 6.65; S, 3.81. Found: C, 56.85; H, 6.72; N, 6.53; S, 3.93. IR (CH_2_Cl_2_): ṽ/cm^–1^ = 2162sh, 2137vs (C≡N), 1747m, 1718m (μ-CNCy), 1531m (μ-CNCyMe). IR (solid state): ṽ/cm^–1^ = 2126s (C≡N), 1701m (μ-CNCy), 1533w (μ-CNCyMe), 1250vs (SO_3_). ^1^H NMR (acetone-d_6_): δ/ppm = 5.06 (tt, ^3^J_HH_ = 12.0 Hz, 3.4 Hz, 1H, μ-CNCH ^Cy^Me); 4.94, 4.90 (s, 10H, Cp); 4.09 (s, 3H, NMe); 3.94 (m, 1H, μ-CNCH ^Cy^), 3.60 (m, 2H, t-CNCH ^Cy^); 2.45 (d, ^3^J_HH_ = 12.3 Hz), 1.96–1.27 (m) (40H, CH_2_^Cy^). ^13^C­{^1^H} NMR (acetone-d_6_): δ/ppm = 331.6 (μ-CNCyMe); 230.1 (μ-CNCy); 163.4, 162.4 (C≡N); 122.3 (q, ^1^J_CF_ = 322.1 Hz, CF_3_SO_3_); 87.2, 87.1 (Cp); 76.6 (μ-CNCH^Cy^Me); 69.7 (μ-CNCH^Cy^); 55.7, 55.4 (t-CNCH^Cy^); 44.6 (NMe); 35.5, 33.8, 33.6, 32.0, 31.8, 26.5, 26.4, 26.3, 25.9, 25.5, 25.2, 23.4 (CH_2_^Cy^).

[Fe_2_Cp_2_(CNXyl)_2_{CN­(Me) (Xyl)}_2_]­[CF_3_SO_3_]_2_, **[3a]­[CF_3_SO_3_]_2_** (Figure 15).

**Figure 15:**
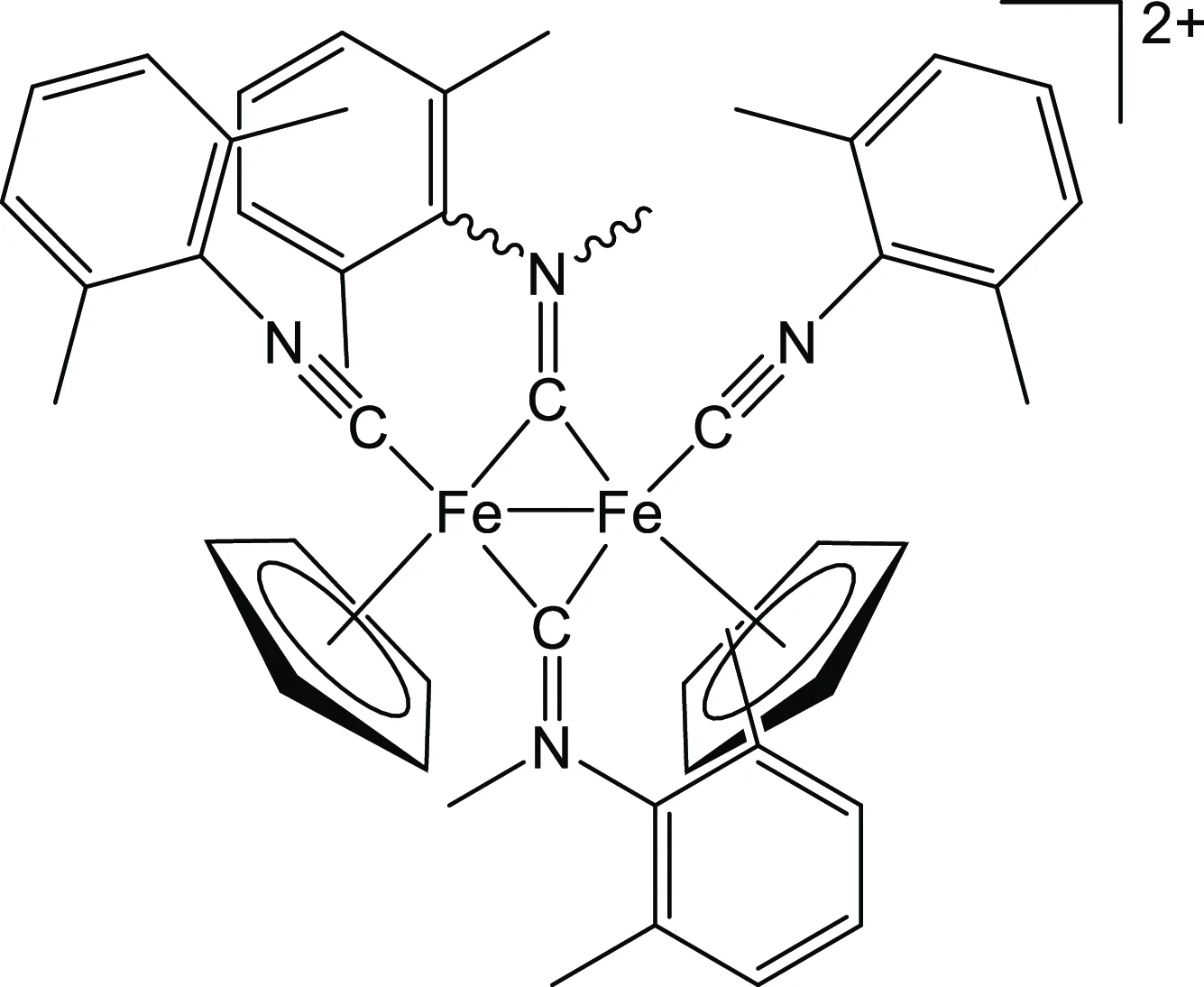
Structure of **[3a]** ^**2+**^ (wavy bonds denote *syn*/*anti* isomerism).

A solution of **1a** (107 mg, 0.140 mmol) in toluene (30 mL) was treated with methyl triflate (38 μL, 0.35 mmol, 2.5 equiv) and heated at reflux for 3 h. During this time, the product precipitated as a green solid. The precipitate was isolated by filtration from the yellow mother liquor, washed with toluene and hexane, and dried under reduced pressure to afford **[3a]­[CF**_**3**_ **SO**_**3**_ **]**_**2**_ as a green solid. Yield 131 mg (85%). Anal. Calcd for C_50_H_52_F_6_Fe_2_N_4_O_6_S_2_: C, 54.85; H, 4.79; N, 5.12; S, 5.86. Found: C, 54.57; H, 4.64; N, 5.03; S, 6.02. IR (CH_3_CN): ṽ/cm^–1^ = 2132vs, 2118sh (C≡N), 1527m (μ-CN). IR (solid state): ṽ/cm^–1^ = 2118s (C≡N), 1522s (μ-CN), 1255vs (SO_3_). ^1^H NMR (acetone-d_6_): δ/ppm = 7.65–7.18 (m, 12H, arom-CH); 6.18, 5.48, 4.82 (s, 10H, Cp); 5.00, 4.74 (s, 6H, NMe); 2.90, 2.89, 2.60, 2.26, 2.23, 2.09 (s, 24H, Me^Xyl^). *Cis*-*anti*/*cis*-*syn* ratio: 1.5. ^13^C­{^1^H} NMR (acetone-d_6_): δ/ppm = 327.6, 327.0 (μ-CN); 164.8 (C≡N); 147.9, 147.5 (arom *N*–C); 136.6, 135.7, 134.6, 133.0, 132.9 (arom Me–C + arom *N*–C); 131.2, 131.0, 130.9, 130.5, 130.4, 129.5, 128.7, 128.3 (arom-CH); 93.2, 92.2, 91.2 (Cp); 57.7, 56.3 (NMe); 20.3, 19.6, 19.5, 18.5 (Me^Xyl^). Crystals of *cis*-*anti*-(SS)-**[3a]­[CF**_**3**_ **SO**_**3**_ **]**_**2**_ suitable for single-crystal X-ray diffraction were obtained by slow diffusion of Et_2_O into a CH_2_Cl_2_ solution at −30 °C.

[Fe_2_Cp_2_(CNCy)_2_{CN­(Me) (Cy)}_2_]­[CF_3_SO_3_]_2_, **[3b]­[CF_3_SO_3_]**_2_ (Figure 16).

**Figure 16:**
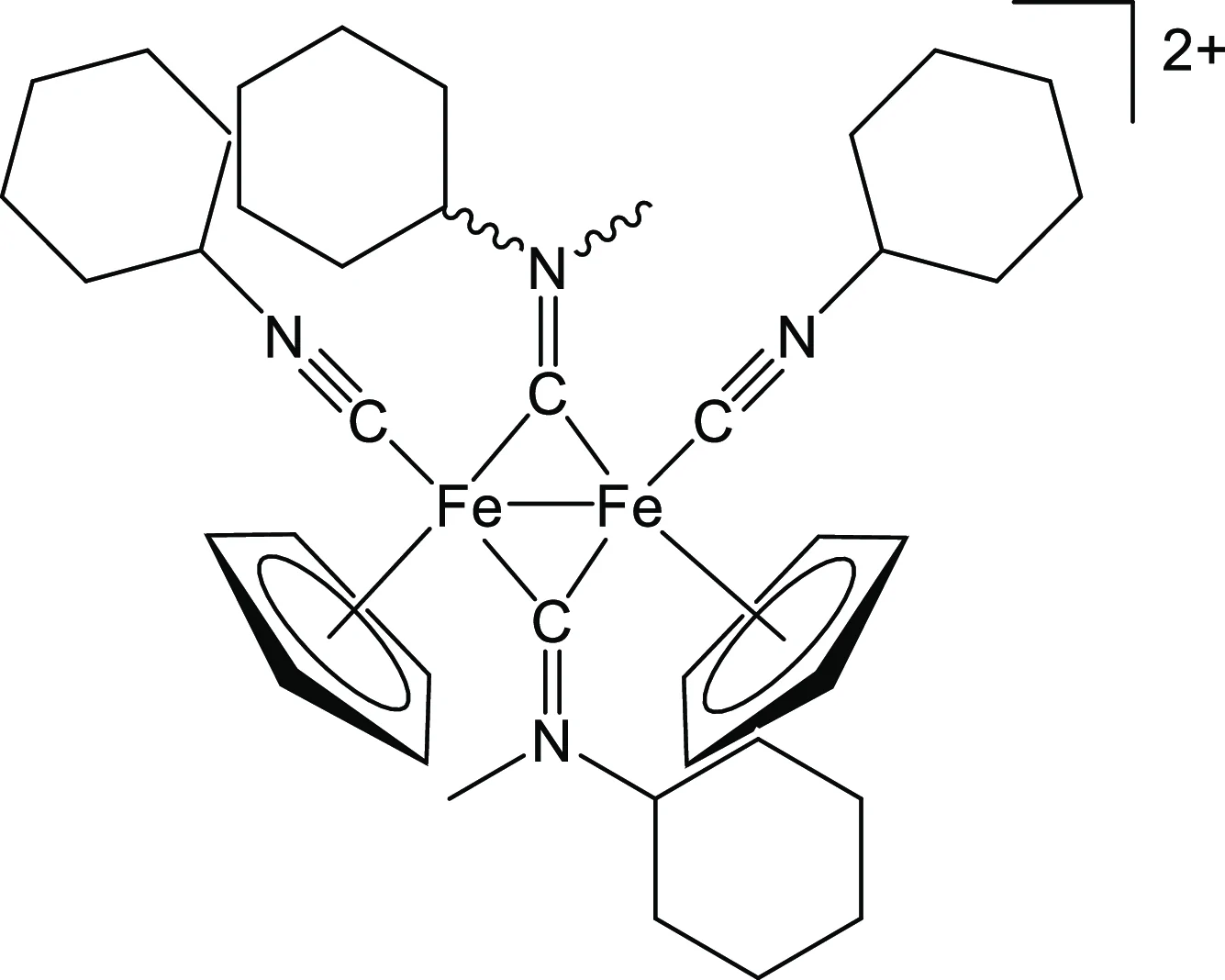
Structure of [**3b**]^**2+**^ (wavy bonds denote *syn*/*anti* isomerism).

A solution of [Fe_2_Cp_2_(CO)_4_] (290 mg, 0.819 mmol) in xylene (20 mL) was treated with cyclohexyl isocyanide (0.84 mL, 6.6 mmol, 8 equiv). The reddish-brown mixture was stirred for 3 h at reflux, affording a dark red solution together with a black insoluble precipitate, attributed to unidentified byproducts. IR monitoring confirmed complete consumption of [Fe_2_Cp_2_(CO)_4_] and formation of **1b**. Methyl triflate (0.19 mL, 1.64 mmol, 2.0 equiv) was then added, and the mixture was stirred at reflux for a further 3 h. After removal of volatiles under reduced pressure, the residue was dissolved in CH_2_Cl_2_ (15 mL) and loaded onto an alumina column (d = 3.5 cm, h = 7 cm). Elution with CH_2_Cl_2_/THF mixtures removed impurities, and subsequent elution with CH_3_CN/MeOH (10:1 v/v) gave a green fraction corresponding to the title compound. The solvent was evaporated under reduced pressure, and the residue washed with toluene and hexane to afford **[3b]­[CF**_**3**_ **SO**_**3**_ **]**_**2**_ as a green powder. Yield 298 mg (36%). Anal. Calcd for C_42_H_60_F_6_Fe_2_N_4_O_6_S_2_: C, 50.11; H, 6.01; N, 5.57; S, 6.37. Found: C, 49.70; H, 6.05; N, 5.46; S, 6.22. IR (MeCN): ṽ/cm^–1^ = 2170vs (C≡N), 1558m (μ-CN). IR (solid state): ṽ/cm^–1^ = 2161s, 2153sh (C≡N), 1551s (μ-CN), 1262vs (SO_3_). ^1^H NMR (CD_3_NO_2_): δ/ppm = 5.33, 5.32, 5.28 (s, 10H, Cp); 4.79 (tt, ^3^J_HH_ = 12.0 Hz, 3.4 Hz, 2H, μ-CNCH ^Cy^); 4.09, 4.07 (s, 6H, Me); 3.67 (m, 2H, CH^Cy^); 2.43 (d, ^3^J_HH_ = 12.6 Hz), 2.13–1.19 (m) (40 H, CH_2_^Cy^). *Cis*-*anti*/*cis*-*syn* ratio: 9. ^13^C­{^1^H} NMR (CD_3_NO_2_): δ/ppm = 317.6 (μ-CN); 150.6, 149.9 (C≡N); 121.1 (q, ^1^J_CF_ = 320.7 Hz, CF_3_SO_3_); 89.8, 89.5, 89.2 (Cp); 77.8, 77.3 (μ-CNCH^Cy^); 57.2, 56.6, 56.1 (CH^Cy^); 45.1, 44.9 (NMe); 33.0, 32.7, 31.1, 30.8, 30.6, 26.0, 25.4, 25.3, 25.0, 24.8, 24.3, 23.4, 23.0 (CH_2_^Cy^). A crop of crystals suitable for single-crystal X-ray diffraction, containing *cis*-*anti*-(RR)-[**3b**]^2+^, *cis*-*anti*-(SS)- [**3b**]^2+^ and *cis-*syn-[**3b**]^2+^, was obtained from vapor diffusion of diethyl ether into a nitromethane solution of **3b**.

[Fe_2_Cp_2_(CNMe)_2_(μ-CNMe_2_)_2_]­[CF_3_SO_3_]_2_, **[3c]­[CF_3_SO_3_]_2_** (Figure 17).

**Figure 17:**
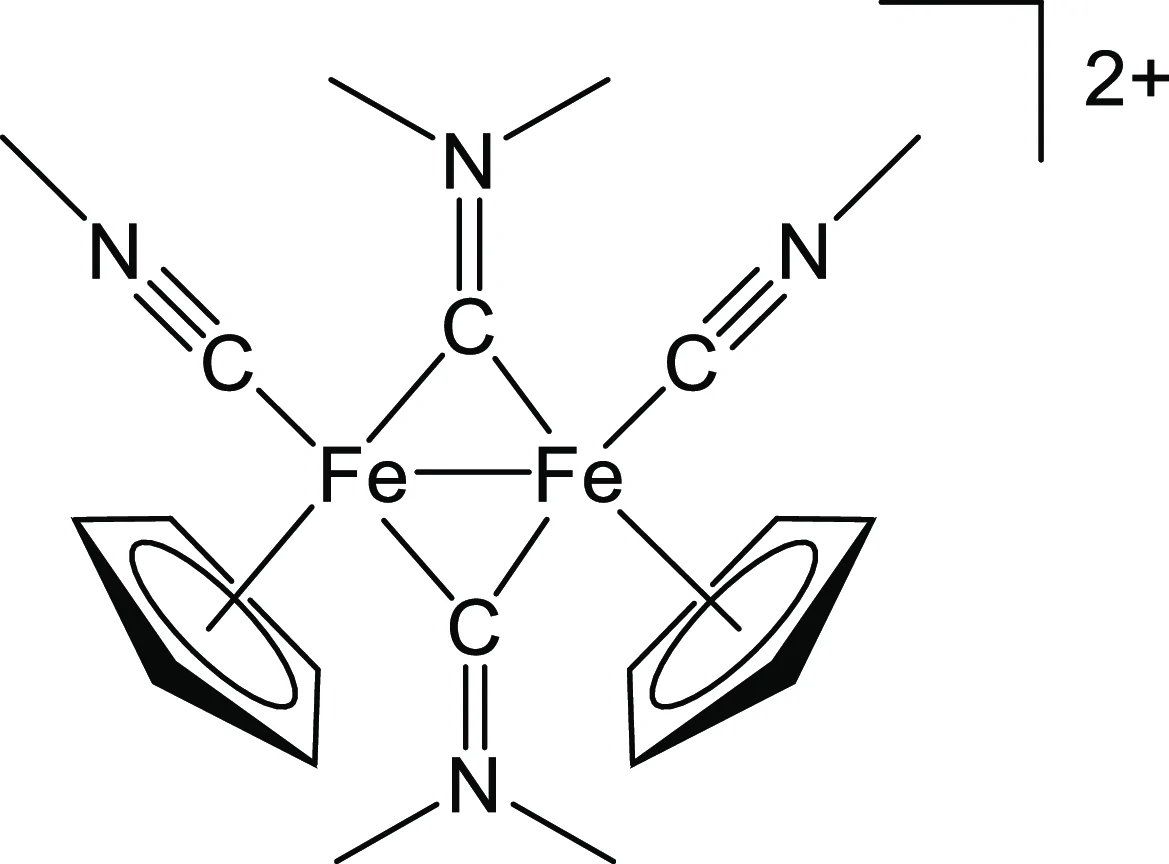
Structure of **[3c]** ^**2+**^.

The title compound was previously obtained from [Fe_2_Cp_2_(CN)_2_(CNMe_2_)_2_]·H_2_O and methyl triflate through an eight-step sequence starting from [Fe_2_Cp_2_(CO)_4_], in <12% overall yield, and characterized by IR, ^1^H and ^13^C NMR spectroscopy.^52^

A solution of [Fe_2_Cp_2_(CO)_4_] (225 mg, 0.636 mmol) in xylene (ca. 10 mL) was treated with methyl isocyanide (756 μL, 12.7 mmol, 20 equiv). The dark-red mixture was heated at reflux for 16 h, during which it turned dark-pink and a black insoluble precipitate formed, attributed to unidentified byproducts. IR monitoring confirmed complete consumption of [Fe_2_Cp_2_(CO)_4_] and formation of **1c**, which was not isolated. IR (xylene): ṽ/cm^–1^ = 2161vs, 2125s (t-C≡N), 1679sh, 1664s (μ-CN). The isolation of **1c** (50–60% yield) from [Fe_2_Cp_2_(CO)_4_] was previously documented using an 8-fold molar excess of the isocyanide, to afford a mixture of [Fe_2_Cp_2_(CO) (CNMe)_3_] and **1c** after 3 h heating in boiling xylene.^39,48^ The suspension obtained was then treated with methyl triflate (140 μL, 1.27 mmol, 2.0 equiv) and heated at reflux for 2 h. After removal of volatiles under reduced pressure, the residue was dissolved in acetonitrile and subjected to alumina column chromatography (d = 3.5 cm, h = 4 cm). The solvent was removed under reduced pressure, and the product was extracted from the residue with CH_2_Cl_2_ to afford pure **[3c]­[CF**_**3**_ **SO**_**3**_ **]**_**2**_ as a green solid. Yield 187 mg (40%). Anal. Calcd for C_22_H_28_F_6_Fe_2_N_4_O_6_S_2_: C, 35.98; H, 3.84; N, 7.63; S, 8.73. Found: C, 35.76; H, 3.76; N, 7.49; S, 8.58. IR (MeCN): ṽ/cm^–1^ = 2209vs (t-C≡N), 1596w (μ-CN). IR (solid state): ṽ/cm^–1^ = 2197s (C≡N), 1590s (μ-CN), 1260vs (SO_3_). ^1^H NMR (CD_3_CN): δ/ppm = 5.18 (s, 10H, Cp); 4.05 (s, 12H, μ-CNMe_2_); 3.09 (s, 6H, t-CNMe). ^13^C­{^1^H} NMR (CD_3_CN): δ/ppm = 318.7 (μ-CN); 149.2 (C≡N); 122.1 (q, ^1^J_CF_ = 321 Hz, CF_3_SO_3_); 89.8 (Cp); 53.3 (μ-CNMe_2_); 31.4 (t-CNMe).

### 5.1 X-Ray Crystallography

Crystal data and collection details for **[2a]­[CF**_**3**_ **SO**_**3**_ **]**, *cis*-*anti*-**[3a]­[CF**_**3**_ **SO**_**3**_ **]**_**2**_, *cis-anti*-(RR)-**[3b]­[CF**_**3**_ **SO**_**3**_ **]**_**2**_ · 0.5H_2_O, *cis-anti*-(SS)-**[3b]­[CF**_**3**_ **SO**_**3**_ **]**_**2**_ and *cis-syn-* **[3b]­[CF**_**3**_ **SO**_**3**_ **]**_**2**_ are summarized in Table 5. Data for *cis-anti*-**[3a]­[CF**_**3**_ **SO**_**3**_ **]**_**2**_ and *cis-anti*-(RR)-**[3b]­[CF**_**3**_ **SO**_**3**_ **]**_**2**_ **·** 0.5H_2_O were collected on a Bruker APEX II diffractometer equipped with a PHOTON2 detector using Mo–Kα radiation. Data for **[2a]­[CF**_**3**_ **SO**_**3**_ **]** were collected on a Rigaku XtaLAB Synergy-S diffractometer using Cu–Kα radiation. Data for *cis-anti*-(SS)-**[3b]­[CF**_**3**_ **SO**_**3**_ **]**_**2**_ and *cis-syn-* **[3b]­[CF**_**3**_ **SO**_**3**_ **]**_**2**_ were collected on a Bruker D8 Venture diffractometer equipped with a Mo Kα microfocus source (λ = 0.71073 Å) and a Photon II 2D detector. Data were corrected for Lorentz polarization and absorption effects (empirical absorption correction SADABS).^78^ Structures were solved by direct methods and refined by full-matrix least-squares based on all data using *F* ^2^.^79^ Hydrogen atoms were fixed at calculated positions and refined by a riding model. All non-hydrogen atoms were refined with anisotropic displacement parameters. Crystals of *cis-anti*-(RR)-**[3b]­[CF**_**3**_ **SO**_**3**_ **]**_**2**_ **·** 0.5H_2_O were found to be nonmerohedrally twinned. The TwinRotMat routine of PLATON was used to determine the twinning matrices and to write the reflection data file containing the four twin components.^80^ Refinement was performed using the instruction HKLF 5 in SHELXL and three BASF parameters, which were refined as 0.24(5), 0.21(8) and 0.13(8). In *cis-anti*-(SS)-**[3b]­[CF**_**3**_ **SO**_**3**_ **]**_**2**_ and *cis-syn-* **[3b]­[CF**_**3**_ **SO**_**3**_ **]**_**2**_, significant disorder was observed in several CF_3_SO_3_^–^ anions and in some cyclohexyl groups, and their electron densities were modeled over multiple positions. The occupancies of all atoms involved were freely refined using the FVAR instructions. EADP constraints were applied to minimize excessive anisotropy of C, F, and O atoms in specific positions, while DFIX and DANG restraints were employed to optimize the geometry of the atoms within the triflate anions. For *cis-anti*-(SS)-**[3b]­[CF**_**3**_ **SO**_**3**_ **]**_**2**_, stabilization of the refinement required the use of the TWIN and BASF instructions, with the latter converging to a final value of 0.0839. Although no clear macroscopic indications of twinning were apparent in the overall reciprocal lattice analysis, the precession image along the h0l plane revealed elongated and partially split reflections (Figure S34), consistent with nonmerohedral twinning.

**Table 5:** Crystal Data and Measurement Details for **[2a]­[CF**_**3**_ **SO**_**3**_ **]**, *cis*-*anti*-**[3a]­[CF**_**3**_ **SO**_**3**_ **]**_**2**_, *cis-anti*-(RR)-**[3b]­[CF**_**3**_ **SO**_**3**_ **]**_**2**_ · 0.5H_2_O, *cis-anti*-(SS)-**[3b]­[CF**_**3**_ **SO**_**3**_ **]**_**2**_ and *cis-syn-* **[3b]­[CF**_**3**_ **SO**_**3**_ **]**_**2**_

	[2a][CF_3_SO_3_]	*cis*-*anti*-[3a][CF_3_SO_3_]_2_	*cis-anti*-(RR)-[3b][CF_3_SO_3_]_2_ · 0.5H_2_O	*cis-anti*-(SS)-[3b][CF_3_SO_3_]_2_	*cis-syn-*[3b][CF_3_SO_3_]_2_
Formula	C_48_H_49_F_3_Fe_2_N_4_O_3_S	C_50_H_52_F_6_Fe_2_N_4_O_6_S_2_	C_42_H_61_F_6_Fe_2_N_4_O_6.5_S_2_	C_42_H_60_F_6_Fe_2_N_4_O_6_S_2_	C_42_H_60_F_6_Fe_2_N_4_O_6_S_2_
FW, g·mol^–1^	930.67	1094.77	1015.76	1006.76	1006.76
T, K	100(2)	100(2)	100(2)	273(2)	100(2)
λ, Å	1.54184	0.71073	0.71073	0.71073	0.71073
Crystal system	Orthorhombic	Monoclinic	Monoclinic	Orthorhombic	Orthorhombic
Space group	*Pca*2_1_	*C*2/*c*	*P*2	*P2*_1_2_1_2	*Pbcn*
*a*, Å	16.4450(8)	19.3986(9)	11.3585(18)	16.4192(6)	16.6826(5)
*b,* Å	18.4852(7)	14.5889(6)	12.5631(19)	11.7475(4)	23.8604(7)
*c*, Å	14.0873(7)	18.7218(8)	16.495(3)	12.6155(4)	46.4615(12)
β,deg	90	113.8050(10)	93.048(7)	90.00	90.00
Cell Volume, Å^3^	4282.4(3)	4847.6(4)	2350.4(6)	2433.34(14)	18494.2(9)
Z	4	4	2	2	16
*D*_*c*_, g·cm^–3^	1.444	1.500	1.435	1.374	1.446
μ, mm^–1^	6.390	0.762	0.780	0.752	0.791
F(000)	1936	2264	1062	1052	8416
Crystal size, mm	0.352 × 0.026 × 0.025	0.240 × 0.210 × 0.150	0.150 × 0.120 × 0.090	0.840 × 0.182 × 0.086	0.315 × 0.257 × 0.046
θ limits,deg	3.597–66.564	1.807–27.000	1.621–25.024	2.04–26.40	1.92–25.04
Reflections collected	15,285	36,522	22,215	42,128	158,586
Independent reflections	5929 [*R*_*int*_ = 0.0328]	5313 [*R*_*int*_ = 0.0398]	8301 [*R*_*int*_ = 0.0759]	4997 [*R*_*int*_ = 0.0552]	16,318 [*R*_*int*_ = 0.0664]
Data/restraints/parameters	5929/364/485	5313/0/321	8301/803/643	4997/26/277	16,318/44/1117
Goodness on fit on F^2^ ^a^	1.057	1.040	1.098	1.100	1.036
*R*_1_ (*I* > 2σ(*I*))^b^	0.0746	0.0273	0.1199	0.0749	0.0928
*wR*_2_ (all data)^c^	0.1883	0.0694	0.3142	0.2196	0.2512
Largest diff. Peak and hole, e Å^–3^	0.911/–0.453	0.442/–0.361	3.322/–2.462	1.671/–0.643	2.974/–1.628

a Goodness on fit on F^2^ = [Σ*w*(*F*_O_^2^ – *F*_C_^2^)^2^/(*N*_*ref*_ –N_*param*_)]^1/2^, where *w* = 1/[σ^2^(*F*_O_^2^) + (*aP*)^2^ + *bP*], where *P* = (*F*_O_^2^ + 2*F*_C_^2^)/3; *N*_ref_ = number of reflections used in the refinement; *N*_param_ = number of refined parameters.

b R_1_ = Σ||*F*_O_|–|*F*_C_||/Σ|*F*_O_|.

c wR_2_ = [Σ*w*(*F*_O_^2^ – *F*_C_^2^)^2^/Σ*w*(*F*_O_^2^)^2^]^1/2^, where *w* = 1/[σ^2^(*F*_O_^2^) + (*aP*)^2^ + *bP*], where *P* = (*F*_O_^2^ + 2*F*_C_^2^)/3. 4Computational studies.

The *cis-anti* (ca) and *cis-syn* (cs) structures of compounds **[2a,b]** ^**+**^ and **[3a,b]** ^**2+**^ were optimized at the B3LYP level of theory^81^ in conjunction with the def2-TZVP basis set,^82^ including Grimme’s D3 dispersion correction^83^ with the Becke-Johnson (BJ) damping scheme.^84^ The optimizations were carried out in the gas phase using the Gaussian 16 (revision A03) quantum chemical software.^85^ Calculations on the electrochemical species derived from **[2a]** ^**+**^ and **[3a]** ^**2+**^ were performed by optimizing their geometries, starting from the *cis-anti* conformation, by adding or removing one electron from the parent complexes. All computations were carried out at the B3LYP level of theory using the PCM model with acetonitrile as the solvent.

### 5.2 Electrochemistry

Cyclic voltammetry (CV) measurements were carried out using a PalmSens4 instrument interfaced to a computer running PSTrace5 electrochemical software. Anhydrous CH_3_CN (Merck) was stored under argon over 3Å molecular sieves. [N^*n*^Bu_4_]­PF_6_ (Fluka, electrochemical grade) was used without further purification. CV measurements were carried out under argon using 0.1 mol dm^–3^ [N^*n*^Bu_4_]­PF_6_ in CH_3_CN as the supporting electrolyte. The working and counter electrodes consisted of a Pt disk sealed in a PEEK tube and a Pt gauze, respectively. A leakless miniature Ag/AgCl/KCl (3.4 mol dm^–3^) electrode (eDAQ) was employed as a reference. The home-built three-electrode cell was predried by heating under vacuum and filled with argon. The Schlenk-type construction of the cell maintained anhydrous and anaerobic conditions. The supporting electrolyte solution, prepared under argon, was introduced into the cell and the CV of the solvent was recorded. The analyte was then added and voltammograms were collected. Under the present experimental conditions, the one-electron oxidation of ferrocene in CH_3_CN occurred at E° = +0.42 V vs Ag/AgCl. IR-SEC measurements were carried out using an OTTLE cell^67^ equipped with CaF_2_ windows, platinum mini-grid working and auxiliary electrodes, and a silver wire pseudoreference electrode. During the microelectrolysis procedures, the electrode potential was controlled by a PalmSens4 instrument interfaced to PSTrace5 software. Argon-saturated CH_3_CN solutions containing [N^*n*^Bu_4_]­PF_6_ 0.1 mol dm^–3^ and the analyzed compound were employed. The *in situ* SEC experiments were performed by recording IR spectra at fixed time intervals during oxidation or reduction while continuously increasing or decreasing the initial working potential at a scan rate of 1.0 or 2.0 mV s^–1^.

### 5.3 Behavior in Aqueous Solutions

#### 5.3.1 Solubility in Water (D_2_O)

A suspension of the selected iron complex (3–5 mg) in a D_2_O solution (0.7 mL) containing 6.98 · 10^–3^ M dimethylsulfone (Me_2_SO_2_) or 3.03 · 10^–3^ M sodium 3-(trimethylsilyl)-2,2,3,3-tetradeuteropropanoate (TMSP-*d*_4_) as internal standard was vigorously stirred at ca. 23 °C for 1 h. The resulting mixture (a saturated solution of the iron complex in D_2_O with standard + undissolved solid) was filtered through Celite, and the filtered solution was transferred into an NMR tube and analyzed by ^1^H NMR spectroscopy (delay time = 10 s; number of scans = 16). The appropriate integral value was assigned to a selected signal of each iron complex (for example, 12 as integral to the signals of the aromatic protons in [**3a**]^2+^). The concentration (=solubility, **S**) was then calculated using the equation: **S** = **C**_**s**_ x **(I**_**s**_ **/n)** (where **C**_**s**_ is the concentration of Me_2_SO_2_, and **I**_**s**_ is the integral of the standard signal at δ = 3.14 ppm for Me_2_SO_2_ and 0.00 ppm for TMSP-*d*_4_, corresponding to **n** protons, 6 or 9 for Me_2_SO_2_ or TMSP-*d*_4_, respectively).

#### 5.3.2 Octanol/Water Partition Coefficient (Log *P*_*ow*_)

Partition coefficients (P_*ow*_; IUPAC: K_D_ partition constant), defined as P_*ow*_ = c_org_/c_aq_, where c_org_ and c_aq_ are the molar concentrations of the selected complex in the organic and aqueous phases, respectively, were determined by the shake-flask method combined with UV–vis measurements. All operations were carried out at 21 ± 1 °C. Deionized water and 1-octanol were mixed and vigorously stirred for 24 h at ambient temperature to allow saturation of both phases. The phases were then separated by centrifugation and used in the subsequent experiments. A solution of the diiron complex (1–2 mg) in water-saturated octanol (or octanol-saturated water in the case of [**3c**]^2+^) was prepared, and its UV–vis spectrum was recorded (A^0^_org_). An aliquot of this solution was transferred into a test tube and an equal volume of the other phase was added (V_org_ = V_aq_ = 1.2 mL). The mixture was vigorously stirred for 30 min at room temperature and then centrifuged (2500 rpm, 5 min). The UV–vis spectrum of the initial phase was recorded (A^f^_org_ or A^f^_aq_) and the partition coefficient was calculated as P_*ow*_ = (A^f^_org_–A_bkg_)/(A^0^_org_–A^f^_org_), when the initial phase was octanol, or P_*ow*_ = (A^0^_aq_–A^f^_aq_)/(A^f^_aq_–A_bkg_), when the initial phase was aqueous. A^0^ and A^f^ are the absorbances of the initial phase before and after equilibration, respectively (subscripts “org” and “aq” refer to the organic or aqueous initial phase), and A_bkg_ is the absorbance of the corresponding solvent blank. The absorbance at a selected wavelength (λ_max_) was used for quantitation. The procedure was repeated three times for each sample using the same stock solution.

#### 5.3.3 Stability in DMSO/Water and DMSO/DMEM

##### 5.3.3.1 NMR Method

The selected compound (**[3a-c]­[CF**_**3**_ **SO**_**3**_ **]**_**2**_, 3–4 mg) was dissolved in DMSO-*d*_6_ and then diluted with D_2_O containing 7.00 · 10^–3^ M dimethylsulfone (Me_2_SO_2_) or 3.03 · 10^–3^ M sodium 3-(trimethylsilyl)-2,2,3,3-tetradeuteropropanoate (TMSP-*d*_4_) as internal standard. The resulting solution (iron complex ≈2 mM; total volume ≈0.8 mL; DMSO content depending on the complex, as reported in Table 3) was filtered through a cotton pad, transferred into an NMR tube, and the ^1^H NMR spectrum was recorded. The solution was then maintained at 37 °C for 72 h, afterward a second ^1^H NMR spectrum was recorded. The residual amount of starting material (relative to the initial spectrum) was determined from the integral ratio with respect to the internal standard. The same procedure was performed using deuterated cell culture medium (DMEM-d) instead of D_2_O. DMEM-d was prepared by dissolving powdered DMEM medium (1000 mg/L glucose and l-glutamine, without sodium bicarbonate or phenol red; D2902 - Merck) in D_2_O (10 mg/mL, according to the manufacturer’s instructions). The solution was supplemented with Me_2_SO_2_ or TMSP-*d*_4_ and NaHCO_3_ (3.7 g/L), then stored under N_2_. NMR spectra were recorded using the following parameters: number of scans = 16; relaxation delay = 10 s.

##### 5.3.3.2 UV–vis Method

Stock solutions of **[2a-b]­[CF**_**3**_ **SO**_**3**_ **]** in DMSO were diluted with H_2_O (*V*_tot_ = 2 mL, 40% v/v of DMSO) to obtain 0.5 · 10^–3^ M solutions. Saturated aqueous solutions of **[3a-c]­[CF**_**3**_ **SO**_**3**_ **]**_**2**_ were filtered through a cotton pad. The five solutions were analyzed by UV–vis spectroscopy (λ = 200–800 nm; scan rate = 750 nm/min; optical path = 1 cm) and then maintained at 37 °C for 72 h. UV–vis spectra were recorded after 1, 3, 6, 24, 48, and 72 h. The percentage of residual starting material after 72 h was calculated as (A^72h^/A^0h^) × 100. The absorbance at λ_max_ = 393 nm was used for quantitation.

### 5.4 Cellular Studies

A549 cells (lung cancer) were cultured in Ham’s F-12 medium, A2780 cells (ovarian cancer) were cultured in RPMI 1640 medium, MRC-5 (normal fibroblast cells) and U87 cells (glioblastoma) were cultured in DMEM; all the media were from Gibco (Life Technologies, USA) and they were supplemented with 10% of fetal bovine serum (Gibco) and 1% penicillin–streptomycin mixture (Gibco). All cell lines were maintained in a humidified atmosphere at 37 °C and 5% CO_2_.

#### 5.4.1 Cytotoxicity

Cells were seeded in 96-well plates at the following densities: 6000 cells per well for A549; 8000 cells per well for A2780 and MRC-5; 9000 cells per well for U87; then, cells were incubated overnight at 37 °C. Then, cells were treated for 48 h with the compounds diluted in fresh culture medium (100 μL/well) at different concentrations (0.01–0.03–0.1–0.3–1–3–10–30–100 μM) from a 10 mM DMSO stock solution. The maximum concentration of DMSO in the medium was 1%. Cisplatin was diluted in sodium chloride solution at 1 mM. Cells were treated with the compounds diluted in fresh culture medium at different concentrations (0.01–0.03–0.1–0.3–1–3–10–30–100 μM) for 48 h (100 μL/well). After 48 h, the compound solutions were replaced with 100 μL of fresh medium containing 0.2 mg/mL resazurin (Acros Organics). After 4 h of incubation at 37 °C, 5% CO_2_, the fluorescence signal of the resorufin product was read (λ_exc_ = 540 nm; λ_em_ = 590 nm) using a Cytation 5 Cell Imaging Multi-Mode Reader (Biotek - Agilent). Fluorescence data were normalized and fitted using GraphPad Prism Software, and IC_50_ values were calculated by nonlinear regression. All experiments were performed in triplicate.

#### 5.4.2 ICP–MS Cellular Uptake Studies

U87 cells were seeded at a density of 0.5 × 10^6^ cells/plate in p100 plates (for whole cell analysis) or 1.5 × 10^6^ cells/plate in p150 plates (for mitochondrial extraction) and incubated at 37 °C, 5% CO_2_. After 2 days, cells were treated with compounds diluted in cell culture medium at a final concentration of 4 μM from a 10 mM stock solution in DMSO. Samples for whole cell analysis were treated with all the compounds, namely **[2a]­[CF**_**3**_ **SO**_**3**_ **]**, **[2b]­[CF**_**3**_ **SO**_**3**_ **]**, **[3a]­[CF**_**3**_ **SO**_**3**_ **]**_**2**_, **[3b]­[CF**_**3**_ **SO**_**3**_ **]**_**2**_, **[3c]­[CF**_**3**_ **SO**_**3**_ **]**_**2**_, and **B–Me**, while samples for mitochondrial fraction analysis were treated only with the most active compounds, namely **[2a]­[CF**_**3**_ **SO**_**3**_ **]** and **[2b]­[CF**_**3**_ **SO**_**3**_ **]**. After 24 h, cells were collected, counted, and 5·10^6^ cells were centrifuged and dried for whole cell analysis, while 20 × 10^6^ cells were collected for mitochondrial isolation using a mitochondrial extraction kit (Thermo Fisher Scientific). ICP–MS samples were prepared as follows: samples were digested using 70% nitric acid (0.5 mL, 60 °C, overnight) and then further diluted 1:30 (100 μL in 2.9 mL MQ water) and analyzed using ICP–MS. All ICP–MS measurements were performed on an Agilent 7900 Quadrupole ICP–MS operated in collision/reaction mode with He as collision gas, located at the Institut de Physique du Globe de Paris (France). The monitored isotope was ^56^Fe. The amount of metal detected in the cell samples was converted from ppb to ng of metal. Metal accumulation in the mitochondria was calculated as a percentage of the metal present in the whole cell samples.

#### 5.4.3 Mitochondrial Respiration Studies

U87 cells were seeded at a density of 15,000 cells/well in a Seahorse XF Cell 96-well culture microplate using 80 μL of DMEM supplemented with 10% FBS and incubated at 37 °C with 5% CO_2_. The following day, 80 μL of culture medium containing compounds **[2a]­[CF**_**3**_ **SO**_**3**_ **]**, **[2b]­[CF**_**3**_ **SO**_**3**_ **]**, **[3a]­[CF**_**3**_ **SO**_**3**_ **]**_**2**_, or cisplatin as a control, were added to the wells already containing 80 μL of culture medium, in order to obtain a final concentration corresponding to their IC_50_ (calculated on 48 h cytotoxicity assay). After 24 h of treatment, the compounds were removed and replaced with Seahorse XF assay medium, and the plate was incubated at 37 °C for 1 h in a non-CO_2_ incubator. The Mito stress test was run in an Agilent Seahorse XFe96 instrument at 37 °C using multiple inhibitors, i.e., ATP synthase inhibitor (oligomycin, 1 μM), proton gradient, mitochondrial membrane potential collapsing agent (FCCP, 1 μM), and mitochondrial respiratory complex I and III inhibitors (rotenone, 1 μM and antimycin A, 1 μM, respectively). At the end of the run, the cells were fixed using a 4% p-formaldehyde solution and stained with Hoechst 33,342. The wells were imaged in a Cytation 5 Cell Imaging Multimode Reader, BioTek using a 10× objective lens. Finally, the number of cells from each image was calculated using Gen5 software and used to normalize the Mito stress test results according to the number of cells present in each well.

#### 5.4.4 Mitochondrial Membrane Potential Assay

The JC-1-Mitochondrial Membrane Potential Assay Kit (Abcam, Catalog no. ab113850) was used to evaluate mitochondrial depolarization in A549 and U87 cells. Briefly, A549 and U87 cells were seeded at a density of 60,000 and 40,000 cells/well, respectively, in 8-well plates for confocal imaging (ibidi GmbH) and incubated overnight at 37 °C, 5% CO_2_. The next day, the culture medium was replaced with fresh culture medium containing **[2a]­[CF**_**3**_ **SO**_**3**_ **]**, **[2b]­[CF**_**3**_ **SO**_**3**_ **]**, and **[3a]­[CF**_**3**_ **SO**_**3**_ **]**_**2**_, at concentrations corresponding to their IC_50_ values determined after 48 h exposure, and the dishes were incubated at 37 °C, 5% CO_2_. After 22 h, cells were incubated with 20 μM JC-1 dye diluted in dilution buffer (provided with the kit) containing the compounds at the same concentration as the initial incubation at 37 °C for 20 min in the dark. Cells were then washed with DMEM without phenol red and incubated for an additional 2 h with compounds diluted in DMEM without phenol red and then directly imaged using a confocal laser scanning microscope (Leica Microsystems, Nanterre, France) equipped with x63/1.40 plan apochromat oil objective. The excitation/emission wavelengths were 488/505–545 nm (green monomers) and 488/580–650 nm (red aggregates). Laser intensities were kept as low as possible to avoid any phototoxicity (1%). Fluorescence intensities were calculated and single-channel images were merged using the ImageJ software.

#### 5.4.5 Intracellular ROS Detection

A549 and U87 cells were seeded at a density of 60,000 and 40,000 cells/well, respectively, in 8-well plates for confocal imaging (ibidi GmbH) and incubated overnight at 37 °C in a 5% CO_2_ atmosphere. The following day, the culture medium was replaced with fresh culture medium containing **[2a]­[CF**_**3**_ **SO**_**3**_ **]**, **[2b]­[CF**_**3**_ **SO**_**3**_ **]**, or **[3a]­[CF**_**3**_ **SO**_**3**_ **]**_**2**_ at concentrations corresponding to their IC_50_ values determined after 48 h of exposure, and the dishes were incubated for a further 24 h. Cells were then incubated with 20 μM 2′,7′-dichlorodihydrofluorescein diacetate (H_2_DCFDA, Thermo Fisher Scientific) in culture medium for 30 min at 37 °C in the dark. Upon cleavage of the acetate groups by intracellular esterases and oxidation, nonfluorescent H_2_DCFDA is converted to highly fluorescent 2′,7′ dichlorofluorescein (DCF). Cells were washed with phenol red–free DMEM and then imaged using a confocal laser scanning microscope (Leica Microsystems, Nanterre, France) equipped with x63/1.40 plan apochromat oil objective. The excitation/emission wavelengths to detect DCF were 488/500–550 nm. Laser intensity was kept as low as possible to minimize phototoxicity (0.5%). Single-channel images were merged using the ImageJ software.

### 5.5 Interaction with Biomolecules

Stock solutions of [**2a**]^+^ (3.22 mM) and [**3a**]^2+^ (2.38 mM) in DMSO were prepared by weight. The working solutions (3.19 × 10^–5^ M for [**2a**]^+^, 2.36 × 10^–5^ M for [**3a**]^2+^) were obtained by dilution in the buffer (ultrapure water containing NaCl 0.1 M and sodium cacodylate 0.01 M, pH = 7.0). Absorbance measurements were carried out using a Shimadzu UV-2450 spectrophotometer. For the stability studies of [**2a**]^+^ and [**3a**]^2+^ in the buffer, UV–vis spectra (200–800 nm range) were recorded every 10 min over a total period of 90 min. In the DNA binding experiments, DNA from calf thymus (Merck) was used. DNA solutions were prepared according to a previously described procedure, and C_DNA_ represents the molar concentration expressed in base pairs (ε_260 nm_ = 13,200 M^–1^ cm^–1^).^86^ Ethidium bromide (EB, Merck) stock solutions were prepared in buffer by weight and stored at 4 °C; the molar concentration (C_EB_) was checked spectrophotometrically (ε_480 nm_ = 5740 M^–1^ cm^–1^).^87^ The starting mixture was prepared directly in the spectrofluorometer cuvette by adding 500 μL of buffer, 50 μL of DNA solution (1.67 mM) and 60 μL of EB solution (6.04 × 10^–4^ M in buffer). The initial concentrations in the cuvette were C_DNA_ = 1.37 × 10^–4^ M and C_EB_ = 5.94 × 10^–5^ M; under these conditions, the polynucleotide is saturated with EB.^87^ Fluorescence spectra were recorded (λ_ex_ = 520 nm, λ_em_ = 530–650 nm, slits width = 4 nm, acquisition speed = 200 nm/min) after each incremental addition of [**2a**]^+^ or [**3a**]^2+^. A control experiment was carried out by adding a solution prepared from 10 μL DMSO and 1 mL buffer to reproduce the experimental conditions (final DMSO volume <10%) and verify that this limited variation in DMSO content did not affect the fluorescence signal.

Bovine serum albumin (BSA) was obtained from Merck (crystallized and lyophilized powder, ≥98% agarose gel electrophoresis, ≤0.005% fatty acids). Concentrated solutions (ca. 0.1 mM) were prepared by weight, and the exact concentration was assessed spectrophotometrically (ε_278 nm_ = 45,000 M^–1^ cm^–1^).^88^ BSA binding was studied by direct titrations, where increasing aliquots of the metal complex were added directly to the BSA solution (C_BSA_ = 8.61 × 10^–7^ M for [**2a**]^+^, 2.00 × 10^–6^ M for [**3a**]^2+^) in buffer in the spectrofluorimetric cuvette. Fluorescence emission spectra were recorded (λ_exc_ = 280 nm, λ_em_ = 300–400 nm) upon incremental addition of the metal complex. The binding isotherm was extrapolated from the decrease in fluorescence intensity at 345 nm (Figure S61). The binding isotherm data for **[2a]** ^**+**^ and **[3a]** ^**2+**^ were first analyzed using a Stern–Volmer plot; the slope of the plot is higher than 1000 (>2 × 10^5^ in both cases), consistent with the formation of a BSA-small molecule adduct.^63^ The spectral changes were then fitted using the HypSpec software (www.hyperquad.co.uk/): they were found to agree with a simple 1:1 binding model, and the corresponding binding constant (logβ) was obtained from the fitting procedure. The initial estimate required by the software was varied to verify the robustness of the log β value.

## Supplementary Material





## Notes

Cartesian coordinates of the DFT-optimized structures are collected in a separate.xyz file (TXT)
IR and NMR spectra and data; crystallographic, computational and electrochemical details; IC_50_ plots and cellular uptake details; DNA and BSA binding studies (PDF) (PDF)
