# Supplementary material for: Diiron(I) Complexes with Promising Anticancer Properties Obtained by CO-To-Isocyanide Substitution of [Fe2Cp2(CO)4]
Source: Inorg Chem. 2026 Sep 24;65(39):23284–303. doi: 10.1021/acs.inorgchem.6c03964 (PMC13644309; doi:10.1021/acs.inorgchem.6c03964)

Supporting Information

Diiron(I) Complexes with Promising Anticancer Properties Obtained by CO-to-Isocyanide Substitution of $[Fe_2Cp_2(CO)_4]$

Sara Benetti,[a] Giulia Salluce,[b] Axia Marlin,[b] Tommaso Nottoli,[a] Giulio Bresciani,[a] Filippo Lipparini,[a] Tiziana Funaioli,[a] Tarita Biver,[a] Pierre Mesdom,[b] Stefano Zacchini,[c] Massimo Guelfi,[a,d] Lorenzo Biancalana,[a] Gilles Gasser,[b,*] Fabio Marchetti [a,*]

[a] University of Pisa, Department of Chemistry and Industrial Chemistry, Via Moruzzi 13, 56124 Pisa, Italy.

[b] PSL University, Chimie ParisTech, CNRS, Institute of Chemistry for Life and Health Sciences, 11 Rue Pierre et Marie Curie, 75005 Paris, France.

[c] University of Bologna, Department of Industrial Chemistry "Toso Montanari", Via Gobetti 85, 40129 Bologna, Italy.

[d] The Centre for Instrument Sharing of the University of Pisa (CISUP), Lungarno Pacinotti 43/44, 56126 Pisa, Italy.

Corresponding Authors

*E-mail addresses: gilles.gasser@chimieparistech.psl.eu; fabio.marchetti@unipi.it

**Table of contents**	*Pages*
Comparative IR and NMR data (**Table S1**)	S3
IR spectra (**Figures S1-S11**)	S4-S7
^{1}H, ^{13}C and ^{1}H NOESY NMR spectra (**Figures S12-S25**)	S8-S15
Symmetry and isomerism (**Schemes S1-S2** and **Figures S26-S27**)	S16-S17
Computational studies (**Figures S28-S30** and **Tables S2-S7**)	S18-S24
Single-crystal X-ray crystallography (**Figures S31-S34**)	S25-S26
Electrochemistry (**Figures S35-S40**)	S27-S31
NMR and UV-Vis stability studies in aqueous media (**Table S8** and **Figures S41-S54**)	S32-S42
Cellular studies (**Figures S55-S59** and **Table S9**)	S43-S45
Biomolecule binding studies (**Figures S60-S61**)	S46

Table S1. Comparative IR and NMR data. NMR signals of the second isomer (when present) are reported in italics.

Compound	IR: ũ/cm^{-1}			^{1}H NMR: δ/ppm		^{13}C NMR: δ/ppm				
	**t-CNR**	**μ-CNR**	**μ-CNMeR**	**$C_5\underline{H}_5$**	**N$\underline{Me}$**	**μ-$\underline{C}$NR**	**μ-$\underline{C}$NMeR**	**t-$\underline{C}$NR**	**$\underline{C}_5H_5$**	**N$\underline{Me}$**
**[2a-CO]$^+$** [a]	/	/	1530	5.48<br>4.83	4.44	/	327.8	/	91.3<br>91.1	56.3
**[2a]$^+$** [b,e]	2099	1780	1499	5.18<br>4.42	4.61	229.4	338.5	174.9<br>173.9	89.0<br>86.7	54.7
**Δ(CO → CNR)**	-	-	- 31	- 0.30<br>- 0.41	+ 0.17	-	+ 10.7	-	- 2.3<br>- 4.4	- 1.6
**[2b-CO]$^+$** [a]	/	/	1567	5.36<br>5.27	4.06	/	316.4	/	90.3<br>90.1	46.8
**[2b]$^+$** [b,d]	2137	1747,<br>1718	1531	4.94<br>4.90	4.09	230.1	331.6	163.4<br>162.4	87.2<br>87.1	44.6
**Δ(CO → CNR)**	-	-	- 36	- 0.42<br>- 0.37	+ 0.03	-	+ 15.2	-	-3.1<br>-3.0	- 2.2
**[3a]$^{2+}$** [c,d,h]	2132	/	1527	5.48<br>*6.18*<br>*4.82*	4.74<br>*5.00*	/	327.0<br>*327.6*	164.8	92.2<br>*93.2*<br>*91.2*	56.3<br>*57.7*
**[3b]$^{2+}$** [c,f,h]	2170	/	1558	5.32<br>*5.33*<br>*5.28*	4.09<br>*4.07*	/	317.6	150.6<br>*149.9*	89.5<br>*89.8*<br>*89.2*	44.9<br>*45.1*
**[3c]$^{2+}$** [c,g]	2209	/	1596	5.18	4.05	/	318.7	142.9	89.9	53.3

[a] $[Fe_2Cp_2(CO)_3\{CN(Me)(R)\}]CF_3SO_3$: R = Xyl, **[2a^{CO}][CF$_3$SO$_3$]**; R = Cy, **[2b^{CO}][CF$_3$SO$_3$]**. Taken from the literature.[1] [b]IR spectra recorded in CH_2Cl_2 solutions, [c]IR spectra recorded in CH_3CN solutions, [d]NMR spectra recorded in $(CD_3)_2CO$ solutions. [e] NMR spectra recorded in $CDCl_3$ solutions. [f] NMR spectra recorded in CD_3NO_2 solutions. [g]NMR spectra recorded in CD_3CN solutions. [h] NMR signals attributed to the *cis-anti* isomer.

[1] (a) Biancalana, L.; De Franco, M.; Ciancaleoni, G.; Zacchini, S.; Pampaloni, G.; Gandin, V.; Marchetti, F. Easily available, amphiphilic diiron cyclopentadienyl complexes exhibit *in vitro* anticancer activity in 2D and 3D human cancer cells through redox modulation triggered by CO release, *Chem. Eur. J.*, **2021**, *27*, 10169–10185. (b) Agonigi, G.; Bortoluzzi, M.; Marchetti, F.; Pampaloni, G.; Zacchini, S.; Zanotti, V. Regioselective nucleophilic additions to diiron carbonyl complexes containing a bridging aminocarbyne ligand: a synthetic, crystallographic and DFT study, *Eur. J. Inorg. Chem.*, **2018**, 960–971.

Figure S1. Comparative IR spectra of Fp_2 and **1a-c** recorded in toluene or xylene solutions.

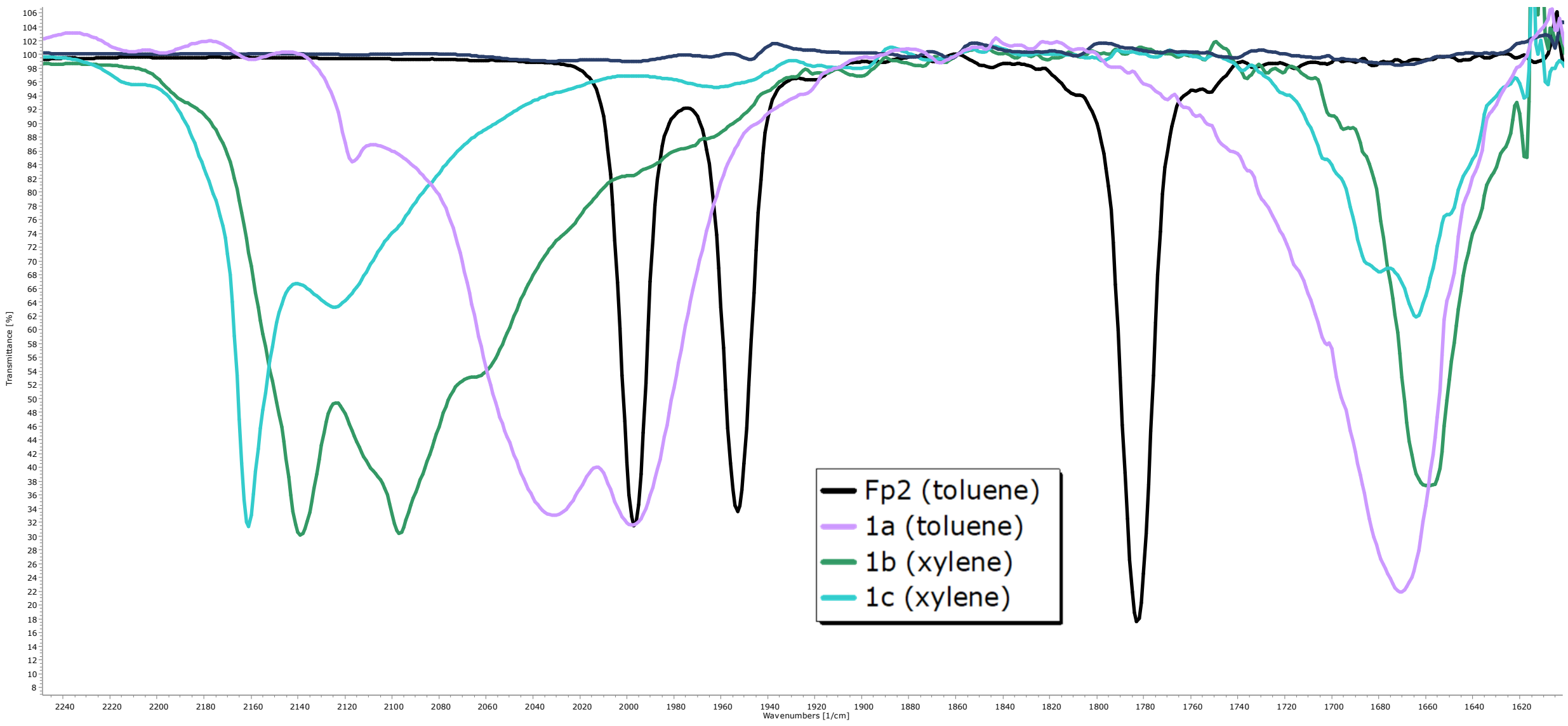

Figure S2. IR spectrum of **[2a][CF$_3$SO$_3$]** in CH_2Cl_2 solution

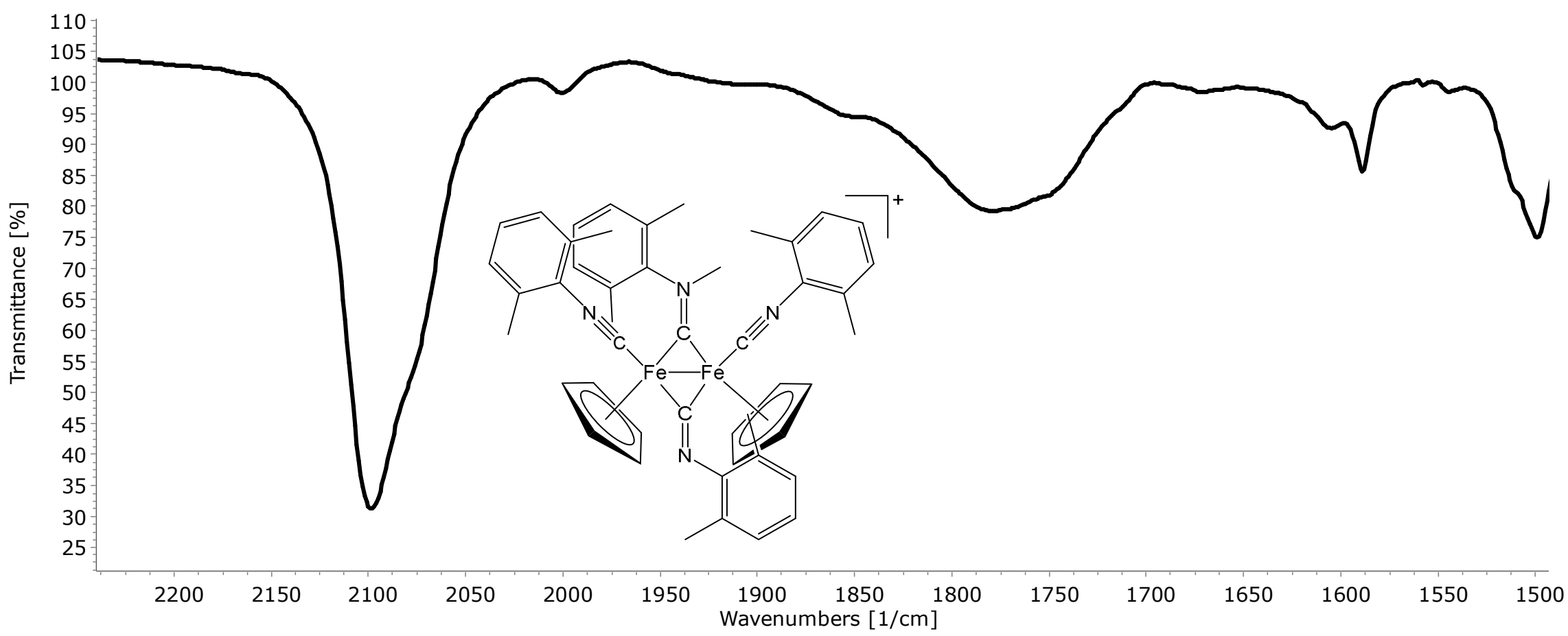

Figure S3. IR spectrum of **[2b][CF$_3$SO$_3$]** in CH_2Cl_2 solution

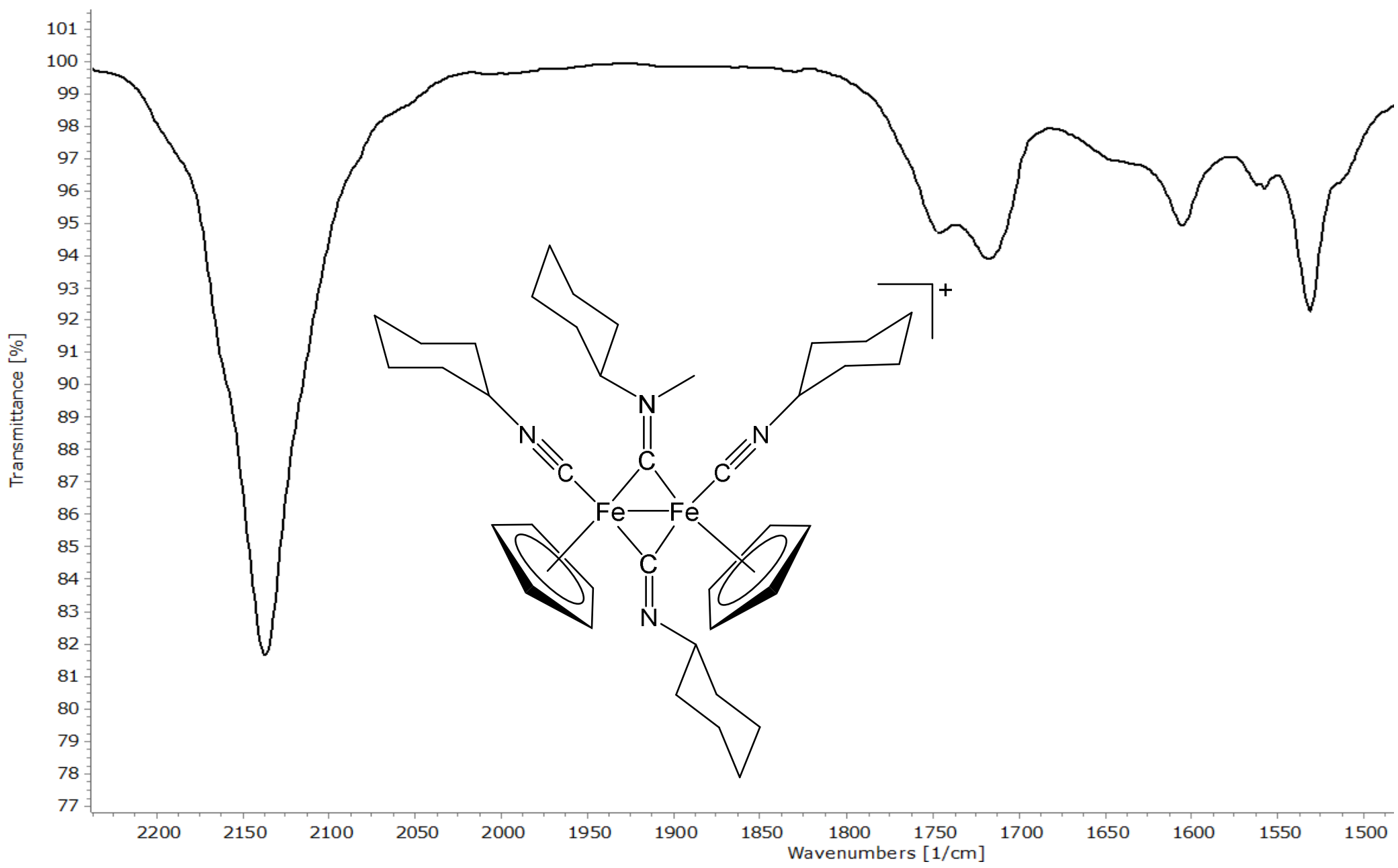

Figure S4. IR spectrum of **[3a][CF_3SO_3]$_2$** in CH_3CN solution

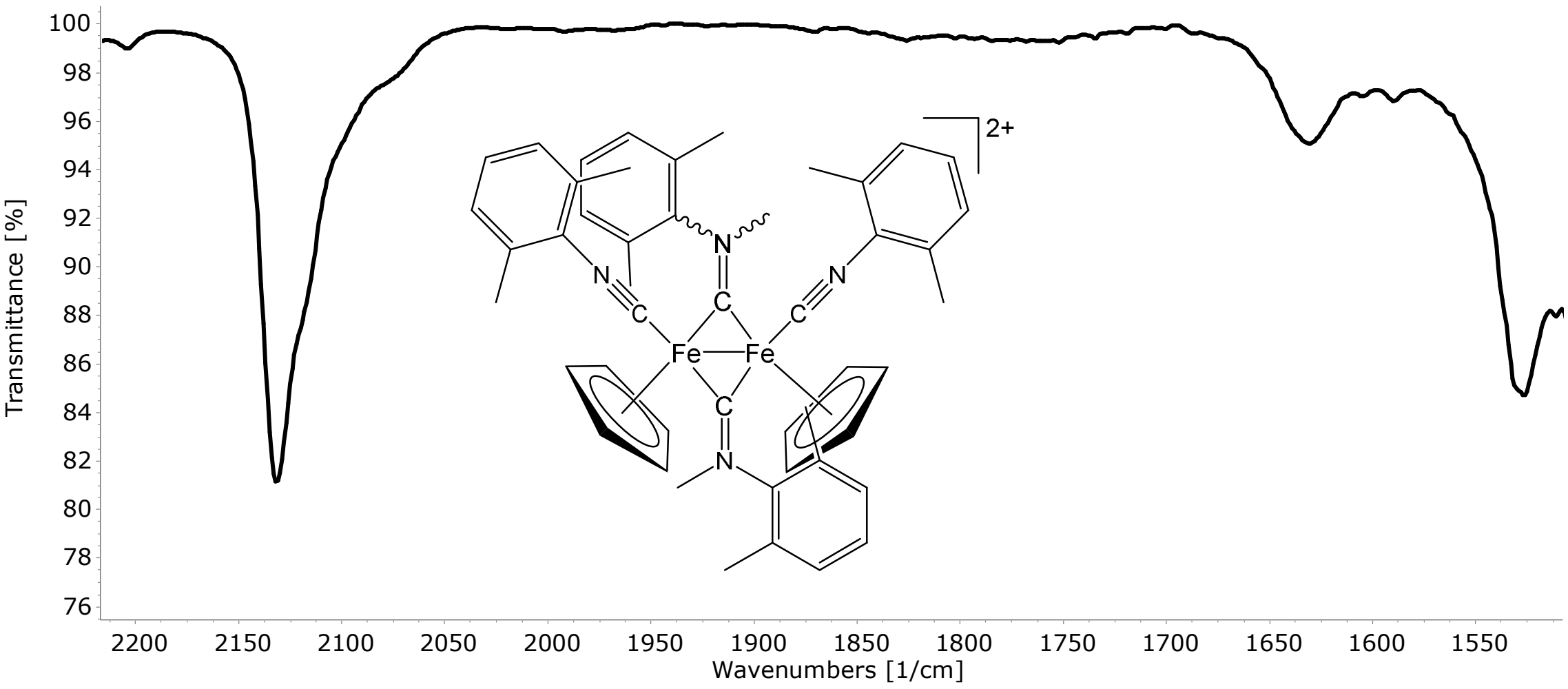

Figure S5. IR spectrum of **[3b][CF_3SO_3]$_2$** in CH_3CN solution

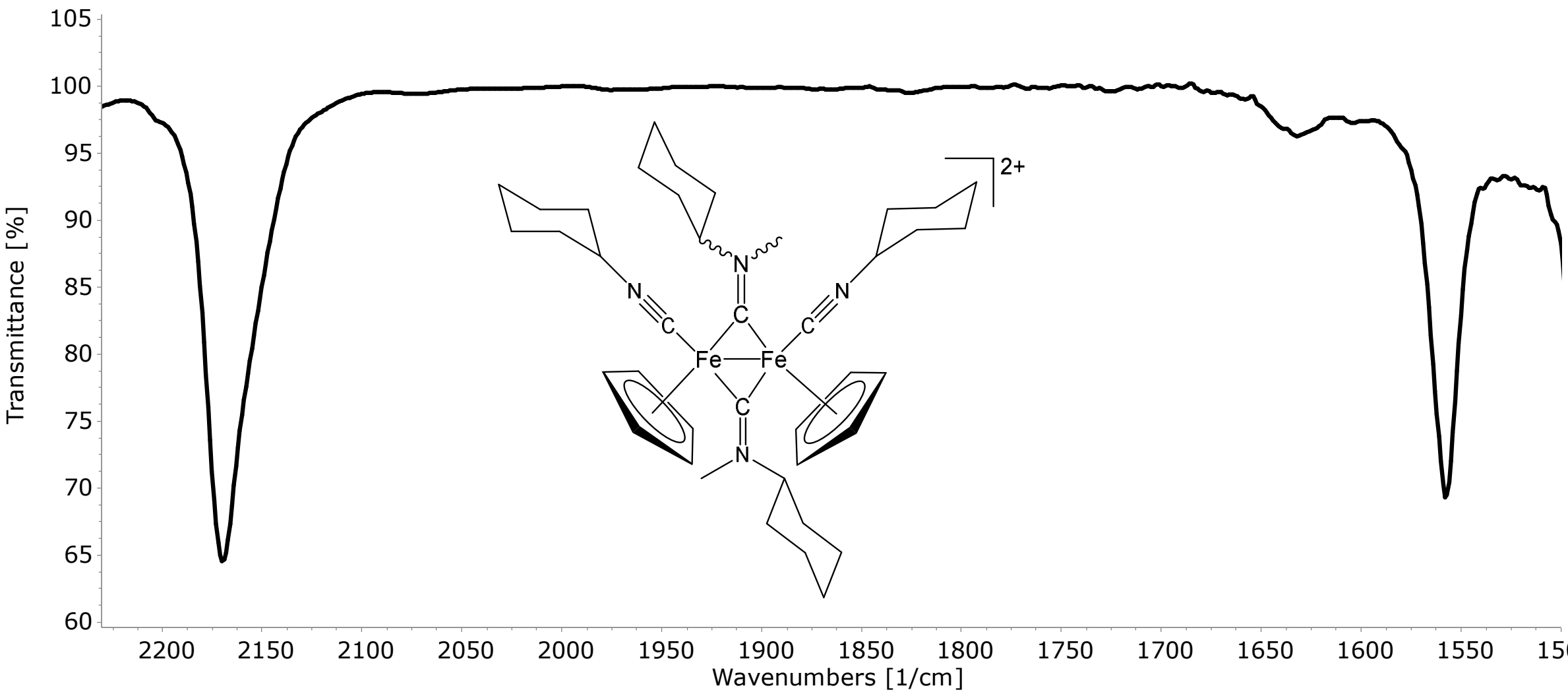

Figure S6. IR spectrum (CH_3CN solution) of **[3c][CF_3SO_3]$_2$** in CH_3CN solution

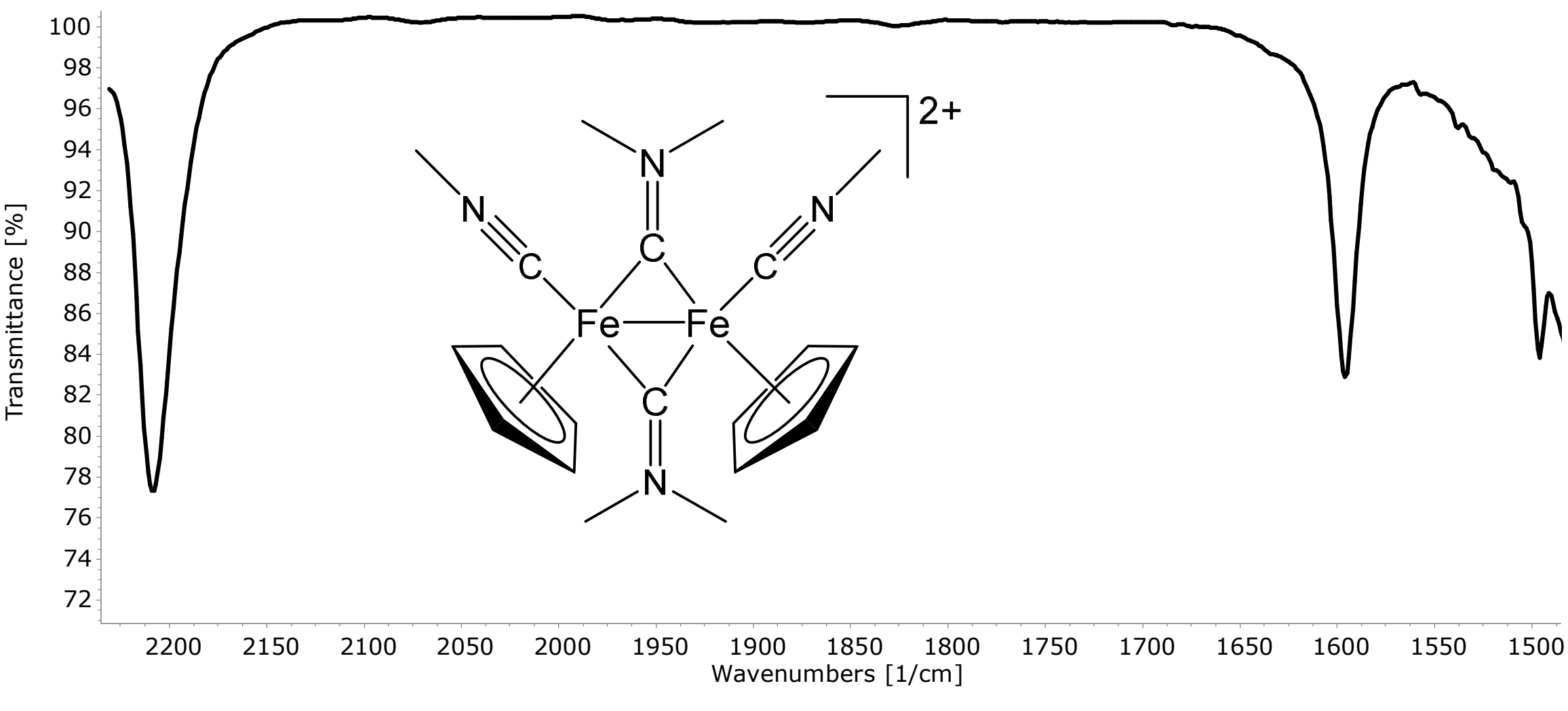

Figure S7. IR spectrum of **[2a][CF$_3$SO$_3$]** in the solid state

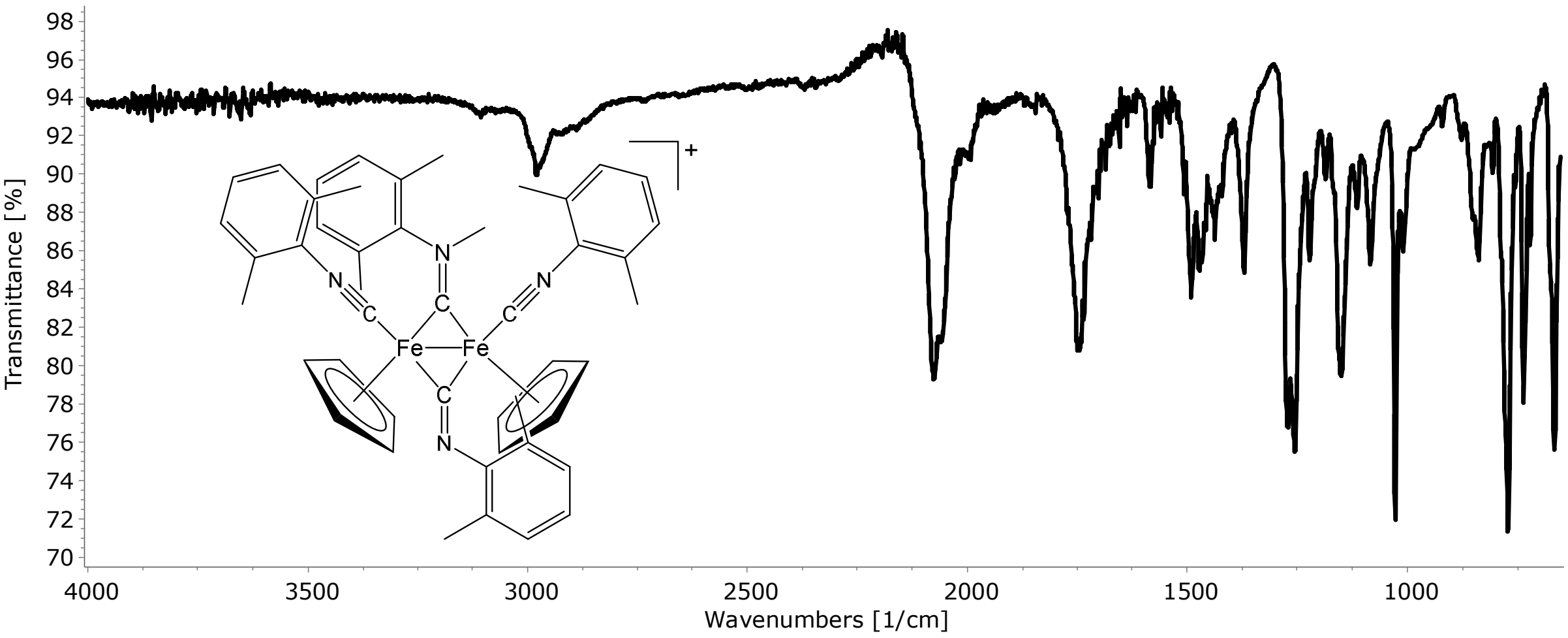

Figure S8. IR spectrum of **[2b][CF$_3$SO$_3$]** in the solid state

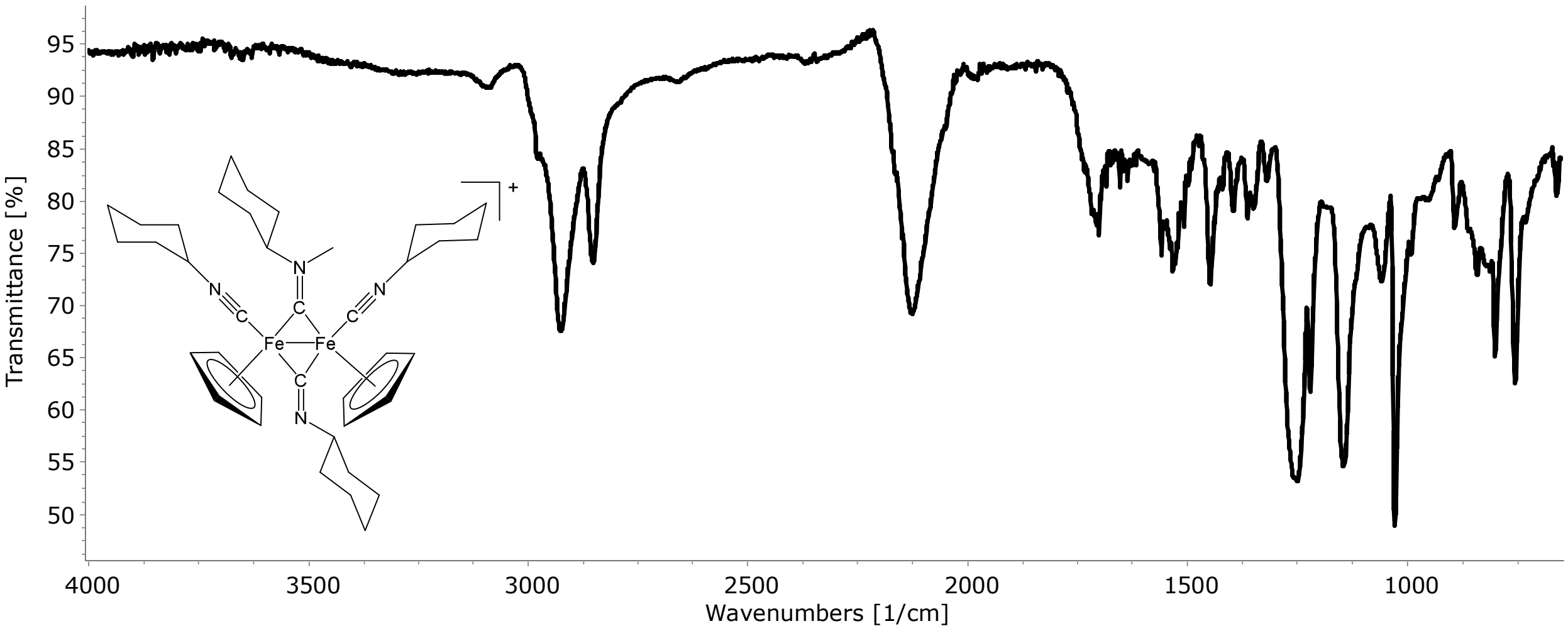

Figure S9. IR spectrum of **[3a][CF$_3$SO$_3$]$_2$** in the solid state

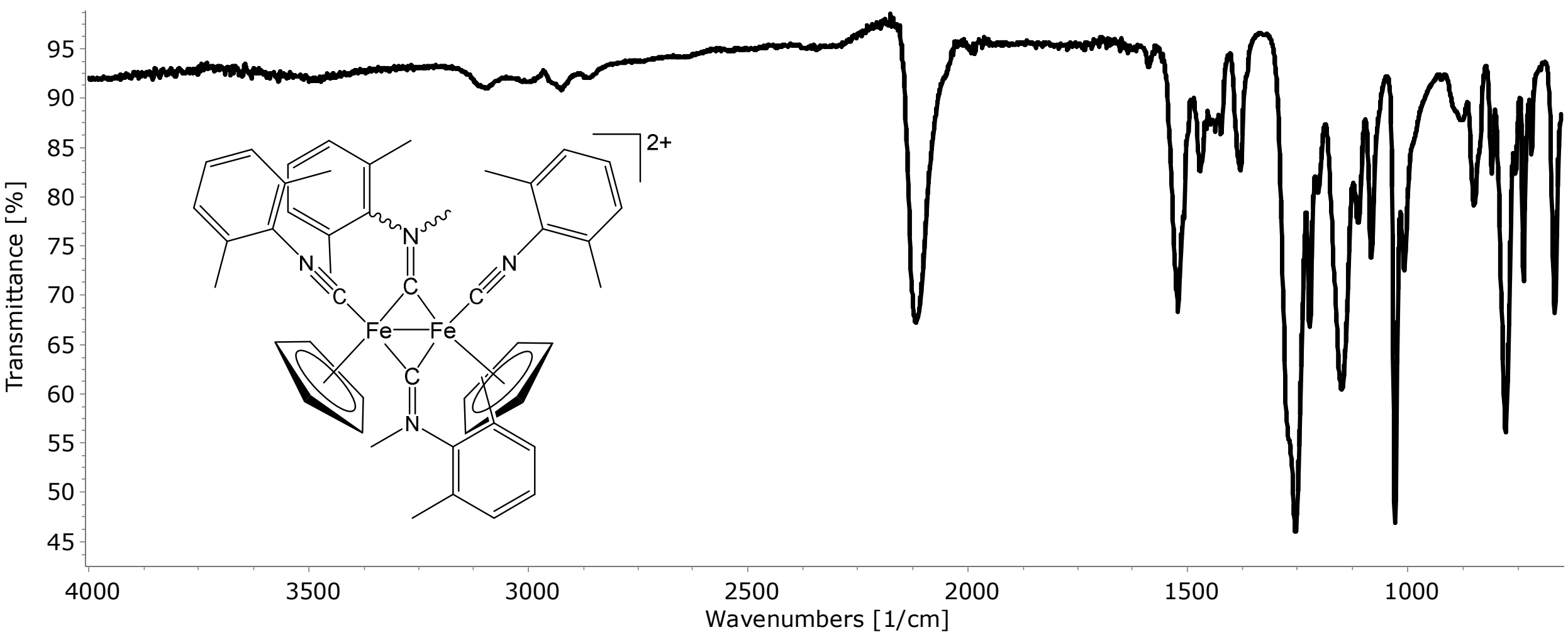

Figure S10. IR spectrum of **[3b][CF_3SO_3]$_2$** in the solid state

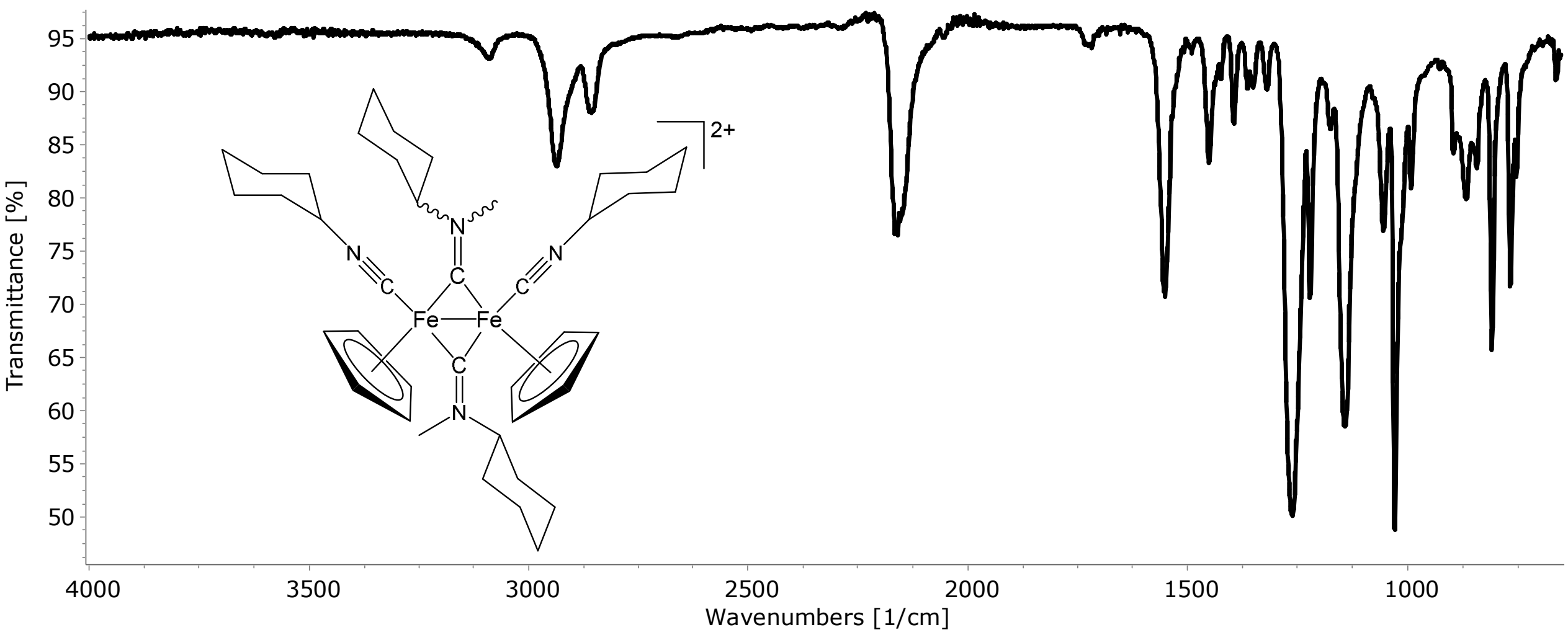

Figure S11. IR spectrum of **[3c][CF_3SO_3]$_2$** in the solid state

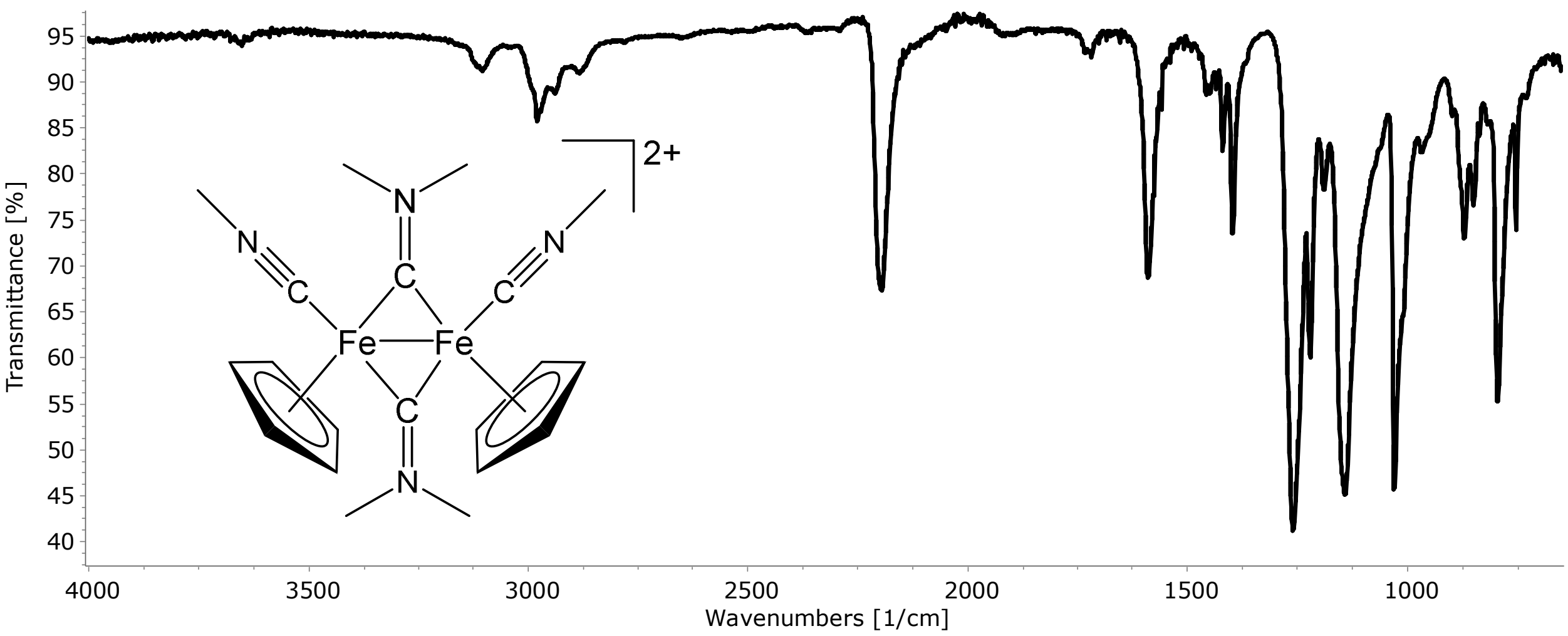

Figure S12. ^{1}H NMR spectrum (401 MHz, $CDCl_3$) of **[2a][CF_3SO_3]**

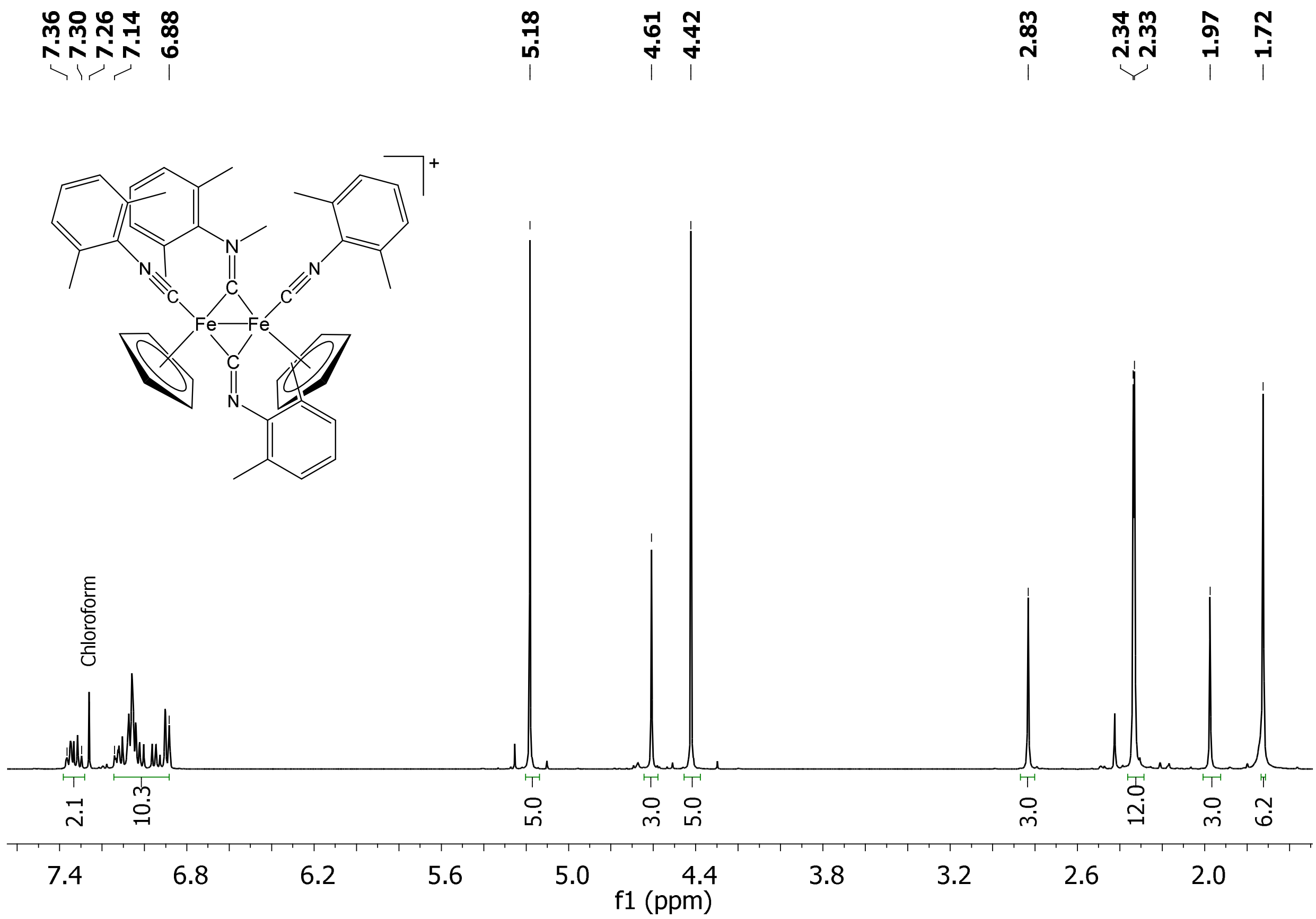

Figure S13. $^{13}C\{^{1}H\}$ NMR spectrum (401 MHz, $CDCl_3$) of **[2a][CF_3SO_3]**

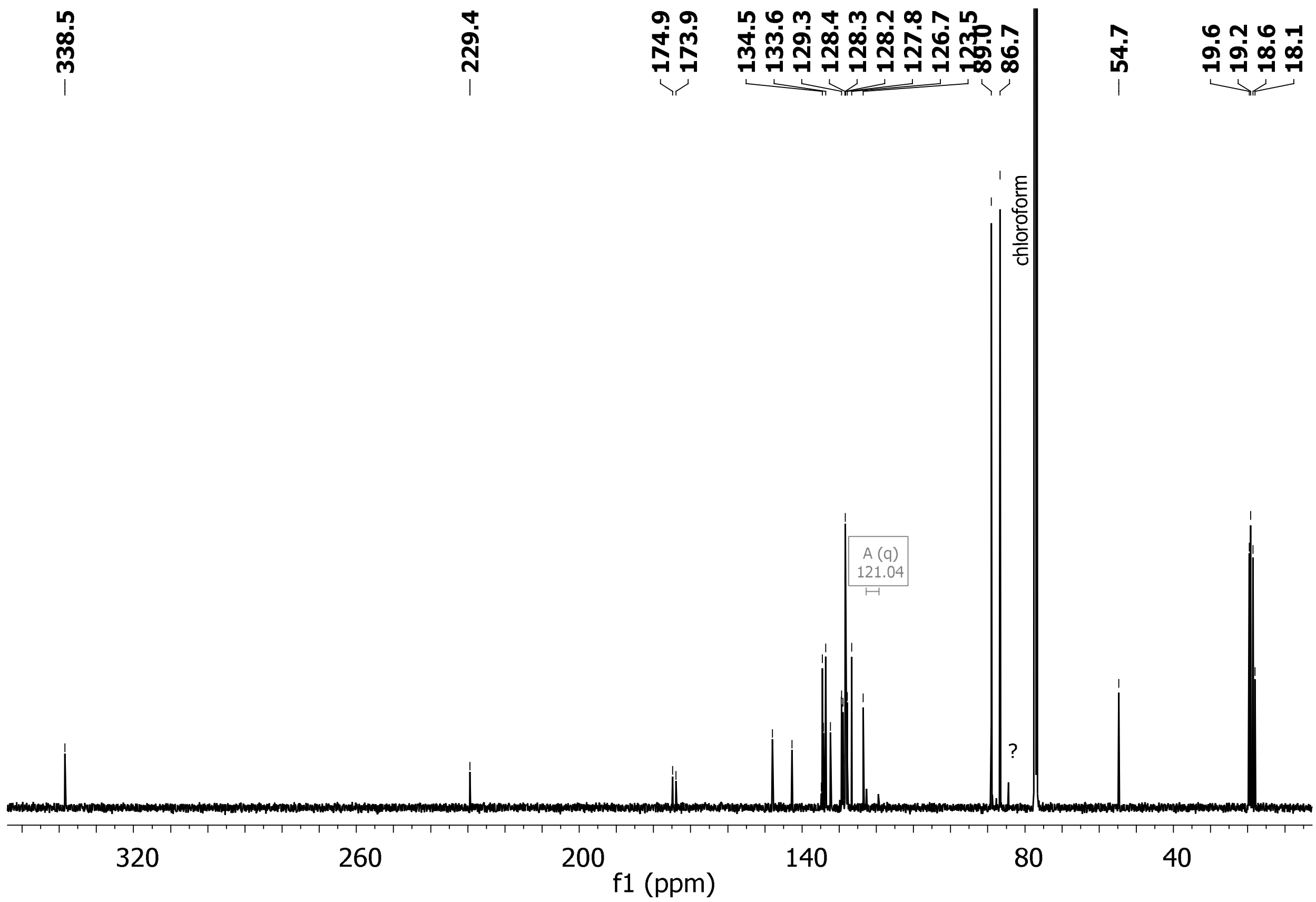

Figure S14. Blue trace: ^{1}H NMR spectrum (401 MHz, $CDCl_3$) of **[2a][CF_3SO_3]**. Green trace: ^{1}H NOESY spectrum with selective irradiation at 5.18 ppm (Cp resonance of the *cis-anti* isomer). Red trace: ^{1}H NOESY spectrum with selective irradiation at 4.42 ppm (Cp resonance of the *cis-anti* isomer).

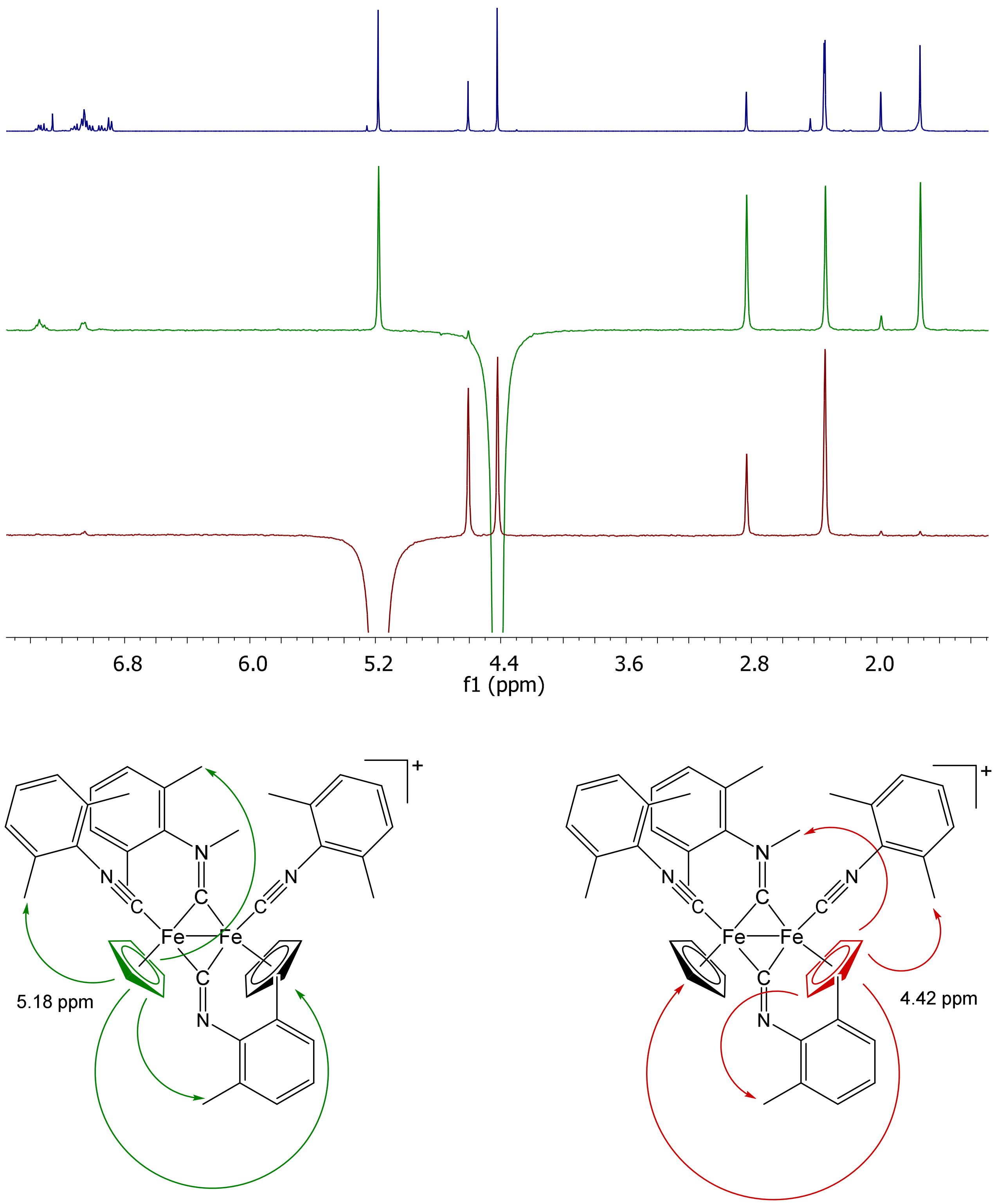

Figure S15. ^{1}H NMR spectrum (401 MHz, acetone-d_6) of **[2b][CF_3SO_3]**

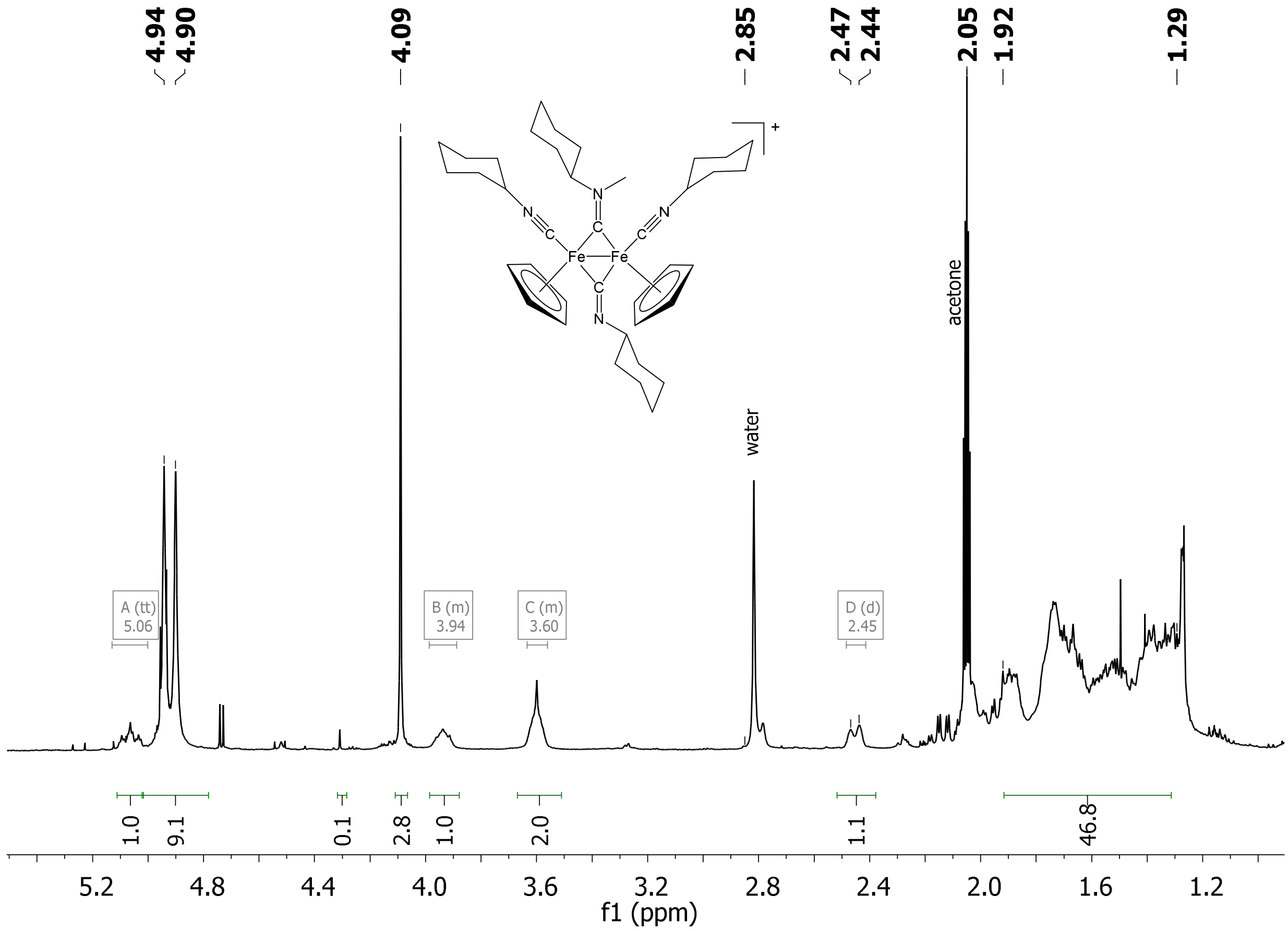

Figure S16. $^{13}C\{^{1}H\}$ NMR spectrum (401 MHz, acetone-d_6) of **[2b][CF_3SO_3]**

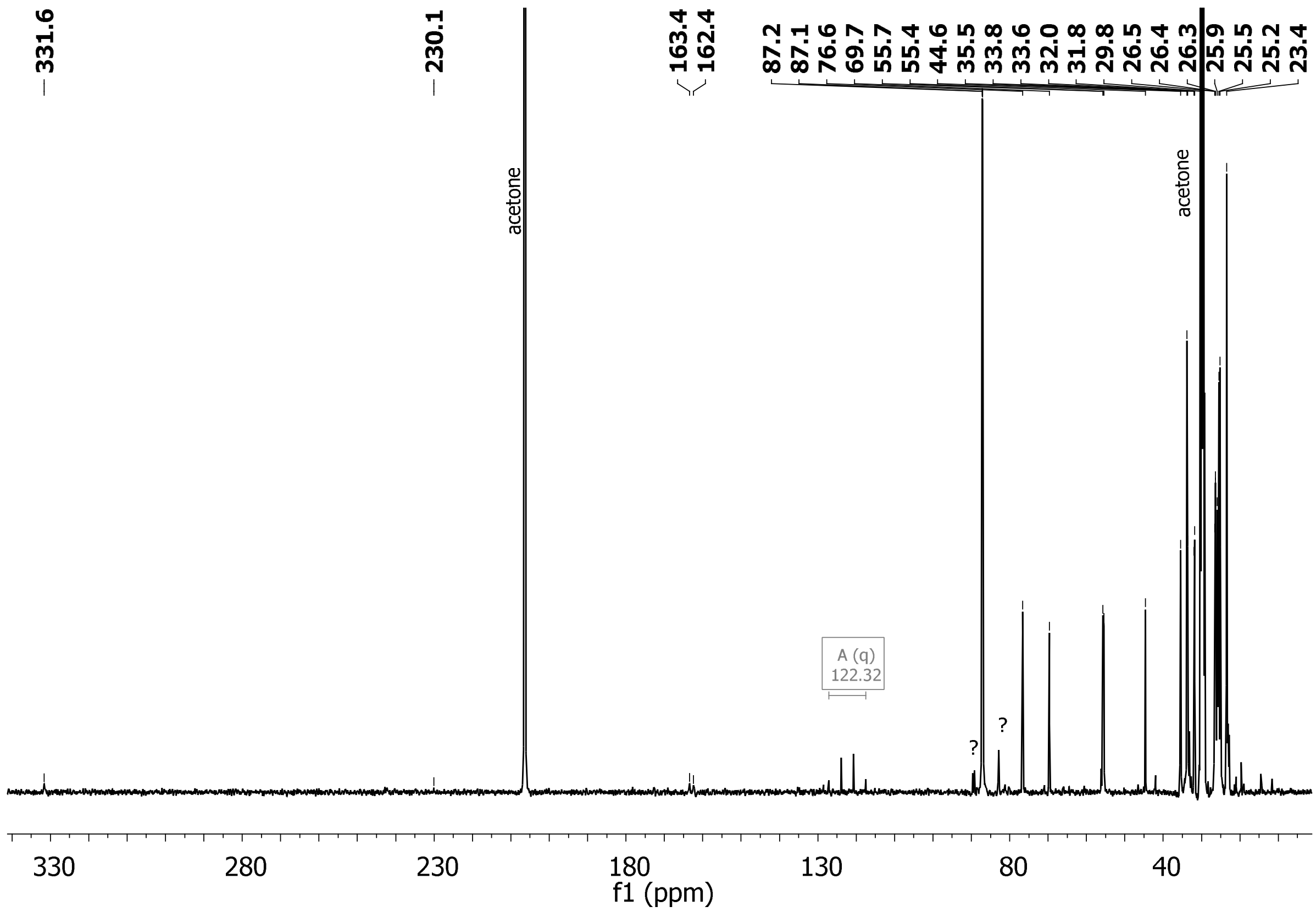

Figure S17. ^{1}H NMR spectrum (401 MHz, acetone-d_6) of **[3a][CF$_3$SO$_3$]$_2$**. Integrals refer to the sum of the two isomers

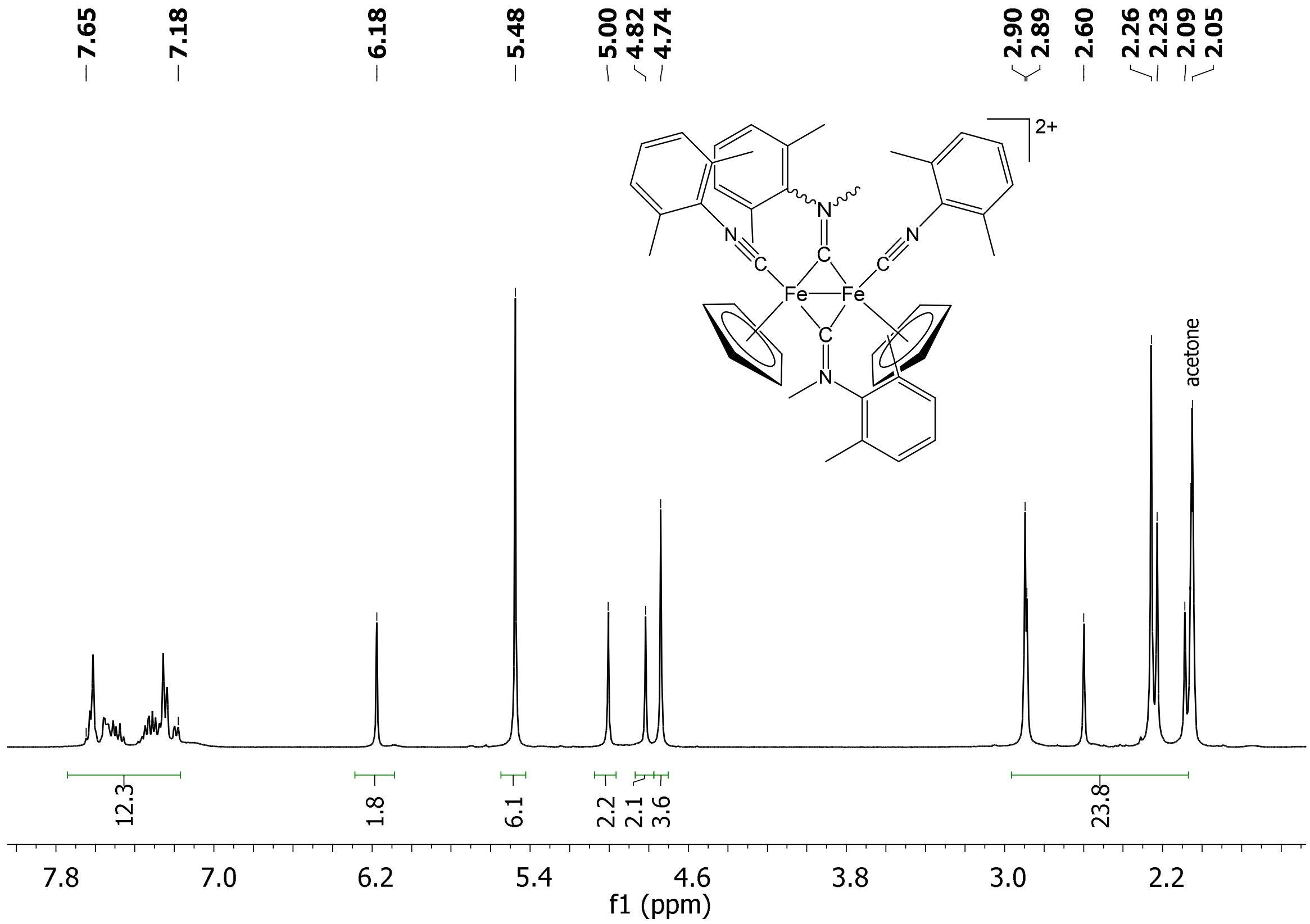

Figure S18. $^{13}C\{^{1}H\}$ NMR spectrum (401 MHz, acetone-d_6) of **[3a][CF$_3$SO$_3$]$_2$**

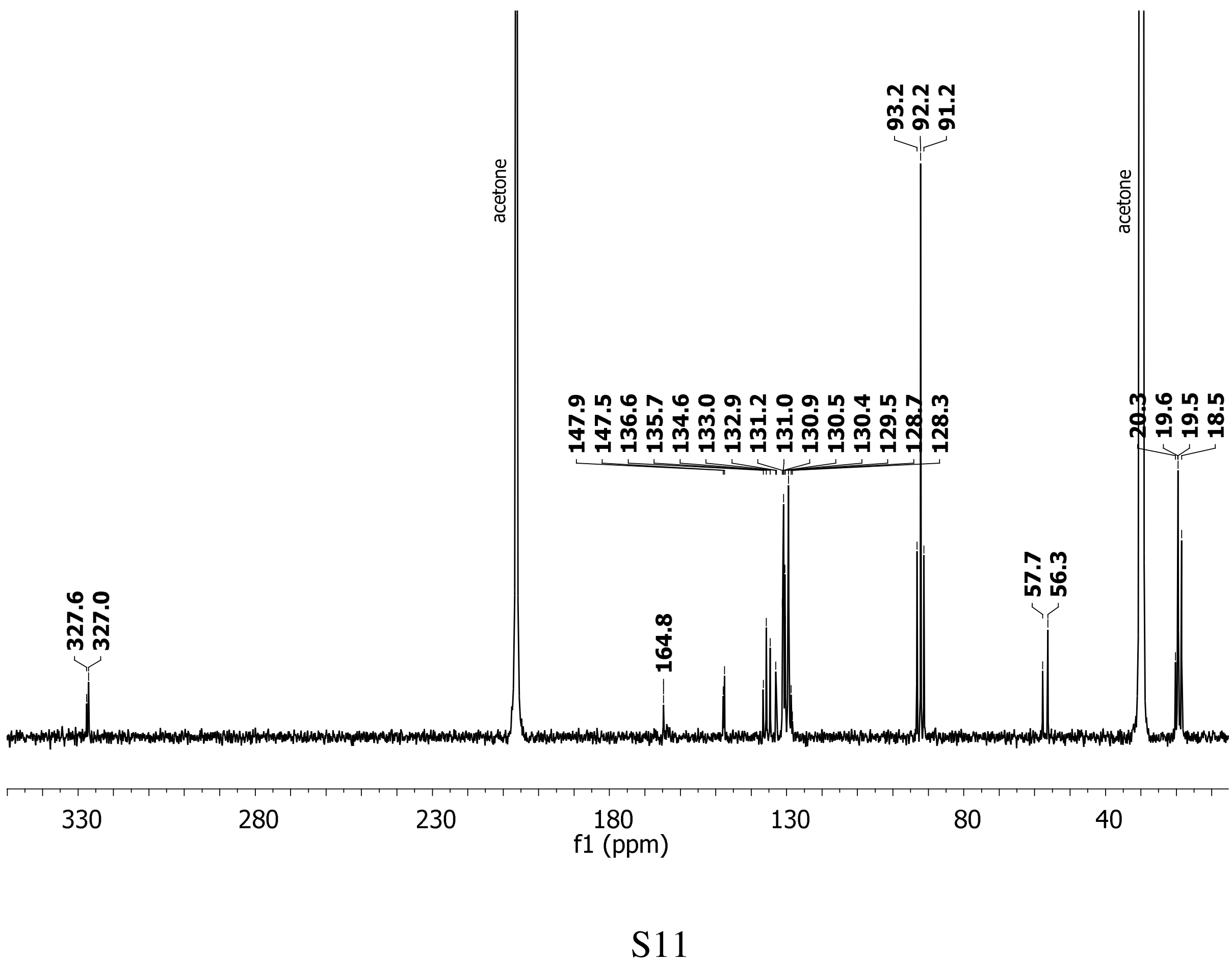

Figure S19. Blue trace: ^{1}H NMR spectrum (401 MHz, acetone-d6) of **[3a][CF$_3$SO$_3$]$_2$**. Red trace: ^{1}H NOESY spectrum with selective irradiation at 5.48 ppm (Cp resonance of the *cis-anti* isomer).

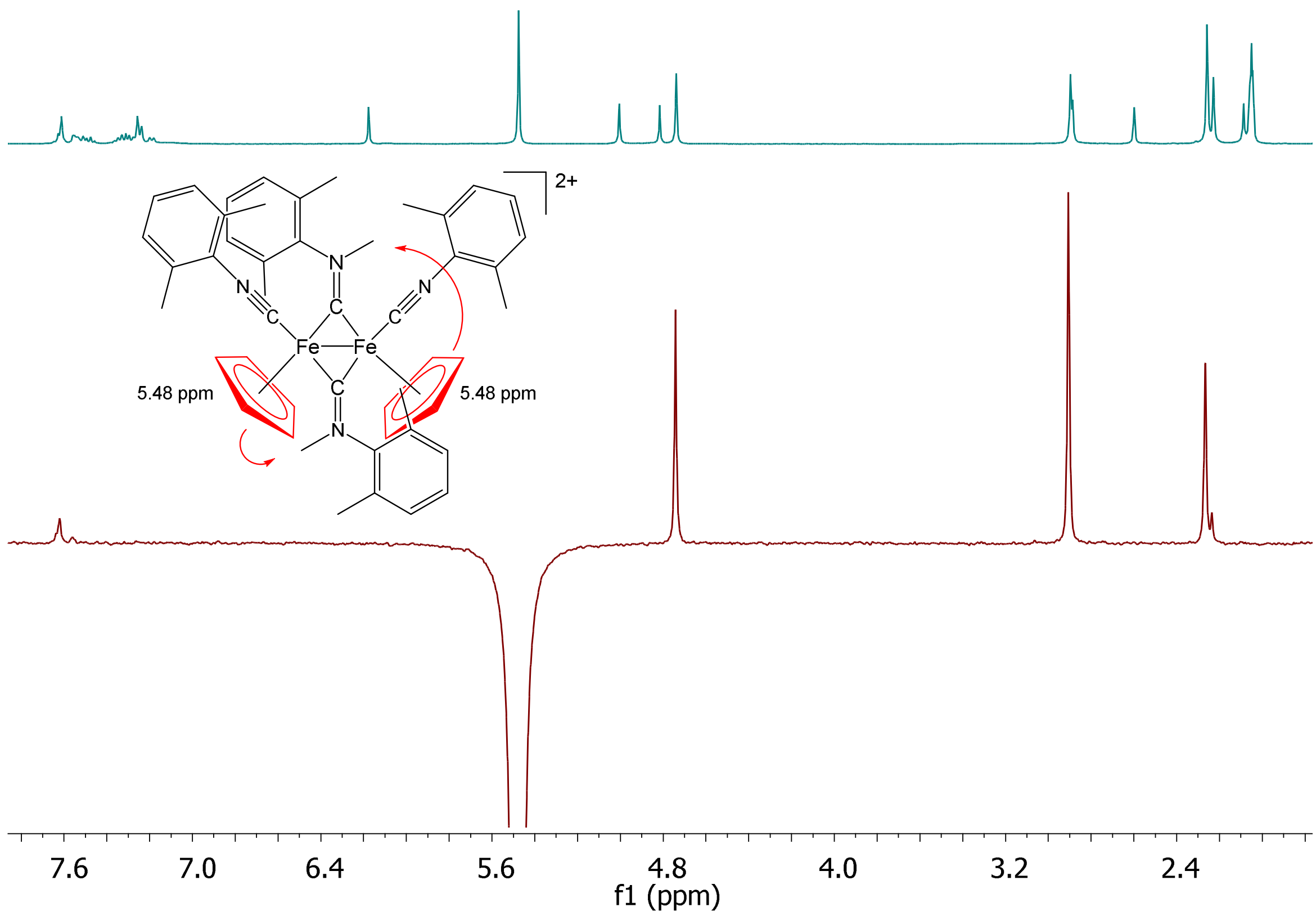

Figure S20. Blue trace: ^{1}H NMR spectrum (401 MHz, acetone-d6) of **[3a][CF$_3$SO$_3$]$_2$**. Red spectrum: ^{1}H NOESY spectrum with selective irradiation at 6.18 ppm (Cp resonance of the *cis-syn* isomer).

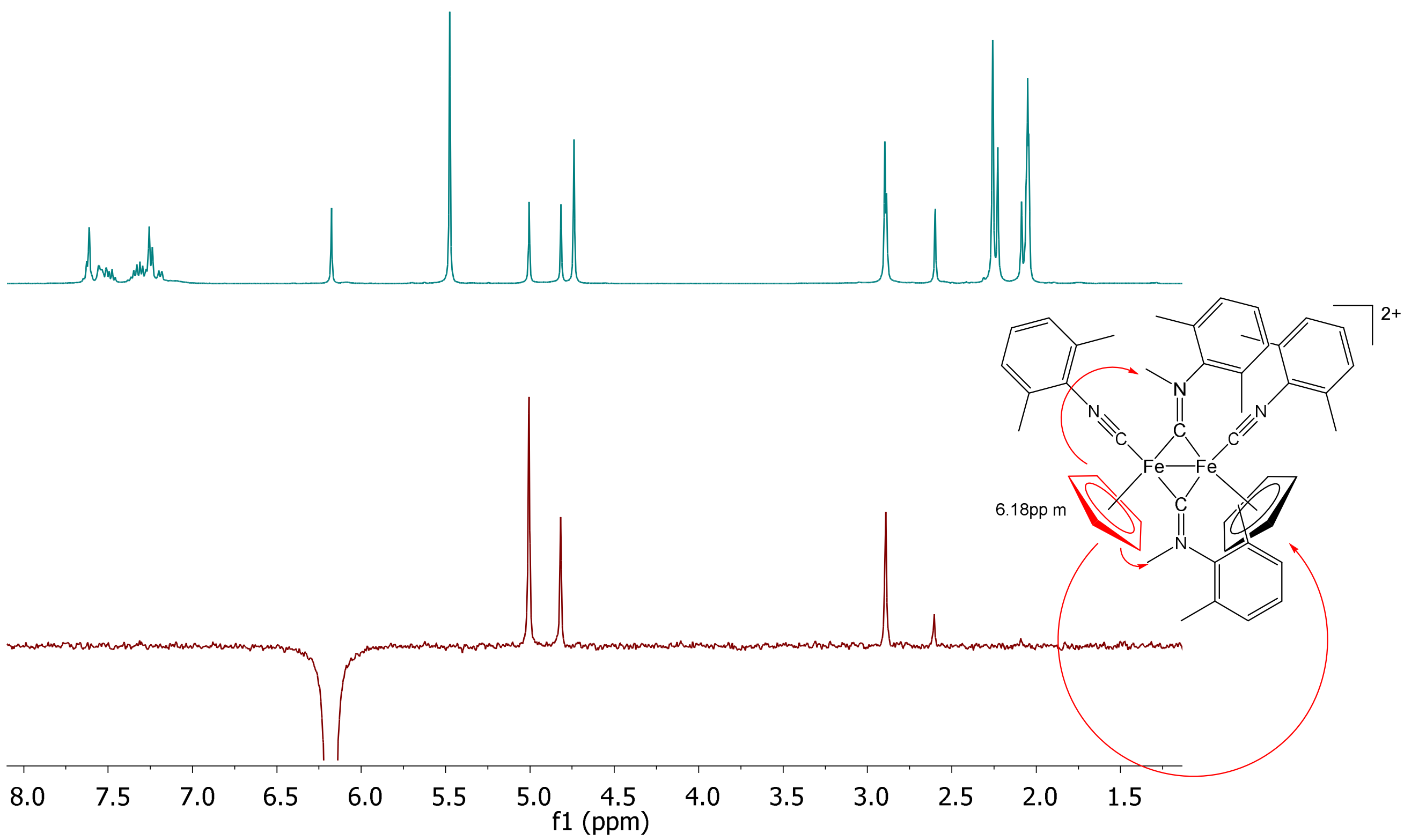

Figure S21. ^{1}H NMR spectrum (401 MHz, CD_3NO_2) of **[3b][CF₃SO₃]₂**. Integrals refer to the sum of the two isomers.

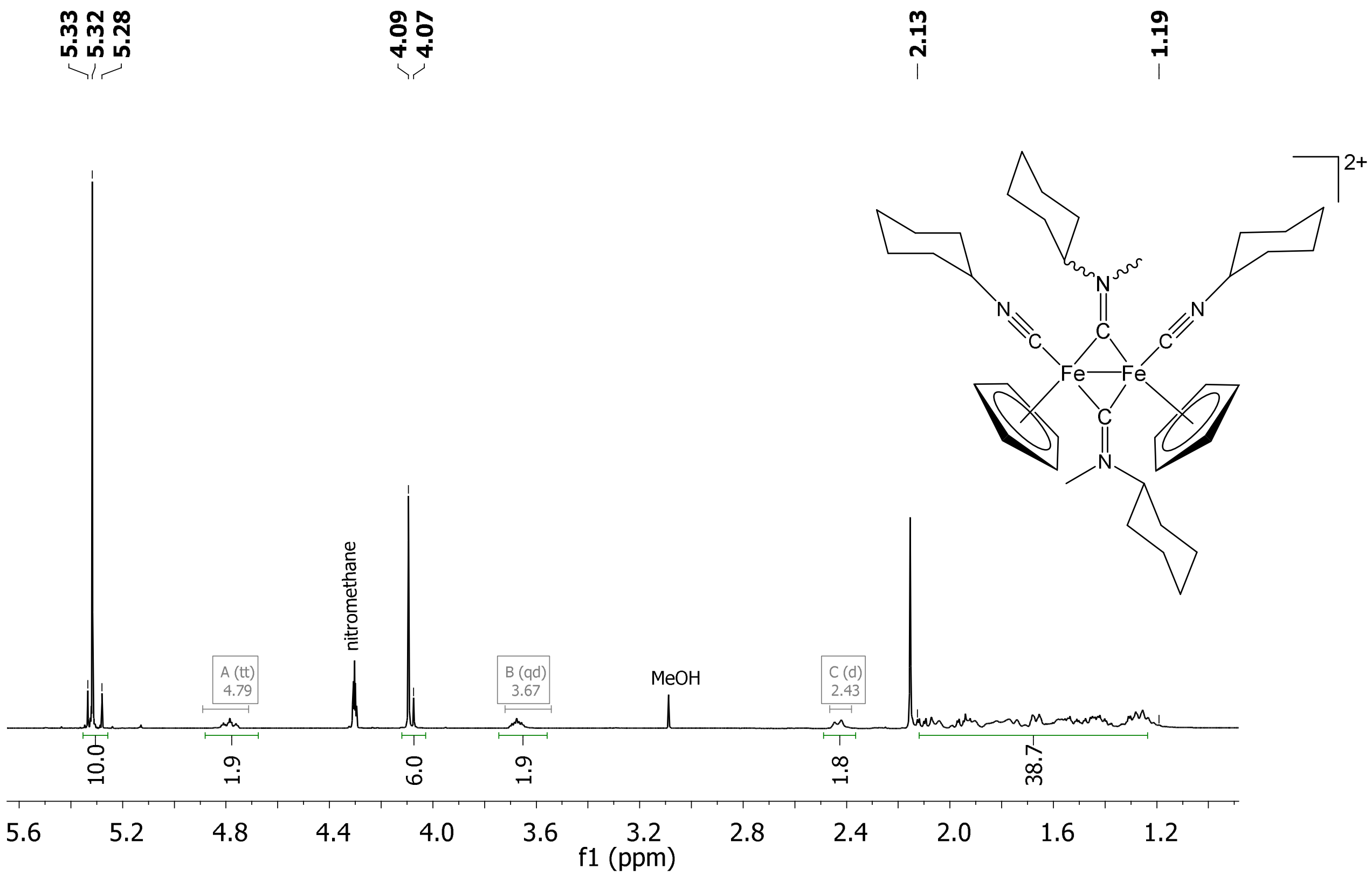

Figure S22. $^{13}C\{^{1}H\}$ NMR spectrum (401 MHz, CD_3NO_2) of **[3b][CF₃SO₃]₂**

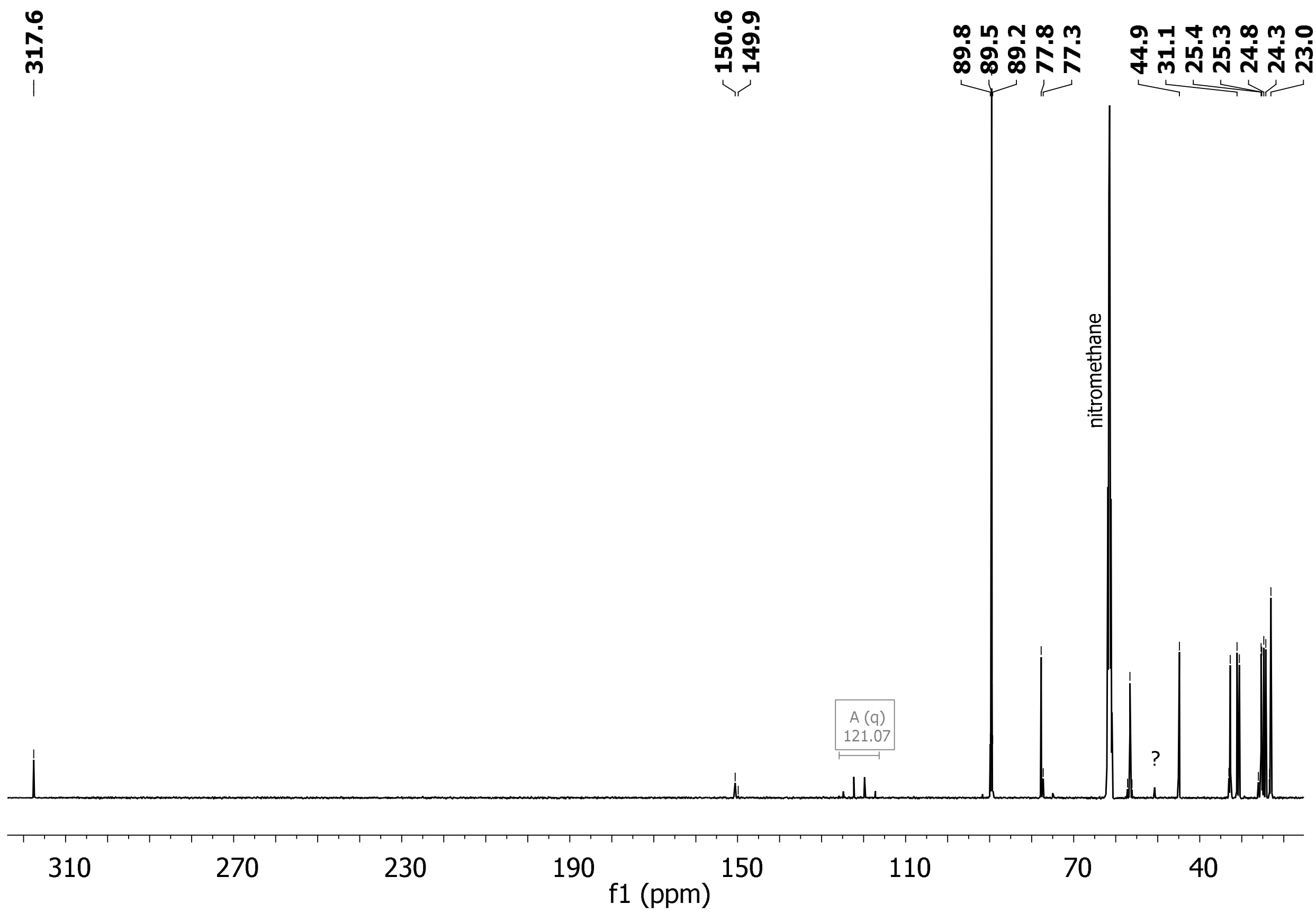

Figure S23. Blue trace: ^{1}H NMR spectrum (401 MHz, CD_3NO_2) of **[3b][CF₃SO₃]₂**. Green trace: ^{1}H NOESY spectrum with selective irradiation at 5.33 ppm (Cp resonance of the *cis-syn* isomer); Red trace: ^{1}H NOESY spectrum with selective irradiation at 5.28 ppm (Cp resonance of the *cis-syn* isomer).

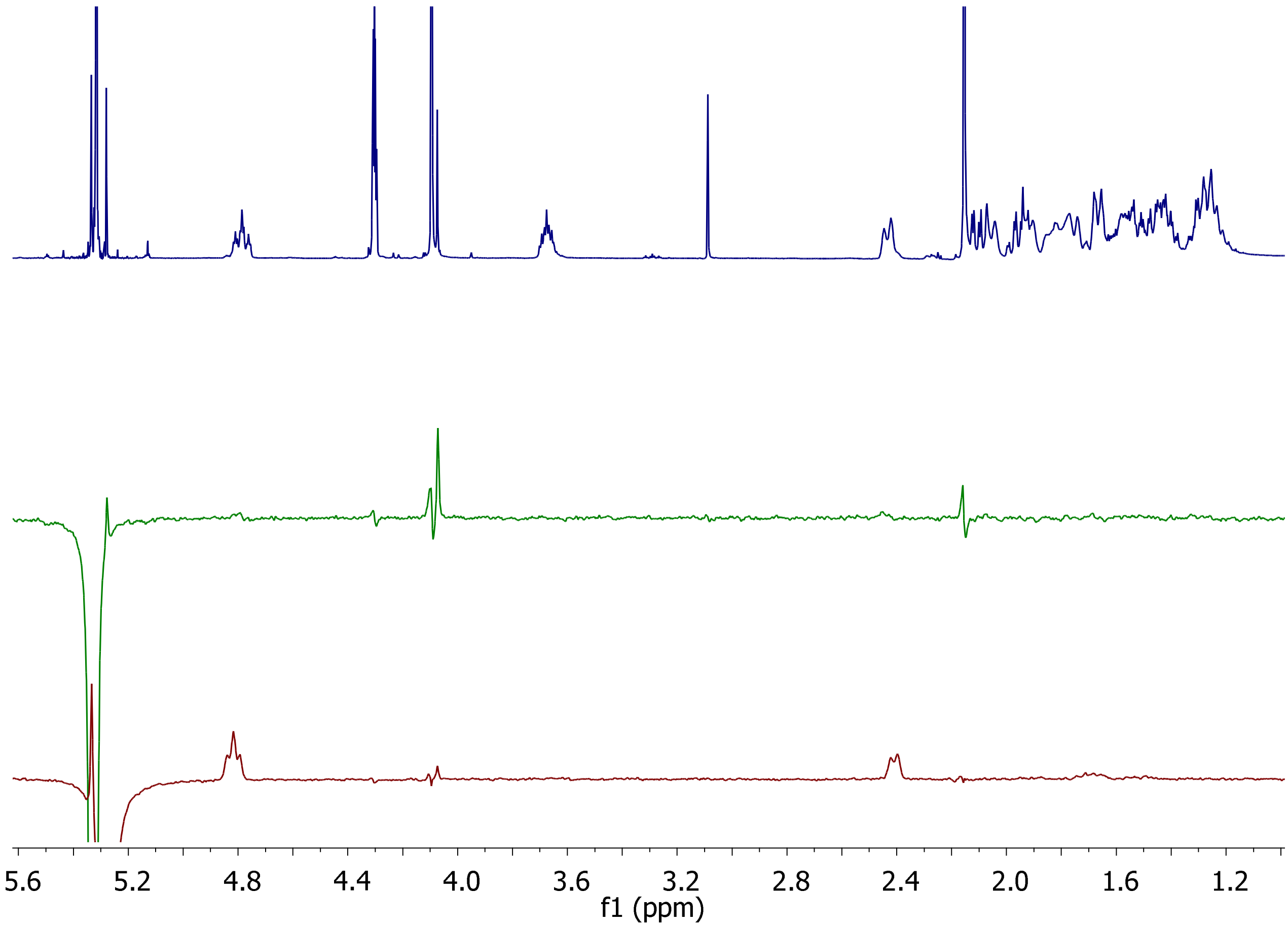

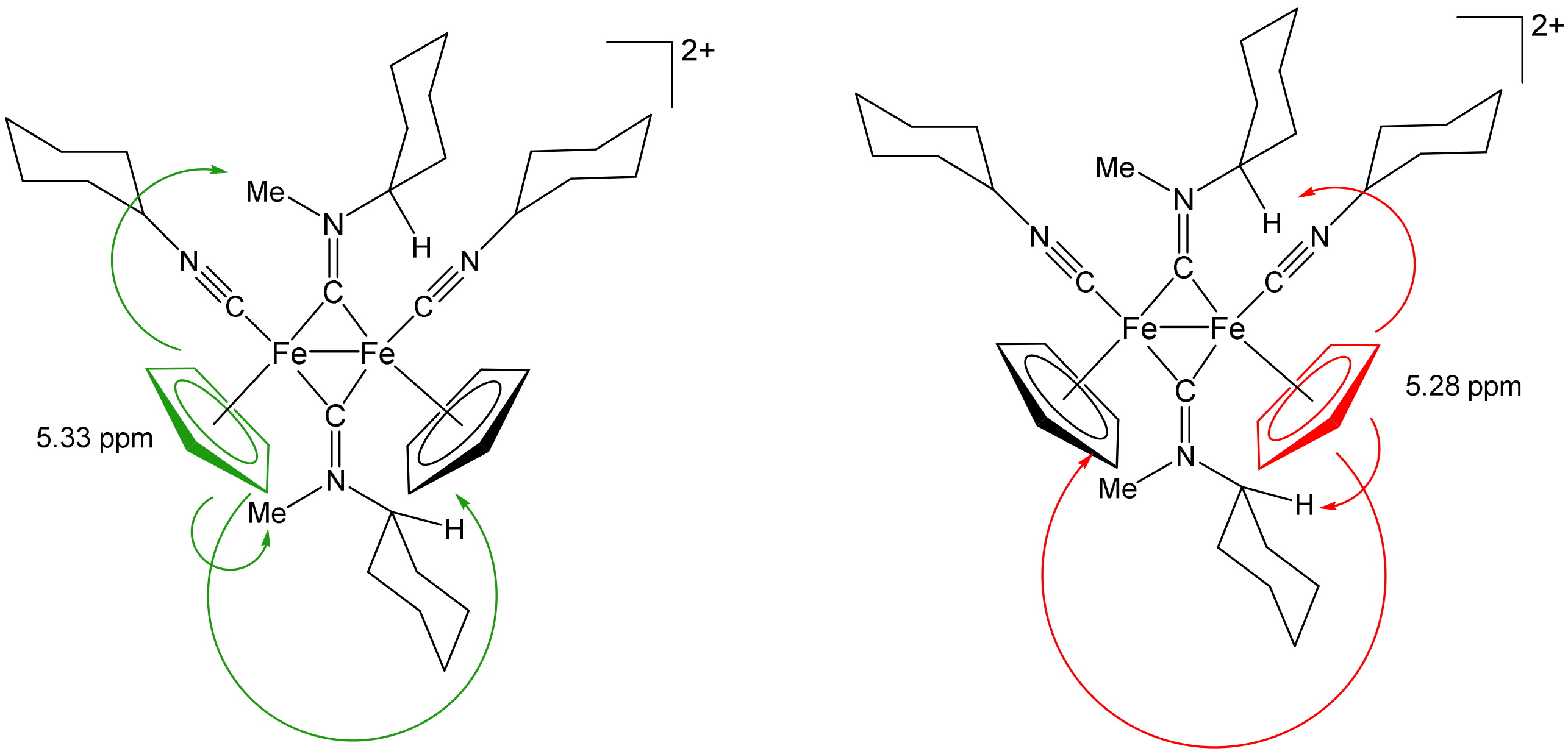

Figure S24. ^{1}H NMR spectrum (401 MHz, CD_3CN) of **[3c][CF₃SO₃]₂**

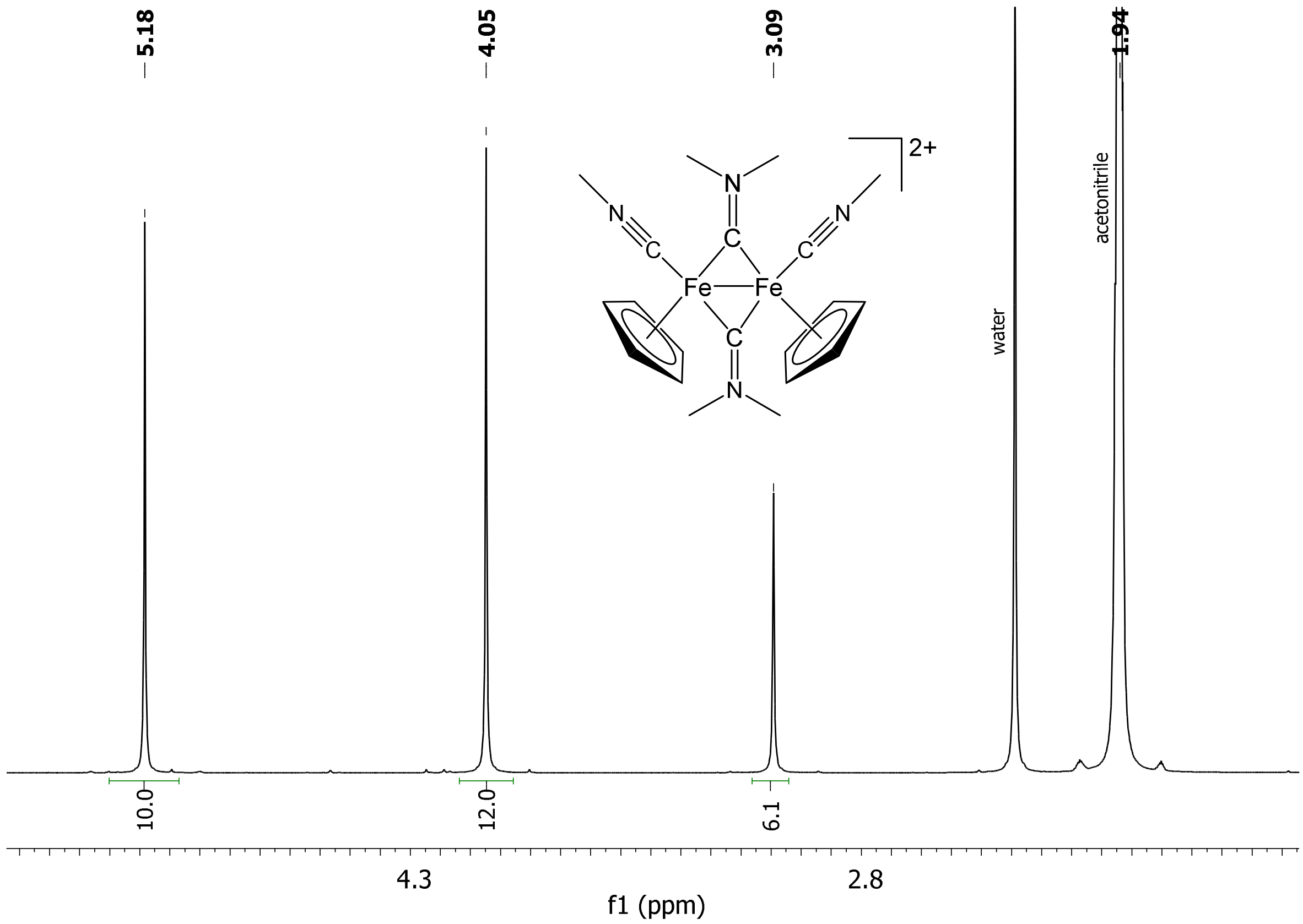

Figure S25. $^{13}C\{^{1}H\}$ NMR spectrum (401 MHz, CD_3CN) of **[3c][CF₃SO₃]₂**

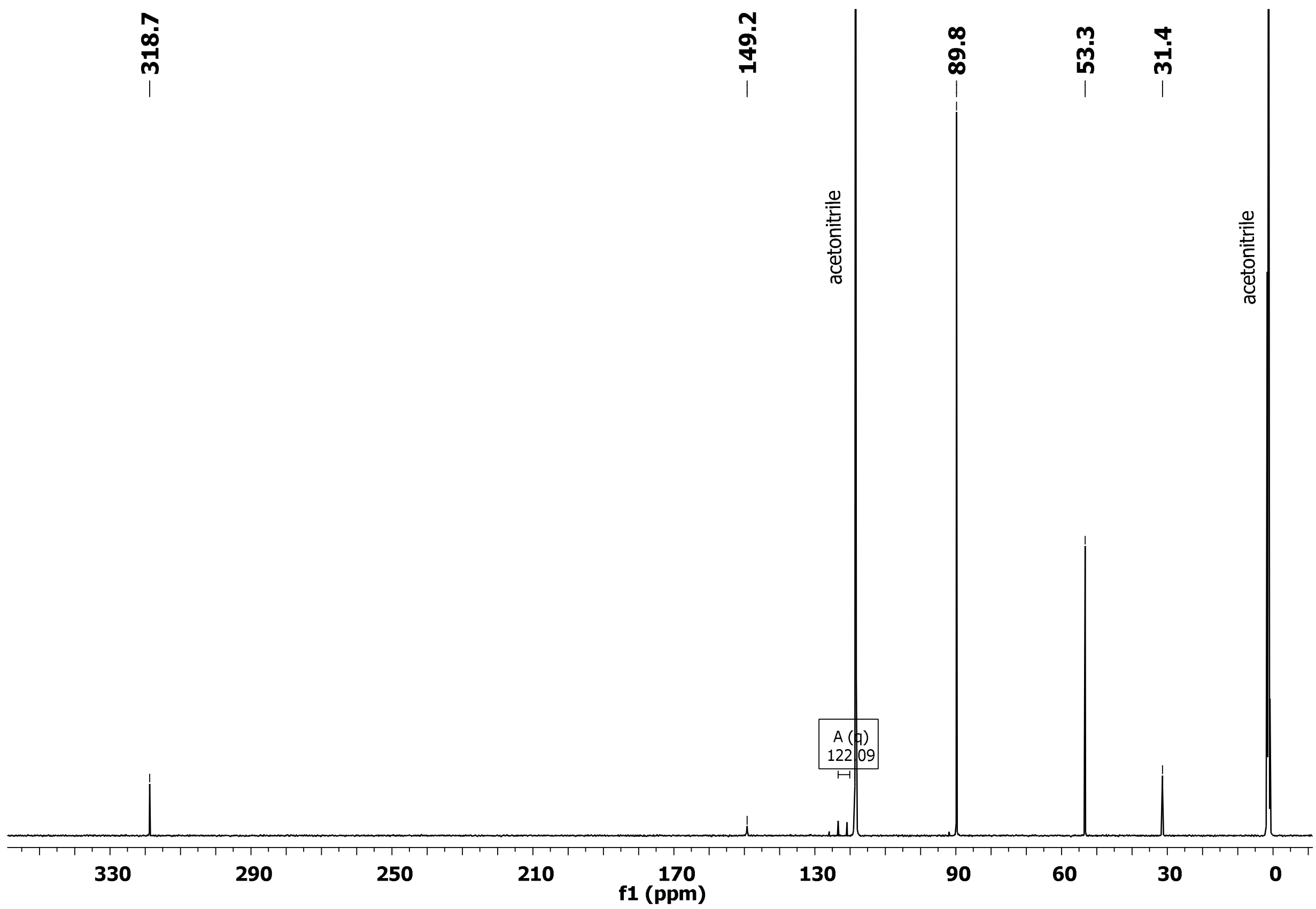

Scheme S1. Possible diastereoisomers of $[Fe_2Cp_2(CNR)_3\{CN(Me)(R)\}]^+$, **[2]**$^+$ (R ≠ Me), arising from the cis/trans orientation of the Cp ligands and the relative syn/anti arrangement of the identical R substituents on the bridging aminocarbyne and bridging isocyanide ligands. The corresponding overall point group symmetry is indicated for each isomer.

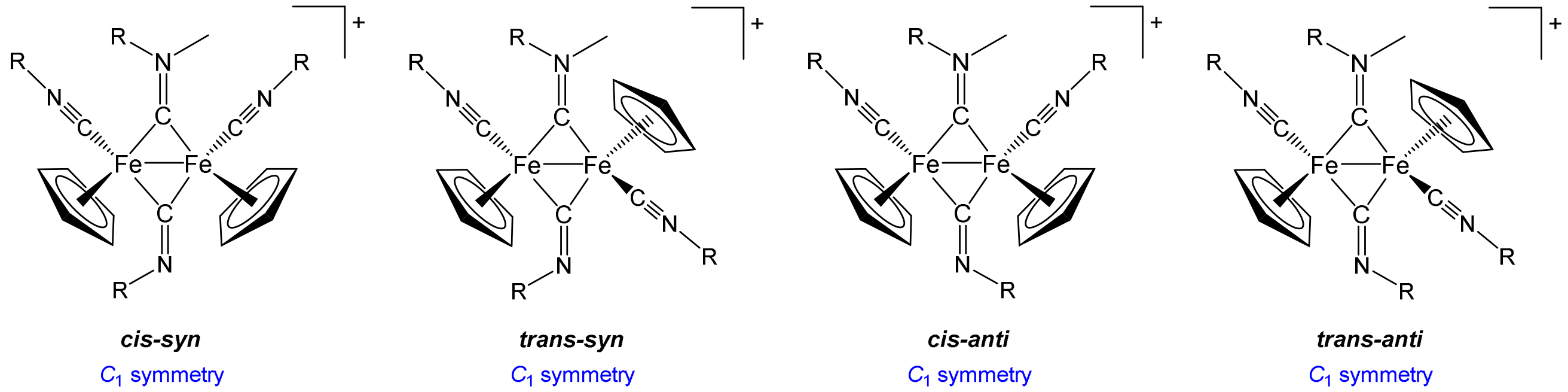

Scheme S2. Possible diastereoisomers of $[Fe_2Cp_2(CNR)_2\{CN(Me)(R)\}_2]^{2+}$, **[3]**$^{2+}$ (R ≠ Me), arising from the cis/trans orientation of the Cp ligands and the relative syn/anti arrangement of the identical R substituents on the two bridging aminocarbyne ligands. The relevant key symmetry elements and the corresponding overall point group symmetry are indicated for each isomer.

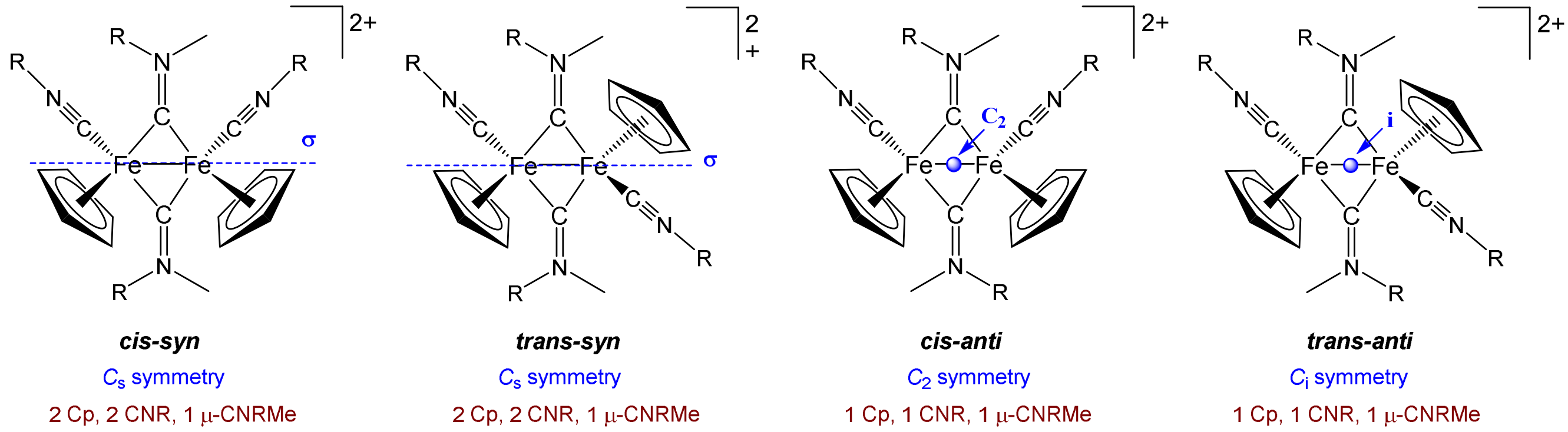

Figure S26. Schematic representation of *cis-anti*-(SS)-**[3b]**$^{2+}$, illustrating the assignment of configuration at the stereogenic iron centre according to the Cahn–Ingold–Prelog (CIP) rules. The priority order is CH_3 < Cp < Cy(trans) < Cy(cis) < CNCy. Atom numbering corresponds to that used in the CIF file.

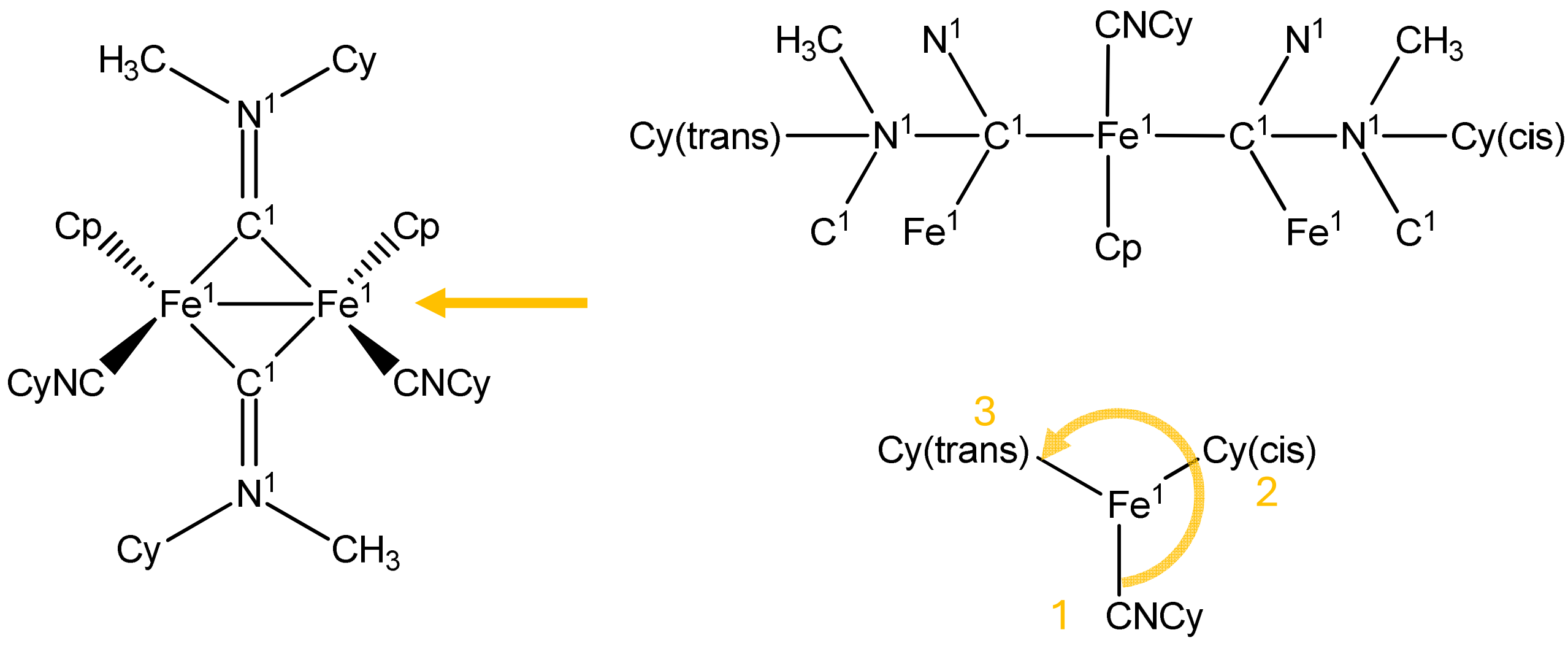

Figure S27. Superposition of the enantiomers *cis-anti*-(RR)-**[3b]**$^{2+}$ (yellow) and *cis-anti*-(SS)-**[3b]**$^{2+}$ (red). Hydrogen atoms are omitted for clarity.

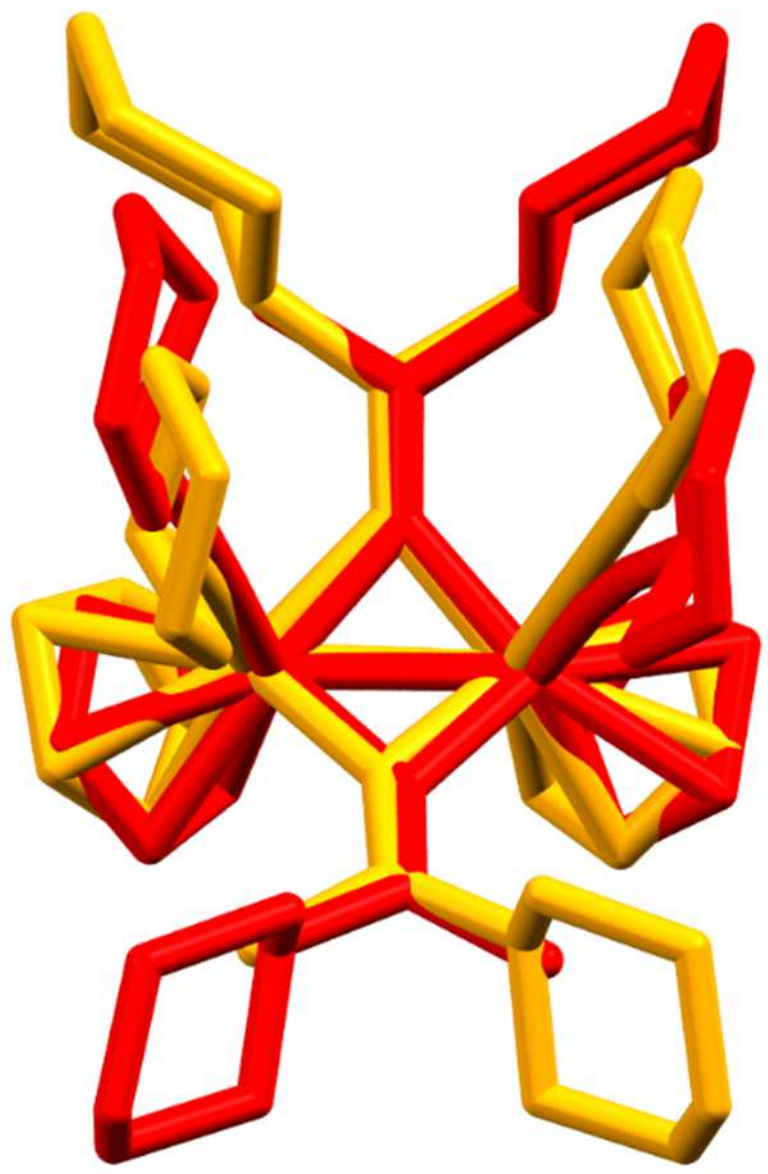

Table S2. Selected calculated bond lengths (Å) and angles (°) for **[2a,b]**$^{+}$ and **[3a,b]**$^{2+}$ in the *cis-anti* (CA) and *cis-syn* (CS) diastereomers. R (C^5-C^8) = *meta*-xylyl for **[2a]**$^{+}$ and **[3a]**$^{2+}$; cyclohexyl for **[2b]**$^{+}$ and **[3b]**$^{2+}$

	$[2a_{CA}]^{+}$	$[2a_{CS}]^{+}$	$[2b_{CA}]^{+}$	$[2b_{CS}]^{+}$	$[3a_{CA}]^{2+}$	$[3a_{CS}]^{2+}$	$[3b_{CA}]^{2+}$	$[3b_{CS}]^{2+}$
**Fe1-C1**	1.854	1.859	1.863	1.855	1.872	1.876	1.871	1.871
**Fe1-C2**	1.816	1.819	1.822	1.825	1.843	1.847	1.844	1.841
**Fe1-C3**	1.973	1.961	1.927	1.970	1.873	1.874	1.876	1.873
**Fe2-C1**	1.869	1.865	1.857	1.864	1.873	1.875	1.876	1.876
**Fe2-C3**	1.932	1.933	1.958	1.920	1.871	1.875	1.871	1.879
**Fe2-C4**	1.821	1.812	1.821	1.820	1.843	1.851	1.844	1.851
**C1-N1**	1.305	1.303	1.298	1.298	1.292	1.292	1.286	1.286
**N1-C7**	1.451	1.452	1.485	1.485	1.460	1.460	1.497	1.496
**C2-N2**	1.171	1.168	1.164	1.165	1.163	1.163	1.159	1.158
**C3-N3**	1.204	1.207	1.213	1.212	1.292	1.293	1.286	1.287
**C4-N4**	1.169	1.169	1.165	1.164	1.163	1.162	1.159	1.159
**N5-C5**	1.383	1.381	1.421	1.431	1.387	1.387	1.439	1.436
**N4-C6**	1.384	1.383	1.429	1.429	1.387	1.386	1.439	1.441
**Fe1-C2-N2**	173.2	173.8	175.6	173.9	172.9	173.6	175.4	178.6
**Fe2-C4-N4**	174.8	175.1	175.9	178.1	172.9	174.7	175.4	174.7
**C2-N2-C5**	170.0	171.8	169.7	173.2	170.5	179.0	174.7	176.4
**C4-N4-C6**	167.5	168.3	173.2	170.7	170.5	175.8	174.7	175.9
**C3-N3-C8**	143.3	141.4	129.6	130.8	123.8	123.4	122.0	122.1
**Sum angles N1**	360.0	360.0	360.0	360.0	360.0	360.0	360.0	360.0
**Sum angles C1**	359.9	360.0	359.9	359.9	360.0	359.9	359.9	359.9

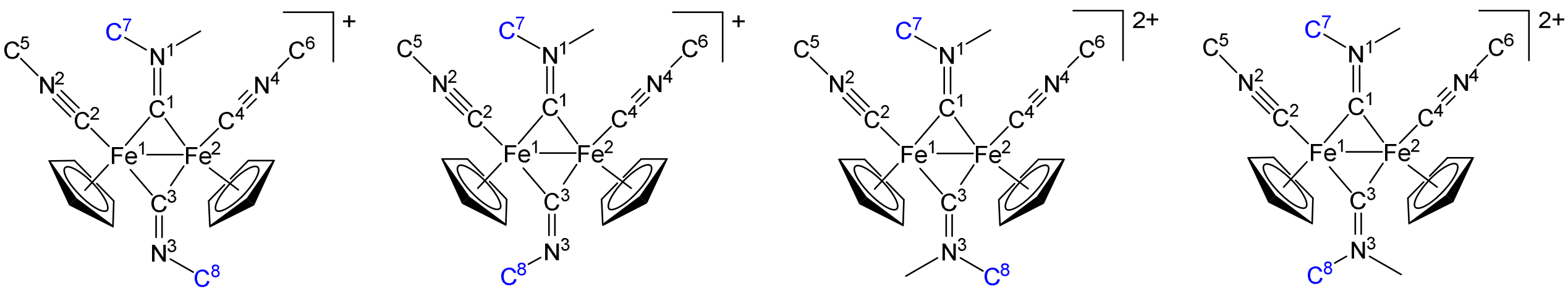

$[2_{CA}]^{+}$ $[2_{CS}]^{+}$ $[3_{CA}]^{+}$ $[3_{CS}]^{+}$

Table S3. Calculated Wiberg bond orders for [**2a,b**]$^+$ and [**3a,b**]$^{2+}$ in the *cis-anti* (CA) and *cis-syn* (CS) diastereomers. R (C^5-C^8) = *meta*-xylyl for **[2a]**$^+$ and **[3a]**$^{2+}$; cyclohexyl for **[2b]**$^+$ and **[3b]**$^{2+}$

	[2a$_{CA}$]$^+$	[2a$_{CS}$]$^+$	[2b$_{CA}$]$^+$	[2b$_{CS}$]$^+$	[3a$_{CA}$]$^{2+}$	[3a$_{CS}$]$^{2+}$	[3b$_{CA}$]$^{2+}$	[3b$_{CS}$]$^{2+}$
**Fe1-Fe2**	0.273	0.281	0.292	0.291	0.324	0.324	0.323	0.326
**Fe1-C1**	0.875	0.878	0.875	0.880	0.834	0.832	0.833	0.839
**Fe1-C2**	0.959	0.941	0.939	0.940	0.876	0.883	0.877	0.877
**Fe1-C3**	0.625	0.632	0.645	0.633	0.839	0.834	0.844	0.838
**Fe2-C1**	0.858	0.847	0.861	0.857	0.839	0.844	0.844	0.840
**Fe2-C3**	0.698	0.700	0.712	0.720	0.834	0.843	0.833	0.837
**Fe2-C4**	0.941	0.949	0.927	0.927	0.876	0.865	0.877	0.875
**C1-N1**	1.405	1.411	1.442	1.444	1.464	1.466	1.501	1.501
**C2-N2**	2.257	2.261	2.346	2.344	2.326	2.333	2.413	2.416
**C3-N3**	2.064	2.049	2.076	2.080	1.464	1.464	1.501	1.505
**C4-N4**	2.269	2.263	2.353	2.357	2.325	2.334	2.413	2.418
**N2-C5**	1.032	1.032	0.967	0.966	1.023	1.024	0.954	0.956
**N4-C6**	1.031	1.030	0.966	0.967	1.022	1.023	0.954	0.953
**N1-C7**	0.913	0.913	0.905	0.906	0.898	0.898	0.886	0.888
**N3-C8**	1.038	1.030	0.954	0.956	0.889	0.898	0.886	0.885

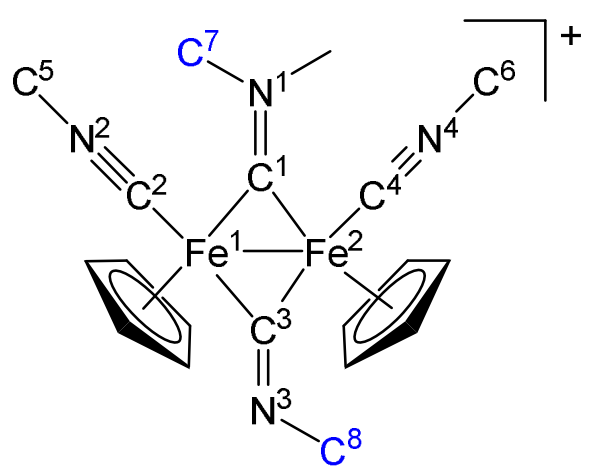

[2$_{CA}$]$^+$

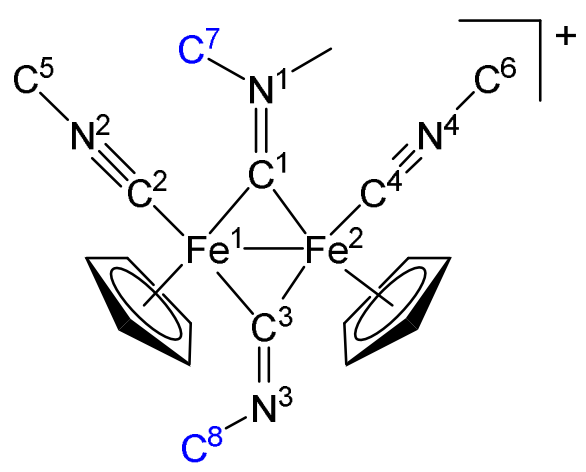

[2$_{CS}$]$^+$

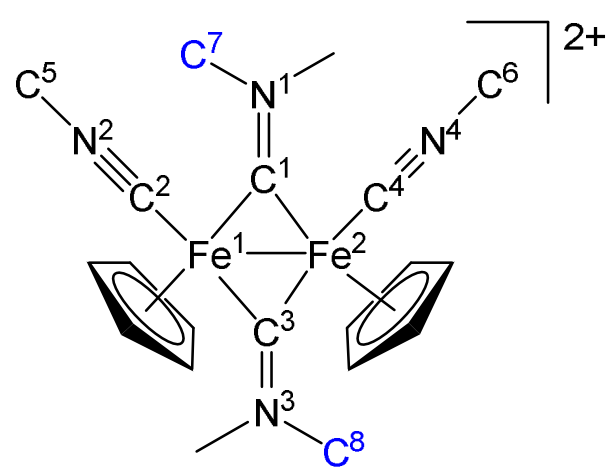

[3$_{CA}$]$^+$

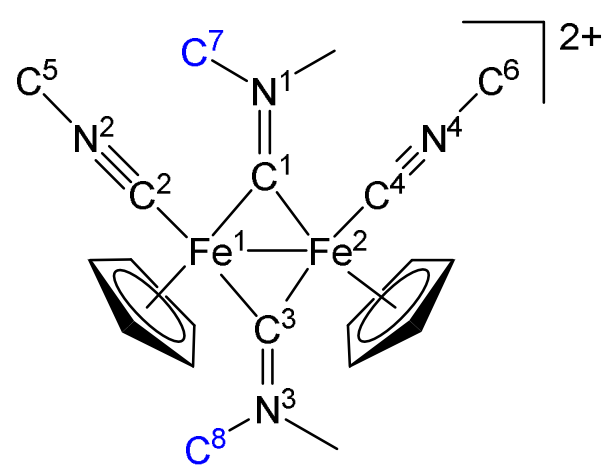

[3$_{CS}$]$^+$

Table S4. Selected calculated bond lengths (Å) and angles (°) for **[2a,b^{CO}]$^{+}$** and **[3a,b^{CO}]$^{2+}$**. For **[3a,b^{CO}]$^{2+}$** only the *cis-anti* diastereomer is included for clarity. R = *meta*-xylyl for **[2a^{CO}]$^{+}$** and **[3a^{CO}]$^{2+}$**; cyclohexyl for **[2b^{CO}]$^{+}$** and **[3b^{CO}]$^{2+}$**

	[2a^{CO}]$^{+}$	[2b^{CO}]$^{+}$	[3a^{CO}]$^{2+}$	[3b^{CO}]$^{2+}$
**Fe1-C1**	1.869	1.879	1.890	1.893
**Fe2-C1**	1.876	1.873	1.882	1.888
**C1-N1**	1.295	1.290	1.285	1.279
**Sum angles N1**	360.0	360.0	360.0	360.0
**Sum angles C1**	360.0	359.9	360.0	359.9

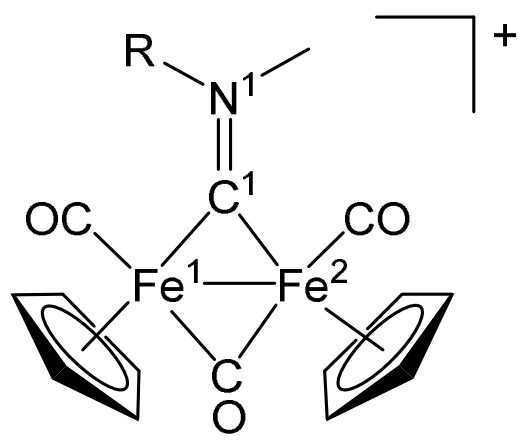

[2^{CO}]$^{+}$

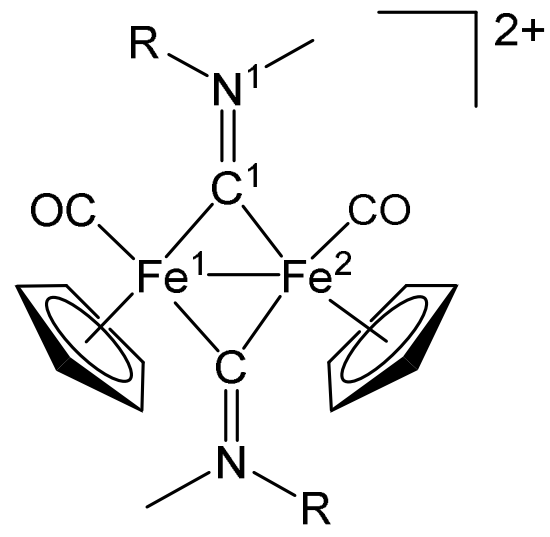

[3^{CO}]$^{+}$

Table S5. Calculated Wiberg bond orders for [**2a,b^{CO}**]$^{+}$ and [**3a,b^{CO}**]$^{2+}$. For [**3a,b^{CO}**]$^{2+}$ only the *cis-anti* diastereomer is included for clarity.

	[2a^{CO}]$^{+}$	[2b^{CO}]$^{+}$	[3a^{CO}]$^{2+}$	[3b^{CO}]$^{2+}$
**Fe1-Fe2**	0.281	0.282	0.309	0.305
**Fe1-C1**	0.851	0.853	0.815	0.813
**Fe2-C1**	0.849	0.842	0.818	0.823
**C1-N1**	1.458	1.500	1.513	1.557

Figure S28. Superposition of the DFT-optimized geometries (purple) with the corresponding single-crystal X-ray diffraction structures (orange), highlighting the excellent agreement for the $Fe_2(\mu\text{-}C)_2$ core.

$[\mathbf{2a}]^{+}$

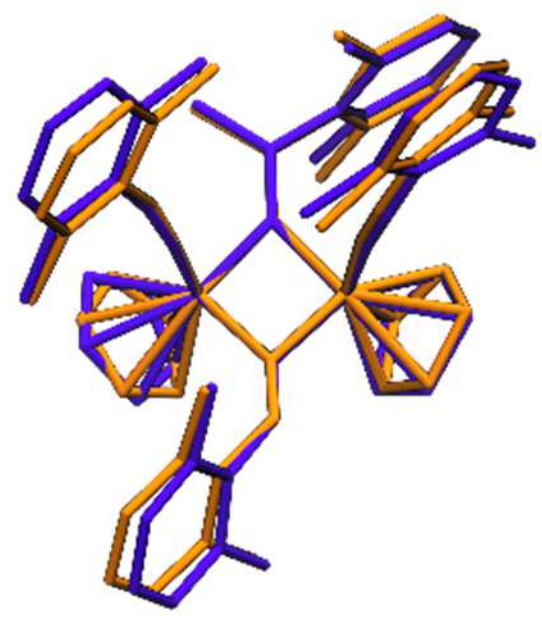

$[\mathbf{3a}]^{2+}$

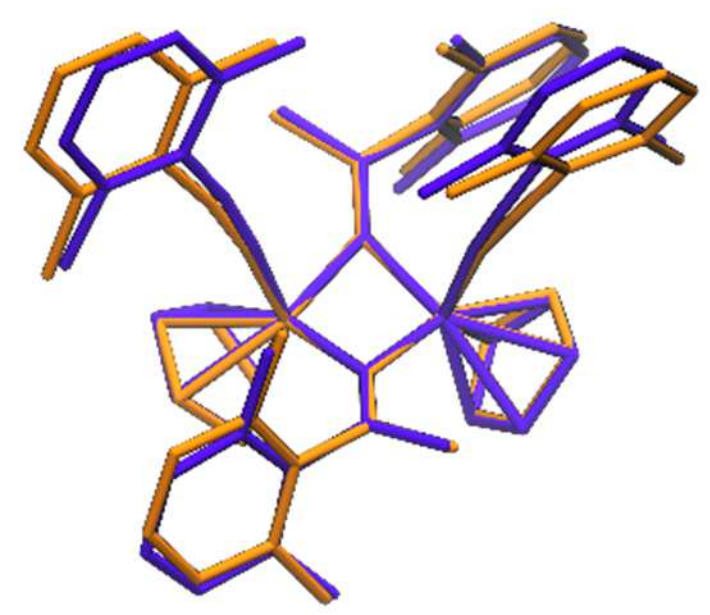

$[\mathbf{3b}]^{2+}$

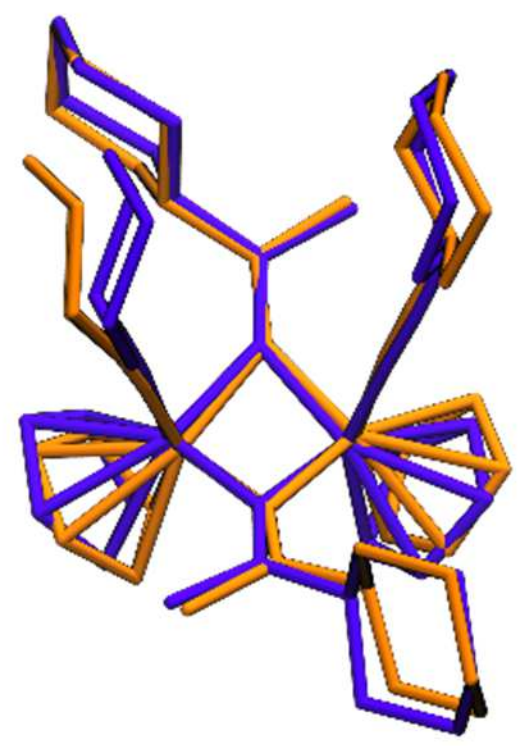

Figure S29. Computed IR spectra of the oxidized and reduced forms of [**2a**]$^{+}$

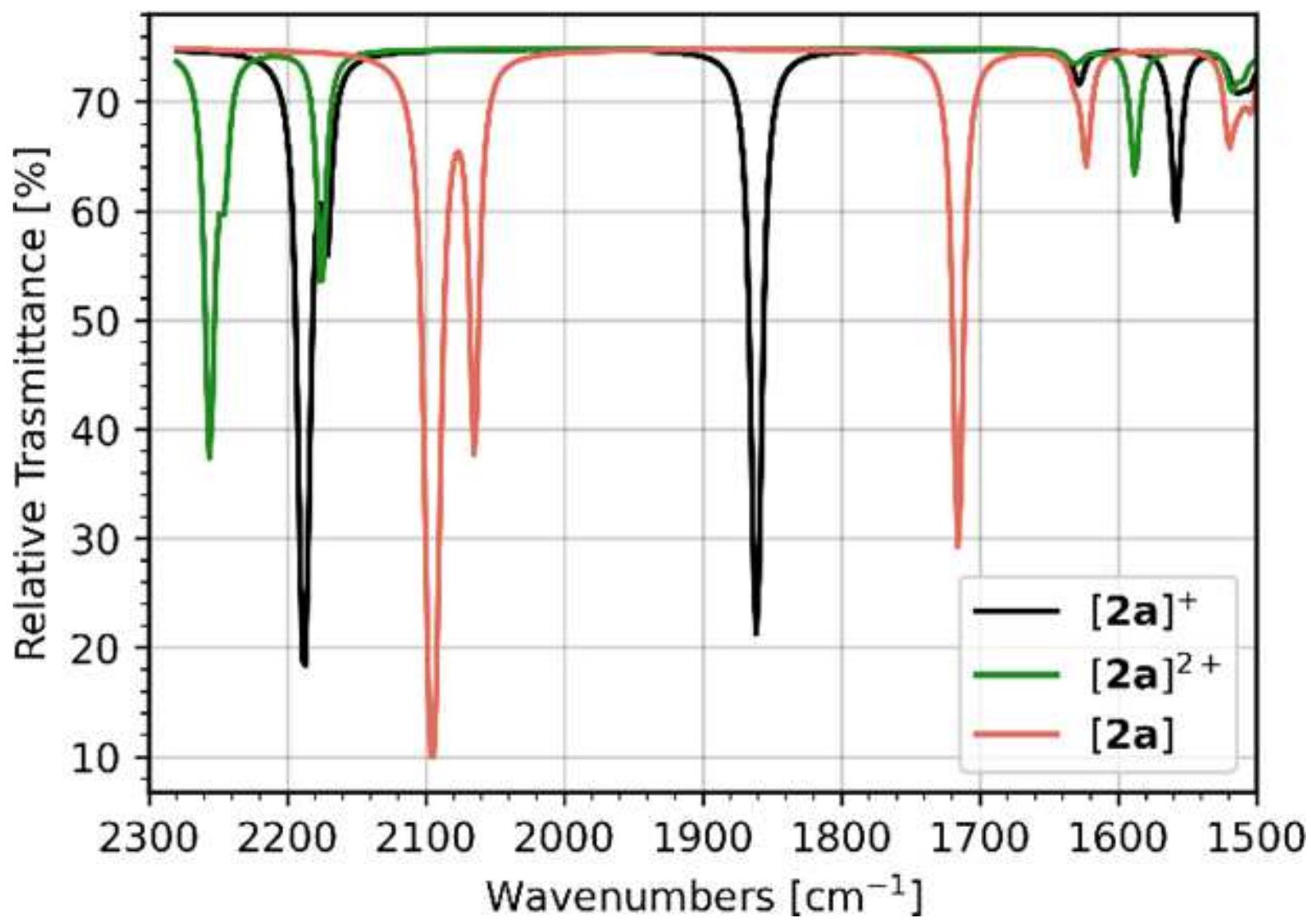

Figure S30. Computed IR spectra of the oxidized and reduced forms of [**3a**]$^{2+}$

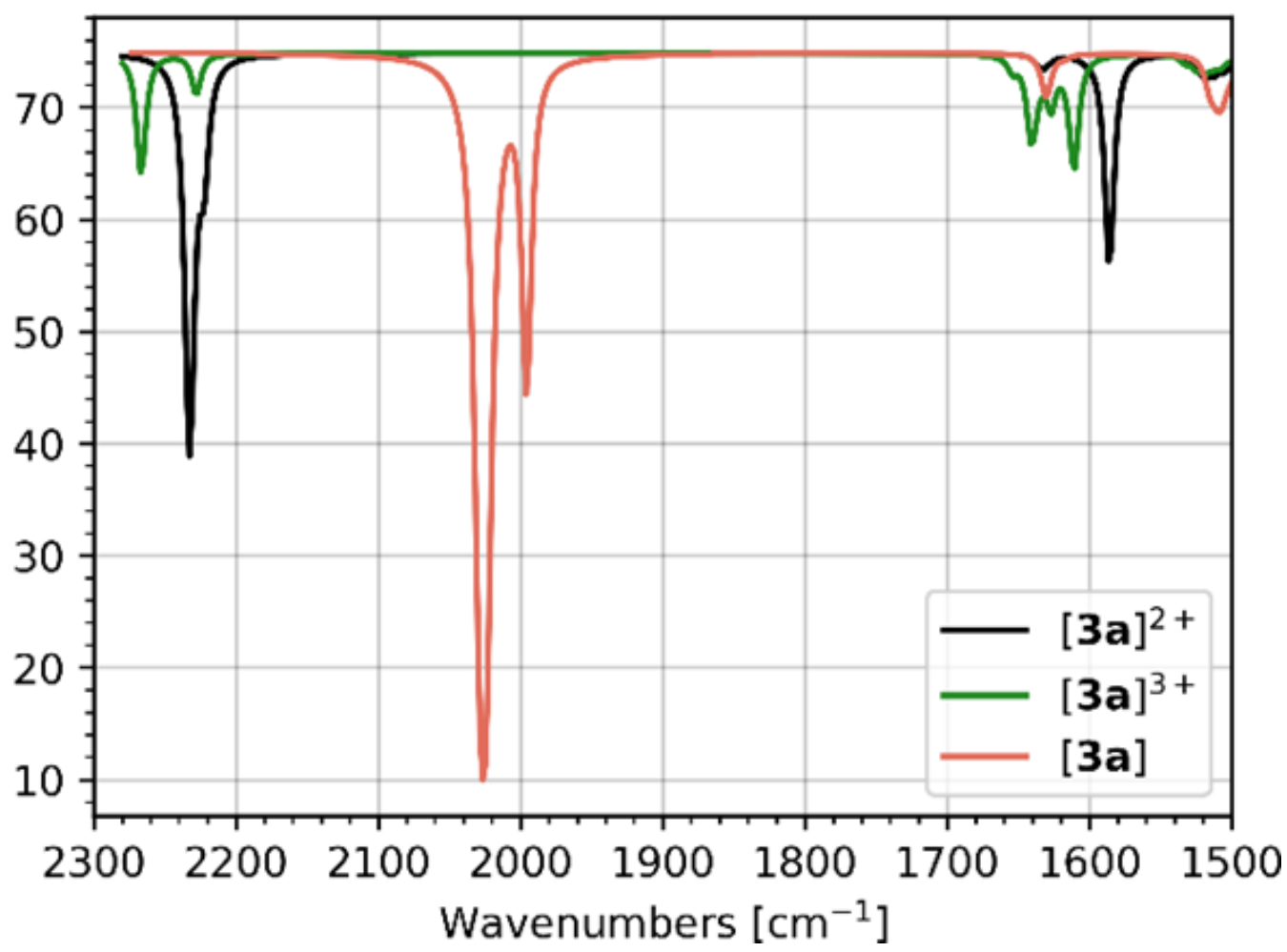

Table S6. Selected calculated bond lengths (Å) and angles (°) for [**2a**], [**2a**]$^{+}$, [**2a**]$^{2+}$, [**2a**]$^{3+}$, [**3a**], and [**3a**]$^{3+}$ in the *cis-anti* diastereomers, respectively.

	[**2a**]	[**2a**]$^{+}$	[**2a**]$^{2+}$	[**2a**]$^{3+}$	[**3a**]	[**3a**]$^{2+}$	[**3a**]$^{3+}$
**Fe1-C1**	1.880	1.855	1.839	1.882	1.908	1.871	1.961
**Fe1-C2**	1.782	1.815	1.865	1.862	1.749	1.837	1.896
**Fe1-C3**	1.939	1.956	2.446	1.886	1.925	1.873	1.982
**Fe2-C1**	1.884	1.869	1.934	1.898	1.925	1.874	1.845
**Fe2-C3**	1.933	1.926	1.853	2.109	1.907	1.872	1.823
**Fe2-C4**	1.784	1.818	1.838	1.867	1.749	1.836	1.870
**C1-N1**	1.312	1.302	1.293	1.283	1.324	1.292	1.281
**C2-N2**	1.183	1.170	1.160	1.157	1.194	1.163	1.162
**C3-N3**	1.242	1.213	1.168	1.176	1.324	1.292	1.280
**C4-N4**	1.182	1.169	1.161	1.158	1.194	1.163	1.159
**N2-C5**	1.378	1.381	1.383	1.383	1.376	1.384	1.372
**N4-C6**	1.380	1.382	1.382	1.382	1.376	1.384	1.388
**Fe1-C2-N2**	173.5	172.2	176.6	173.9	172.6	173.4	173.1
**Fe2-C4-N4**	176.2	175.0	174.8	175.2	172.6	172.6	171.6
**C2-N2-C5**	163.0	170.1	177.2	175.2	154.8	170.6	172.4
**C4-N4-C6**	160.4	167.0	176.4	175.5	154.9	170.3	173.0
**C3-N3-C8**	133.0	139.2	168.9	178.0	122.7	121.7	122.2
**Sum angles N1**	360.0	360.0	360.0	360.0	360.0	360.0	360.0
**Sum angles C1**	359.9	360.0	360.0	360.0	360.0	360.0	360.0

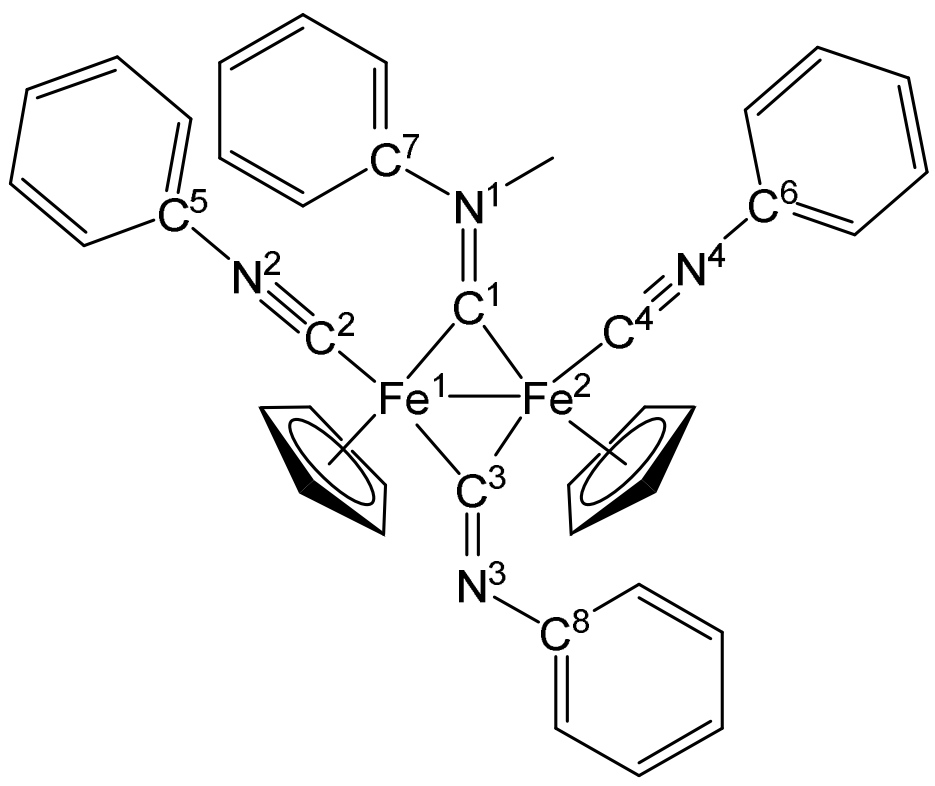

[2a]$^{0,+2,+3}$

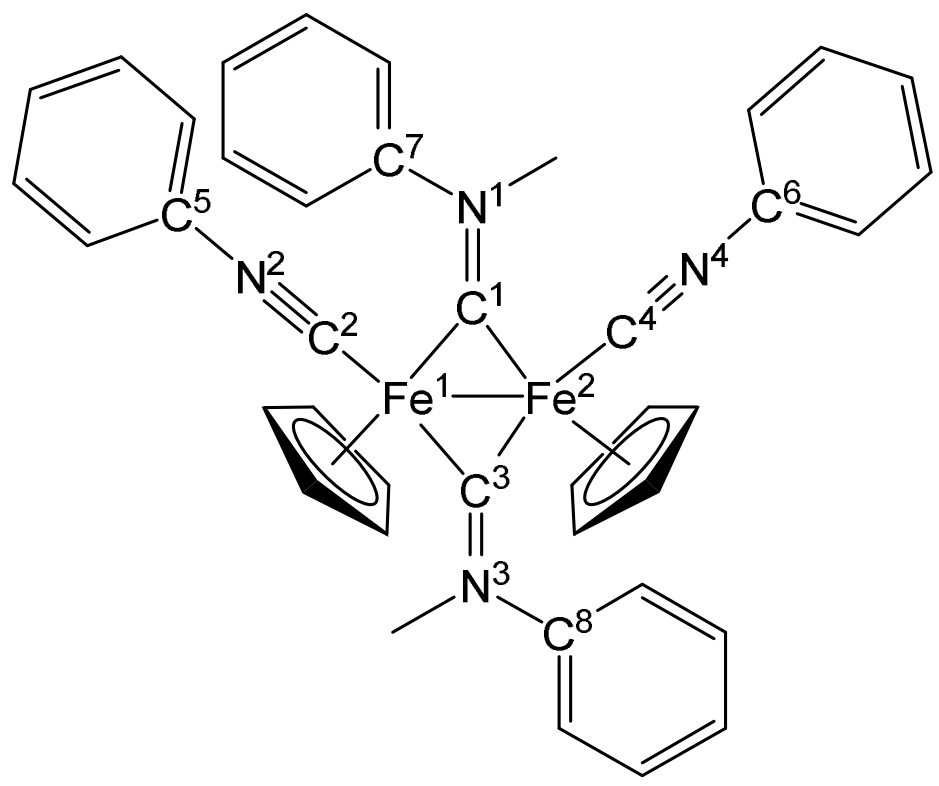

[3a]$^{0,+3}$

Table S7. Calculated Wiberg bond orders for [**2a**], [**2a**]$^{+}$, [**2a**]$^{2+}$, [**2a**]$^{3+}$, [**3a**], and [**3a**]$^{3+}$ in the *cis-anti* diastereomers.

	[**2a**]	[**2a**]$^{+}$	[**2a**]$^{2+}$	[**2a**]$^{3+}$	[**3a**]	[**3a**]$^{2+}$	[**3a**]$^{3+}$
**Fe1-Fe2**	< 0.2	0.273	0.279	0.382	< 0.2	0.324	0.264
**Fe1-C1**	0.834	0.875	0.898	0.830	0.819	0.834	0.700
**Fe1-C2**	1.030	0.959	0.861	0.855	1.126	0.876	0.831
**Fe1-C3**	0.702	0.625	0.211	0.760	0.817	0.839	0.666
**Fe2-C1**	0.840	0.858	0.767	0.809	0.817	0.839	0.898
**Fe2-C3**	0.720	0.698	0.846	0.401	0.820	0.834	0.946
**Fe2-C4**	1.015	0.941	0.893	0.853	1.126	0.876	0.826
**C1-N1**	1.363	1.405	1.476	1.514	1.340	1.464	1.525
**C2-N2**	2.114	2.257	2.363	2.387	2.068	2.326	2.346
**C3-N3**	1.890	2.064	2.267	2.152	1.341	1.464	1.530
**C4-N4**	2.159	2.269	2.330	2.370	2.069	2.325	2.381
**N1-C7**	0.922	0.913	0.900	0.885	0.925	0.898	0.880
**N2-C5**	1.051	1.032	1.027	1.026	1.063	1.023	1.072
**N4-C6**	1.050	1.031	1.025	1.038	1.063	1.022	1.023
**N3-C8**	1.064	1.038	1.033	1.162	0.925	0.889	0.885

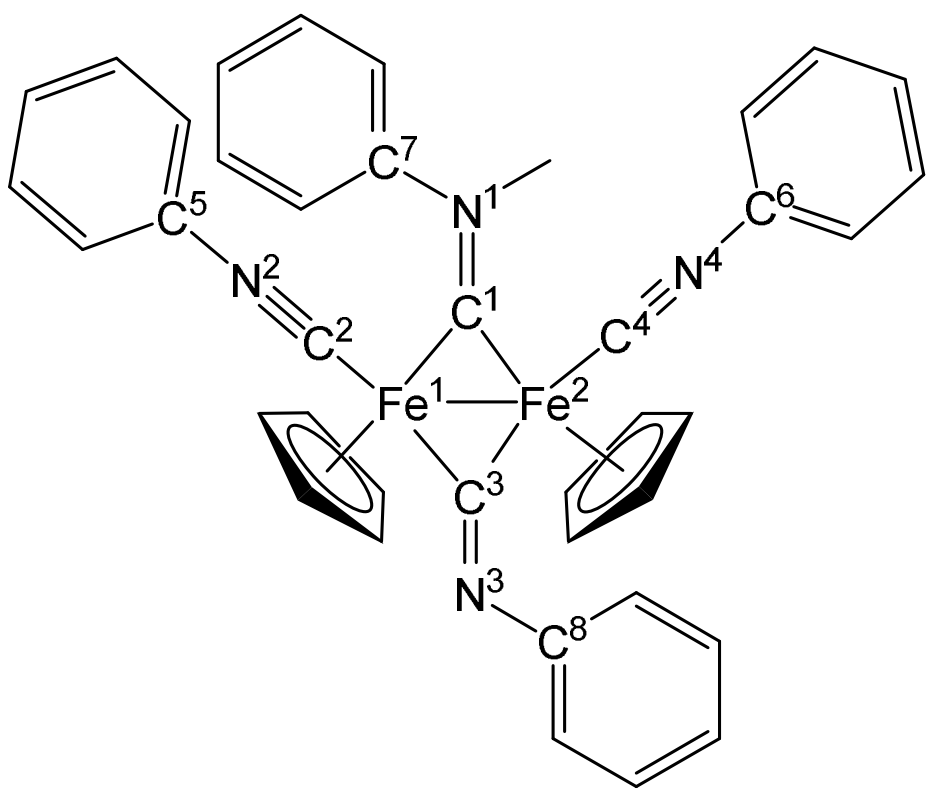

[2a]$^{0,+2,+3}$

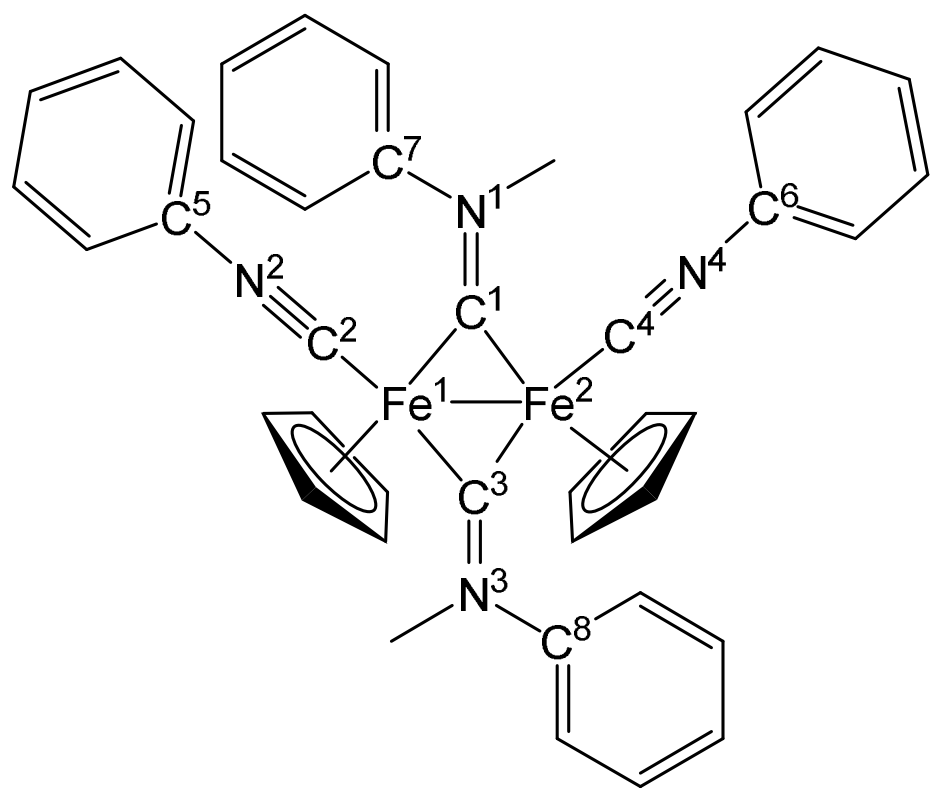

[3a]$^{0,+3}$

Figure S31. Distribution of C–N bond lengths (1.1–1.5 Å) for bridging aminocarbyne ligands in diiron complexes retrieved from a Cambridge Structural Database (CSD – Aug 2025) search under the criteria shown. The Fe–C bond distance was left unrestricted, and R was considered as aryl or alkyl substituent. A total of 48 structures was identified, with a median C-N distance of 1.304 Å and a range of 1.266-1.374 Å.

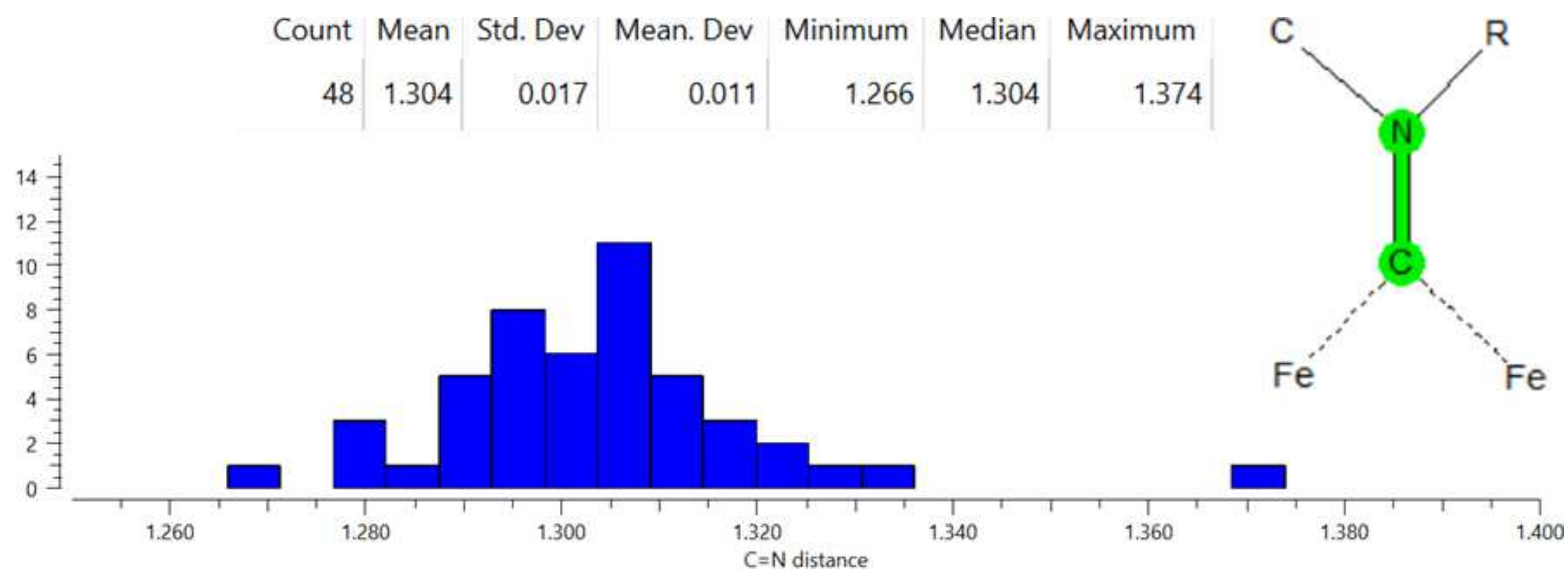

Figure S32. Photograph of single crystals of *cis-anti*-**[3b][CF$_3$SO$_2$]$_2$** and *cis-syn*-**[3b][CF$_3$SO$_2$]$_2$** (green), obtained from the same crystallization batch.

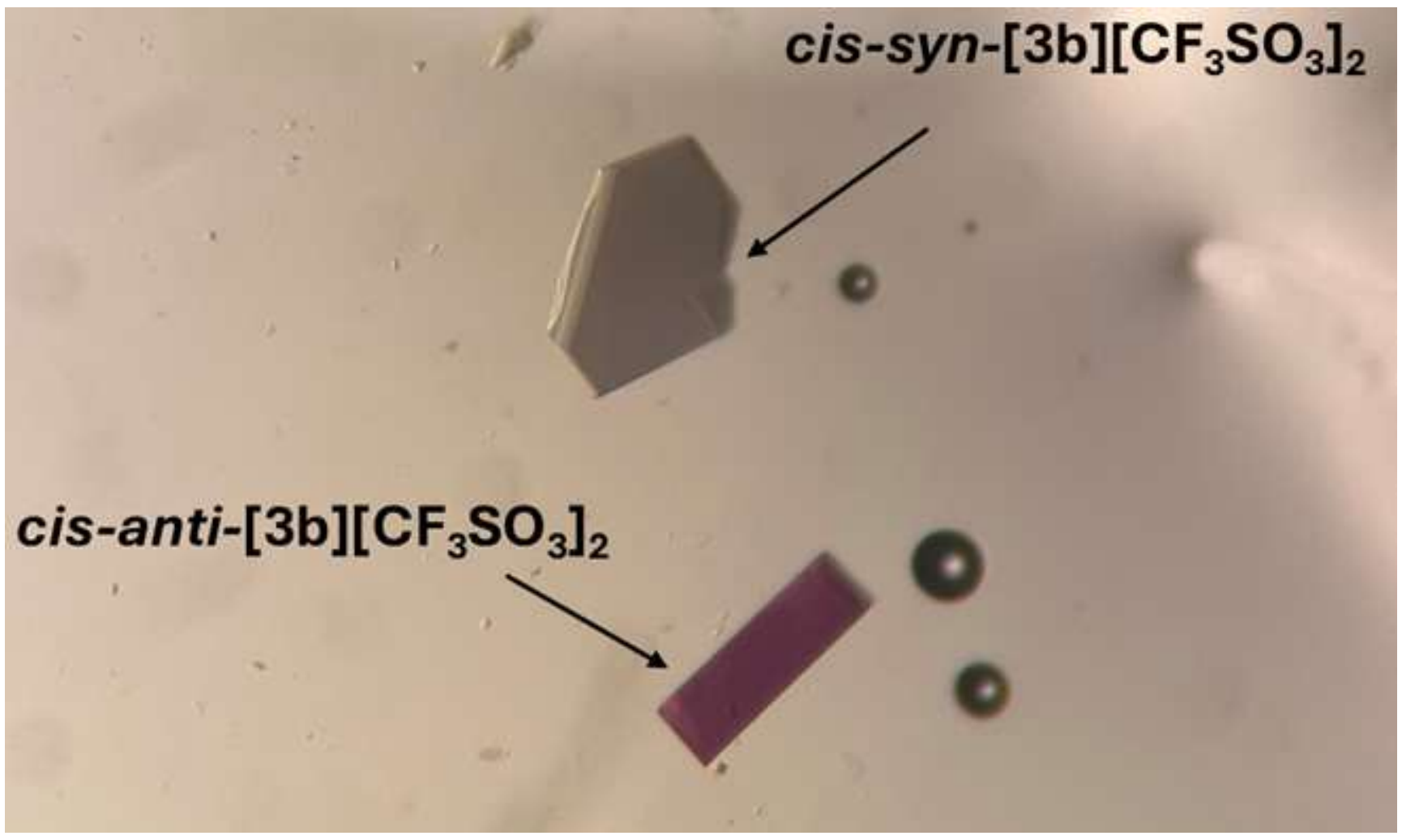

Figure S33. Asymmetric unit of *cis-syn*-**[3b][CF$_3$SO$_2$]$_2$** (H atoms omitted for clarity). This achiral compound crystallizes as green plates in the centrosymmetric space group P$_{bcn}$; the asymmetric unit comprises two crystallographically independent dimers and four non-equivalent triflate anions.

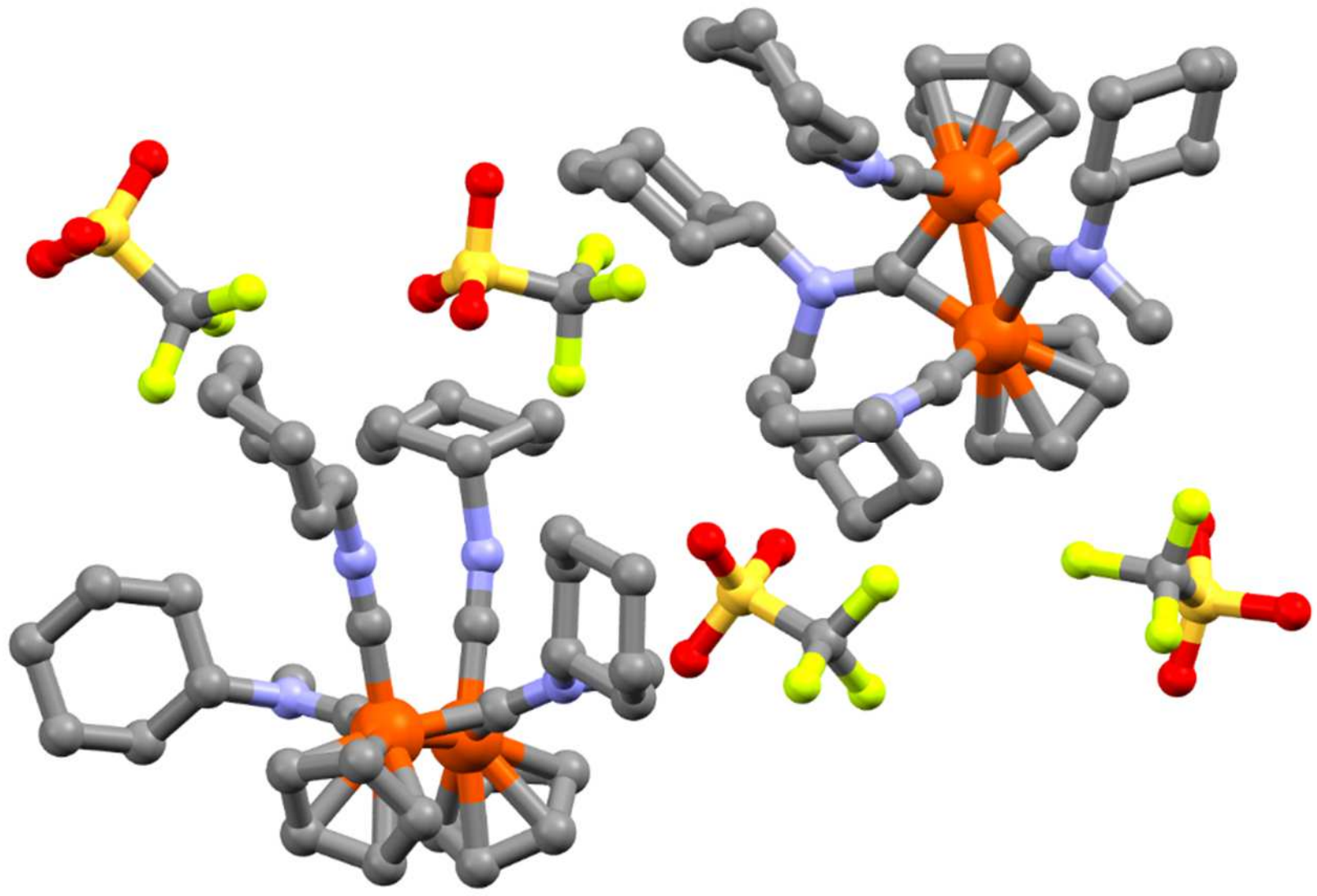

Figure S34. Precession electron diffraction images of the reciprocal lattice planes 0kl, h0l, and hk0 for *cis-anti*-(SS)-**[3b][CF$_3$SO$_2$]$_2$.** While the 0kl and hk0 projections display sharp and well-defined reflections, the h0l plane exhibits elongated and partially split spots, consistent with non-merohedral twinning along this reciprocal direction.

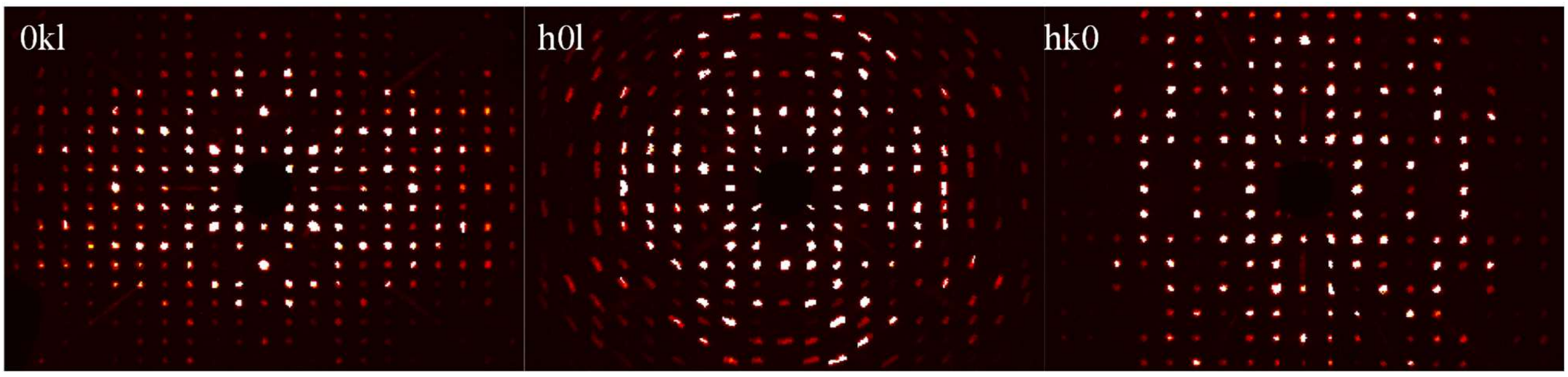

Figure S35. Cyclic voltammogram recorded at a Pt electrode in a CH_3CN solution of **[2a][CF$_3$SO$_3$]** with [N^{n}Bu$_4$]PF$_6$ (0.1 mol dm^{-3}) as supporting electrolyte. Scan rate: 0.1 V s^{-1}

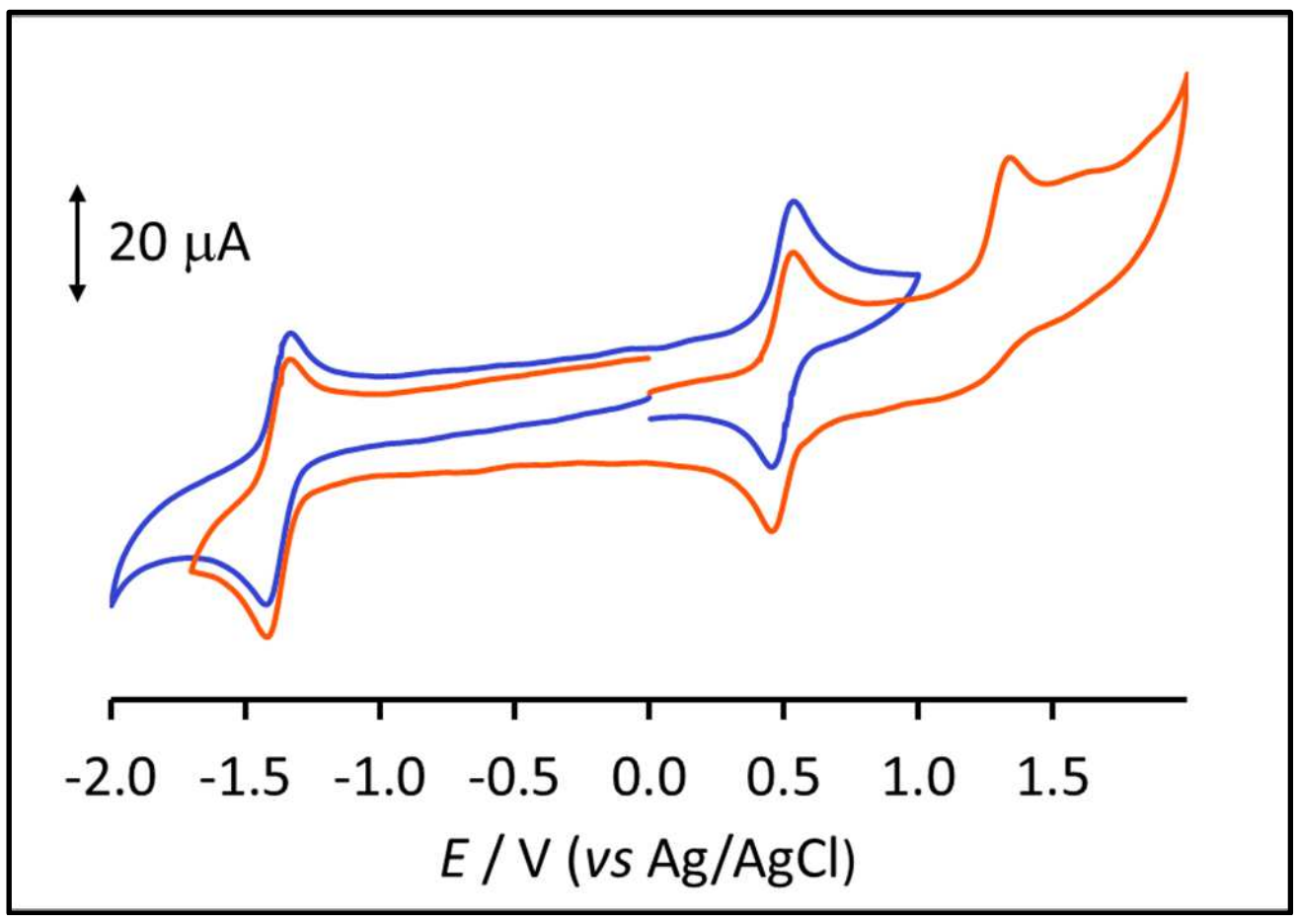

Figure S36. Cyclic voltammogram recorded at a Pt electrode in a CH_3CN solution of **[3a][CF$_3$SO$_3$]$_2$** with [N^{n}Bu$_4$]PF$_6$ (0.1 mol dm^{-3}) as supporting electrolyte. Scan rate: 0.1 V s^{-1}

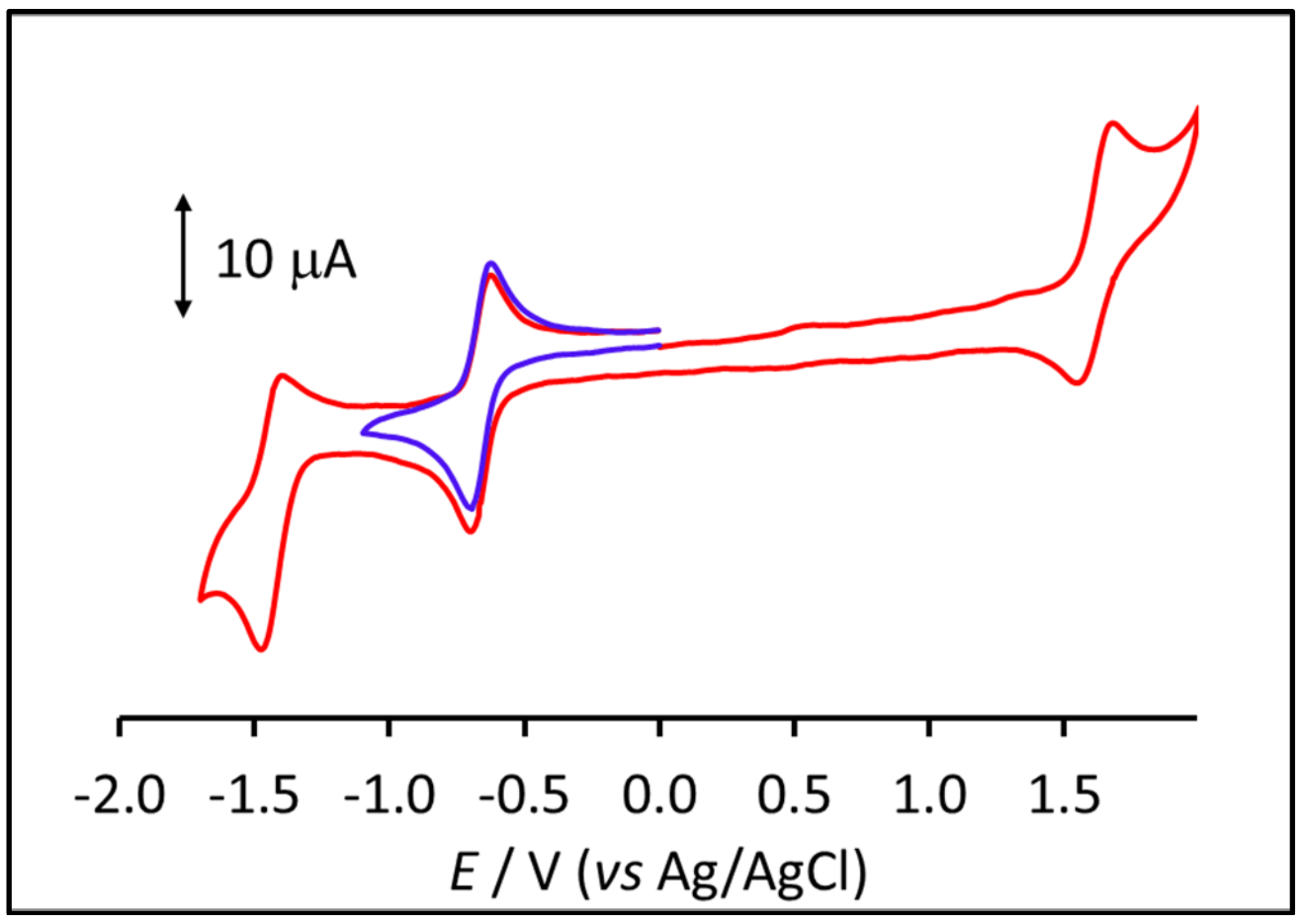

Figure S37. Top: IR spectral changes of a CH_3CN solution of **[2a][CF₃SO₃]** recorded in an OTTLE cell during the progressive decrease of the potential from −1.20 to −1.40 V *vs* Ag pseudoreference electrode (scan rate 1 mV sec^{-1}). Bottom: IR spectra recorded before (black) and after (red) a slow cyclic voltammetry between −1.20 and −1.40 V. The solvent and supporting electrolyte ($[N^nBu_4]PF_6$ 0.1 mol dm^{-3}) absorptions have been subtracted.

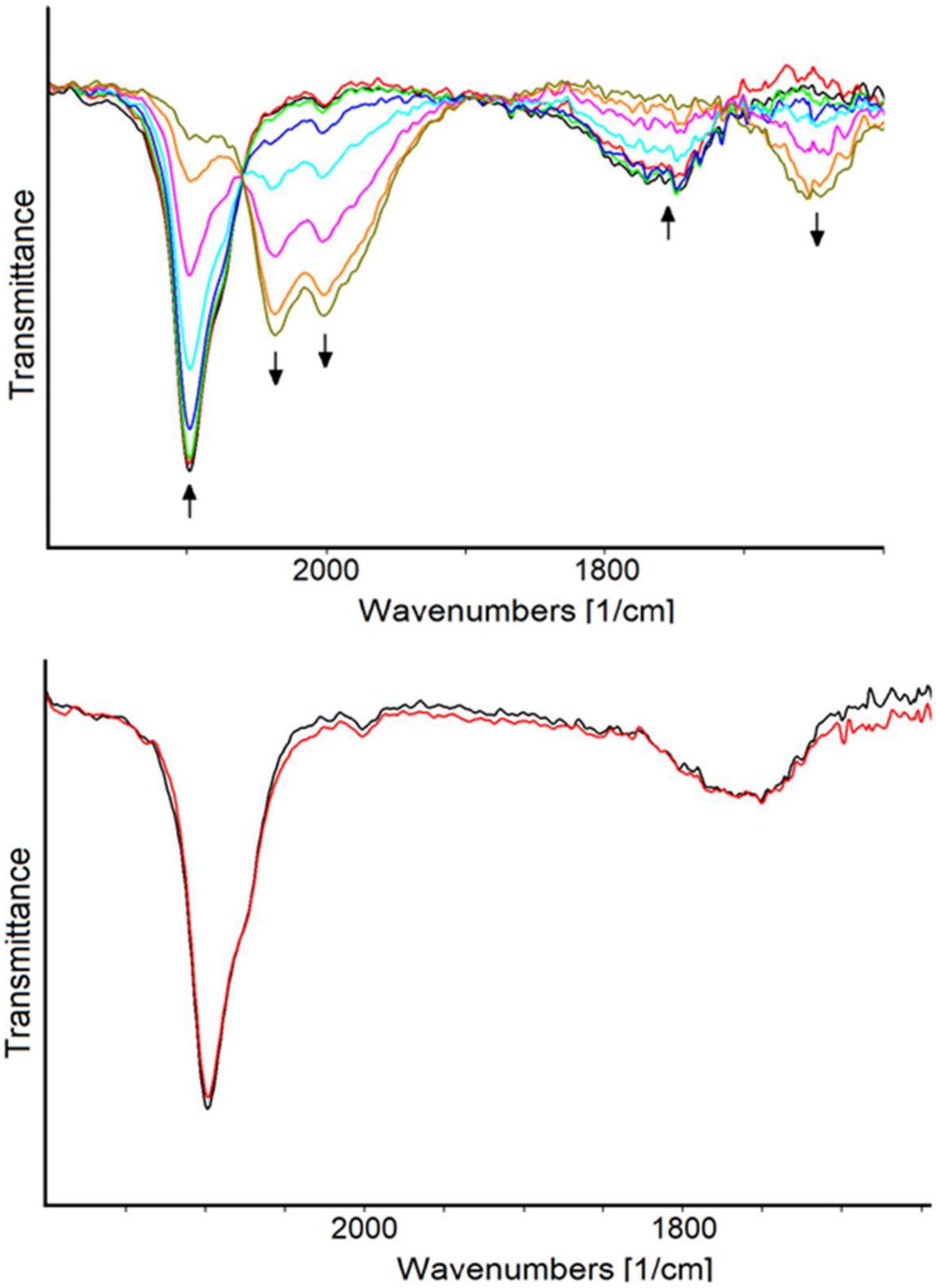

Figure S38. Top: IR spectral changes of a CH_3CN solution of **[2a][CF$_3$SO$_3$]** recorded in an OTTLE cell during the progressive increase of the potential from +0.50 to +0.75 V *vs* Ag pseudoreference electrode (scan rate 1 mV sec^{-1}). Bottom: IR spectra recorded before (black) and after (red) a slow cyclic voltammetry between +0.50 and +0.75 V. The solvent and supporting electrolyte ([N^{n}Bu$_4$]PF$_6$ 0.1 mol dm^{-3}) absorptions have been subtracted.

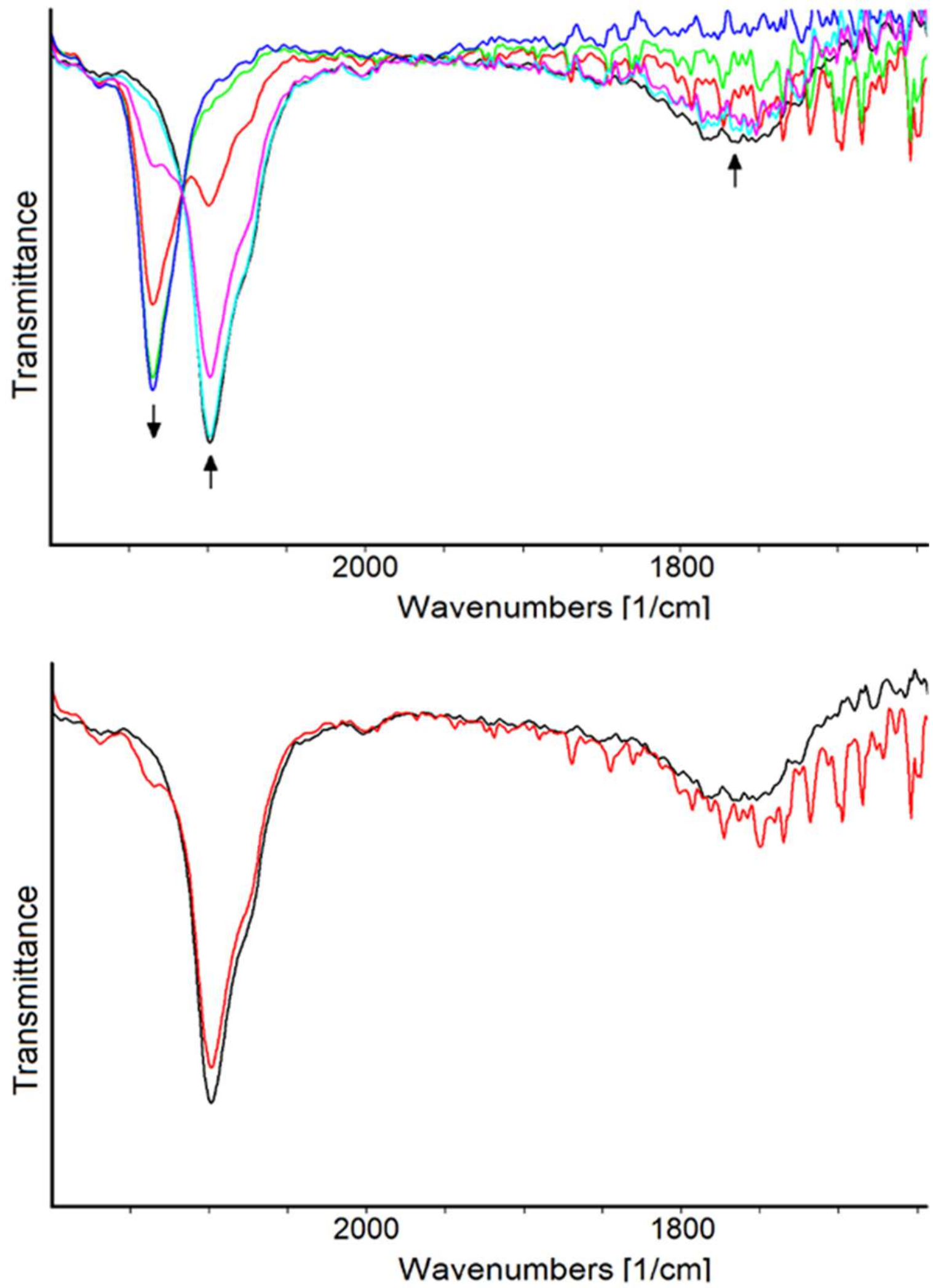

Figure S39. IR spectral changes of a CH_3CN solution of **3a**$^{2+}$ recorded in an OTTLE cell during the progressive decrease of the potential (Top) from: −0.55 to −0.80 V and (Middle) from −0.95 to −1.50 V *vs* Ag pseudoreference electrode (scan rate 1 mV sec^{-1}). Bottom: IR spectra recorded before (black) and after (red) a slow cyclic voltammetry between −0.55 and −1.50 V. The solvent and supporting electrolyte ($[N^nBu_4]PF_6$ 0.1 mol dm^{-3}) absorptions have been subtracted.

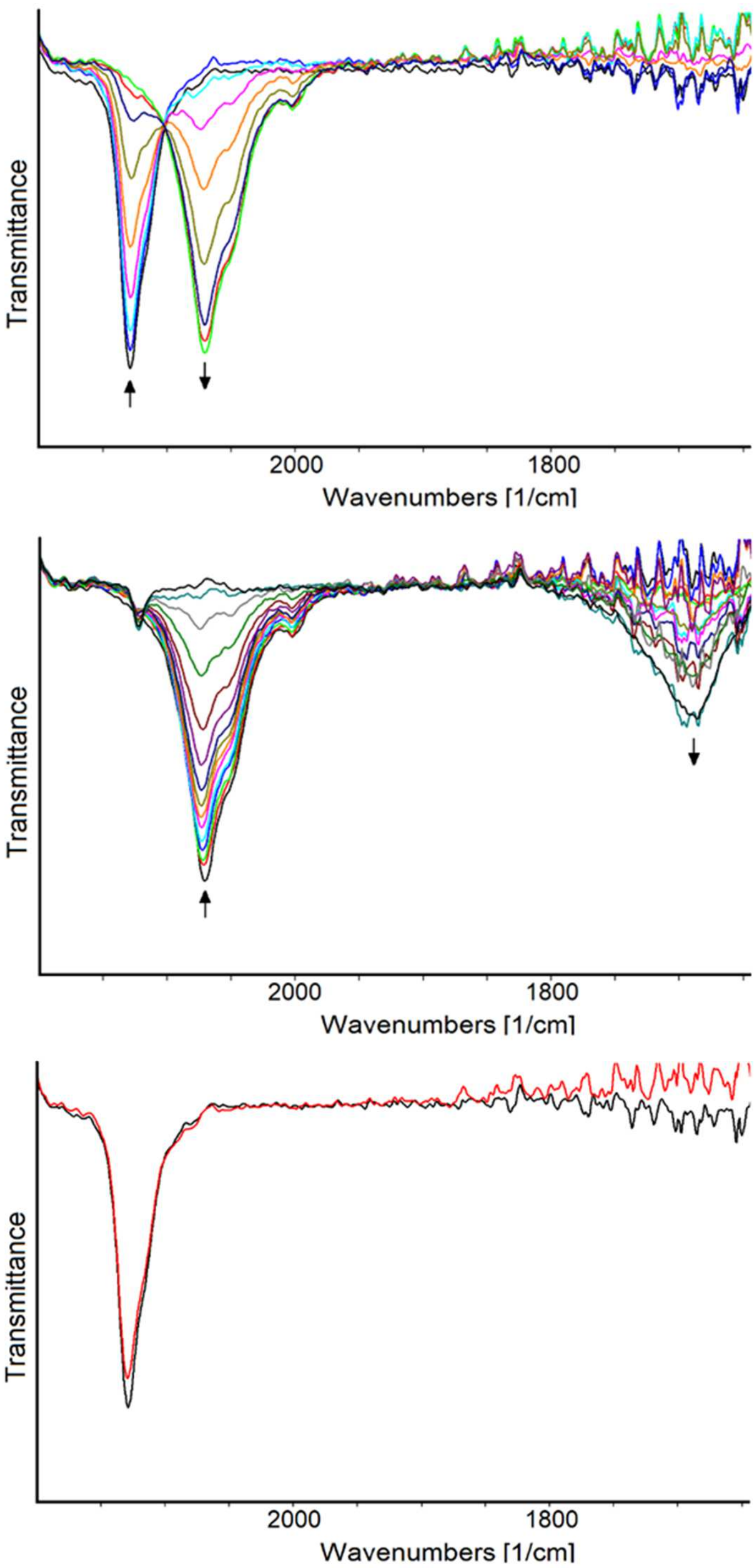

Figure S40. Top: IR spectral changes of a CH_3CN solution of [**3a**][CF_3SO_3]$_2$ recorded in an OTTLE cell during the progressive increase of the potential from +1.55 to +1.95 V *vs* Ag pseudoreference electrode (scan rate 1 mV sec^{-1}). Bottom: IR spectra recorded before (black) and after (red) a slow cyclic voltammetry between +1.55 and +1.95 V. The solvent and supporting electrolyte ([N^nBu_4]PF_6 0.1 mol dm^{-3}) absorptions have been subtracted.

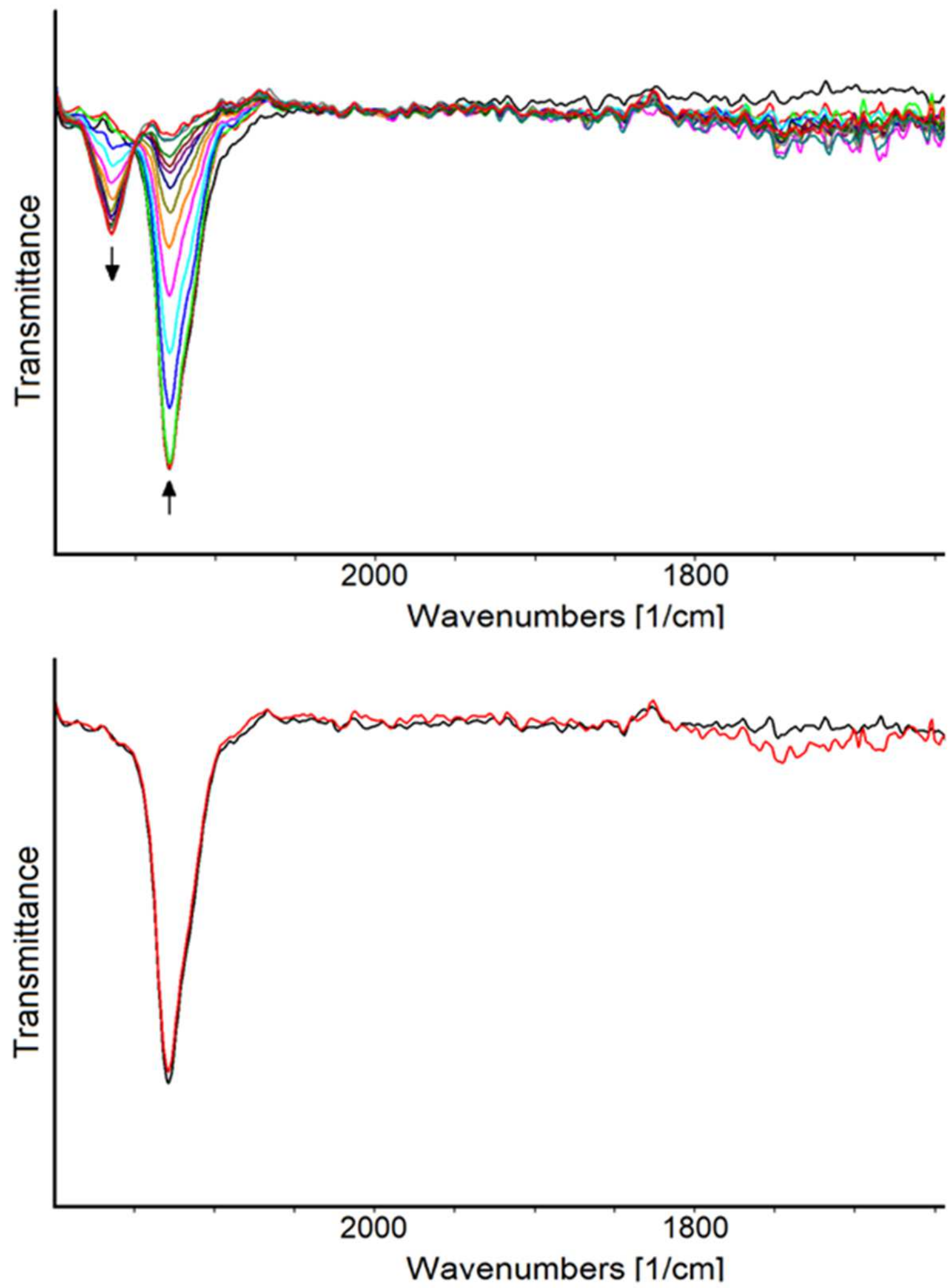

Table S8. ^{1}H NMR data for diiron complexes in aqueous solutions. Chemical shifts are referred to Me_2SO_2 (δ/ppm = 3.10).

**Complex**	**^{1}H NMR (D_2O), δ/ppm**
**[3a][CF_3SO_3]$_2$**	7.54-7.06 (m, 12H, arom-CH); 5.77, *5.41*, 5.16, *4.97* (s, 10H, Cp); 4.58, *4.43* (s, 6H, NMe); 2.92, 2.72, 2.44, *2.11*, *2.04*, *1.90* (s, 24H, Me^{Xyl})
**[3b][CF_3SO_3]$_2$**	5.30, *5.27* (s, 10H, Cp); 4.04, *4.01* (s, 6H, NMe); 3.66 (m, 2H, CH^{Cy}); 2.38-1.22 (m, 40H, CH_2^{Cy})
**[3c][CF_3SO_3]$_2$**	5.28 (s, 10H, Cp); 4.13 (s, 12H, μ-CNMe); 3.15 (s, 6H, t-CNMe)

**Complex**	**^{1}H NMR (D_2O/DMSO-d_6 3:2 v/v), δ/ppm**
**[3a][CF_3SO_3]$_2$**	*cis-anti*: 7.64-7.58, 7.51-7.46, 7.36-7.33, 7.29-7.25 (m, 12H, arom-CH); 5.20 (s, 10H, Cp); 4.47 (s, 6H, NMe); 2.76 (s, 6H), 2.65*, 2.15 (s, 12H), 2.07 (s, 6H) (Me^{Xyl}).<br>*cis-syn*: 7.51-7.46, 7.39-7.29, 7.23-7.19 (m), 7.15 (d) (12H, arom-CH); 5.86 (s, 5H), 4.60 (s, 5H) (Cp); 4.75 (s, NMe); 2.74 (s, 6H), 2.48 (s, 6H), 1.93 (s, 6H) (Me^{Xyl}). *Hidden by DMSO signal.<br>Anti/syn ratio = 1.7 (0 h), 1.3 (72 h); in D_2O/DMSO-d_6.<br>Anti/syn ratio = 1.7 (0 h), 1.3 (72 h); in DMEM-d/DMSO-d_6
**[3b][CF_3SO_3]$_2$**	*cis-anti*: 5.30 (s, 10H, Cp); 4.00 (s, 6H, Me); 3.69 (m, 2H, CH^{Cy}); 2.37 (d), 2.11-1.99, 1.95-1.85, 1.82-1.19 (m) (40 H, CH_2^{Cy}).<br>*cis-syn*: 5.32 (s, 5H), 5.27 (s, 5H) (Cp); 3.98 (s, 6H, Me); 3.60 (m, 2H, CH^{Cy}); other signals are hidden by those of the *anti* isomer.<br>Anti/syn ratio = 8.5 (0 h), 1.8 (72 h); in D_2O/DMSO-d_6.<br>Anti/syn ratio = 5.8 (0 h), 2.8 (72 h); in DMEM-d/DMSO-d_6

Figure S41. ^{1}H NMR spectrum (501 MHz, D_2O) of **[3a][CF$_3$SO$_3$]$_2$** for determination of D_2O solubility. The green box marks the aromatic signals (δ = 7.57-7.08 ppm), which were set to a total integral value of 12.0, whereas the orange box marks the Me_2SO_2 standard signal (δ = 3.14 ppm)

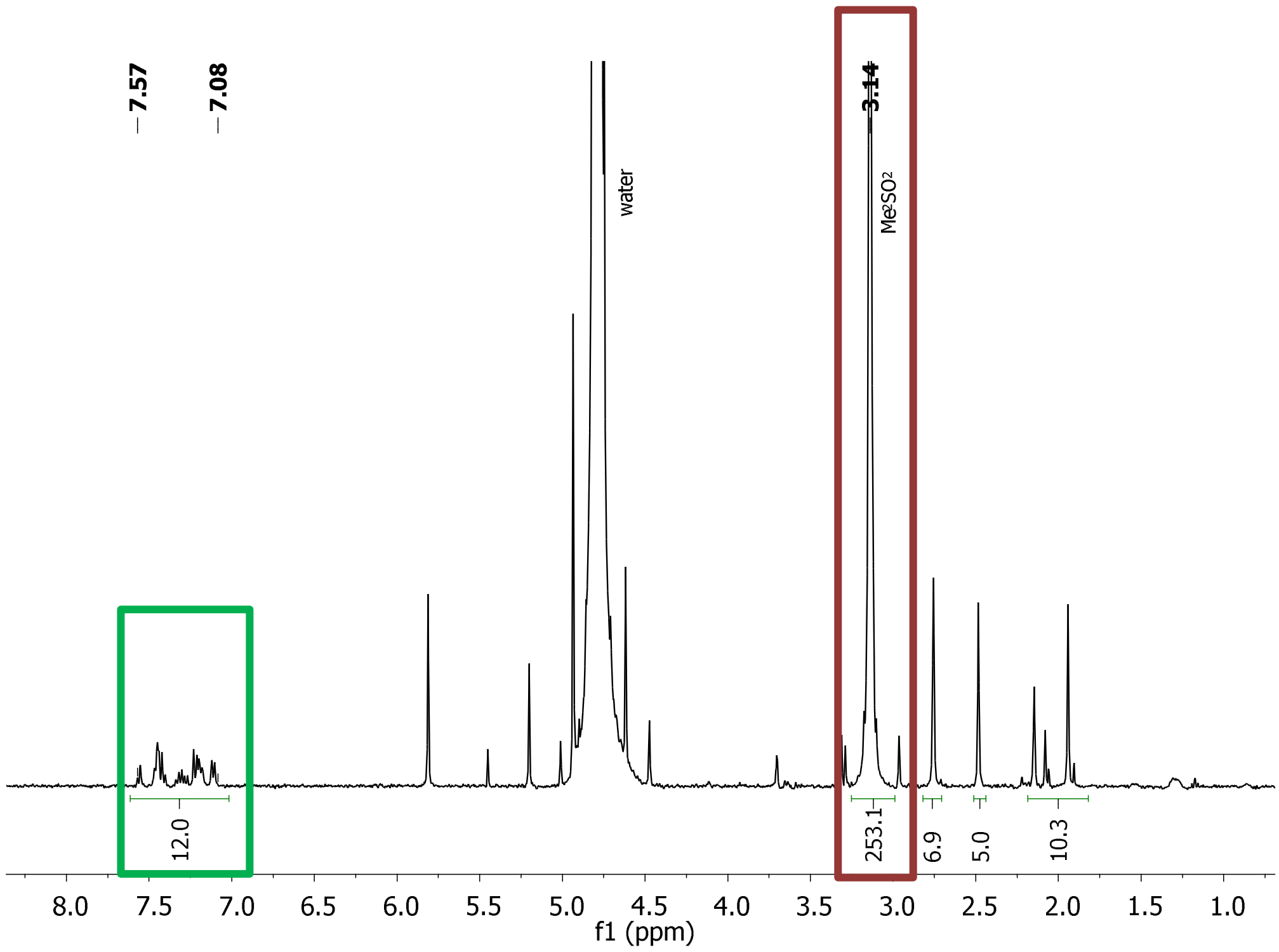

Figure S42. ^{1}H NMR spectrum (501 MHz, D_2O) of **[3b][CF$_3$SO$_3$]$_2$** for determination of D_2O solubility. The green box marks the μ-CNMe signals (δ = 4.04, 4.01 ppm), which were set to a total integral value of 6.0, whereas the orange box marks the Me_2SO_2 standard signal (δ = 3.14 ppm)

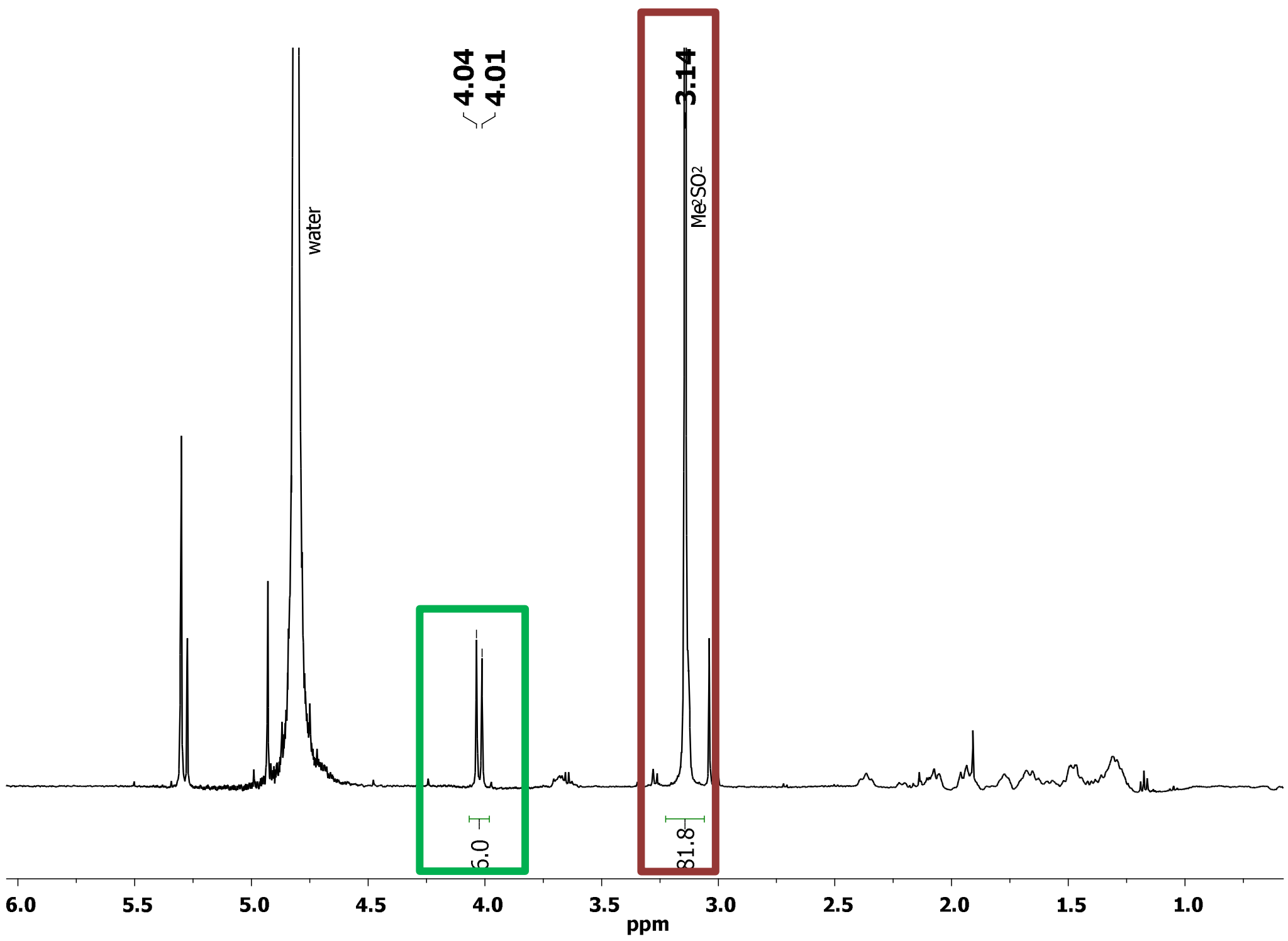

Figure S43. ^{1}H NMR spectrum (501 MHz, D_2O) of **[3c][CF$_3$SO$_3$]$_2$** for determination of D_2O solubility. The green box marks the μ-CNMe signal (δ = 4.13 ppm), which was set to an integral value of 12.0, whereas the orange box marks the TMSP-d$_4$ standard signal (δ = 0.00 ppm)

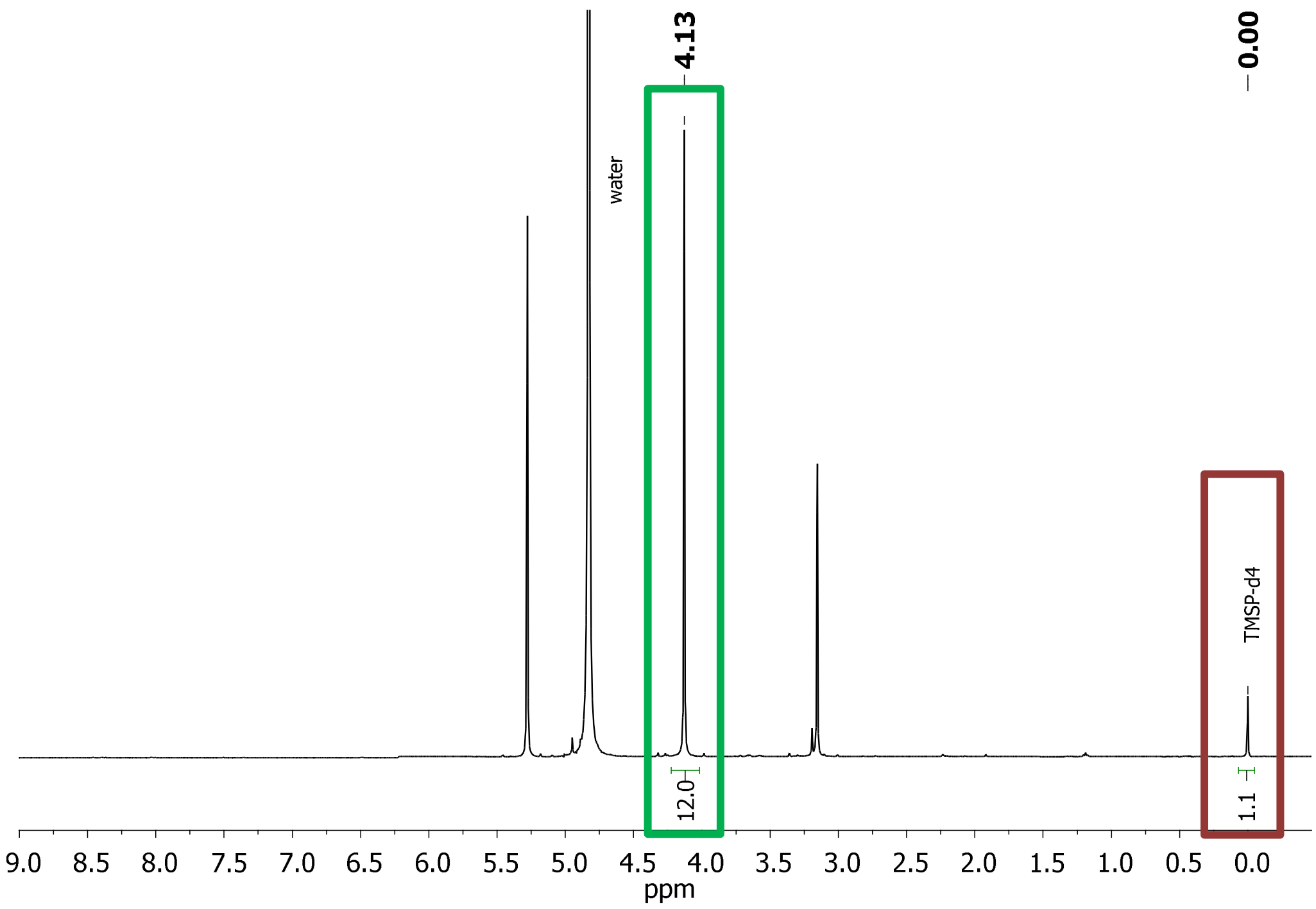

Figure S44. Comparative ^{1}H NMR spectra of **[3a][CF$_3$SO$_3$]$_2$** (501 MHz, D_2O/DMSO-d_6 3:2 *v/v*). **A)** Spectrum recorded at 0 h; **B)** Spectrum recorded after 72 h incubation at 37 °C. The green box marks the aromatic signals (δ = 7.64-7.25 ppm), set to a total integral value of 12.0, whereas the orange box marks the TMSP-d_4 standard signal (δ = 0.0 ppm). The stability of **[3a]$^{2+}$** was estimated by comparing the integrals of the standard signal at 0 h and 72 h.

A)

B)

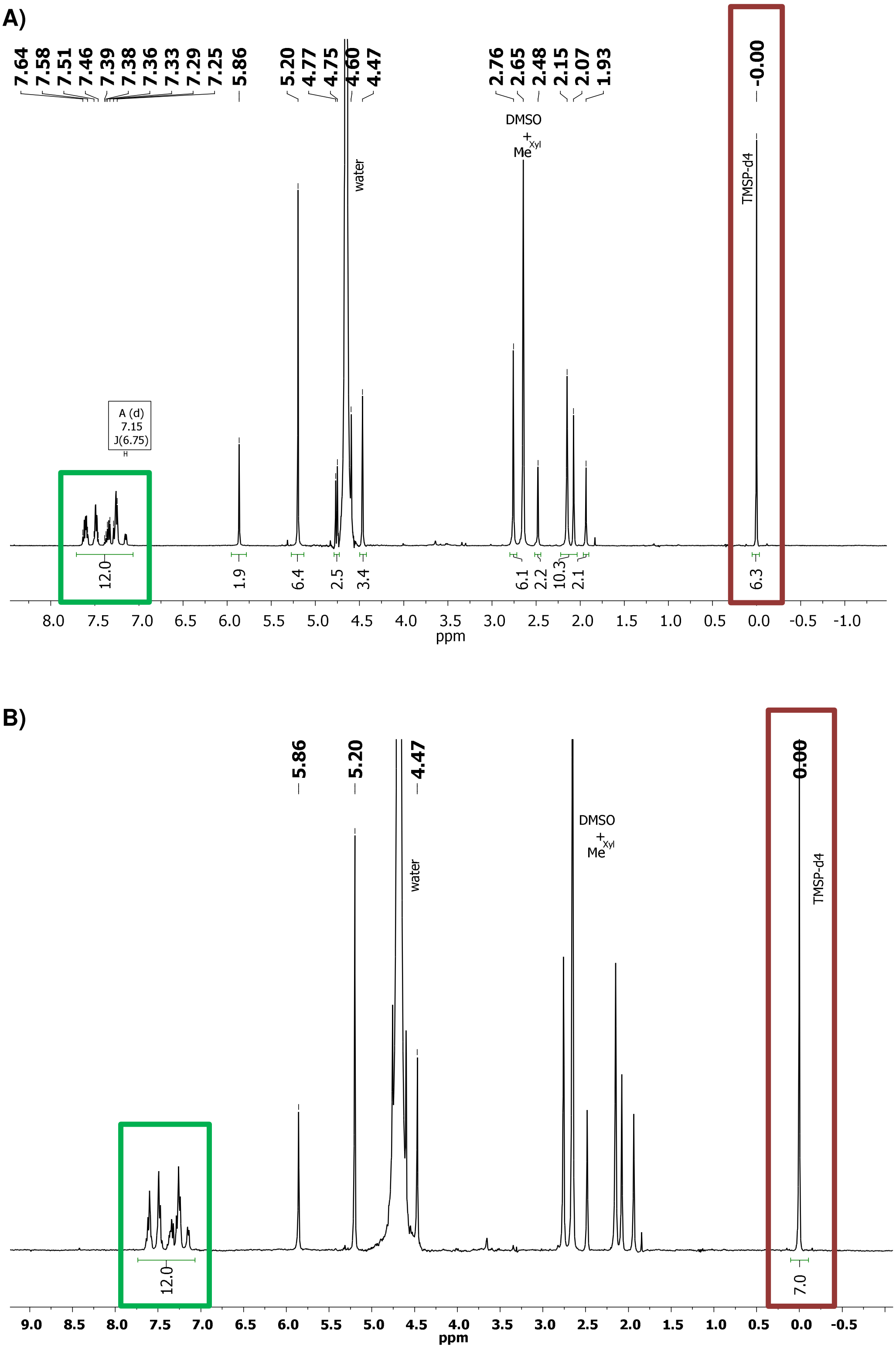

Figure S45. Comparative ^{1}H NMR spectra of **[3a][CF$_3$SO$_3$]$_2$** (501 MHz, DMEM-d/DMSO-d$_6$ 3:2 *v/v*). **A)** Spectrum recorded at 0 h; **B)** Spectrum recorded after 72 h incubation at 37 °C. The green box marks the Cp signal (δ = 5.87, 5.20, 4.47 ppm), set to an integral value of 10.0, whereas the orange box marks the TMSP-d$_4$ standard signal (δ = 0.0 ppm). The stability of **[3a]$^{2+}$** was estimated by comparing the integrals of the standard signal at 0 h and 72 h.

A)

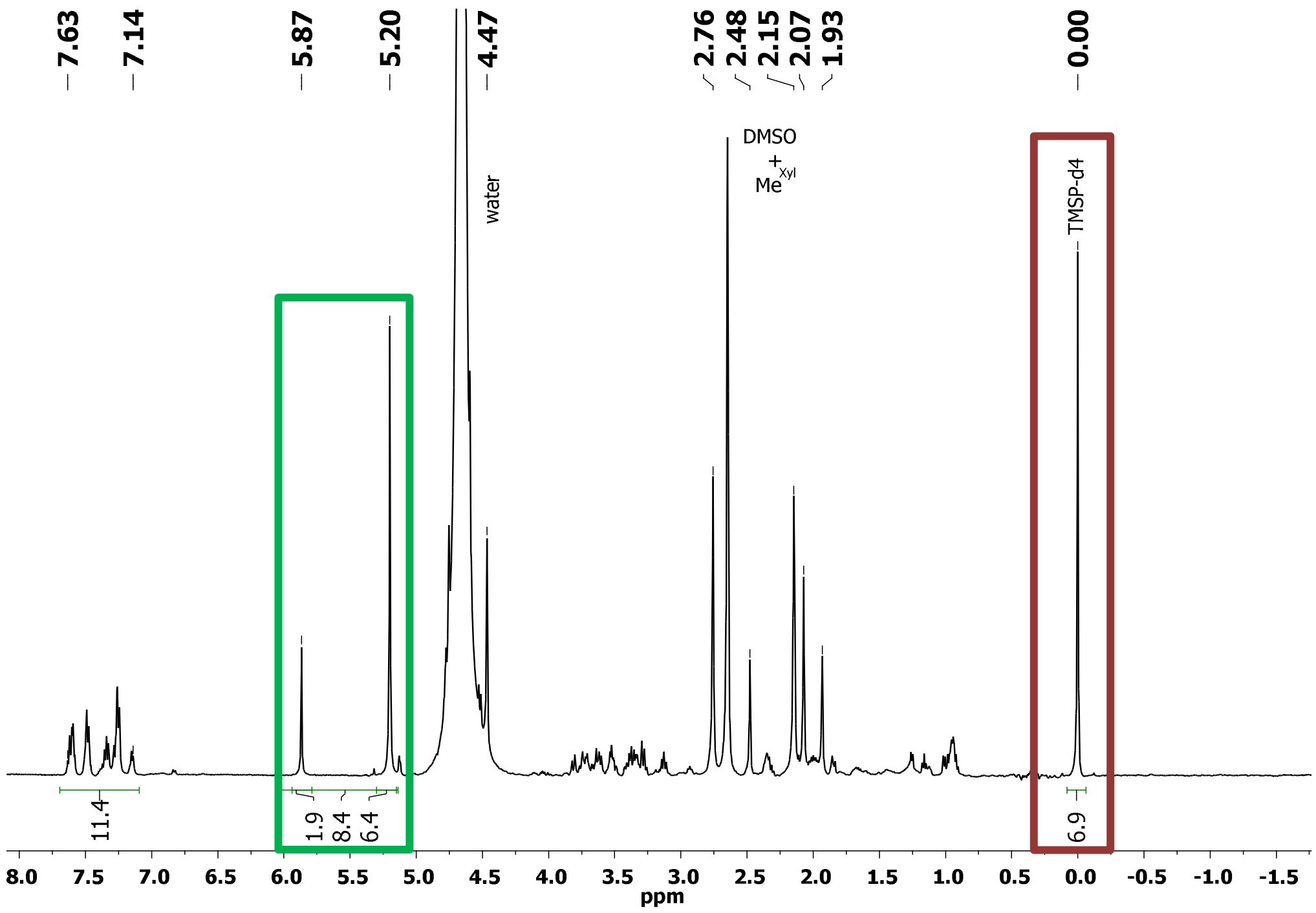

B)

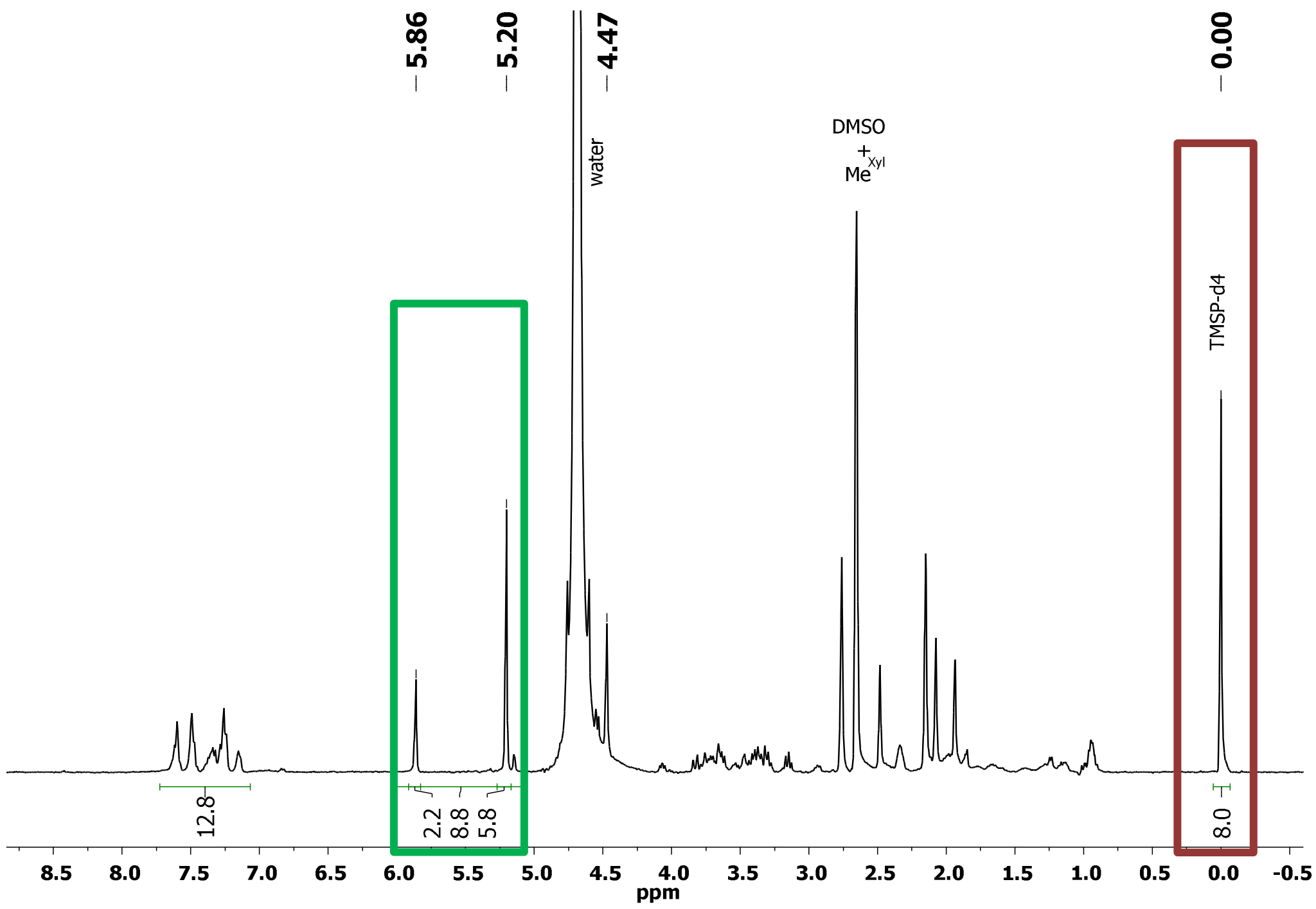

Figure S46. Comparative ^{1}H NMR spectra of **[3b][CF3SO3]2** (501 MHz, D_2O/DMSO-d_6 3:2 *v/v*). **A)** Spectrum recorded at 0 h; **B)** Spectrum recorded after 72 h incubation at 37 °C. The green box marks the Me signals (δ = 4.00, 3.98 ppm), set to a total integral value of 6.0, whereas the orange box marks the Me_2SO_2 standard signal (δ = 3.10 ppm). The stability of **[3b]$^{2+}$** was estimated by comparing the integrals of the standard signal at 0 h and 72 h.

A)

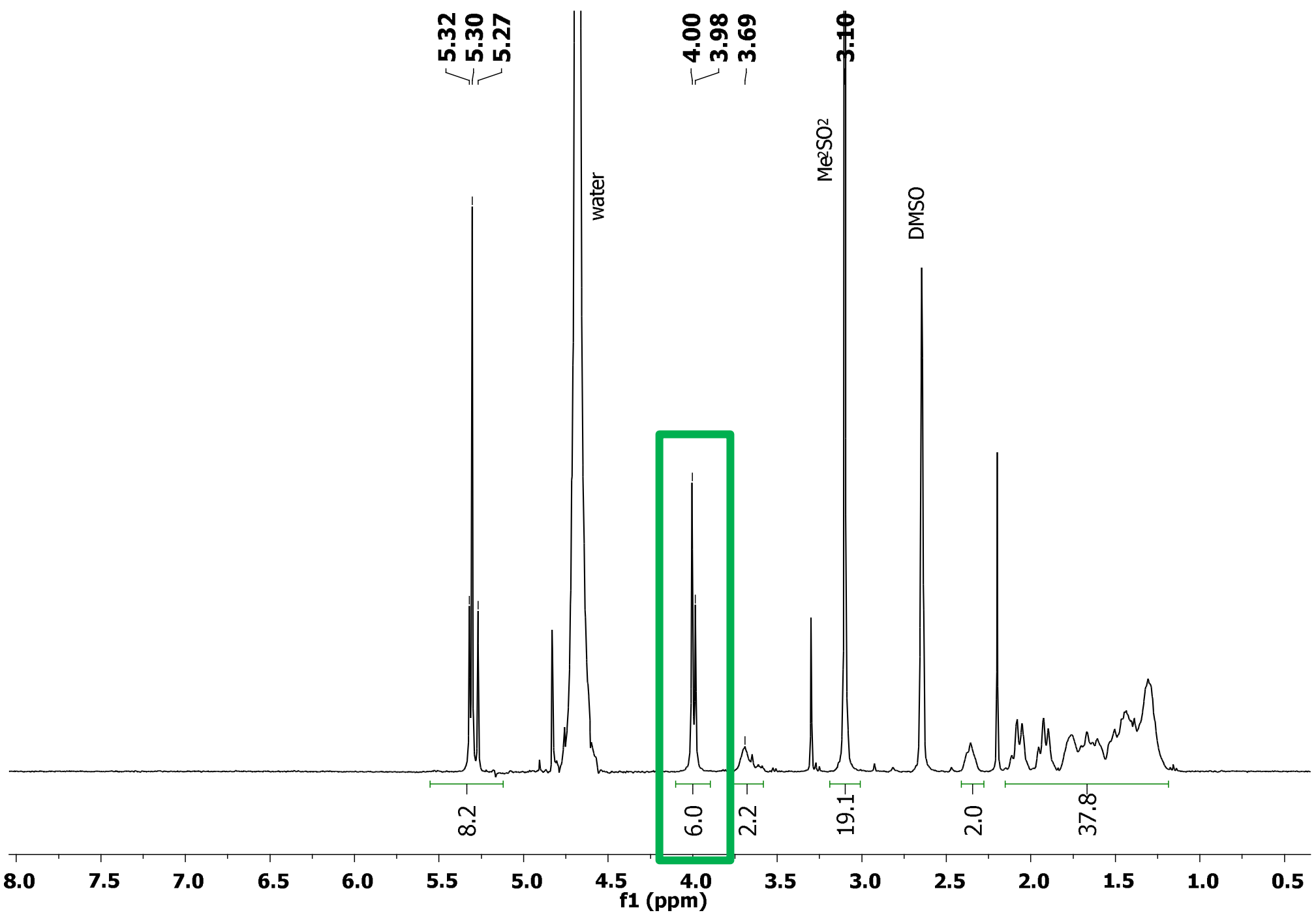

B)

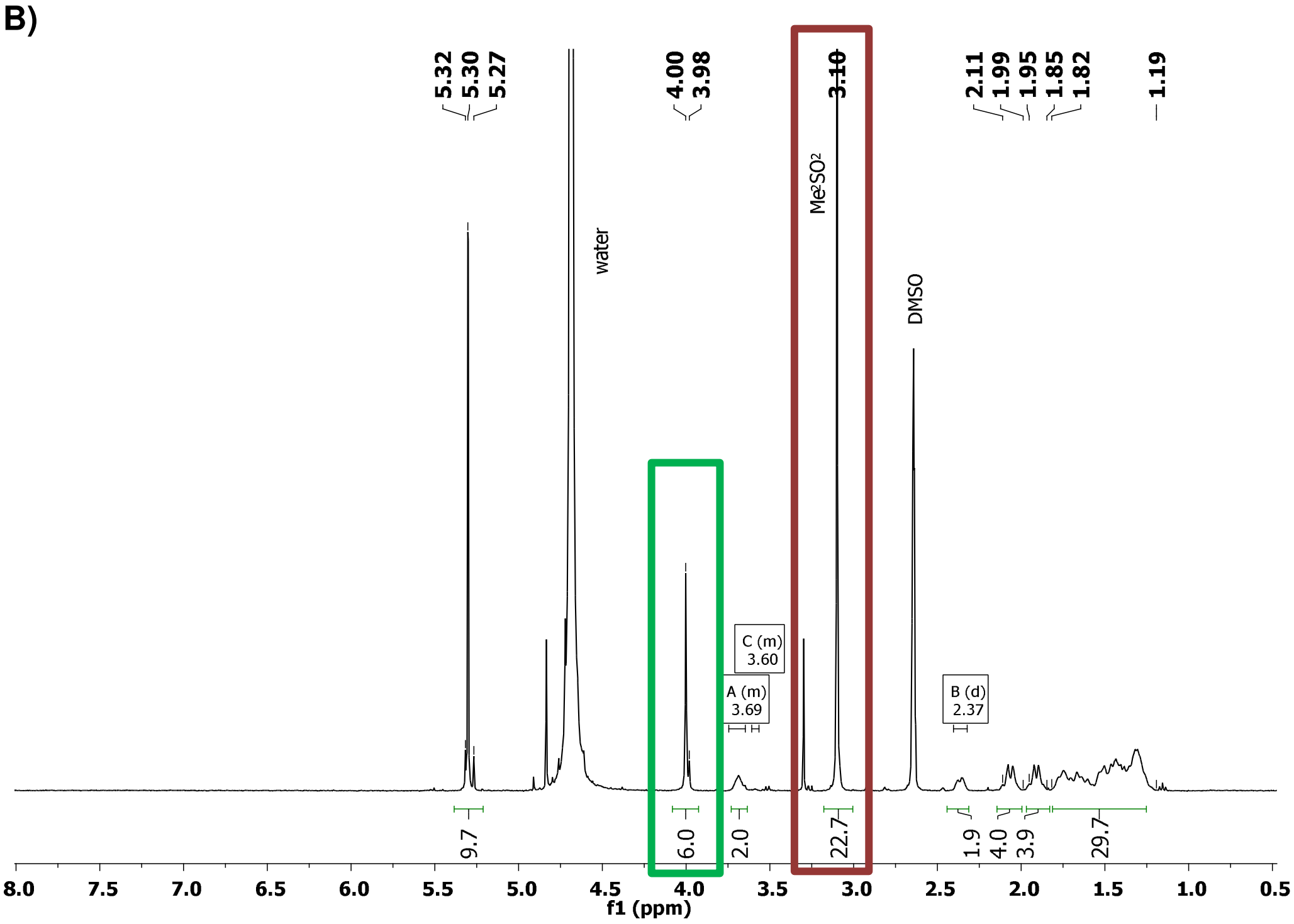

Figure S47. Comparative ^{1}H NMR spectra of **[3b][CF$_3$SO$_3$]$_2$** (501 MHz, DMEM/DMSO-d_6 3:2 *v/v*). **A)** Spectrum recorded at 0 h; **B)** Spectrum recorded after 72 h incubation at 37 °C. The green box marks the Cp signals (δ = 5.33, 5.31, 5.27 ppm), set to a total integral value of 10.0, whereas the orange box marks the Me_2SO_2 standard signal (δ = 3.10 ppm). The stability of **[3b]$^{2+}$** was estimated by comparing the integrals of the standard signal at 0 h and 72 h.

A)

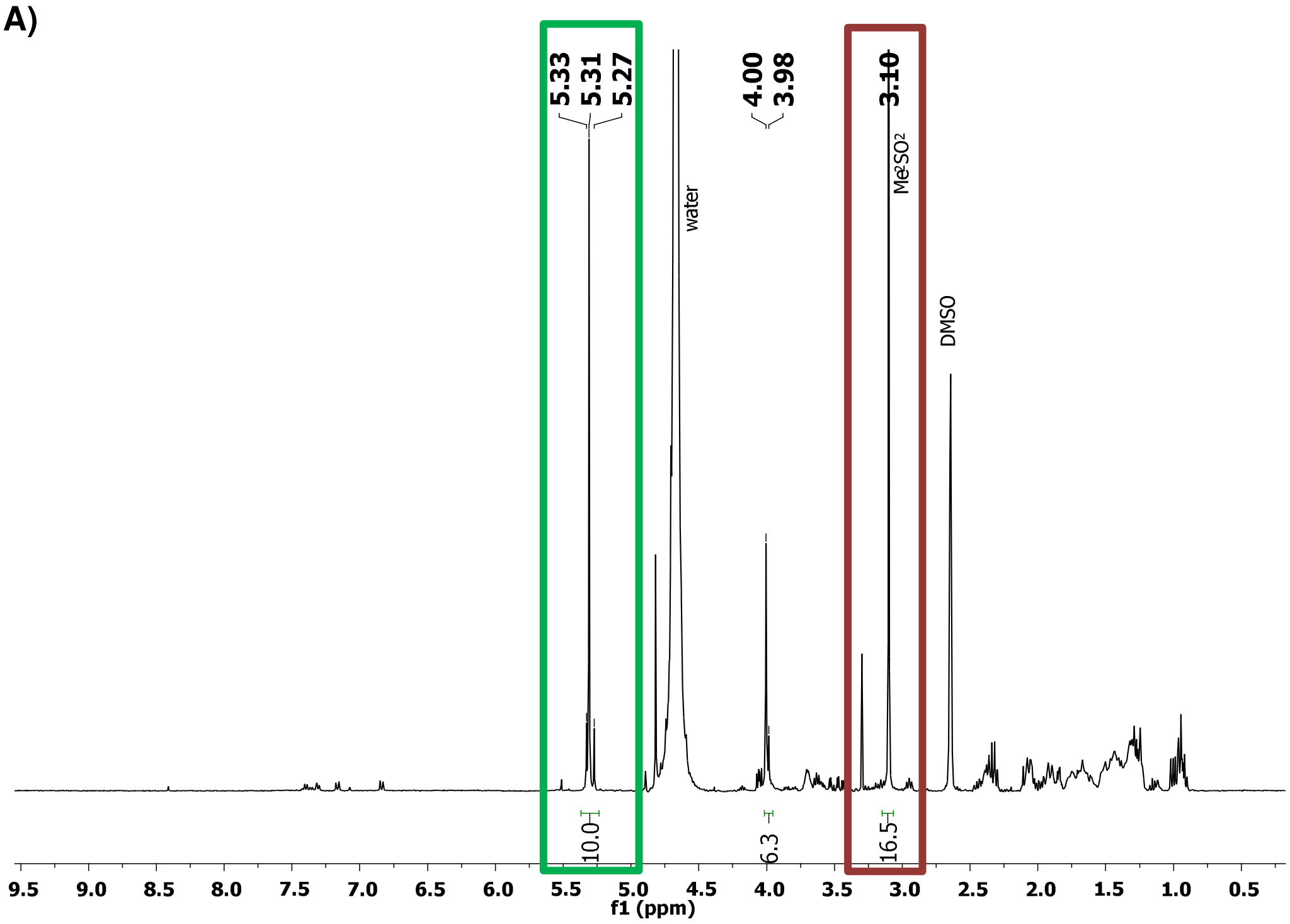

B)

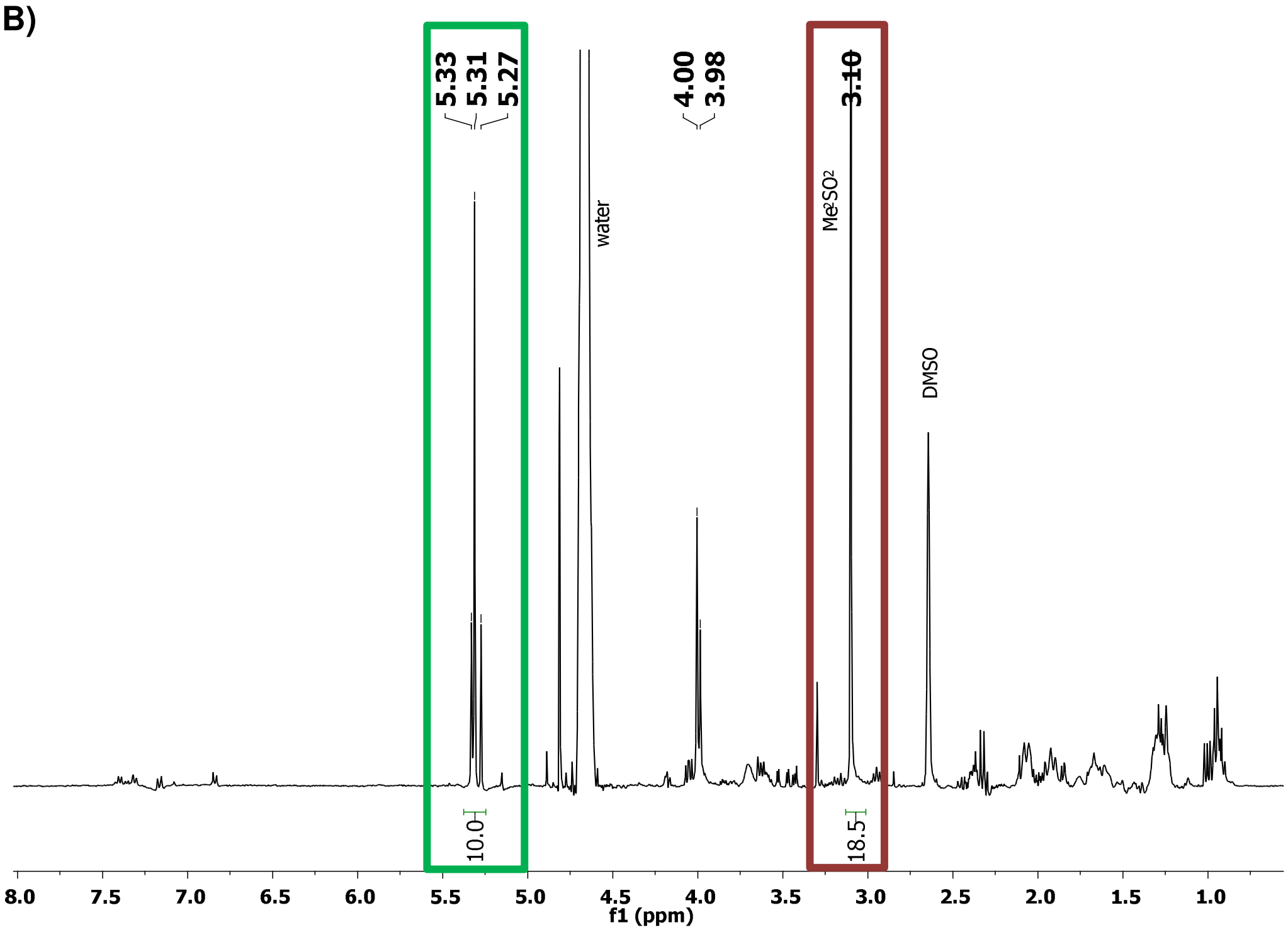

Figure S48. Comparative ^1H NMR spectra of **[3c][CF_3SO_3]$_2$** (501 MHz, D_2O). **A)** Spectrum recorded at 0 h; **B)** Spectrum recorded after 72 h incubation at 37 °C. The green box marks the Cp signal (δ = 5.28 ppm), set to an integral value of 10.0, whereas the orange box marks the Me_2SO_2 standard signal (δ = 3.15 ppm). The stability of **[3c]$^{2+}$** was estimated by comparing the integrals of the standard signal at 0 h and 72 h.

A)

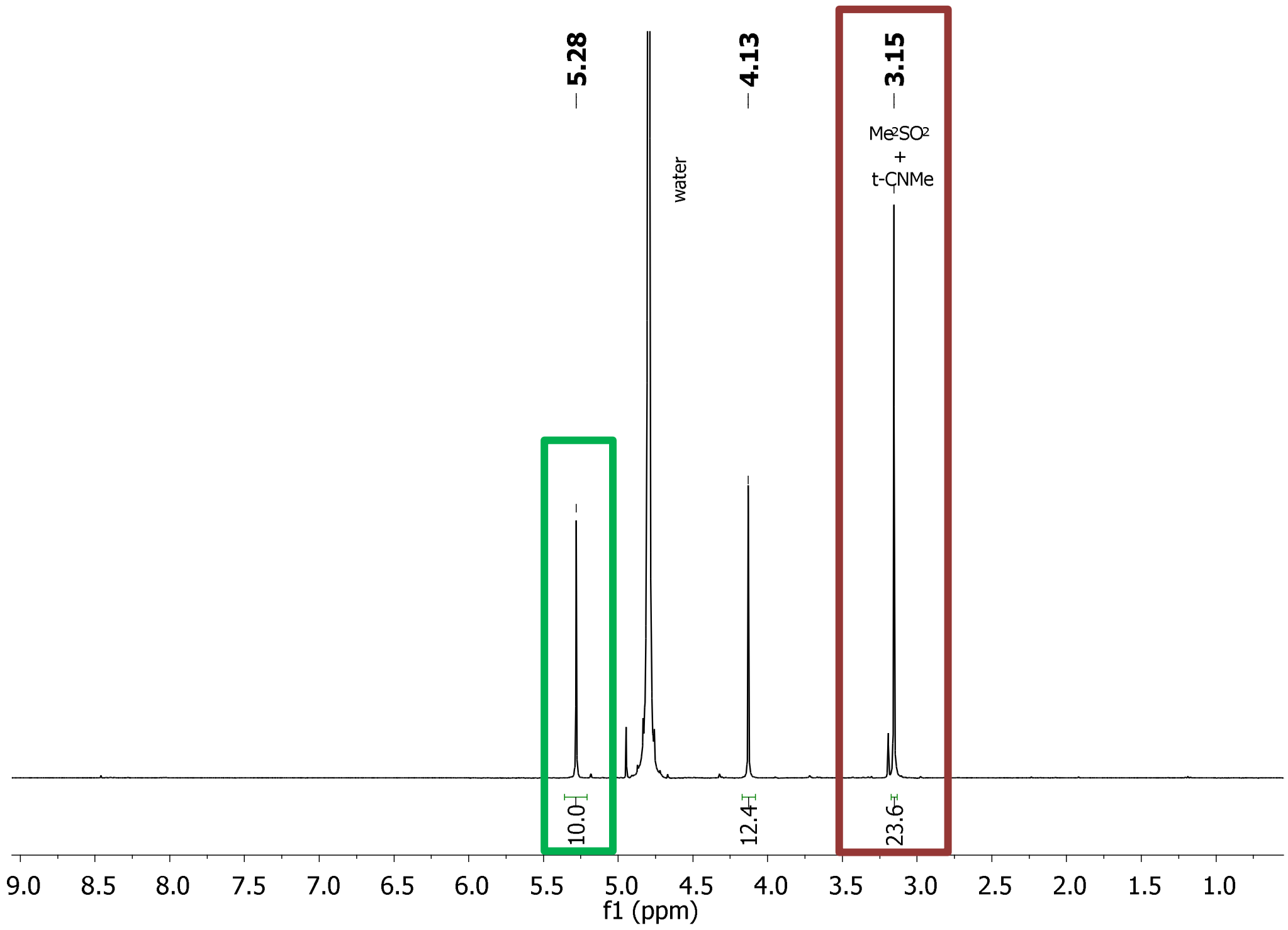

B)

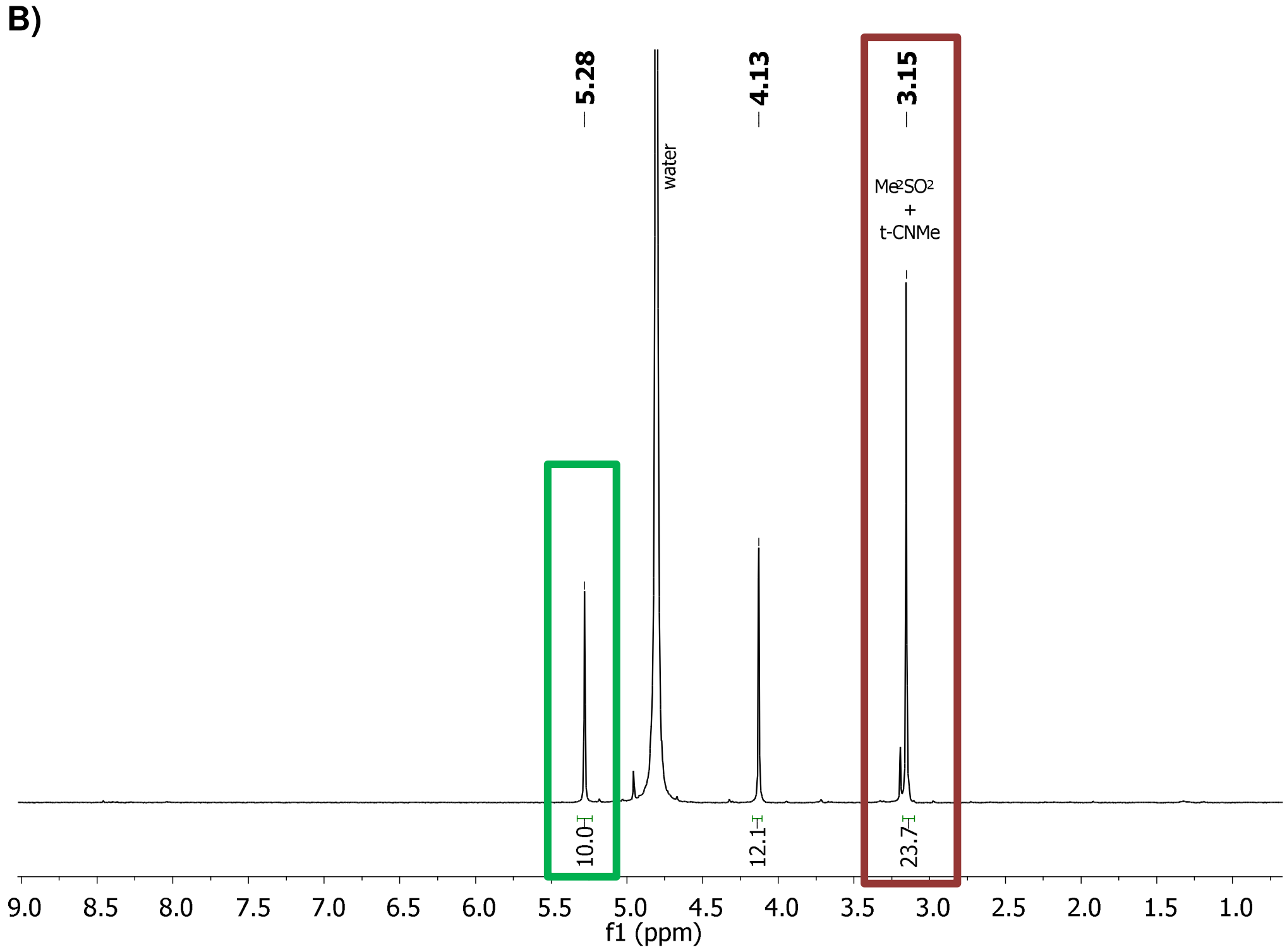

Figure S49. Comparative ¹H NMR spectra of **[3c][CF$_3$SO$_3$]$_2$** (501 MHz, DMEM-d). **A)** Spectrum recorded at 0 h; **B)** Spectrum recorded after 72 h incubation at 37 °C. The green box marks the Cp signal (δ = 5.28 ppm), set to an integral value of 10.0, whereas the orange box marks the Me_2SO_2 standard signal (δ = 3.15 ppm). The stability of **[3c]$^{2+}$** was estimated by comparing the integrals for the standard signal at 0 h and 72 h.

A)

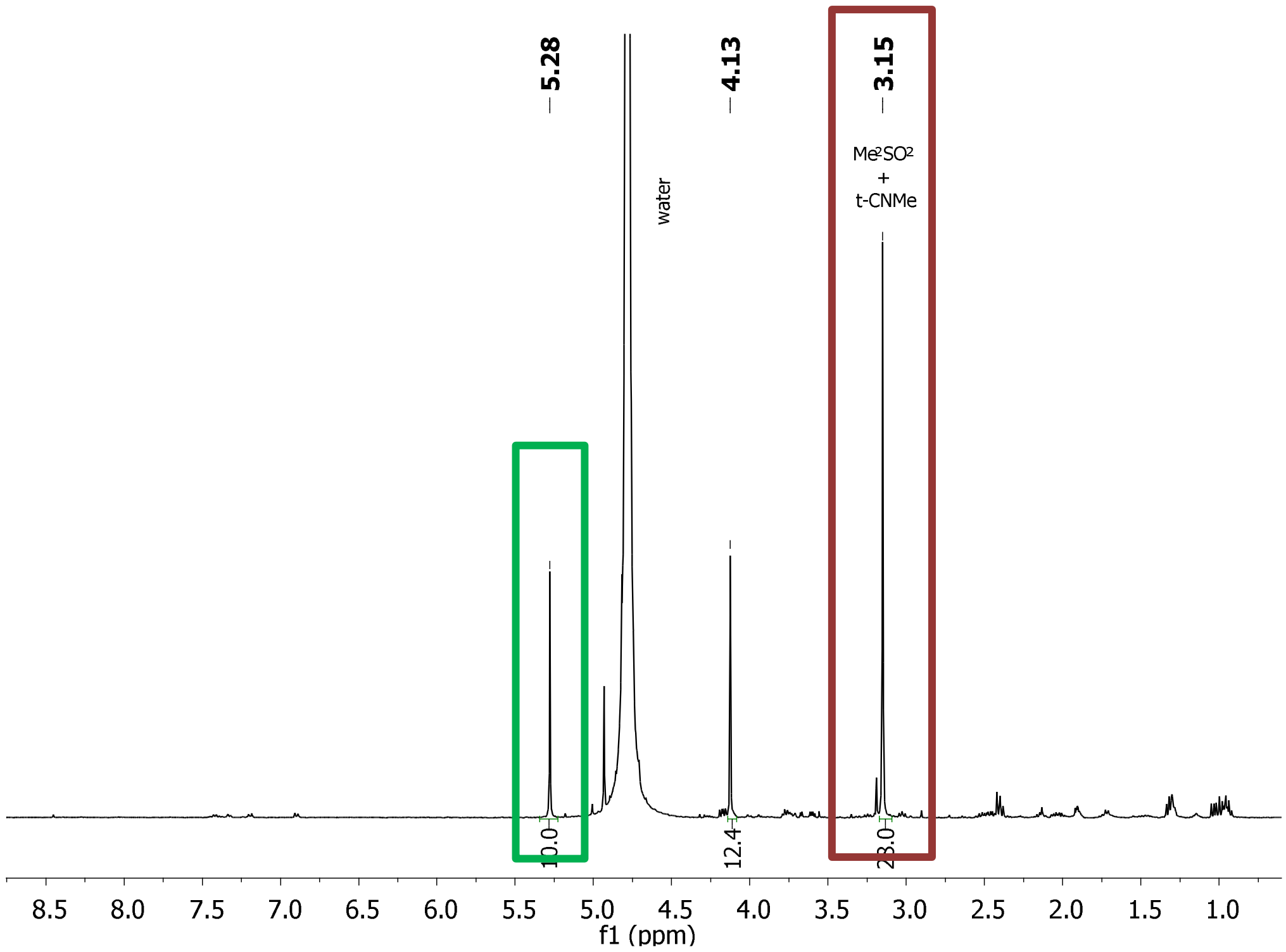

B)

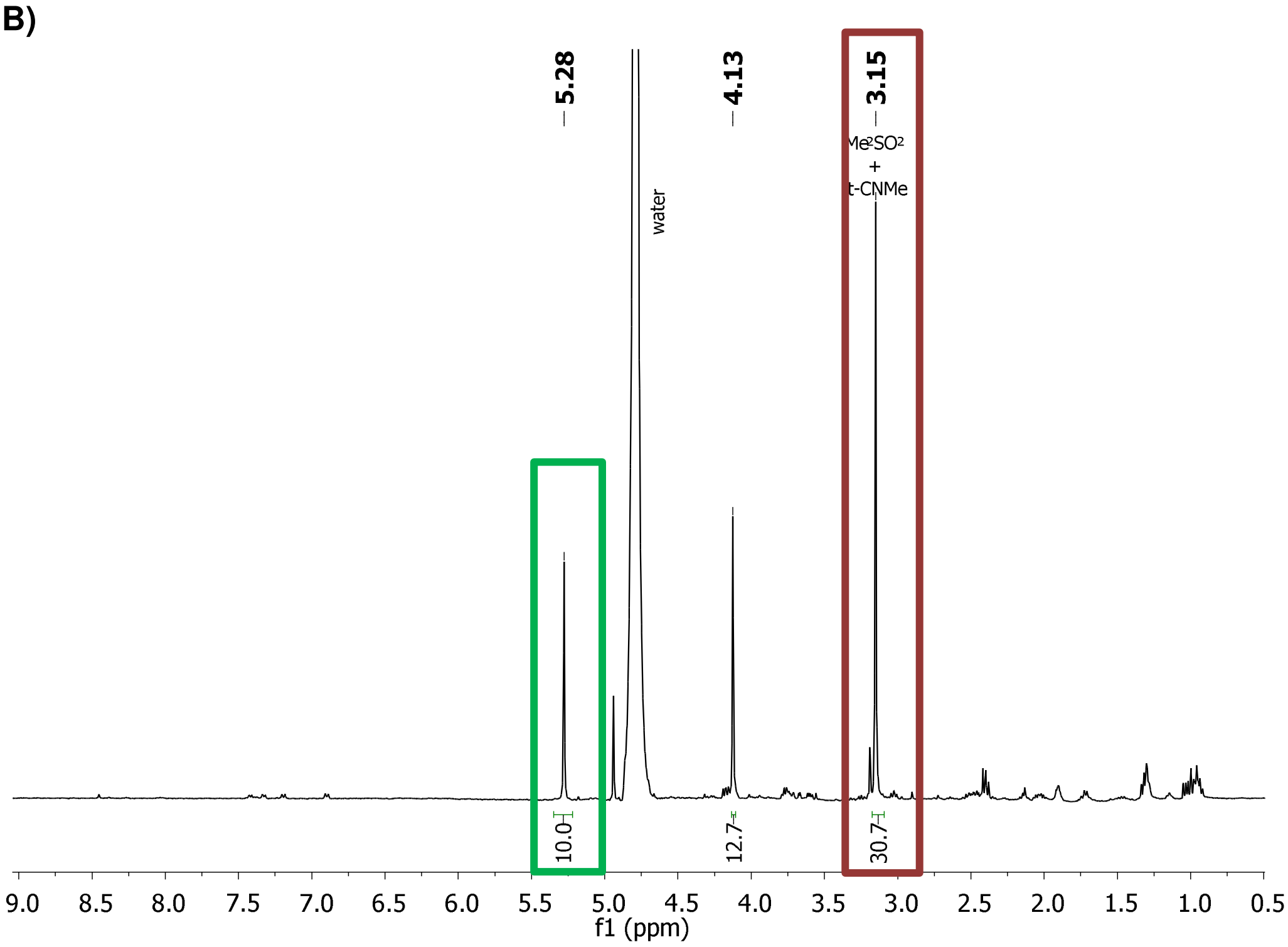

Figure S50. UV-Vis spectra of **[2a][CF_3SO_3]** in H_2O/DMSO (3:2 *v/v*) at t = 0 h (grey) and 72 h (green).

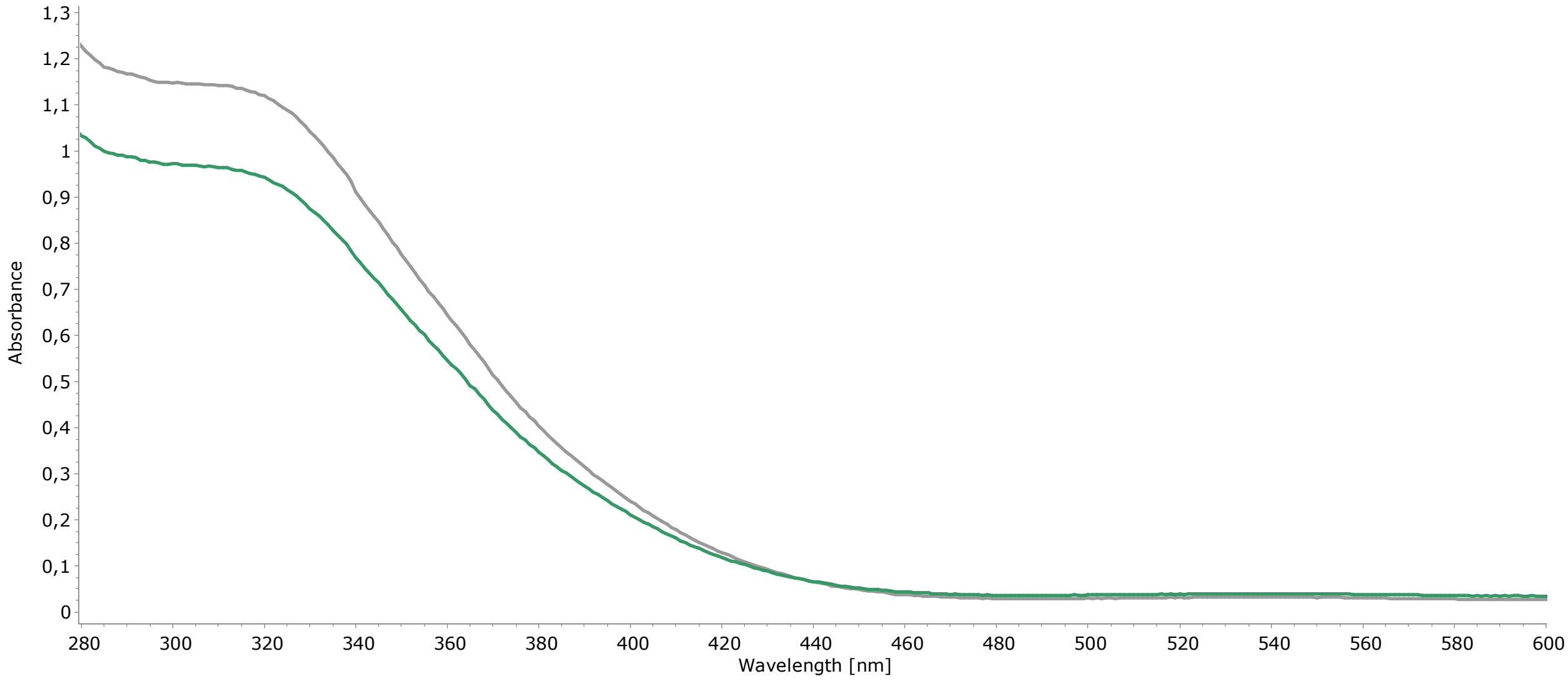

Figure S51. UV-Vis spectra of **[2b][CF_3SO_3]** in H_2O/DMSO (3:2 *v/v*) at t = 0 h (grey) and 72 h (green).

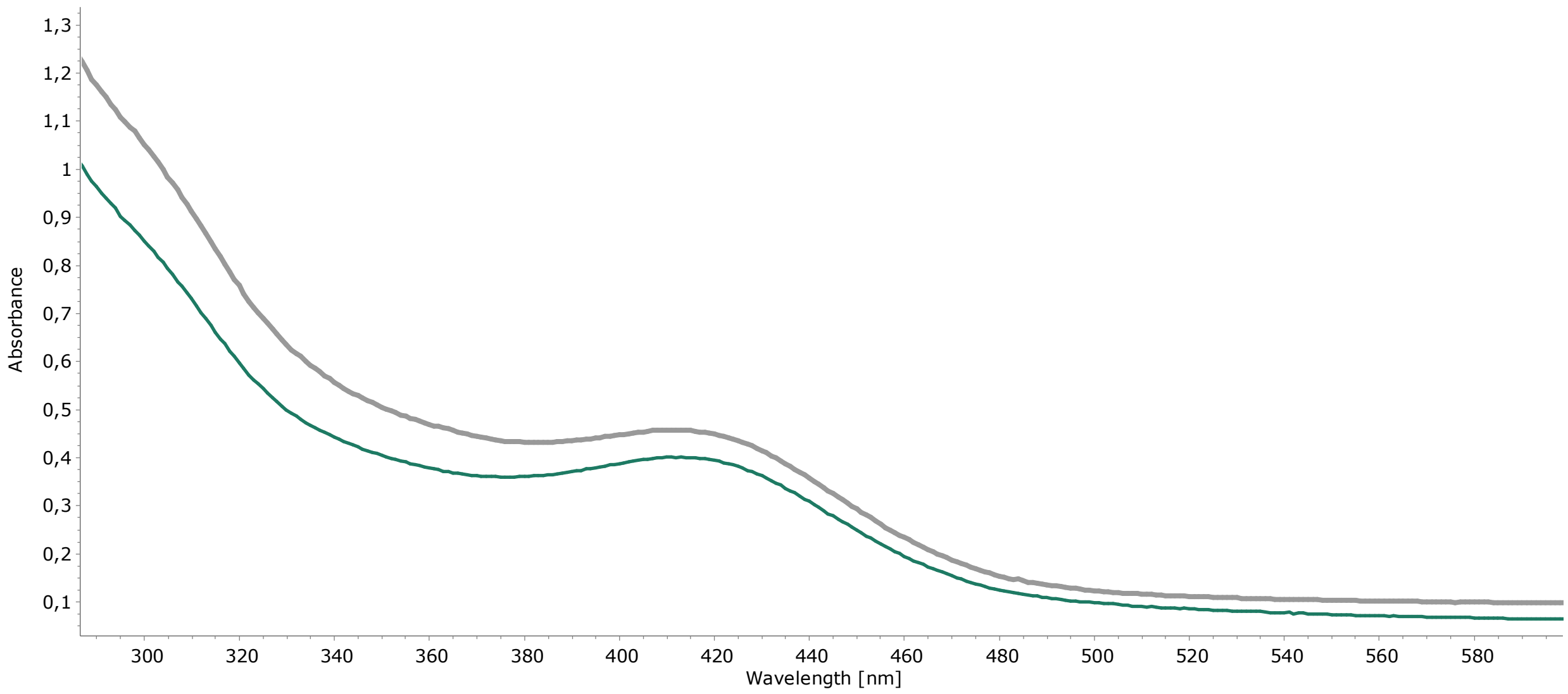

Figure S52. UV-Vis spectra of **[3a][CF_3SO_3]$_2$** in H_2O at t = 0 h (grey) and 72 h (green).

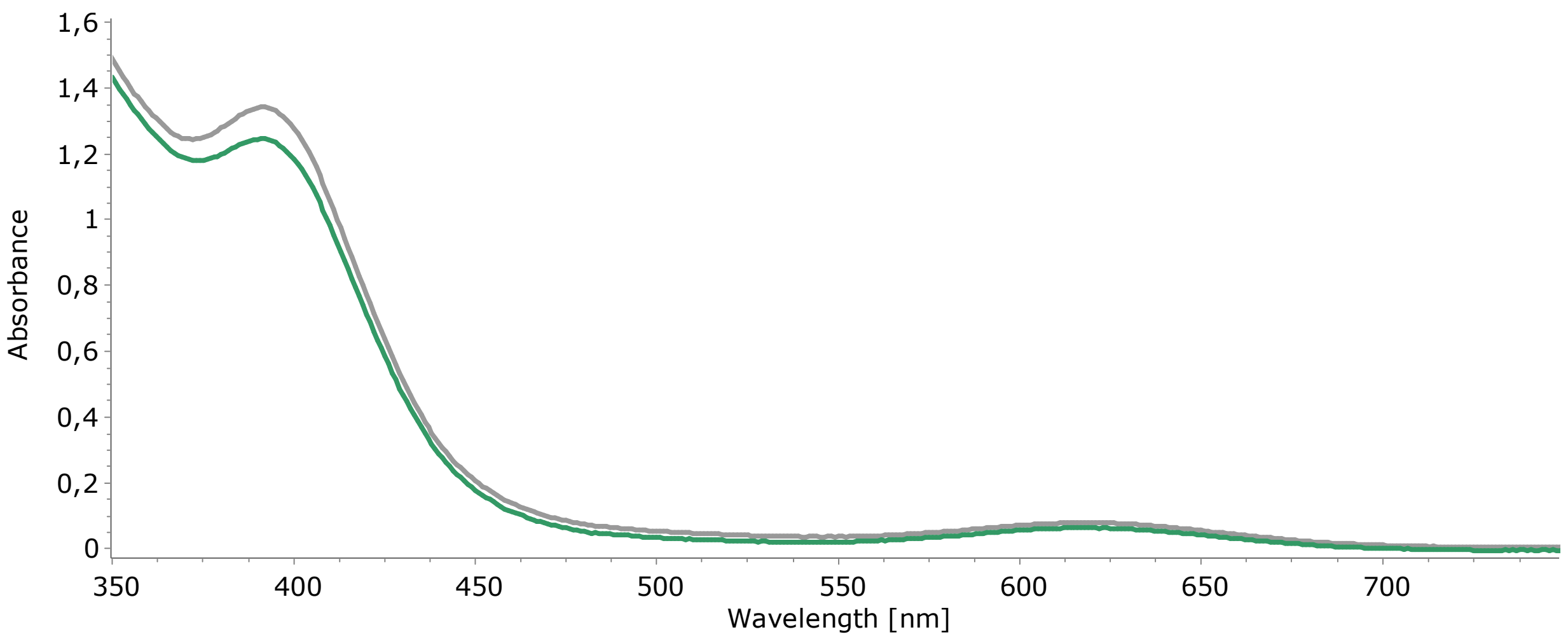

Figure S53. UV-Vis spectra of **[3b][CF_3SO_3]$_2$** in H_2O at t = 0 h (grey) and 72 h (green).

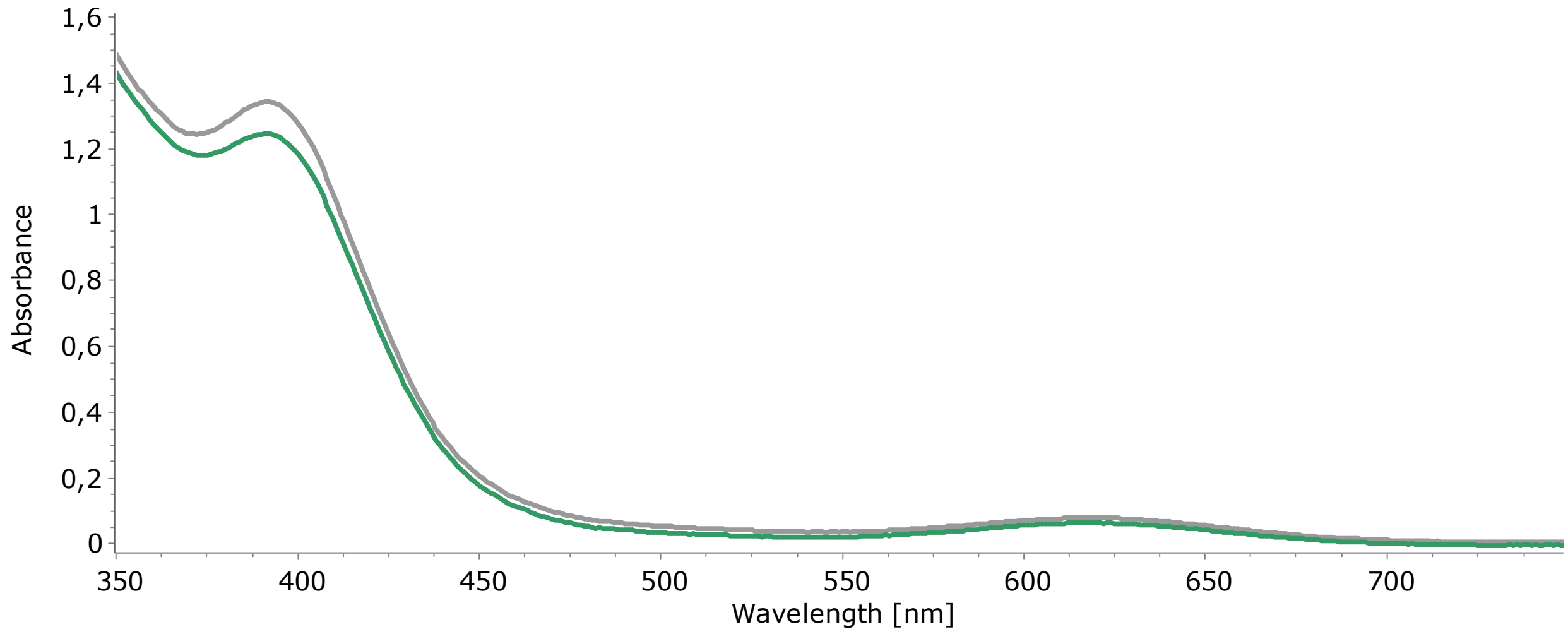

Figure S54. UV-Vis spectra of **[3c][CF_3SO_3]$_2$** in H_2O at t = 0 h (grey) and 72 h (green).

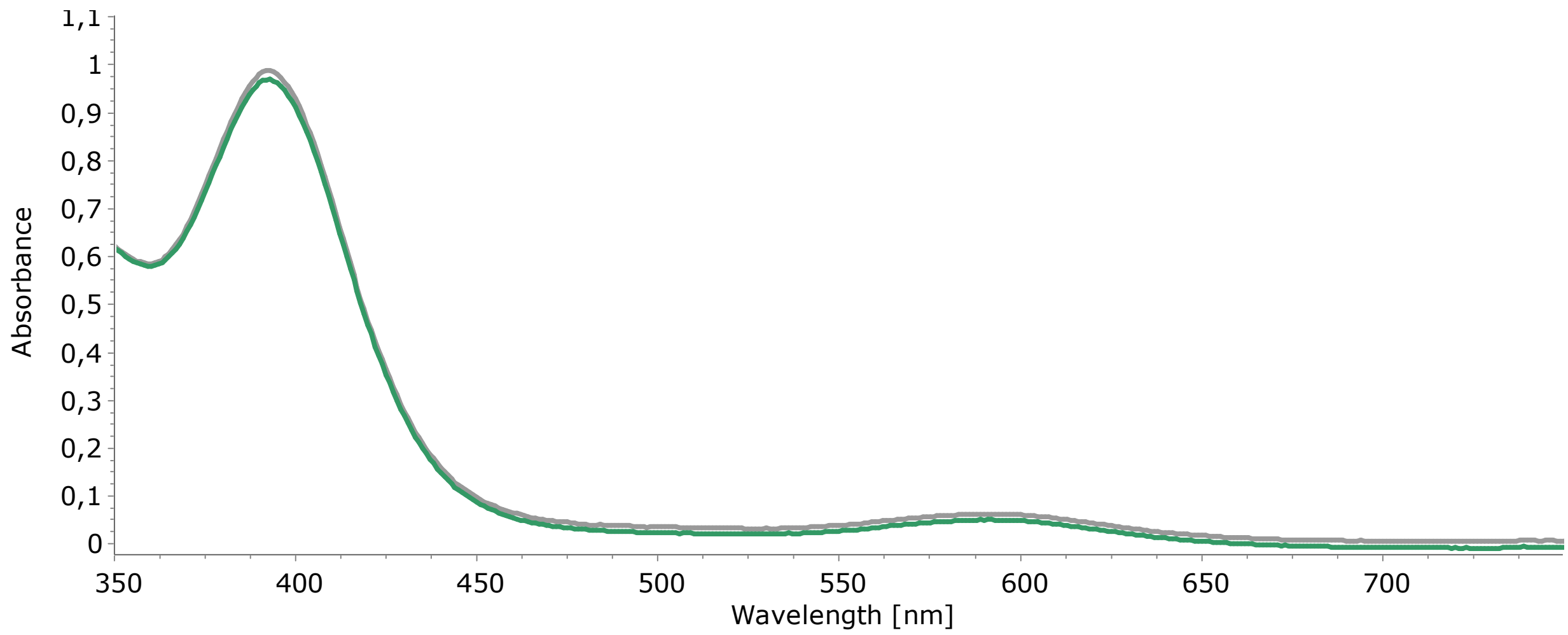

Figure S55. IC_{50} plots showing the inhibitory effects of the metal complexes on the MRC5 healthy cell line. Data represent the mean ± SD of three independent experiments performed in triplicate.

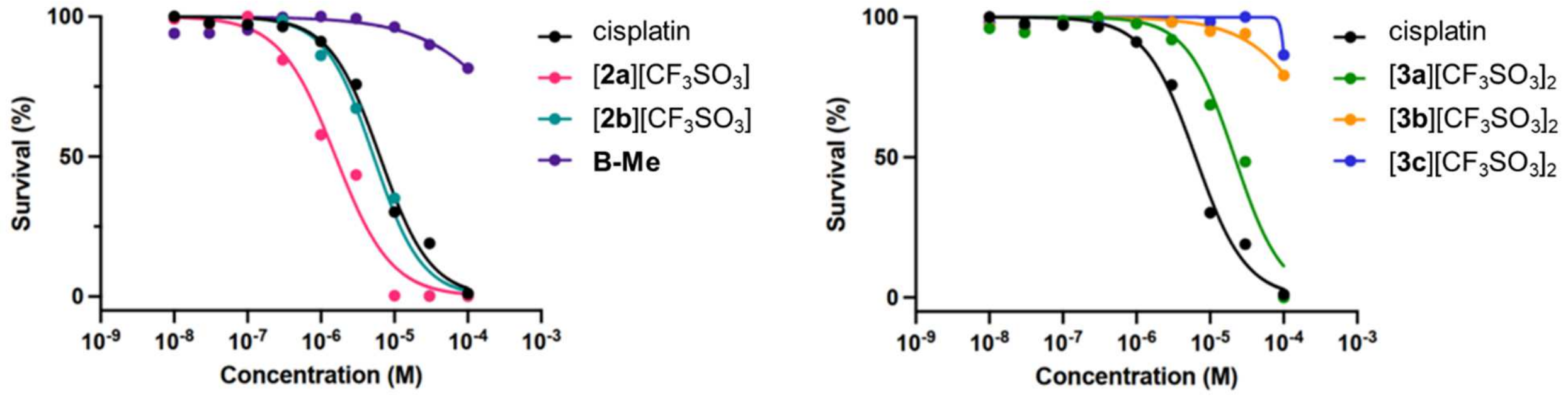

Figure S56. IC_{50} plots showing the inhibitory effects of the metal complexes on the U87 glioblastoma cell line. Data represent the mean ± SD of three independent experiments performed in triplicate.

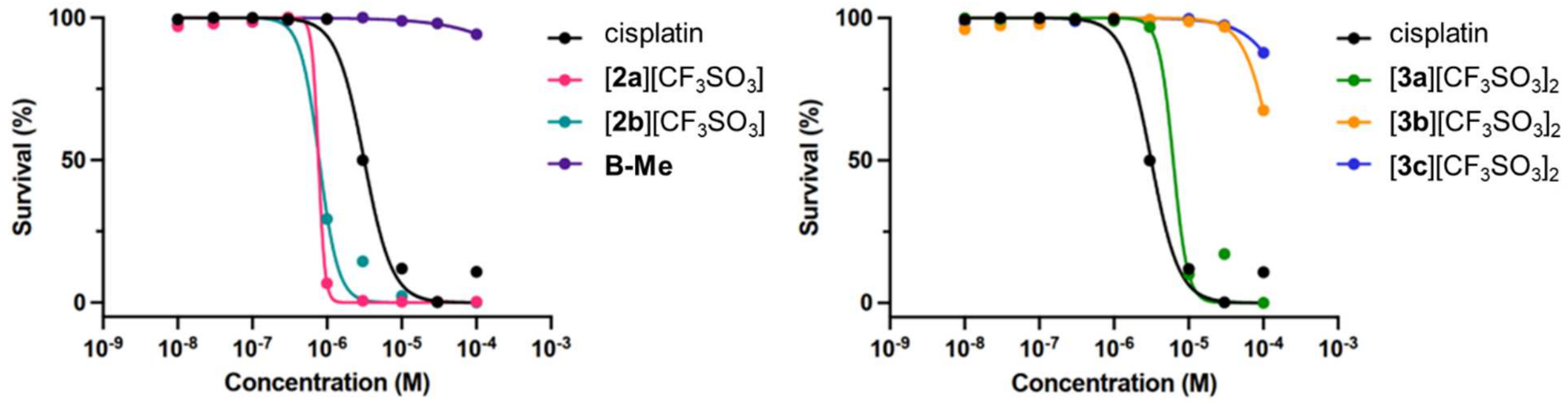

Figure S57. IC_{50} plots showing the inhibitory effects of the metal complexes on the A549 lung cancer cell line. Data represent the mean ± SD of three independent experiments performed in triplicate.

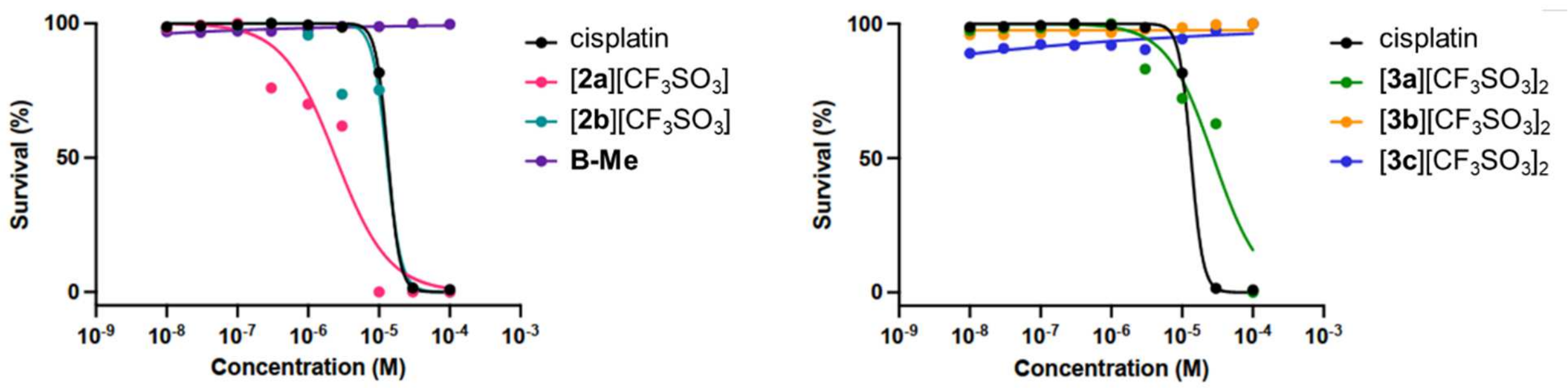

Figure S58. IC_{50} plots showing the inhibitory effects of the metal complexes on the A2780 ovarian cancer cell line. Data represent the mean ± SD of three independent experiments performed in triplicate.

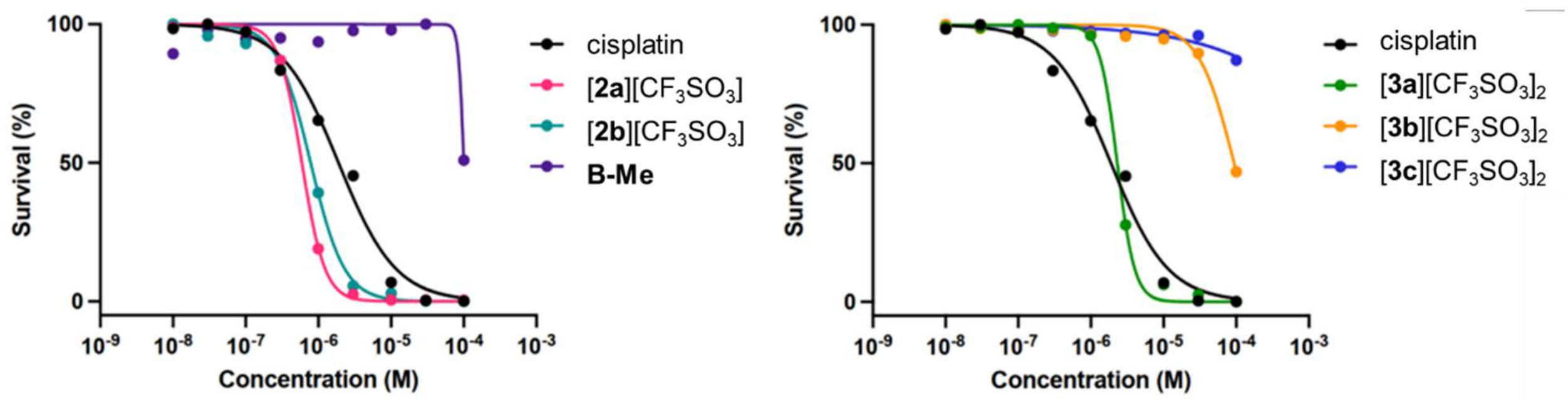

Table S9. Percentage of ^{56}Fe accumulation in the mitochondrial fraction relative to whole-cell samples after 24 h incubation with 4 µM of the complexes (untreated cells served as the control), calculated from ^{56}Fe quantification by ICP-MS. Values are reported as the mean of two replicates ± standard deviation.

	**% Fe in mitochondria**
**Control**	13 ± 11
**[2a][CF_3SO_3]**	3.7 ± 1.6
**[2b][CF_3SO_3]**	3.0 ± 1.6

Figure S59. Evaluation of intracellular ROS production. Representative fluorescence microscopy images of A549 (a) and U87 (b) cells stained with H_2DCFDA following treatment with **[2a][CF_3SO_3]**, **[2b][CF_3SO_3]**, and **[3a][CF_3SO_3]$_2$** (second, third and fourth columns, respectively; untreated control cells are shown in the first column). Cells were treated for 24 h with compounds at concentrations corresponding to their IC_{50} values determined after 48 h exposure. Cells were then incubated for 30 min with 20 µM H_2DCFDA. Upon cleavage of the acetate groups by intracellular esterases and oxidation, non-fluorescent H_2DCFDA is converted to highly fluorescent 2',7' dichlorofluorescein (DCF, green). Representative merged fluorescence and bright-field images are shown below the corresponding fluorescence image. Scale bars = 50 µm.

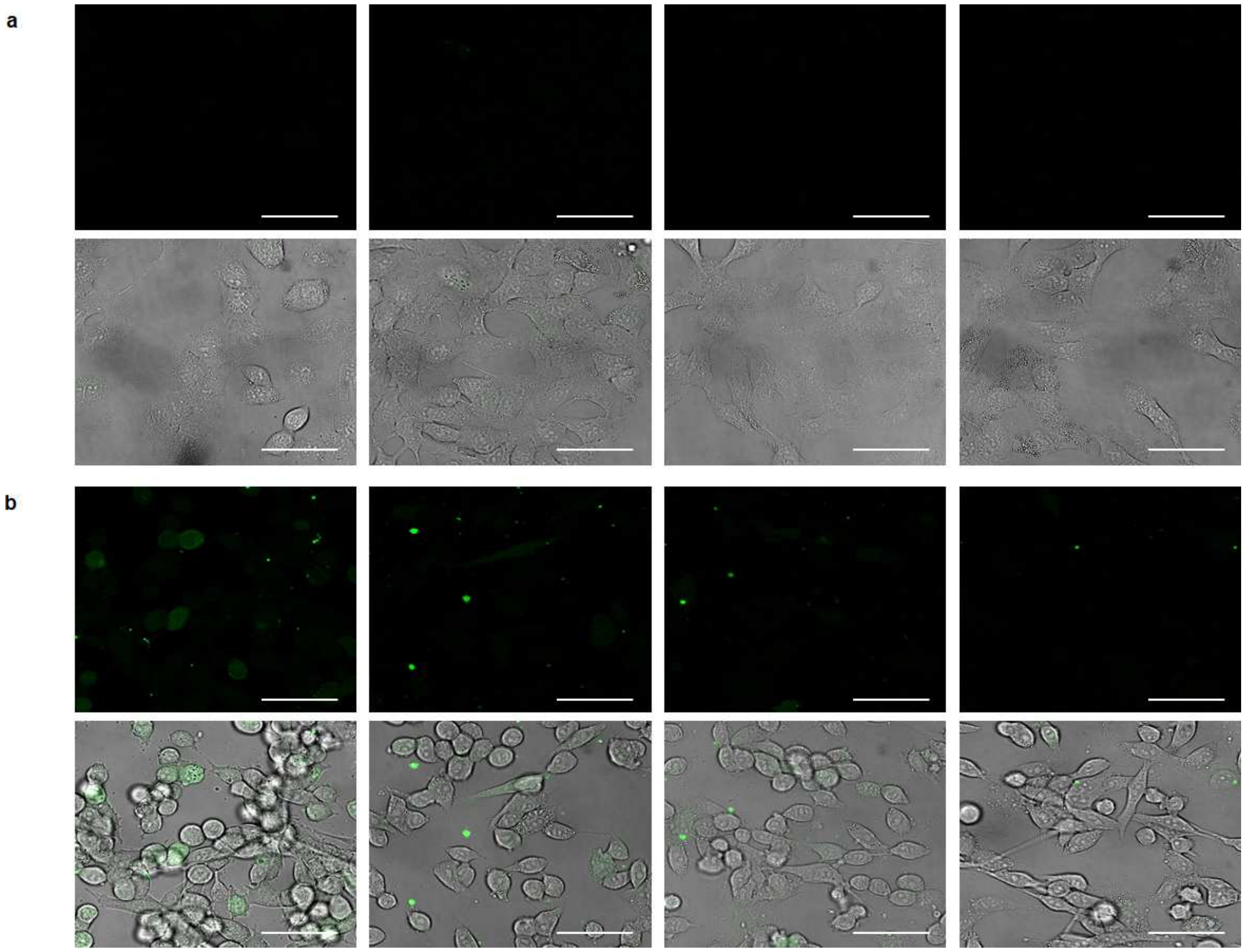

Figure S60. DNA binding studies performed by ethidium bromide (EB) displacement titration. F% = 100*F/F_0, where F_0 is the fluorescence intensity in the absence of metal complex and F is corrected for dilution effects. C_{DNA} = 1.37×10^{-4} M, C_{EB} = 5.94×10^{-5} M, NaCl 0.1 M, sodium cacodylate 0.01 M, pH 7.0, 25.0 °C, λ_{ex} = 520 nm, λ_{em} = 590 nm. A blank experiment was performed by adding DMSO at the same v/v% used in the titrations.

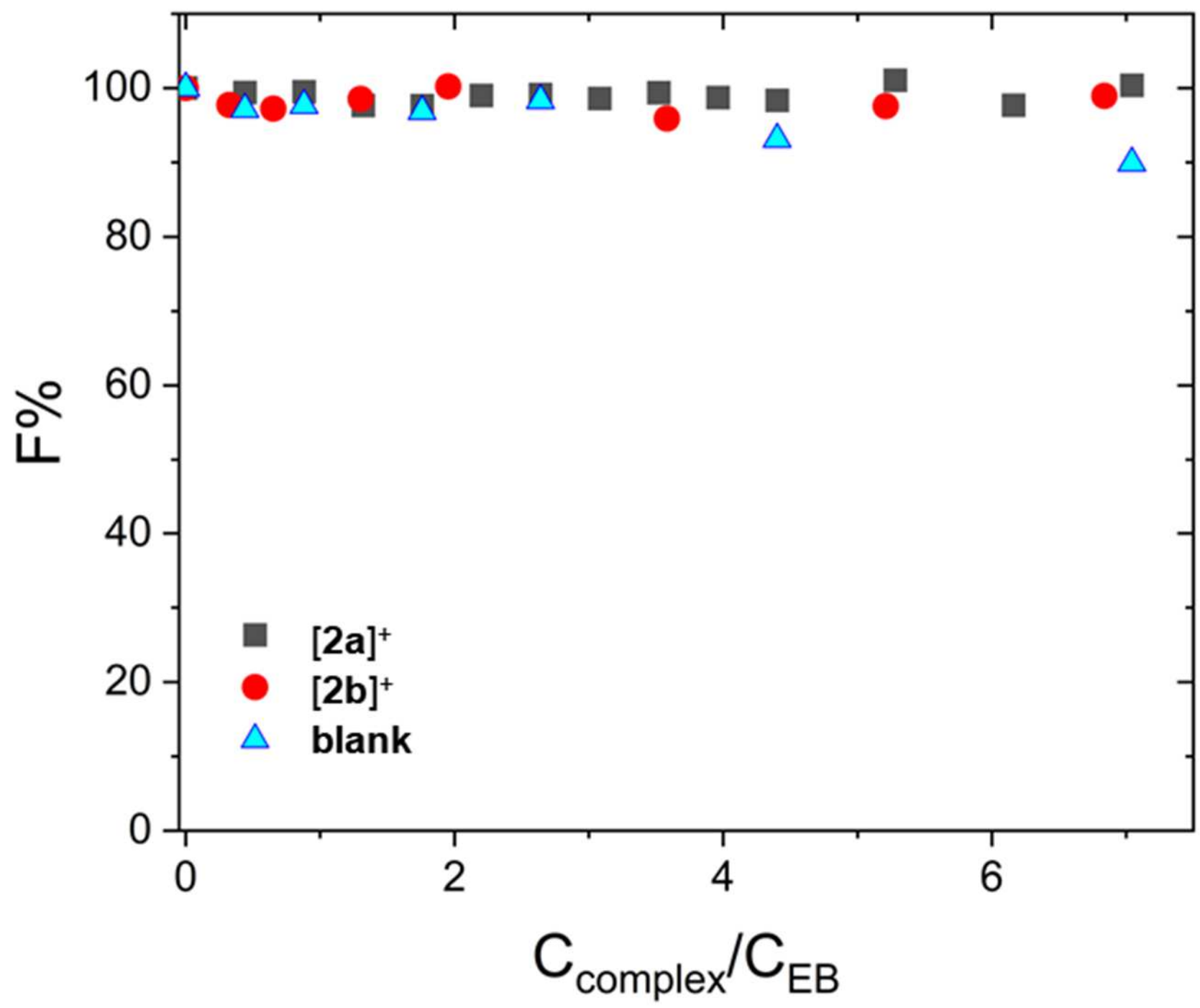

Figure S61. BSA binding studies: spectrofluorimetric binding isotherm for the titration of the protein with **[2a][CF$_3$SO$_3$]** and **[3a][CF$_3$SO$_3$]$_2$**. ΔF = F – $\varphi_{BSA}C_{BSA}$, where φ_{BSA} = F_0/C^0_{BSA}. C_{BSA} = 8.61 × 10^{-7} M for **[2a]$^+$** and 2.00 × 10^{-6} M for **[3a]$^{2+}$**, NaCl 0.1 M, sodium cacodylate 0.01 M, pH 7.0, 25.0 °C, λ_{ex} = 280 nm, λ_{em} = 345 nm.

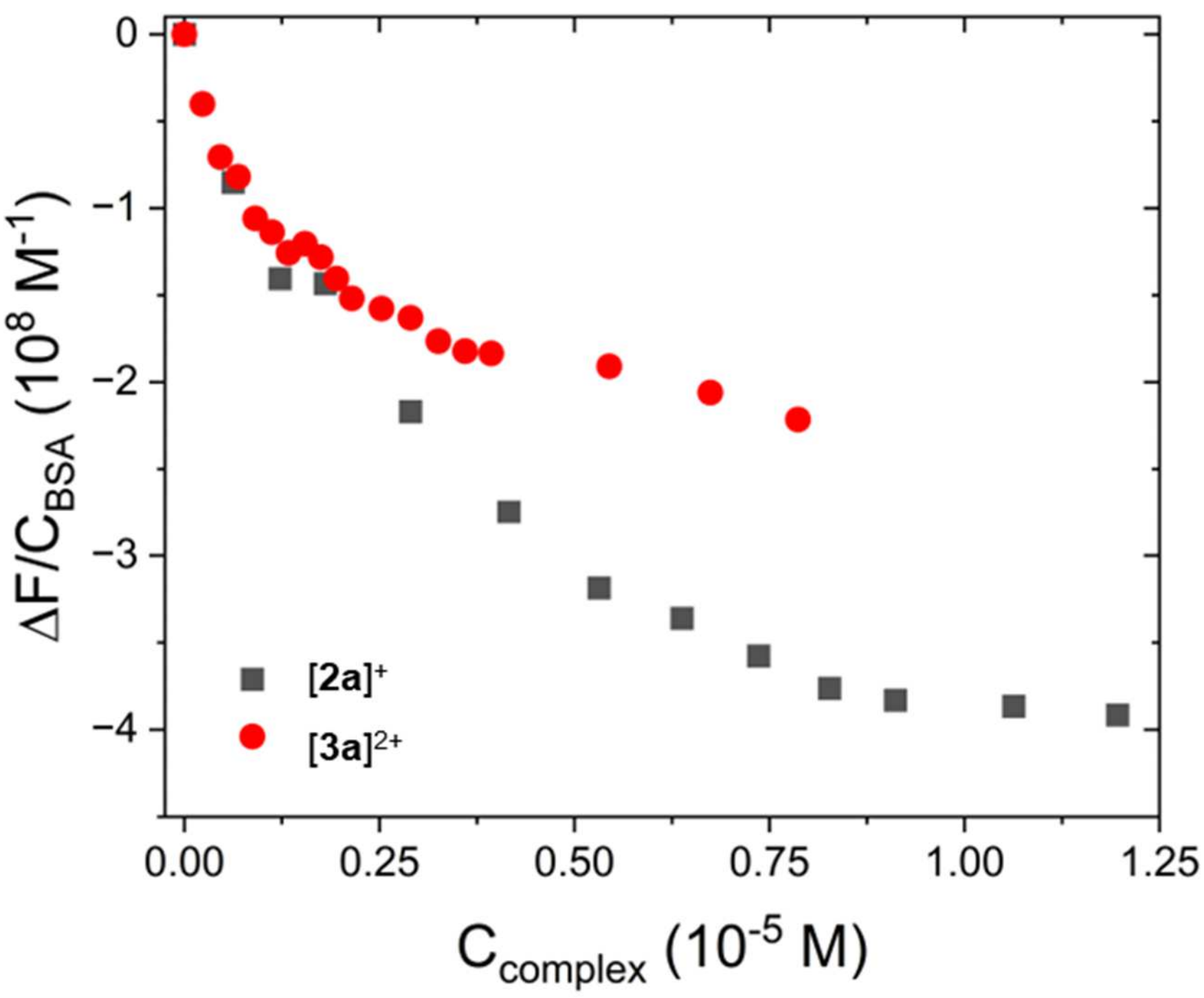

Supplement: Supplementary file 1 [file ic6c03964_si_002.pdf]
